# Supplementary material for: Controlling forward and backward rotary molecular motion on demand
Source: Nat Commun. 2022 Apr 19;13:2124. doi: 10.1038/s41467-022-29820-5 (PMC9019045; doi:10.1038/s41467-022-29820-5)
Supplement: Supplementary file 1 — Supplementary Information [file 41467_2022_29820_MOESM1_ESM.pdf]

SUPPLEMENTARY MATERIALS

Controlling Forward and Backward Rotary Molecular Motion on Demand

*L. Pfeifer[1,†], S. Crespi[1,‡], P. van der Meulen[1], J. Kemmink[1], R. M. Scheek[1], M. Hilbers[2], W. J. Buma[2,3] & B. L. Feringa[1,4]**

* Corresponding author. Email: b.l.feringa@rug.nl (B.L.F.).

[1]Stratingh Institute for Chemistry, University of Groningen, Nijenborgh 4, 9747 AG Groningen, The Netherlands.

[2]Van't Hoff Institute for Molecular Sciences, University of Amsterdam, Science Park 904, 1098 XH, Amsterdam, The Netherlands.

[3]Institute for Molecules and Materials, FELIX Laboratory, Radboud University, Toernooiveld 7c, 6525 ED Nijmegen, The Netherlands.

[4]Zernike Institute for Advanced Materials, University of Groningen, Nijenborgh 4, 9747 AG Groningen, The Netherlands.

[†]Present address: Laboratory of Photonics and Interfaces, Department of Chemistry and Chemical Engineering, École Polytechnique Fédérale de Lausanne, Station 6, Lausanne CH-1015, Switzerland.

[‡]Present address: Department of Chemistry, Ångström Laboratory, Uppsala University, Box 523, 751 20 Uppsala, Sweden.

Table of Contents

1. General Information 3
2. Preparation and Characterization of Compounds 3
   2.1. Upper Half 3
   2.2. Lower Half 5
   2.3. Motor 6
3. X-Ray Diffraction Studies 10
4. NMR Studies 11
   4.1. 2D NMR Studies 11
   *4.2. Photochemical and Thermal Isomerization of **1** 19*
   4.3. Protonation of $\mathbf{1_s}$ 21
   *4.4. Photochemical and Thermal Isomerization of **(1–H)**$^+$ 22*
   4.5. Exchange Spectroscopy (EXSY) 23
5. UV-vis Absorption Studies 30
   5.1. Steady-State UV-vis Absorption Spectra of $\mathbf{1_s}$, at PSS and During Isomerization 30
   5.2. UV-vis Spectra of $\mathbf{1_s}$ in Different Solvents 32
   5.3. Eyring Study on $\mathbf{1_m}$ 33
   *5.4. Fatigue Study on **1** 34*
   5.5. Protonation of $\mathbf{1_s}$ 34
   5.6. Steady-State UV-vis Absorption Spectra of $\mathbf{(1–H)_s^+}$, at PSS and During Isomerization 35
   5.7. UV-vis Spectra of $\mathbf{(1–H)_s^+}$ in Different Solvents 36
   5.8. Eyring Study on $\mathbf{(1–H)_m^+}$ 37
   *5.9. Fatigue Study on **(1–H)**$^+$ 38*
6. Kamlet-Taft Analysis 39
7. Computational Analysis 40
8. NMR Spectra 50
9. Supplementary References 57

1. General Information

Reagents were purchased from Sigma Aldrich, Acros or TCI Europe and were used as received. Solvents were reagent grade and used without prior water removal unless otherwise indicated. Anhydrous solvents were obtained from an MBraun SPS-800 solvent purification system or directly bought from Acros. Solvents were degassed by purging with N_2 for a minimum of 30 min or by three freeze-pump-thaw cycles. In the case of samples for UV-vis absorption studies HBF_4 was handled inside an MBraun glove box as it was observed that doing so under ambient conditions can lead to the sample solutions turning cloudy.

Flash column chromatography was performed on a Büchi Reveleris purification system using Büchi silica cartridges. Thin layer chromatography was carried out on aluminum sheets coated with silica gel 60 F254 (Merck). Compounds were visualized with a UV lamp and/or by staining with $KMnO_4$, cerium ammonium molybdate (CAM) or vanillin.

^{1}H and ^{13}C NMR spectra were recorded on a Varian Mercury-Plus 400 or a Bruker Avance 600 NMR spectrometer at 25 °C unless otherwise indicated. Photostationary state (PSS) and protonation studies were performed on a Varian Unity Plus 500 NMR spectrometer. Chemical shifts are given in parts per million (ppm) relative to the residual solvent signal. Multiplets in ^{1}H NMR spectra are designated as follows: s (singlet), d (doublet), t (triplet), q (quartet), p (pentet), m (multiplet), br (broad). High resolution mass spectrometry was performed on an LTQ Orbitrap XL spectrometer. Steady-state UV-vis absorption spectra were recorded on an Agilent 8453 UV-vis Diode Array System, equipped with a Quantum Northwest Peltier controller, in 10 mm quartz cuvettes. Irradiation experiments were performed using fiber-coupled LEDs (M395F3, M470F3, M530F2, M595F2) obtained from Thorlabs Inc. as well as a home-made system using a 528 nm LED (OSRAM Oslon SSL 80 LTCP7P-KXKZ (KZ)).

2. Preparation and Characterization of Compounds

2.1. Upper Half

*5-Methoxy-2-methyl-2,3-dihydro-1H-cyclopenta[a]naphthalen-1-one (**S1**)*

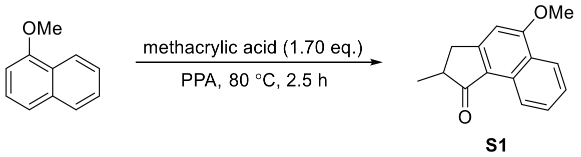

A three-neck flask equipped with a mechanical stirrer was charged with polyphosphoric acid (PPA, 115% H_3PO_4, 80 mL), heated to 80 °C and 1-methoxynaphthalene (13.1 mL, 90.0 mmol) was added over 3 min. After 5 min methacrylic acid (13.0 mL, 153 mmol) was added over 3 min and the resulting mixture was stirred at 80 °C for 2.5 h. The dark red mixture was allowed to cool to room temperature and the reaction was quenched by adding ice. After stirring the mixture overnight, it was extracted with EtOAc and the combined organic layers were dried over $MgSO_4$ and concentrated *in vacuo*. The crude product was purified *via* recrystallization from hot EtOH (250 mL) to obtain **S1** (14.94 g, 66.02 mmol, 73%) as a pale yellow solid. Data is in accordance with literature[1].

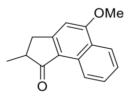

S1

^{1}H NMR (600 MHz, $CDCl_3$) *δ* 9.13 (d, *J* = 8.3 Hz, 1H), 8.25 (d, *J* = 8.4 Hz, 1H), 7.66 (ddd, *J* = 8.2, 7.0, 1.2 Hz, 1H), 7.52 (ddd, *J* = 8.3, 6.8, 1.3 Hz, 1H), 6.77 (s, 1H), 4.08 (s, 3H), 3.44 – 3.38 (m, 1H), 2.84 – 2.64 (m, 2H), 1.36 (d, *J* = 7.3 Hz, 3H); **^{13}C NMR** (151 MHz, $CDCl_3$) *δ* 208.5, 161.8, 159.3, 130.7, 129.4, 126.1, 125.3, 124.0, 123.6, 122.6, 101.6, 56.1, 42.3, 36.0, 17.0.

*5-Hydroxy-2-methyl-2,3-dihydro-1H-cyclopenta[a]naphthalen-1-one (**S2**)*

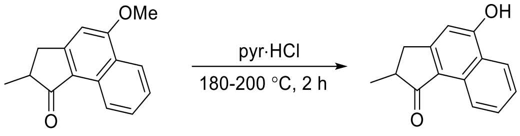

Pyr·HCl (69.5 g, 601 mmol) was melted (m.p. 146 °C) in a flask at 180 °C before **S1** (4.00 g, 17.7 mmol) was added and the resulting mixture was heated to 200 °C. After 2 h the solution was poured onto ice and extracted with EtOAc. The combined organic layers were dried over Na_2SO_4 and concentrated *in vacuo*. Flash column chromatography (SiO_2, dry load on celite, 50–90% EtOAc in pentane) gave **S2** (3.346 g, 15.76 mmol, 89%) as an off-white solid. Data is in accordance with literature[2].

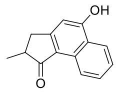

S2

^{1}H NMR (400 MHz, DMSO-*d*$_6$) *δ* 11.41 (s, 1H), 8.96 (d, *J* = 8.3 Hz, 1H), 8.20 (d, *J* = 8.4 Hz, 1H), 7.67 (t, *J* = 7.5 Hz, 1H), 7.53 (t, *J* = 7.7 Hz, 1H), 6.92 (s, 1H), 3.37 (dd, *J* = 18.4, 8.3 Hz, 1H), 2.80 – 2.61 (m, 2H), 1.21 (d, *J* = 7.2 Hz, 3H); **^{13}C NMR** (101 MHz, DMSO-*d*$_6$) *δ* 207.0, 160.4, 159.8, 130.5, 129.1, 125.4, 124.1, 122.9, 122.8, 121.1, 105.6, 41.4, 34.9, 16.6.

*2-Methyl-1-oxo-2,3-dihydro-1H-cyclopenta[a]naphthalen-5-yl acetate (**2**)*

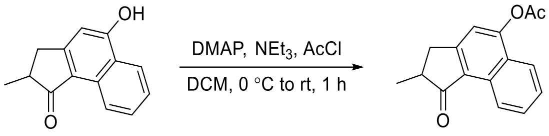

A flame dried Schlenk flask was charged with **S2** (637 mg, 3.00 mmol) and DMAP (18 mg, 0.15 mmol). The mixture was dissolved in DCM (36 mL) before adding NEt_3 (2.09 mL, 15.0 mmol) and cooling to 0 °C. AcCl (533 µL, 7.50 mmol) was added and after stirring at 0 °C for 5 min the mixture was allowed to warm to room temperature. After 1 h the reaction was quenched by adding H_2O and the mixture was extracted with DCM. The combined organic layers were washed with 2 M HCl, H_2O and brine, dried over Na_2SO_4 and concentrated *in vacuo*. Flash column chromatography (SiO_2, dry load on celite, 5–10% EtOAc in pentane) gave **2** (748 mg, 2.94 mmol, 98%) as an off-white solid.

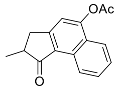

2

^{1}H NMR (600 MHz, $CDCl_3$) *δ* 9.19 (dd, *J* = 8.4, 1.2 Hz, 1H), 7.94 (dd, *J* = 8.3, 1.2 Hz, 1H), 7.70 (ddd, *J* = 8.6, 6.8, 1.5 Hz, 1H), 7.58 (ddd, *J* = 8.4, 6.8, 1.2 Hz, 1H), 7.33 (s, 1H), 3.52 – 3.45 (m, 1H), 2.87 – 2.78 (m, 2H), 2.50 (s, 3H), 1.38 (d, *J* = 7.2 Hz, 3H); **^{13}C NMR** (151 MHz, $CDCl_3$) *δ* 209.1, 169.0, 157.0, 152.4, 130.9, 129.6, 128.4, 127.1, 126.3, 124.4, 121.8, 116.3, 42.6, 35.6, 21.3, 16.7; **HRMS** (ESI pos) *m/z* calcd for $C_{16}H_{14}O_3Na_1$ $[M+Na]^+$ 277.08352, found 277.08349.

2.2. Lower Half

*3,6-Dibromophenanthrene-9,10-dione (**S3**)*

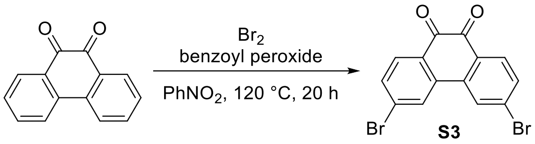

9,10-Phenanthrenedione (10.00 g, 48.03 mmol) and benzoyl peroxide (75 wt% in H_2O, 465 mg, 1.44 mmol) were dissolved in nitrobenzene (50 mL). Bromine (5.44 mL, 106 mmol) was added over 2 min under vigorous stirring. The mixture was heated to 120 °C for 20 h. After cooling to room temperature the formed precipitate was filtered off and washed with pentane. The mother liquor was concentrated *in vacuo* and filtered to give a second batch of product. **S3** (15.57 g, 42.54 mmol, 89%) was obtained as a yellow powder. Data is in accordance with literature[3].

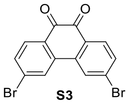

^{1}H NMR (600 MHz, $CDCl_3$) δ 8.12 (d, J = 1.7 Hz, 2H), 8.07 (d, J = 8.3 Hz, 2H), 7.67 (dd, J = 8.3, 1.7 Hz, 2H); **^{13}C NMR** (151 MHz, $CDCl_3$) δ 179.0, 136.1, 133.6, 132.2, 130.0, 127.5.

*3,6-Dibromo-9H-fluoren-9-one (**S4**)*

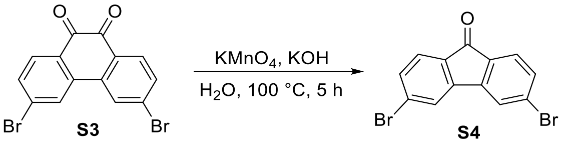

H_2O (58 mL) was added to a mixture of dibromophenanthrene **S3** (14.05 g, 38.39 mmol) and KOH (28.00 g, 499.0 mmol) and the mixture was heated to 100 °C. $KMnO_4$ (32.16 g, 203.5 mmol) was added portion wise over 2 h to the suspension and the final mixture was kept at 100 °C for an additional 3 h. It was then allowed to cool to room temperature and conc. H_2SO_4 was added until neutral pH was obtained. Na_2SO_3 was added until the solution turned light yellow. The solid product was filtered off and after drying in an oven at 90 °C overnight product **S4** (8.27 g, 24.5 mmol, 64%) was obtained as a pale yellow solid. Data is in accordance with literature[3].

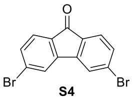

^{1}H NMR (600 MHz, $CDCl_3$) δ 7.67 (d, J = 1.6 Hz, 2H), 7.55 (d, J = 7.8 Hz, 2H), 7.50 (dd, J = 7.8, 1.6 Hz, 2H); **^{13}C NMR** (151 MHz, $CDCl_3$) δ 191.5, 144.9, 133.0, 132.9, 130.0, 125.9, 124.3.

*3,6-Dibromo-9-diazo-9H-fluorene (**3**)*

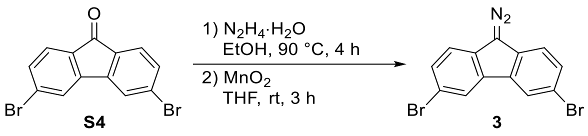

To a mixture of **S4** (3.00 g, 8.88 mmol) and absolute EtOH (90 mL) was added hydrazine monohydrate (50–60% in H_2O, 11.7 mL, 133 mmol) and the resulting light-yellow suspension was heated to 90 °C. After 4 h the mixture was allowed to cool to room temperature and H_2O (10 mL) was added. The green suspension was put in the freezer at –25 °C to allow crystallization overnight. The solids were collected and a second batch was crystallized by adding water (15 mL) to the mother liquor and putting it in the freezer at –25 °C. The collection of both batches yielded a pale green solid as the corresponding hydrazone (1.94 g, 5.51 mmol, 62%). The crude hydrazone (1.67 g, 4.74 mmol) was dissolved in anhydrous THF (53 mL) and MnO_2 (4.12 g, 47.4 mmol) was added. The black suspension was stirred at room temperature for 3 h and then filtered over a plug of celite. The orange solution was concentrated *in vacuo* to yield product **3** (946 mg, 2.70 mmol, 57%) as an orange solid. Note: This compound is not stable under ambient conditions for prolonged periods of time and should be stored at –20 °C. Data is in accordance with literature[3].

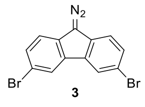

^{1}H NMR (600 MHz, $CDCl_3$) δ 8.03 (s, 2H), 7.52 (d, *J* = 8.5 Hz, 2H), 7.37 (d, *J* = 8.2 Hz, 2H); **^{13}C NMR** (151 MHz, $CDCl_3$) δ 131.9, 131.8, 129.9, 124.5, 120.6, 118.4, 64.0.

2.3. Motor

*1-(3,6-Dibromo-9H-fluoren-9-ylidene)-2-methyl-2,3-dihydro-1H-cyclopenta[a]naphthalen-5-yl acetate (**4$_s$**)*

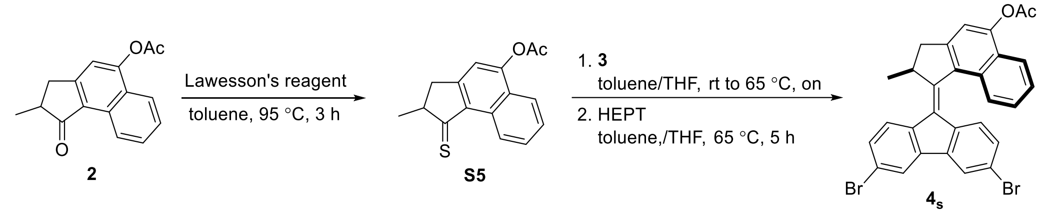

A Schlenk flask was charged with **2** (477 mg, 1.88 mmol), Lawesson's reagent (2.43 g, 6.00 mmol) and toluene (40 mL). The mixture was heated at 95 °C for 3 h, cooled to room temperature and concentrated *in vacuo*. Thioketone **S5** was isolated by flash column chromatography (SiO_2, dry load on celite, 4–7% EtOAc in pentane) and immediately dissolved in toluene (60 mL). A solution of **3** (528 mg, 1.50 mmol) in THF (40 mL) was added and the resulting mixture was stirred at room temperature for 3 h. It was then heated at 65 °C overnight before adding tris(diethylamino)phosphine (HEPT) (1.23 mL, 4.50 mmol), stirring for another 5 h and concentrating *in vacuo*. Flash column chromatography (SiO_2, dry load on celite, 5–15% EtOAc in pentane) gave **4$_s$** (357 mg, 0.64 mmol, 42%) as a yellow-orange solid.

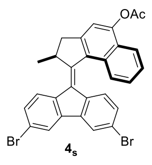

^{1}H NMR (600 MHz, $CDCl_3$) δ 8.02 (dd, *J* = 8.5, 1.0 Hz, 1H), 7.92 (d, *J* = 1.9 Hz, 1H), 7.83 (d, *J* = 1.9 Hz, 1H), 7.80 (d, *J* = 8.4 Hz, 1H), 7.68 (d, *J* = 8.5 Hz, 1H), 7.55 – 7.48 (m, 2H), 7.43 (s, 1H), 7.38 (ddd, *J* = 8.2, 6.8, 1.3 Hz, 1H), 6.94 (dd, *J* = 8.5, 2.0 Hz, 1H), 6.55 (d, *J* = 8.5 Hz, 1H), 4.24 (p, *J* = 6.6 Hz, 1H), 3.57 (dd, *J* = 15.1, 5.6 Hz, 1H), 2.77 (d, *J* = 15.1 Hz, 1H), 2.54 (s, 3H), 1.38 (d, *J* = 6.7 Hz, 3H); **^{13}C NMR** (151 MHz, $CDCl_3$) δ 169.3, 152.2, 148.9, 148.2, 140.7, 140.2, 138.6, 136.0, 133.8, 130.4, 129.4, 128.7, 127.6, 127.5, 127.2, 126.2, 125.9, 125.4, 123.2, 122.5, 122.3,

121.2, 121.1, 116.8, 45.6, 42.1, 21.3, 19.4; **HRMS** (ESI pos) *m/z* calcd for $C_{29}H_{21}Br_2O_2$ $[M+H]^+$ 560.98824, found 560.98742.

*1-(3,6-dicyano-9H-fluoren-9-ylidene)-2-methyl-2,3-dihydro-1H-cyclopenta[a]naphthalen-5-yl acetate (**5$_s$**)*

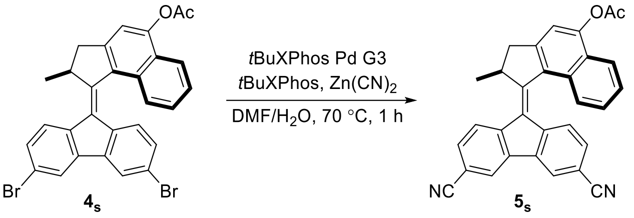

A Schlenk flask was charged with **4$_s$** (248 mg, 0.442 mmol), *t*BuXPhos Pd G3 (53 mg, 66 µmol), *t*BuXPhos (51 mg, 0.12 mmol) and $Zn(CN)_2$ (69 mg, 0.59 mmol). A degassed (three freeze-pump-thaw cycles) mixture of DMF/H_2O (99:1, 6.6 mL) was added and the mixture was heated to 70 °C for 1 h. It was then separated between H_2O and DCM, the aqueous layer was extracted with DCM and the combined organic layers were washed with brine, dried over Na_2SO_4 and concentrated *in vacuo*. Flash column chromatography (SiO_2, dry load on celite, 20–30% EtOAc in pentane) gave **5$_s$** (148 mg, 0.326 mmol, 74%) as an orange solid.

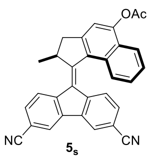

^{1}H NMR (600 MHz, $CDCl_3$) δ 8.15 (dd, *J* = 1.6, 0.6 Hz, 1H), 8.11 – 8.05 (m, 3H), 7.75 (dd, *J* = 8.2, 1.6 Hz, 1H), 7.56 (dtd, *J* = 8.6, 3.5, 1.2 Hz, 2H), 7.49 (s, 1H), 7.39 (ddd, *J* = 8.2, 6.8, 1.3 Hz, 1H), 7.15 (dd, *J* = 8.3, 1.6 Hz, 1H), 6.76 (d, *J* = 8.2 Hz, 1H), 4.33 (p, *J* = 6.6 Hz, 1H), 3.65 (dd, *J* = 15.3, 5.5 Hz, 1H), 2.88 (d, *J* = 15.3 Hz, 1H), 2.55 (s, 3H), 1.45 (d, *J* = 6.8 Hz, 3H); **^{13}C NMR** (151 MHz, $CDCl_3$) δ 169.2, 159.1, 150.1, 150.1, 143.3, 140.4, 138.4, 137.8, 132.9, 131.6, 130.5, 128.3, 128.0, 127.1, 126.6, 126.2, 126.1, 124.5, 123.9, 123.3, 122.8, 119.3, 116.9, 110.3, 110.2, 46.0, 42.4, 21.4, 19.7; **HRMS** (ESI pos) *m/z* calcd for $C_{31}H_{21}N_2O_2$ $[M+H]^+$ 453.15975, found 453.15950.

*(S)-9-(5-hydroxy-2-methyl-2,3-dihydro-1H-cyclopenta[a]naphthalen-1-ylidene)-9H-fluorene-3,6-dicarbonitrile (**S6$_s$**)*

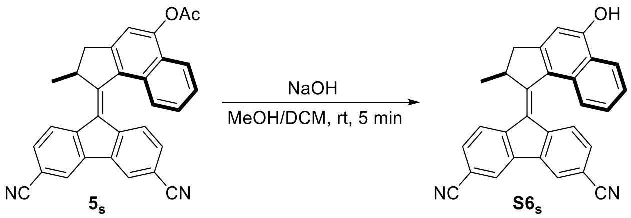

To a solution of **5$_s$** (97 mg, 0.21 mmol) in a MeOH/DCM (2:1, 19 mL) mixture was added NaOH (9.4 mg, 0.24 mmol). After stirring for 5 min at room temperature the reaction was quenched with sat. aq. NH_4Cl and the resulting mixture was extracted with EtOAc, dried over $MgSO_4$ and concentrated *in vacuo*. The product **S6$_s$** (88 mg, 0.21 mmol, quant.) was obtained as a red solid and used in the next reaction without further purification.

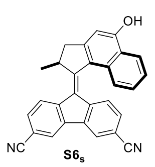

^{1}H NMR (600 MHz, DMSO-*d_6*) *δ* 11.46 (s, 1H), 8.66 (d, *J* = 1.6 Hz, 1H), 8.59 (d, *J* = 1.6 Hz, 1H), 8.31 (d, *J* = 8.4 Hz, 1H), 8.11 (d, *J* = 8.3 Hz, 1H), 7.91 (d, *J* = 8.2 Hz, 1H), 7.50 (t, *J* = 7.6 Hz, 1H), 7.38 (t, *J* = 7.6 Hz, 1H), 7.35 (d, *J* = 8.3 Hz, 1H), 7.31 (d, *J* = 8.4 Hz, 1H), 7.12 (s, 1H), 6.65 (d, *J* = 8.4 Hz, 1H), 4.22 (p, *J* = 6.4 Hz, 1H), 3.53 (dd, *J* = 15.5, 5.5 Hz, 1H), 2.81 (d, *J* = 15.5 Hz, 1H), 1.35 (d, *J* = 6.6 Hz, 3H); **^{13}C NMR** (151 MHz, DMSO-*d_6*) *δ* 161.8, 159.0, 154.0, 142.6, 139.8, 137.0, 136.6, 131.3, 130.4, 129.9, 128.2, 126.1, 125.0, 124.9, 124.9, 124.7, 124.2, 123.9, 123.8, 123.7, 123.6, 119.5, 119.4, 107.9, 107.7, 106.9, 45.1, 41.8, 20.2; **HRMS** (ESI neg) *m/z* calcd for $C_{29}H_{17}N_2O_1$ $[M-H]^-$ 409.13354, found 409.13333.

*(S)-1-(3,6-dicyano-9H-fluoren-9-ylidene)-2-methyl-2,3-dihydro-1H-cyclopenta[a]naphthalen-5-yl trifluoromethanesulfonate (**6_s**)*

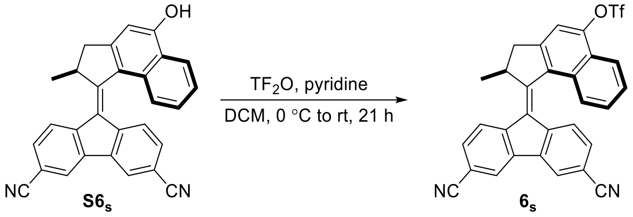

To a solution of **$S6_s$** (86 mg, 0.21 mmol) in DCM (4.5 mL) at 0 °C was added pyridine (25 µL, 0.31 mmol) and triflic anhydride (42 µL 0.25 mmol). The reaction mixture was allowed to warm to room temperature and stirred for 16 h before a second batch of pyridine (13 µL, 0.16 mmol) and triflic anhydride (21 µL, 0.13 mmol) was added. After stirring for another 5 h the mixture was separated between H_2O and DCM. The aqueous layer was extracted with DCM and the combined organic layers were washed with 0.5 M aq. HCl, sat. aq. $NHCO_3$, H_2O and brine, dried over $MgSO_4$ and concentrated *in vacuo*. Flash column chromatography (SiO_2, dry load on celite, 3–6% EtOAc in pentane) gave **6_s** (71 mg, 0.13 mmol, 62%) as an orange solid.

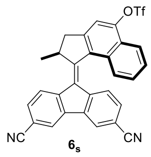

^{1}H NMR (600 MHz, $CDCl_3$) *δ* 8.23 (d, *J* = 8.5 Hz, 1H), 8.14 (d, *J* = 1.5 Hz, 1H), 8.10 – 8.05 (m, 2H), 7.76 (dd, *J* = 8.1, 1.6 Hz, 1H), 7.69 (ddd, *J* = 8.3, 6.8, 1.1 Hz, 1H), 7.67 – 7.62 (m, 2H), 7.48 (ddd, *J* = 8.3, 6.8, 1.2 Hz, 1H), 7.17 (dd, *J* = 8.2, 1.5 Hz, 1H), 6.70 (d, *J* = 8.2 Hz, 1H), 4.38 (p, *J* = 6.6 Hz, 1H), 3.69 (dd, *J* = 15.4, 5.5 Hz, 1H), 2.93 (d, *J* = 15.4 Hz, 1H), 1.46 (d, *J* = 6.7 Hz, 3H); **^{13}C NMR** (151 MHz, $CDCl_3$) *δ* 157.3, 149.1, 148.0, 143.0, 140.1, 138.7, 138.2, 135.5, 131.8, 130.7, 130.6, 129.5, 129.0, 127.9, 127.1, 126.1, 125.6, 124.7, 123.9, 123.4, 122.4, 119.1, 119.1, 118.9 (q, *J* = 320.4 Hz), 116.4, 110.9, 110.8, 46.2, 42.3, 19.4; **^{19}F NMR** (565 MHz, $CDCl_3$) *δ* –73.1; **HRMS** (ESI pos) *m/z* calcd for $C_{30}H_{21}F_3N_3O_3S_1$ $[M+H]^+$ 560.12501, found 560.12531.

*(S)-9-(2-methyl-5-(pyrrolidin-1-yl)-2,3-dihydro-1H-cyclopenta[a]naphthalen-1-ylidene)-9H-fluorene-3,6-dicarbonitrile (**1***$_s$*)*

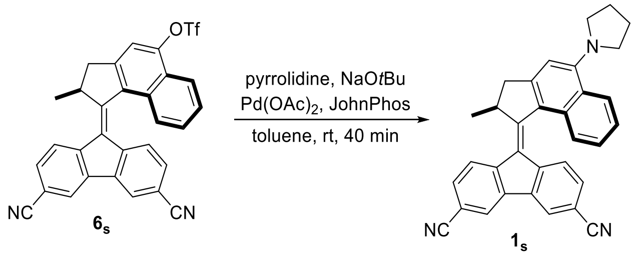

A Schlenk flask was charged with **6**$_s$ (65 mg, 0.12 mmol), NaO*t*Bu (35 mg, 0.36 mmol), $PdOAc_2$ (27 mg, 0.12 mmol) and JohnPhos (72 mg, 0.24 mmol) before adding toluene (2.0 mL) and pyrrolidine (30 µL, 0.36 mmol). After stirring at room temperature for 40 min the mixture was separated between sat. aq. NH_4Cl and DCM. The aqueous layer was extracted with DCM and the combined organic layers were dried over $MgSO_4$ and concentrated *in vacuo*. Flash column chromatography (SiO_2, dry load on celite, 3–10% EtOAc in pentane) gave **1**$_s$ (35 mg, 75 µmol, 63%) as a dark purple solid.

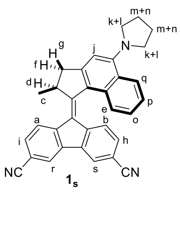

^{1}H NMR (600 MHz, $CDCl_3$) *δ* 8.28 (d, *J* = 8.6 Hz, 1H, H_q), 8.18 (d, *J* = 1.6 Hz, 1H, H_r), 8.11 (d, *J* = 1.6 Hz, 1H, H_s), 8.07 (d, *J* = 8.3 Hz, 1H, H_a), 7.71 (dd, *J* = 8.2, 1.6 Hz, 1H, H_i), 7.46 (d, *J* = 8.3 Hz, 1H, H_e), 7.40 – 7.32 (m, 1H, H_p), 7.29 (d, *J* = 7.7 Hz, 1H, H_o), 7.21 (dd, *J* = 8.3, 1.6 Hz, 1H, H_h), 6.92 (d, *J* = 8.3 Hz, 1H, H_b), 6.76 (s, 1H, H_j), 4.26 (p, *J* = 6.5 Hz, 1H, H_d), 3.85 (q, *J* = 9.1 Hz, 2H, $H_{k/l}$), 3.70 (t, *J* = 8.2 Hz, 2H, $H_{k/l}$), 3.54 (dd, *J* = 15.2, 5.6 Hz, 1H, H_f), 2.76 (d, *J* = 15.2 Hz, 1H, H_g), 2.22 – 2.18 (m, 2H, $H_{m/n}$), 2.05 – 2.01 (m, 2H, $H_{m/n}$), 1.44 (d, *J* = 6.7 Hz, 3H, H_c); **^{13}C NMR** (151 MHz, $CDCl_3$) *δ* 161.8 (C_{quart}), 153.8 (C_{quart}), 153.3 (C_{quart}), 143.4 (C_{quart}), 140.7 (C_{quart}), 137.1 (C_{quart}), 136.4 (C_{quart}), 132.1 (C_{quart}), 130.9 (C_i), 129.9 (C_h), 127.7 (C_o), 127.0 (C_e), 126.6 (C_q), 125.6 (C_b), 125.0 (C_{quart}), 125.0 (C_{quart}), 123.8 (C_r), 123.7 ($C_{a/p}$), 123.6 ($C_{a/p}$), 123.1 (C_s), 122.9 (C_{quart}), 120.0 (C_{quart}), 120.0 (C_{quart}), 108.1 (C_{quart}), 108.0 (C_{quart}), 104.7 (C_j), 53.2 (C_{k+l}), 45.1 (C_d), 42.2 (C_{f+g}), 26.2 (C_{m+n}), 20.9 (C_c); **HRMS** (ESI pos) *m/z* calcd for $C_{33}H_{26}F_3N_3$ $[M+H]^+$ 464.21158, found 464.21186.

A sample of **(1–H)**$_s^+$ for characterization was prepared by adding an excess of HBF_4 to a solution of **1**$_s$ in MeCN-*d$_3$*.

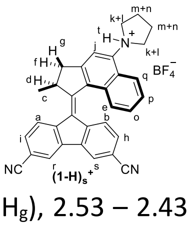

^{1}H NMR (600 MHz, MeCN-*d$_3$*) *δ* 9.49 (bs, 1H, H_t), 8.34 (dd, *J* = 1.6, 0.6 Hz, 1H, H_r), 8.27 (dd, *J* = 1.6, 0.7 Hz, 1H, H_s), 8.21 (m, 2H, H_q and H_a), 7.97 (s, 1H, H_j), 7.86 (dd, *J* = 8.2, 1.6 Hz, 1H, H_i), 7.83 – 7.75 (m, 2H, H_p and H_e), 7.56 – 7.47 (m, 1H, H_o), 7.18 (dd, *J* = 8.3, 1.6 Hz, 1H, H_h), 6.69 (d, *J* = 8.3 Hz, 1H, H_b), 4.40 (p, *J* = 6.7 Hz, 1H, H_d), 4.29 – 4.18 (m, 2H, $H_{k/l}$), 3.95 – 3.84 (m, 2H, $H_{k/l}$), 3.68 (dd, *J* = 15.4, 5.5 Hz, 1H, H_f), 2.93 (d, *J* = 15.8 Hz, 1H, H_g), 2.53 – 2.43 (m, 2H, $H_{m/n}$), 2.43 – 2.33 (m, 2H, $H_{m/n}$), 1.38 (d, *J* = 6.8 Hz, 3H, H_c); **^{13}C NMR** (151 MHz, MeCN-*d$_3$*) *δ* 158.5 (C_{quart}), 150.3 (C_{quart}), 143.8 (C_{quart}), 141.1 (C_{quart}), 139.8 (C_{quart}), 139.4 (C_{quart}), 139.3 (C_{quart}), 138.3 (C_{quart}), 132.9 (C_i), 131.3 (C_{quart}), 131.1 (C_{quart}), 130.8 (C_h), 129.5 (C_o), 128.9 ($C_{e/p}$), 128.8 ($C_{e/p}$), 126.7 (C_{quart}), 126.1 (C_b), 125.8 (C_a), 125.4 (C_r), 125.0 (C_s), 122.1 (C_q), 119.9 (C_{quart}), 119.8 (C_{quart}), 118.6 (C_j), 111.5 (C_{quart}), 111.3 (C_{quart}), 61.0 (C_{k+l}), 60.7 (C_{k+l}), 47.0 (C_d), 42.7 (C_{f+g}), 24.8 (C_{m+n}), 24.7 (C_{m+n}), 19.3 (C_c); **^{19}F NMR** (565 MHz, MeCN-*d$_3$*) *δ* –150.6, –151.7.

3. X-Ray Diffraction Studies

In order to demonstrate the overcrowded alkene structure of compound $\mathbf{1_s}$ in the solid-state single crystals were prepared by vapor diffusion of pentane into a saturated solution of $\mathbf{1_s}$ in DCM.

A single-crystal was mounted on a cryoloop and placed in the nitrogen stream (–173.15 °C) of a Bruker-AXS D8 Venture diffractometer. Data collection and processing was carried out using the Bruker APEX3 software suite[4]. A multi-scan absorption correction was applied, based on the intensities of symmetry-related reflections measured at different angular settings (*SADABS*). The structure was solved using *SHELXT*[5] and refinement was performed using *SHELXL*[6]. The hydrogen atoms were generated by geometrical considerations, constrained by idealized geometries and allowed to ride on their carrier atoms with an isotropic displacement parameter related to the equivalent displacement parameter of their carrier atoms. Possible twinning was ruled out using PLATON[7]. No A- or B-level alerts were raised by CheckCIF for the fully refined structure which was subsequently deposited with the Cambridge Crystallographic Data Centre (CCDC 2143227).

Supplementary Figure 1 shows a displacement ellipsoid plot of the fully refined structure and Supplementary Table 1 contains a summary of the crystallographic parameters.

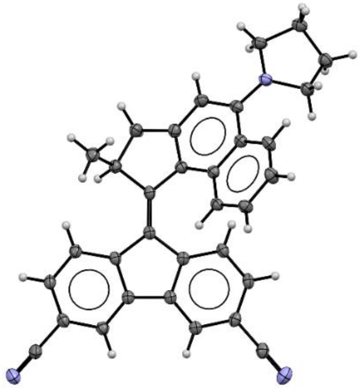

Supplementary Figure 1. Single-crystal X-ray structure of $\mathbf{1_s}$. Displacement ellipsoid plot of single-crystal X-ray structure of $\mathbf{1_s}$ drawn at 50% probability.

Supplementary Table 1. Summary of parameters of single-crystal X-ray structure of $\mathbf{1_s}$.

Nr.	$\mathbf{1_s}$
Name	*(S)-9-(2-methyl-5-(pyrrolidin-1-yl)-2,3-dihydro-1H-cyclopenta[a]naphthalen-1-ylidene)-9H-fluorene-3,6-dicarbonitrile*
Formula	$C_{33}H_{25}N_3$
Molecular Weight	463.56
Crystal System	triclinic
T [K]	100(2)
Space Group	P –1
a [Å]	10.3720(2)
b [Å]	11.0321(3)
c [Å]	11.2795(3)
α [°]	95.7780(10)
β [°]	105.2500(10)
γ [°]	90.9080(10)
V [Å^3]	1237.68(5)
Z	2
D_{calc} [g·cm^{-3}]	1.244
F(0 0 0)	488
h_{min}, h_{max}	–12, 12
k_{min}, k_{max}	–13, 13
l_{min}, l_{max}	–14, 14
μ [mm^{-1}]	0.566
Crystal Size [mm]	0.16 x 0.05 x 0.03
Colour, Shape	clear_dark_red needle
R_{int}	0.0565
ϑ_{min}, ϑ_{max} [°]	4.031, 74.496
Total Reflections (before merge)	23758
Data (I>3 x sigma(I)) [Reflections,Parameters,Restraints]	4987, 326, 0
S (=GooF)	1.034
Min. Residual Density [e/Å^3]	–0.174
Max. Residual Density [e/Å^3]	0.219
Threshold Expression	I>2sigma(I)
R_1	0.0548
wR_2	0.1047

4. NMR Studies

4.1. 2D NMR Spectra

Supplementary Figures 2–5 show 2D NMR spectra of $\mathbf{1_s}$ in $CDCl_3$ recorded on a 600 MHz (^{1}H NMR frequency) Bruker Avance spectrometer.

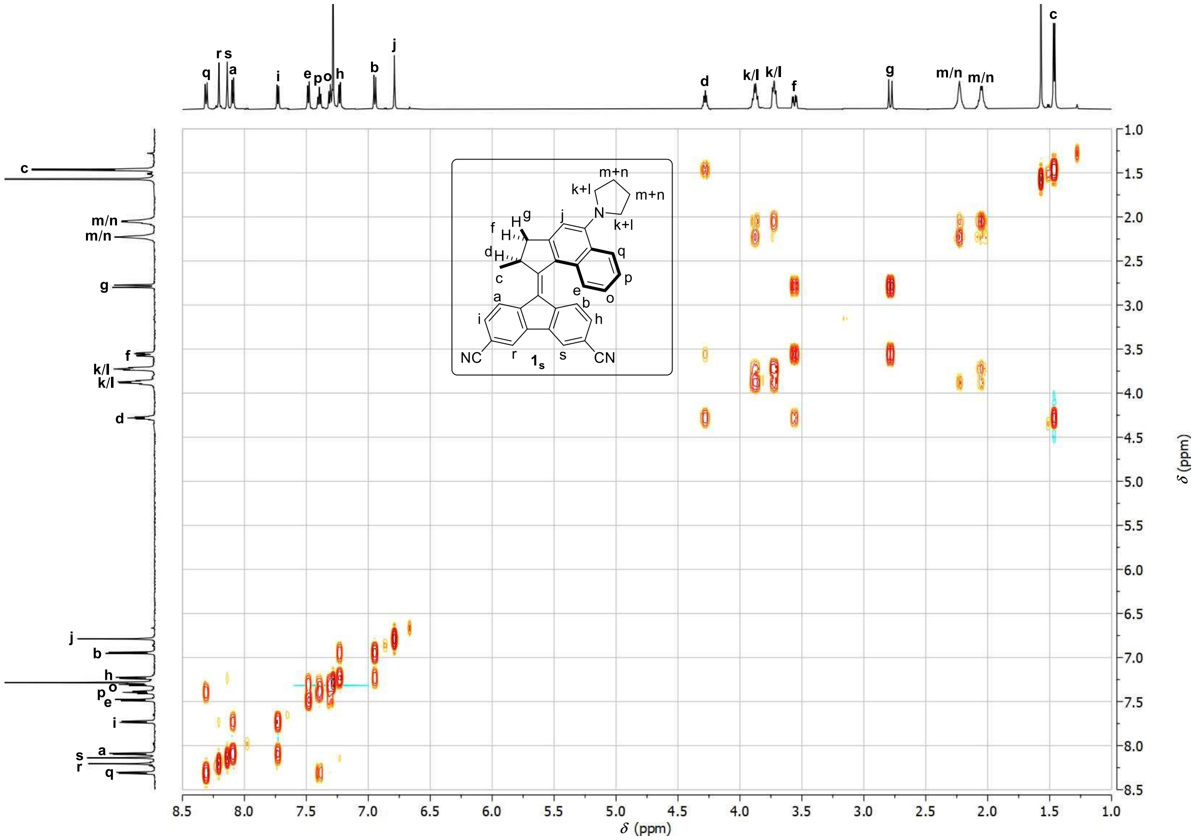

Supplementary Figure 2. COSY spectrum of 1_s. Conditions: 600 MHz, $CDCl_3$, 25 °C.

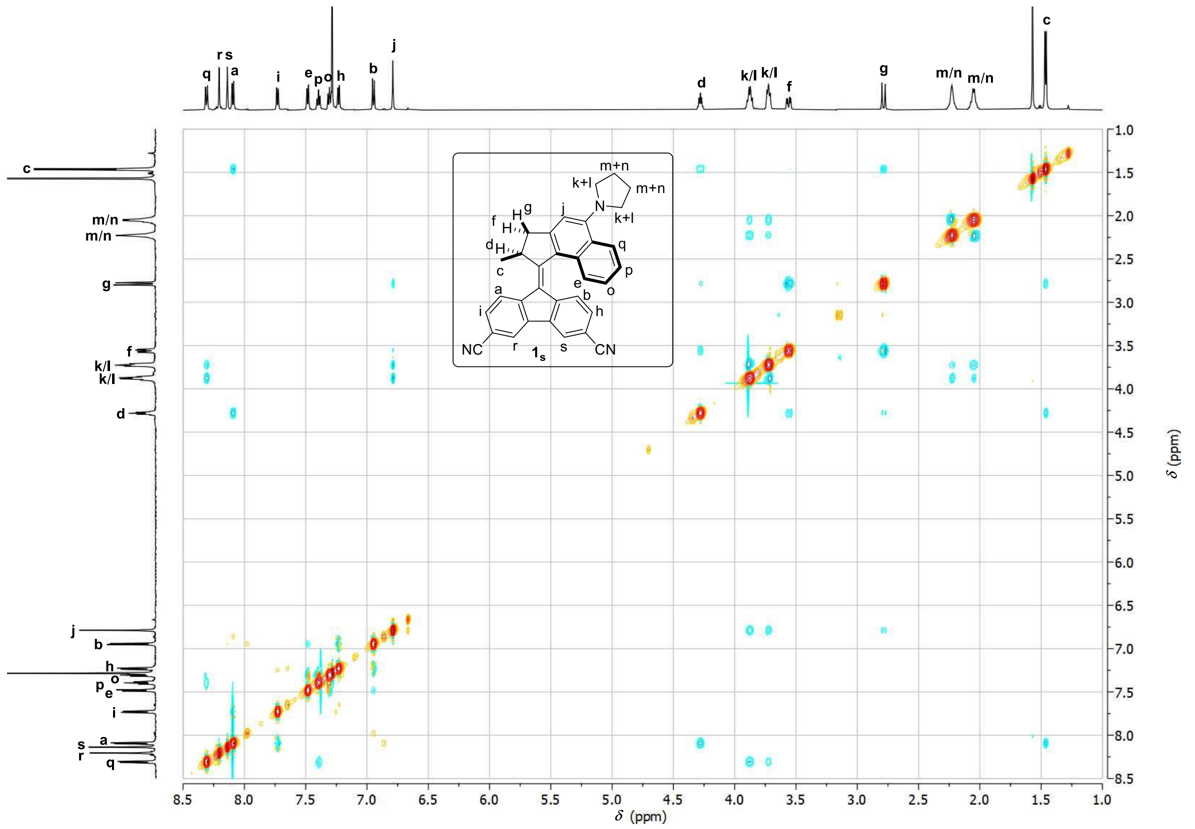

Supplementary Figure 3. 2D NOESY spectrum of 1_s. Conditions: 600 MHz, 300 ms mixing time, $CDCl_3$, 25 °C.

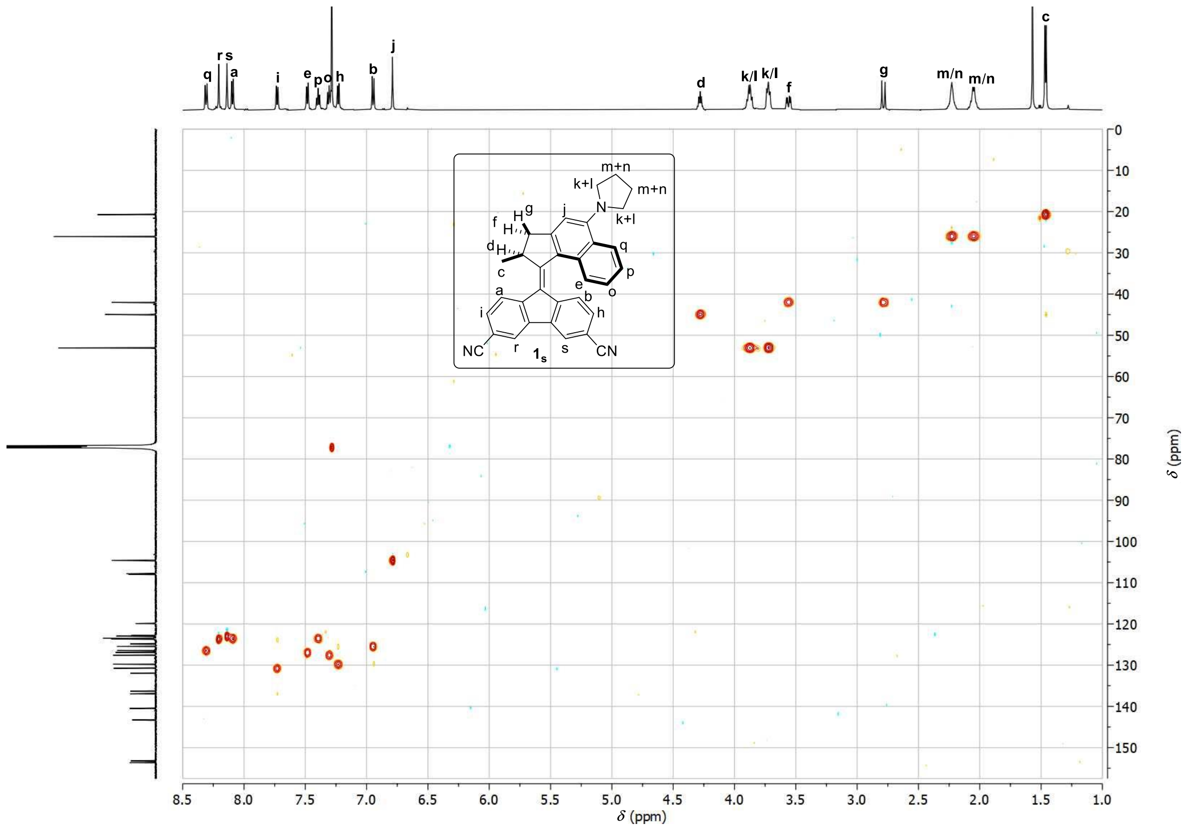

Supplementary Figure 4. HSQC spectrum of 1_s. Conditions: 600 MHz, $CDCl_3$, 25 °C.

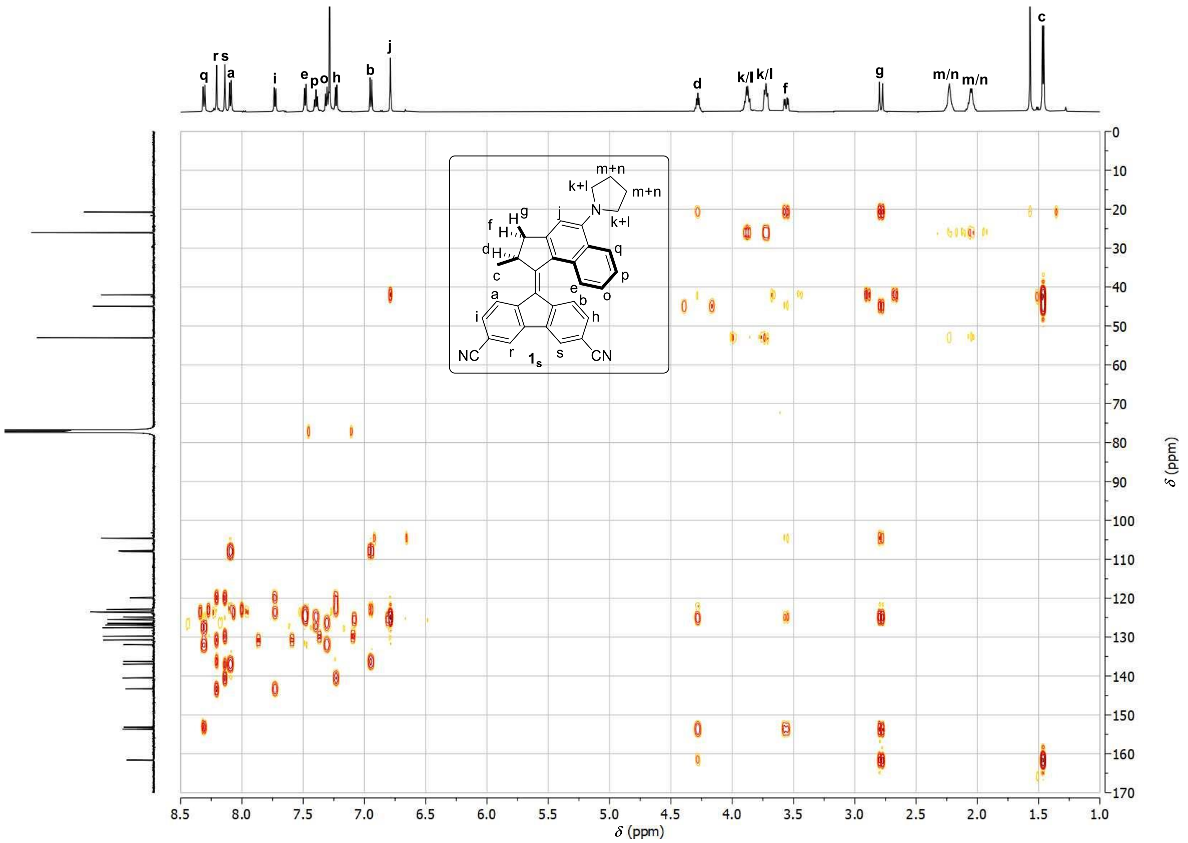

Supplementary Figure 5. HMBC spectrum of 1_s. Conditions: 600 MHz, $CDCl_3$, 25 °C.

Supplementary Figures 6–9 show 2D NMR spectra of **$(1–H)_s^+$** in MeCN-d_3.

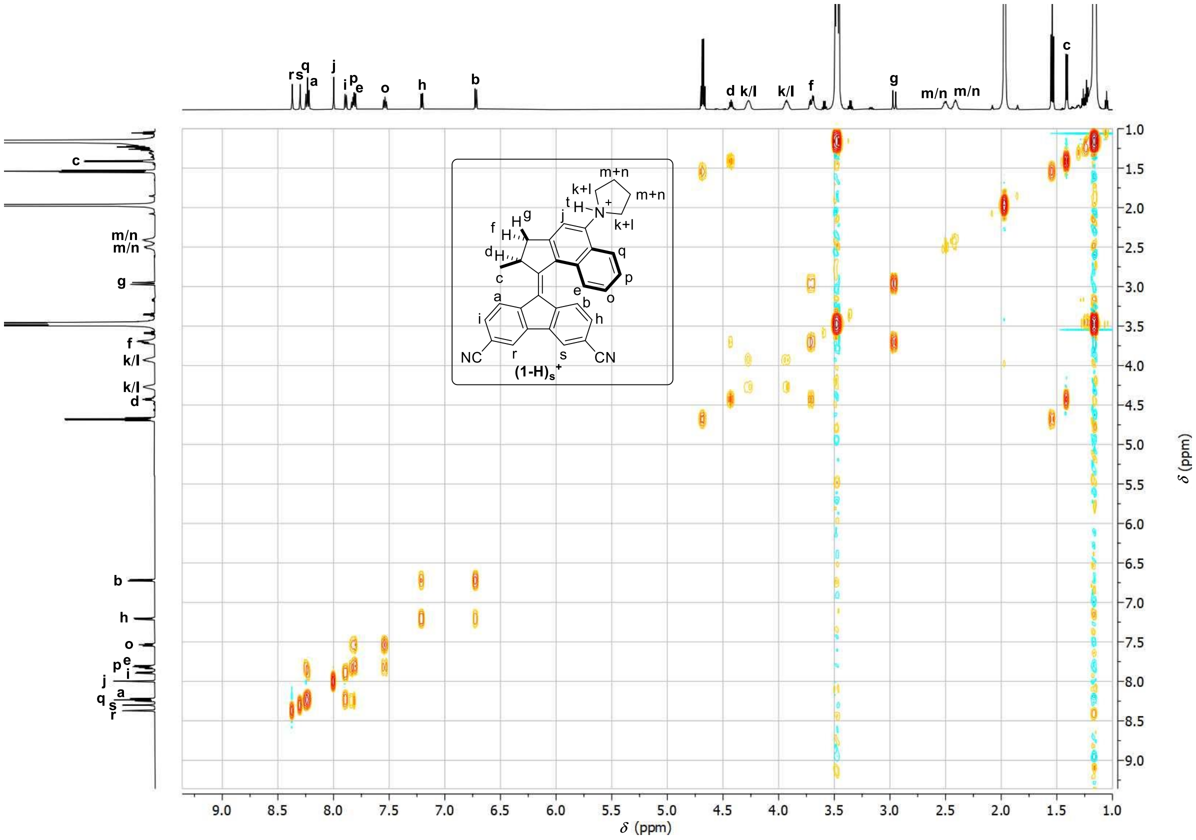

Supplementary Figure 6. COSY spectrum of (1–H)$_s^+$. Conditions: 600 MHz, MeCN-d_3, 25 °C.

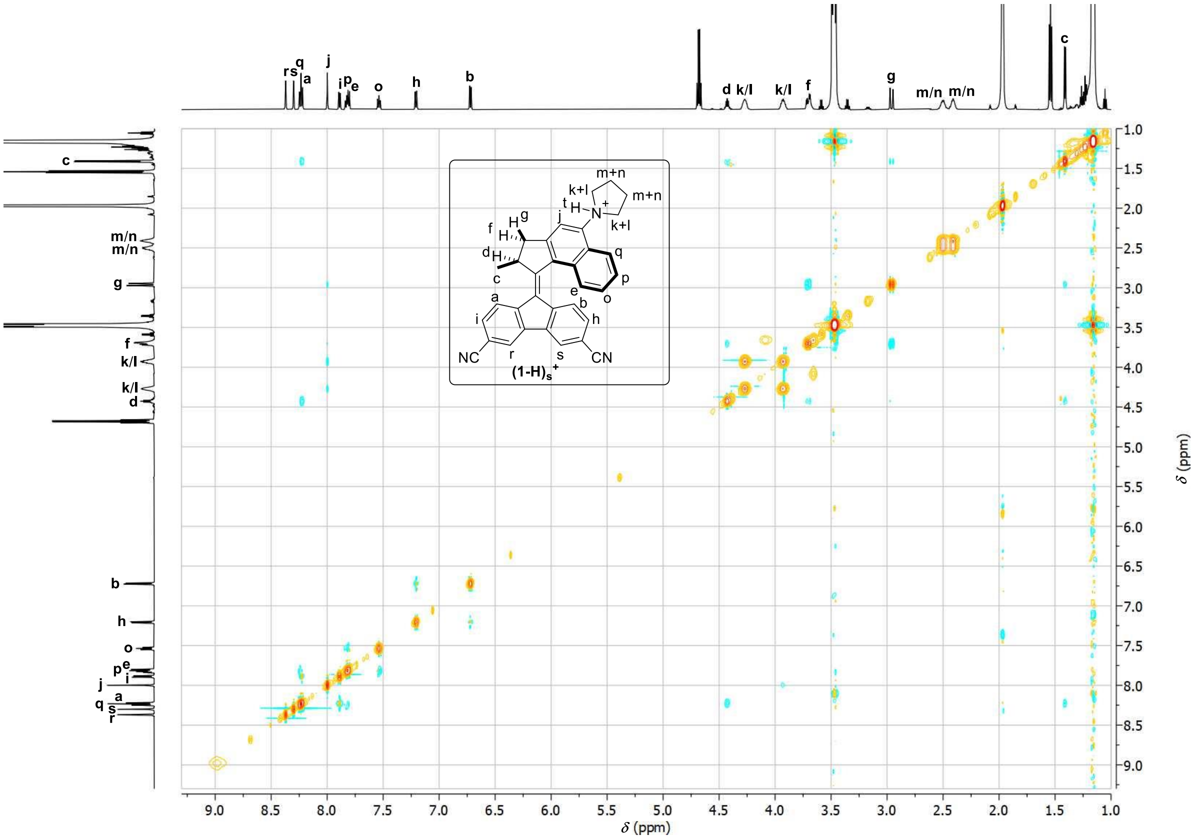

Supplementary Figure 7. 2D NOESY spectrum of $(1\text{–H})_s^+$. Conditions: 600 MHz, 300 ms mixing time, MeCN-d_3, 25 °C.

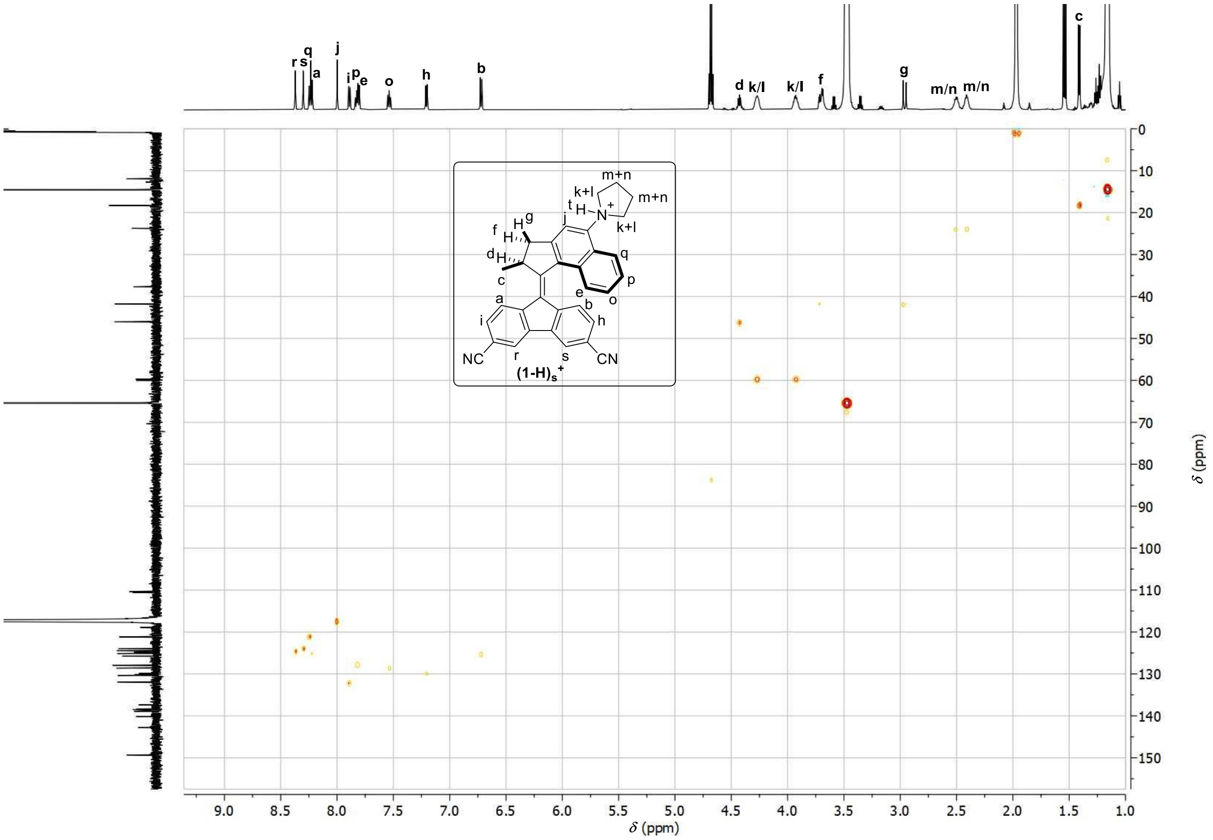

Supplementary Figure 8. HSQC spectrum of $(1–H)_s^+$. Conditions: 600 MHz, MeCN-d_3, 25 °C.

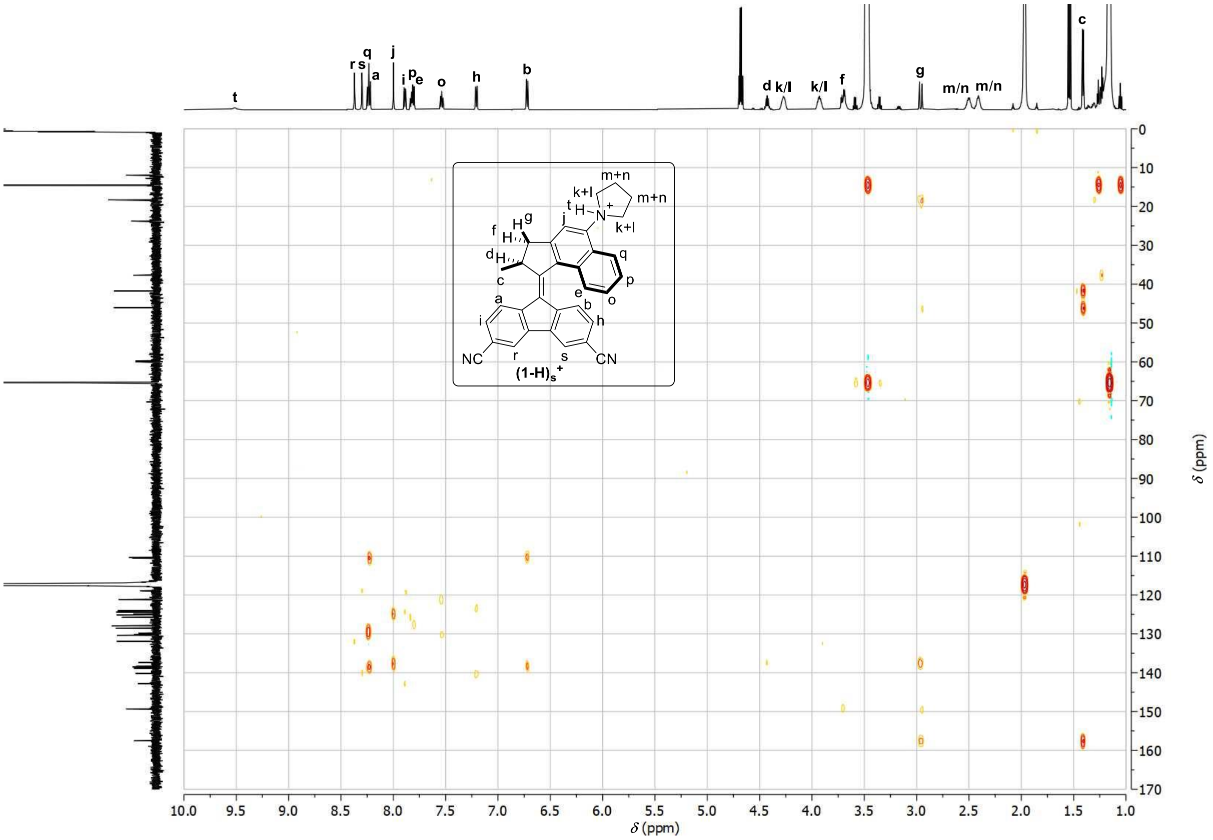

Supplementary Figure 9. HMBC spectrum of (1–H)$_s$$^+$. Conditions: 600 MHz, MeCN-d_3, 25 °C.

4.2. Photochemical and Thermal Isomerization of 1

Solutions of **1**$_s$ (2.5·10^{-3} M) in toluene-d_8 and CD_2Cl_2 were prepared and transferred into NMR tubes which were subsequently fitted with a glass fiber cable for *in situ* irradiation. The samples were placed in a Varian Unity Plus 500 NMR spectrometer and cooled to –59 °C (toluene-d_8) and –85 °C (CD_2Cl_2), respectively. ^{1}H NMR spectra were collected and the solutions were subsequently irradiated with a 530 nm (toluene-d_8) and 470 nm (CD_2Cl_2) LED, respectively, until no further change was observed (PSS). Ratios of metastable:stable isomers at PSS were determined by comparing the integrals of the two signals corresponding to H^d. The samples were then warmed to room temperature for 150 min to allow for complete thermal helix inversion (THI) before recording another set of ^{1}H NMR spectra at –59 °C (toluene-d_8) and –85 °C (CD_2Cl_2), respectively. Spectra stacks are shown in Supplementary Figures 10 and 11 and PSS ratios of metastable:stable isomers are indicated there.

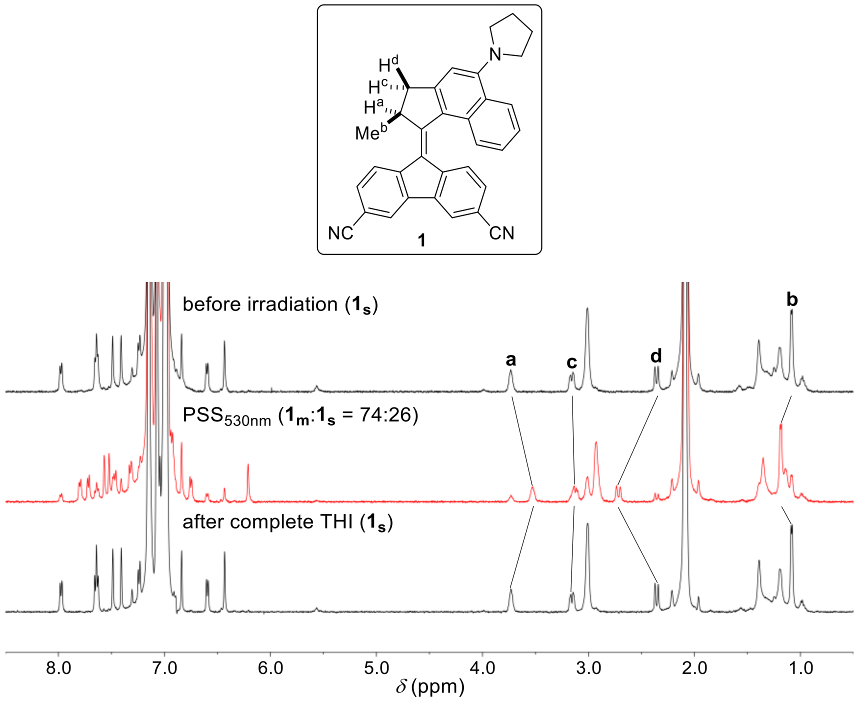

Supplementary Figure 10. 180° rotation of 1 followed by NMR. Stack of ^{1}H NMR spectra of **1** before irradiation with a 530 nm LED (**1**$_s$), at PSS and after completed THI. Conditions: 2.5·10^{-3} M, toluene-*d*$_8$, –59 °C.

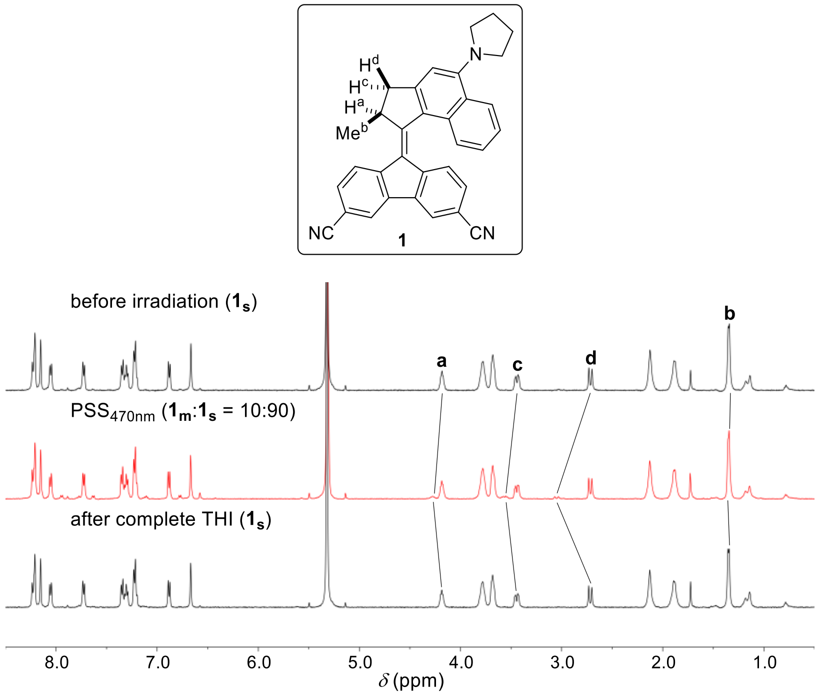

Supplementary Figure 11. 180° rotation of 1 followed by NMR. Stack of ^{1}H NMR spectra of **1** before irradiation with a 470 nm LED (**1**$_s$), at PSS and after completed THI. Conditions: 2.5·10^{-3} M, CD_2Cl_2, –85 °C.

4.3. Protonation of 1_s

Solutions of $\mathbf{1_s}$ ($1.3 \cdot 10^{-3}$ M) in MeCN-d_3 and CD_2Cl_2 were prepared and transferred into NMR tubes which were placed in a Varian Unity Plus 500 NMR spectrometer stabilized at 20 °C. ^{1}H NMR spectra were collected before $HBF_4 \cdot OEt_2$ (1 µL) was added to achieve complete protonation. A second set of ^{1}H NMR spectra was collected before adding NEt_3 (3 µL) and measuring a third set of ^{1}H NMR spectra. Stacks of the spectra collected for both samples are shown in Supplementary Figures 12 and 13.

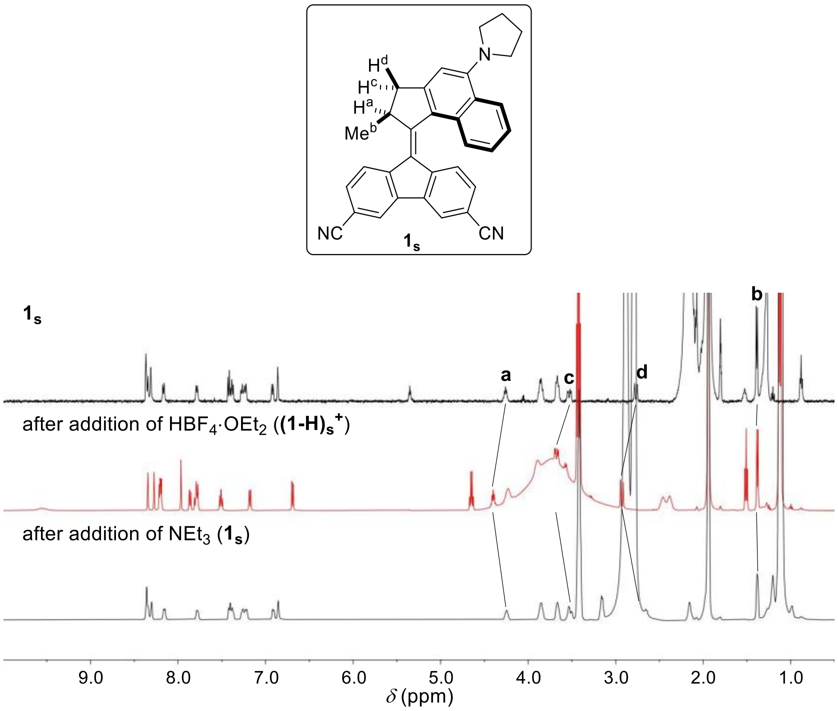

Supplementary Figure 12. Protonation of 1_s and subsequent deprotonation. Stack of ^{1}H NMR spectra of $\mathbf{1_s}$, after addition of $HBF_4 \cdot OEt_2$ ($\mathbf{(1–H)_s^+}$) and after subsequent addition of NEt_3 ($\mathbf{1_s}$). Conditions: $1.3 \cdot 10^{-3}$ M, MeCN-d_3, 20 °C.

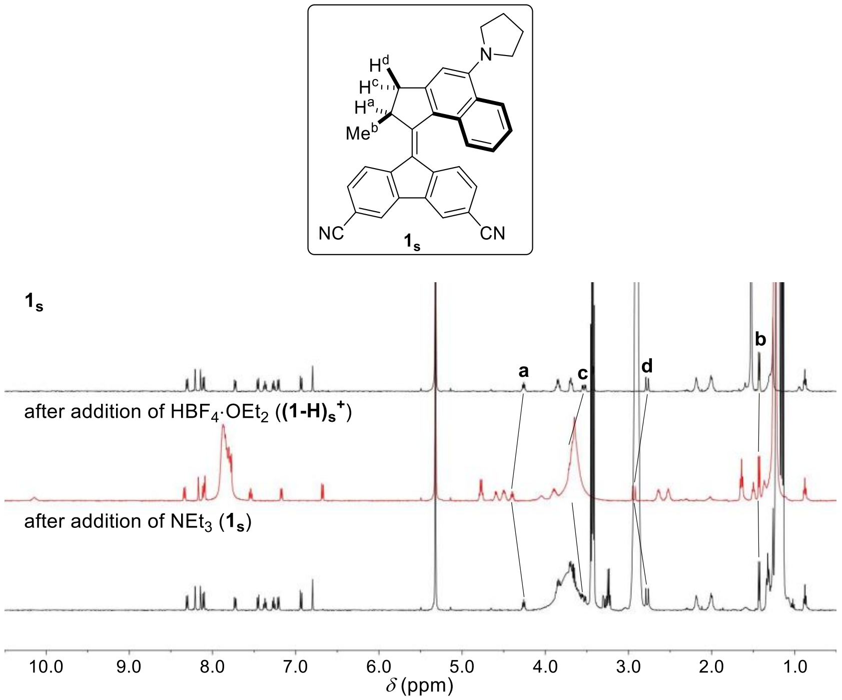

Supplementary Figure 13. Protonation of $\mathbf{1_s}$ and subsequent deprotonation. Stack of ^{1}H NMR spectra of $\mathbf{1_s}$, after addition of $HBF_4{\cdot}OEt_2$ ($\mathbf{(1–H)_s^+}$) and after subsequent addition of NEt_3 ($\mathbf{1_s}$). Conditions: $1.3{\cdot}10^{-3}$ M, CD_2Cl_2, 20 °C.

4.4. Photochemical and Thermal Isomerization of $(1–H)^+$

To a $2.5{\cdot}10^{-3}$ M solution of $\mathbf{1_s}$ in MeCN-d_3 was added $HBF_4{\cdot}OEt_2$ (1.0 µL) before it was transferred into an NMR tube which was subsequently fitted with a glass fiber cable for *in situ* irradiation. The sample was placed in a Varian Unity Plus 500 NMR spectrometer and cooled to –30 °C. A ^{1}H NMR spectrum was collected, and the solution was subsequently irradiated with a 395 nm LED until no further change was observed (PSS). The ratio of metastable:stable isomer at PSS was determined by comparing the integrals of the two signals corresponding to H^d. The sample was then warmed to room temperature for 150 min to allow for complete THI before recording another ^{1}H NMR spectrum at –30 °C. A stack of the collected spectra is shown in Supplementary Figure 14 and the PSS ratio of metastable:stable isomer is indicated there.

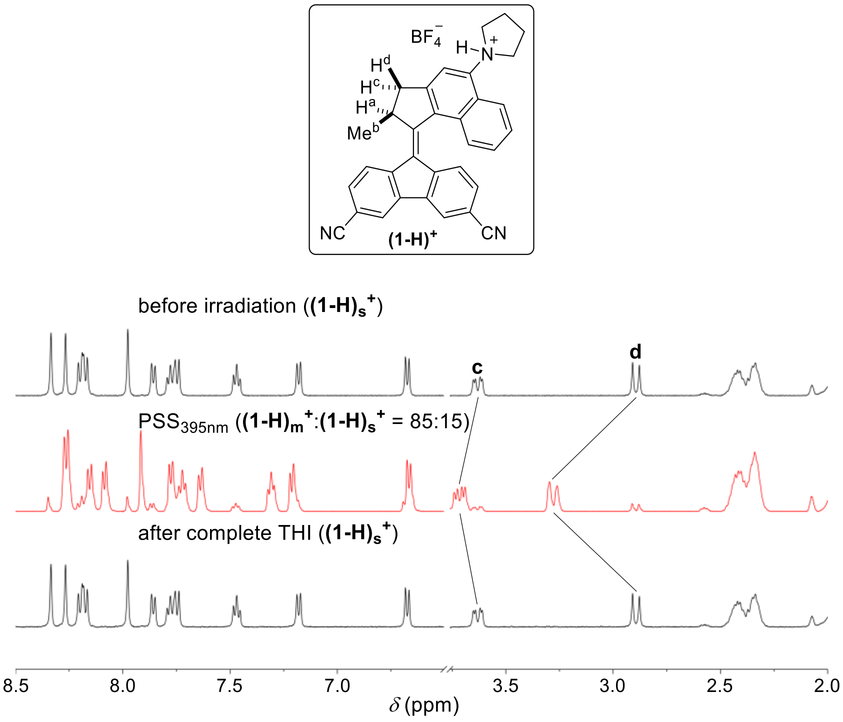

Supplementary Figure 14. 180° rotation of $(1\text{–H})^+$ followed by NMR. Stack of ^{1}H NMR spectra of **$(1\text{–H})^+$** before irradiation with a 395 nm LED **$(1\text{–H})_s^+$**), at PSS and after completed THI. Conditions: $2.5\cdot10^{-3}$ M, MeCN-d_3, –30 °C.

4.5. Exchange Spectroscopy (EXSY)

Exchange reaction

The exchange reaction shown in Supplementary Figure 15 was studied by EXSY.

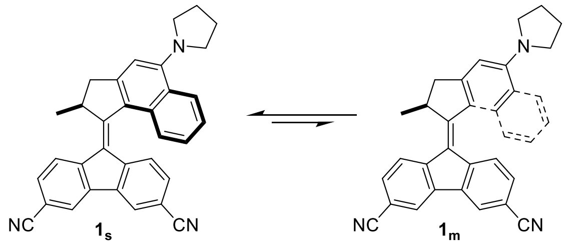

Supplementary Figure 15. Thermal equilibrium between 1_s and 1_m. Thermal exchange reaction between **1_s** and **1_m** studied by EXSY.

NMR Experimental

NMR experiments were performed on a sample ($3.8 \cdot 10^{-3}$ M) dissolved in CD_2Cl_2 on a 500 MHz (^1H NMR frequency) Varian Unity Plus spectrometer. At first a full ^1H spectrum was recorded at 5 °C (Supplementary Figure 16):

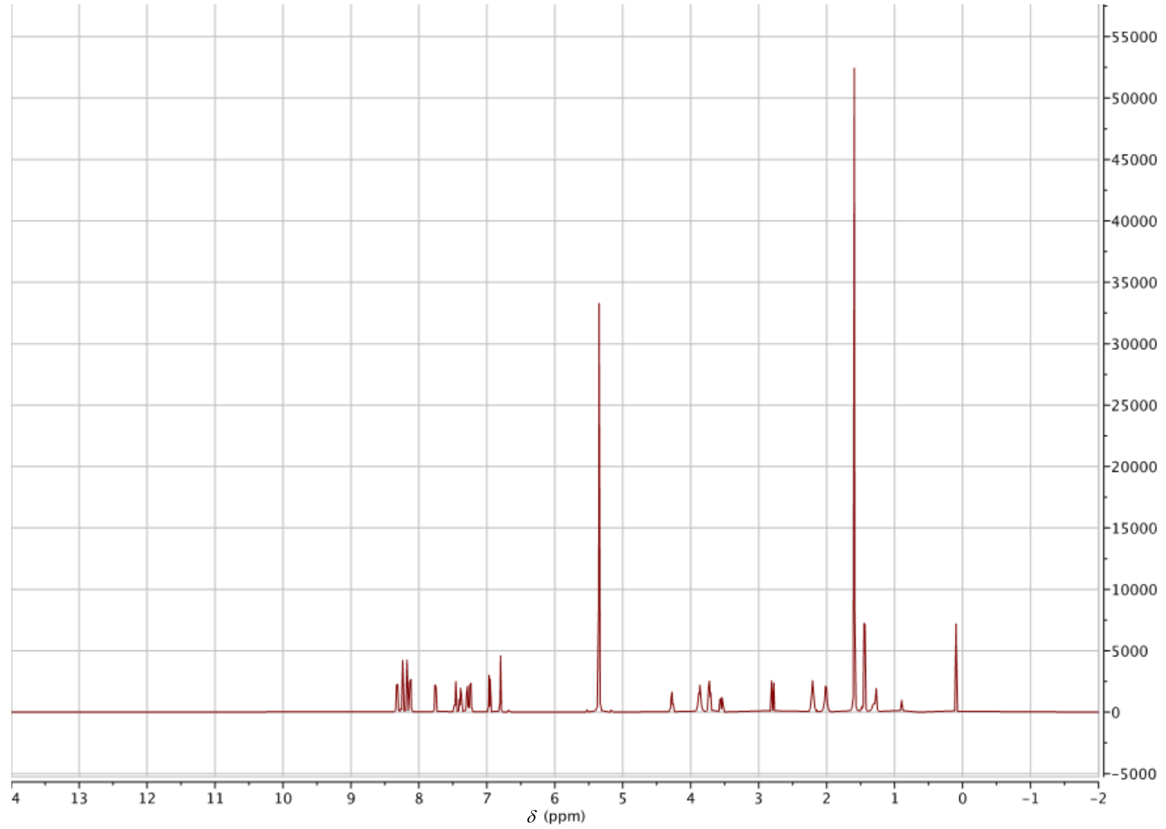

Supplementary Figure 16. ^1H NMR spectrum of 1_s containing a small amount of thermally formed 1_m. Conditions: 500 MHz, $3.8 \cdot 10^{-3}$ M, CD_2Cl_2, 5 °C.

A 2D NOESY recorded at 25 °C revealed several cross-peaks due to exchange (red) and dipolar coupling (blue) (Supplementary Figures 17 and 18):

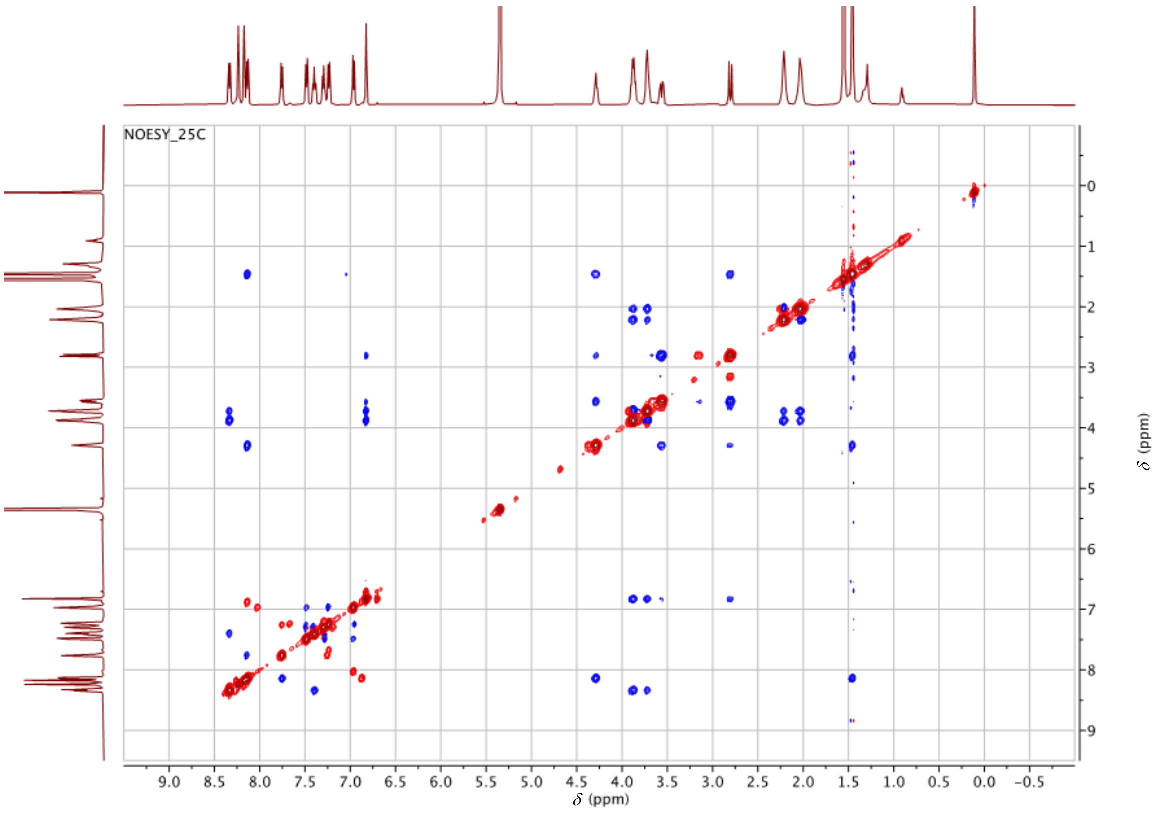

Supplementary Figure 17. 2D NOESY NMR spectrum of a mixture of 1_s and thermally formed 1_m. Conditions: 500 MHz, 500 ms mixing time, $3.8 \cdot 10^{-3}$ M, CD_2Cl_2, 25 °C.

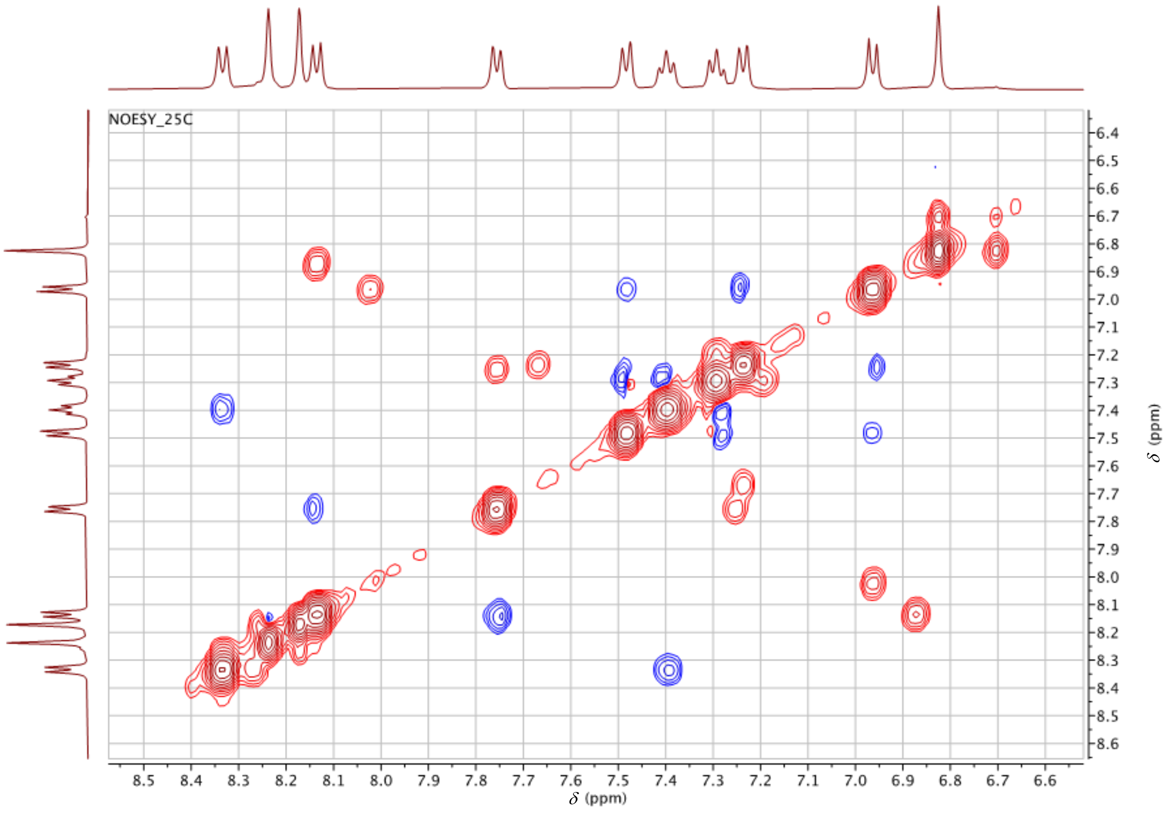

Supplementary Figure 18. Zoom into the aromatic region of Supplementary Figure 17. Conditions: 500 MHz, 500 ms mixing time, $3.8\cdot10^{-3}$ M, CD_2Cl_2, 25 °C.

In order the monitor the chemical exchange between $\mathbf{1_s}$ and $\mathbf{1_m}$ as a function of temperature 1D selective NOESY experiments[8] were recorded with mixing times of 0.1, 0.2, 0.4, 0.8, 1.6, and 3.2 s at 5, 10, 15, 20, and 25 °C. A spectral window of 8000 Hz was employed and 512 scans were acquired for each experiment. The aromatic region of the ^1H spectrum is shown in Supplementary Figure 19 with the doublet at 6.962 ppm being selectively excited indicated with an arrow and the integrated doublet at 8.019 ppm encircled.

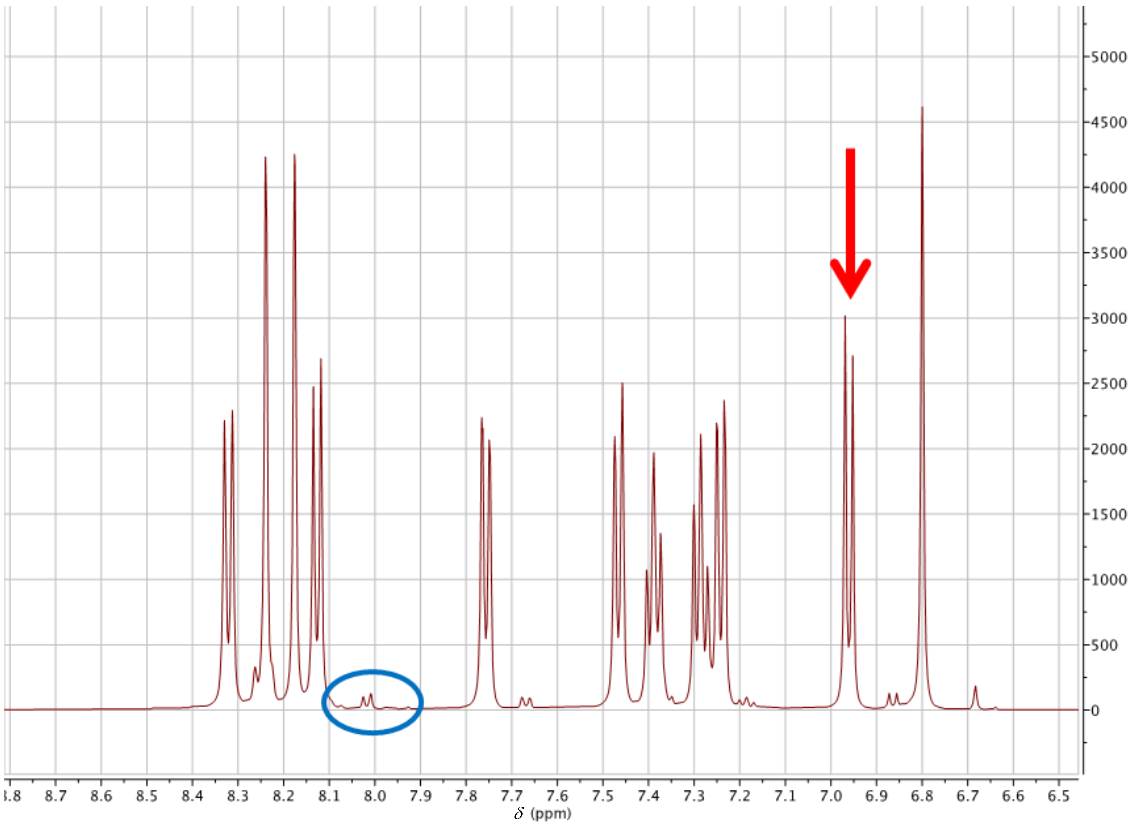

Supplementary Figure 19. Aromatic region of the ^{1}H NMR spectrum of a mixture of $\mathbf{1_s}$ and thermally formed $\mathbf{1_m}$. The signal used for selective excitation to acquire 1D NOESY NMR spectra is indicated by the red arrow. The signal used for integration of the cross-peaks is marked by the blue circle. Conditions: 500 MHz, $3.8{\cdot}10^{-3}$ M, CD_2Cl_2, 25 °C.

Furthermore, equilibrium constants (i.e. population ratios) were determined from 1D ^{1}H spectra and T_1 relaxation rates from inversion recovery experiments. These data were not included as constraints in the modelling procedure (i.e. T_1 values of $\mathbf{1_s}$ and $\mathbf{1_m}$ were assumed to be identical).

Data Modelling

Proposed model:

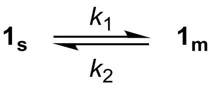

Analysis with $r_1 = r_2 = r$ and cross-peak/diagonal-peak (CP/DP).

$$\frac{\partial \mathbf{1_s}[t]}{\partial t} = (-r - k_1)\mathbf{1_s}[t] + k_2\mathbf{1_m}[t] \qquad \text{Supplementary Equation 1}$$

$$\frac{\partial \mathbf{1_m}[t]}{\partial t} = k_1\mathbf{1_s}[t] + (-r - k_2)\mathbf{1_m}[t] \qquad \text{Supplementary Equation 2}$$

$$\mathbf{1_s}[0] = \frac{k_2}{k_1+k_2} \wedge \mathbf{1_m}[0] = 0$$ Supplementary Equation 3

$$\mathbf{1_s}[t] = \frac{e^{-rt}k_2(e^{-((k_1+k_2)t)}k_1+k_2)}{(k_1+k_2)^2}$$ Supplementary Equation 4

$$\mathbf{1_m}[t] = \frac{e^{-((k_1+k_2+r)t)}(e^{((k_1+k_2)t)}-1)k_1k_2}{(k_1+k_2)^2}$$ Supplementary Equation 5

$$\frac{cross\ peak}{diagonal\ peak} = \frac{\mathbf{1_m}[t]}{\mathbf{1_s}[t]} = \frac{1-e^{-(k_1+k_2)t}}{k_2/k_1+e^{-(k_1+k_2)t}}$$ Supplementary Equation 6

$$P_{\mathbf{1_s}} = \frac{k_2}{k_1+k_2} \text{ and } P_{\mathbf{1_m}} = \frac{k_1}{k_1+k_2}$$ Supplementary Equation 7

$$f[t,T] = \frac{1-e^{-(k_1[T]+k_2[T])t}}{k_2[T]/k_1[T]+e^{-(k_1[T]+k_2[T])t}}$$ Supplementary Equation 8

In which

$$k_1[T] = k_1[T_0]e^{-\frac{\Delta E_1}{R}(\frac{1}{T}-\frac{1}{T_0})}$$ Supplementary Equation 9

$$k_2[T] = k_2[T_0]e^{-\frac{\Delta E_2}{R}(\frac{1}{T}-\frac{1}{T_0})}$$ Supplementary Equation 10

in case of Arrhenius and

$$k_1[T] = \frac{k_B}{h}Te^{-\frac{\Delta H_1^\ddagger}{RT}+\frac{\Delta S_1^\ddagger}{R}}$$ Supplementary Equation 11

$$k_2[T] = \frac{k_B}{h}Te^{-\frac{\Delta H_2^\ddagger}{RT}+\frac{\Delta S_2^\ddagger}{R}}$$ Supplementary Equation 12

In case of Eyring analysis.

Global four parameter fit (k_1[288], k_2[288], ΔE_1, ΔE_2) of CP/DP as function of mixing time (0.1, 0.2, 0.4, 0.8, 1.6, 3.2 s) and temperature (5, 10, 15, 20, 25 °C) using the Arrhenius equation (error estimates derived from a Monte Carlo simulation). Supplementary Table 2 gives a summary of the calculated rate constants (k_1 and k_2) as well as population ratios ($P_{\mathbf{1s}}$) at the temperatures used.

Supplementary Table 2. Summary of the calculated rate constants (k_1 and k_2) and population ratios ($P_{\mathbf{1s}}$) at the different temperatures used according to a global four parameter fit using the Arrhenius equation.

T (°C)	k_1 (s^{-1})	k_2 (s^{-1})	$P_{\mathbf{1s}}$
5	0.041±0.003	1.28±0.07	0.9694±0.0006
10	0.066±0.003	1.92±0.07	0.9666±0.0005
15	0.107±0.003	2.83±0.06	0.9636±0.0004
20	0.169±0.005	4.11±0.08	0.9604±0.0005
25	0.27±0.01	5.9±0.2	0.9572±0.0007

ΔE_1 = 65 ± 3 kJ·mol^{-1}; ΔE_2 = 53 ± 3 kJ·mol^{-1}

Global four parameter fit (ΔH_1, ΔS_1, ΔH_2, ΔS_2) of CP/DP as function of mixing time (0.1, 0.2, 0.4, 0.8, 1.6, 3.2 s) and temperature (5, 10, 15, 20, 25 °C) using the Eyring equation (error estimates derived from a Monte Carlo simulation). Supplementary Table 3 gives a summary of the calculated rate constants (k_1 and k_2) as well as population ratios (P_{1s}) at the temperatures used.

Supplementary Table 3. Summary of the calculated rate constants (k_1 and k_2) and population ratios (P_{1s}) at the different temperatures used according to a global four parameter fit using the Eyring equation.

T (°C)	k_1 (s^{-1})	k_2 (s^{-1})	P_{1s}
5	0.040±0.003	1.28±0.07	0.9694±0.0006
10	0.066±0.003	1.91±0.07	0.9666±0.0005
15	0.107±0.003	2.82±0.06	0.9636±0.0004
20	0.170±0.005	4.11±0.08	0.9604±0.0004
25	0.27±0.01	5.9±0.2	0.9571±0.0007

$\Delta H^{\ddagger}_1 = 62 \pm 3$ kJ·mol^{-1}; $\Delta S^{\ddagger}_1 = -46 \pm 10$ J·K^{-1}·mol^{-1}

$\Delta H^{\ddagger}_2 = 50 \pm 2$ kJ·mol^{-1}; $\Delta S^{\ddagger}_2 = -61 \pm 8$ J·K^{-1}·mol^{-1}

Supplementary Figure 20 shows the global fit of the equations including the Arrhenius temperature dependence and the linearized Eyring plots generated from the data in Supplementary Table 3.

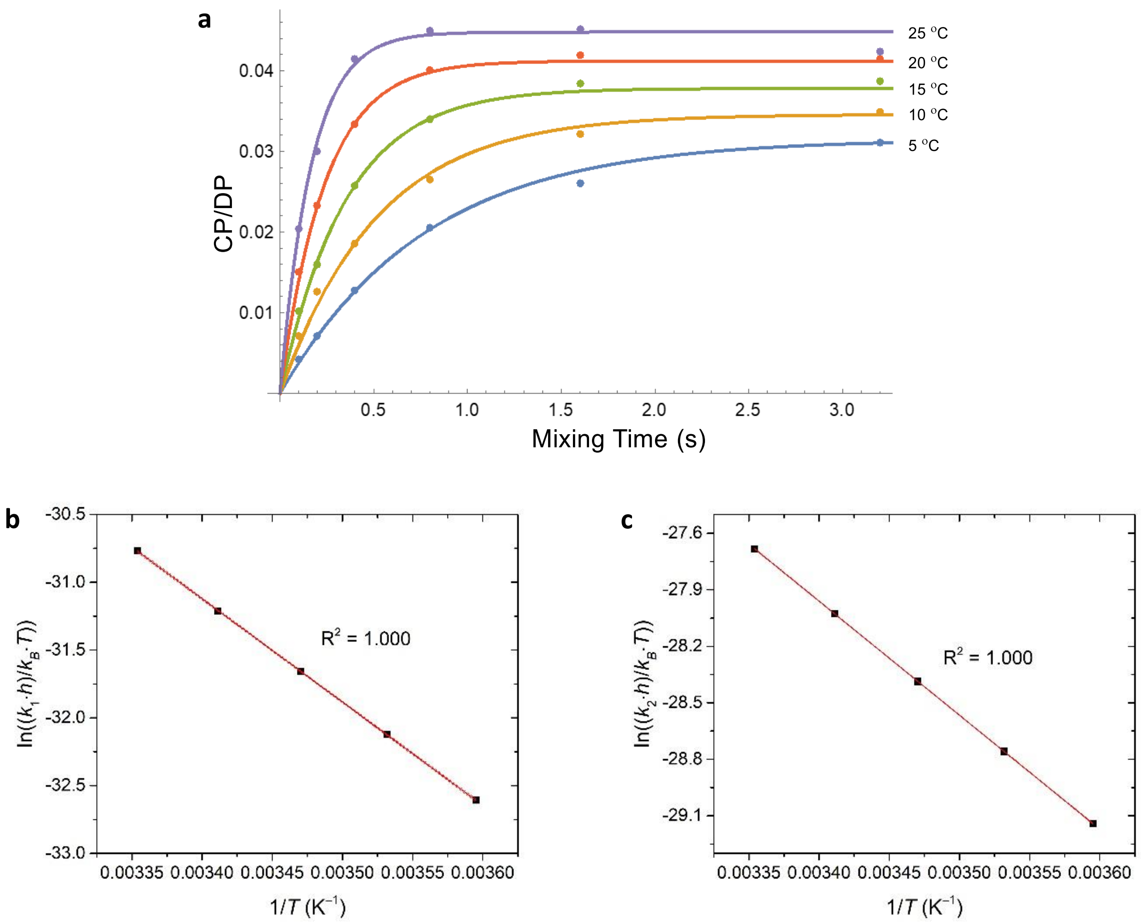

Supplementary Figure 20. Activation barriers of thermal *E*/*Z* isomerization of 1. a Plot showing the ratios of the integrated area under the cross-peaks vs. diagonal peaks (CP/DP) at different mixing times and temperatures and the global fit of the equations including the Arrhenius temperature dependence. **b** Linearized Eyring plot for the formation of $\mathbf{1}_\mathbf{m}$ from $\mathbf{1}_\mathbf{s}$. Dotted lines represent 95% confidence intervals. **c** Linearized Eyring plot for the formation of $\mathbf{1}_\mathbf{s}$ from $\mathbf{1}_\mathbf{m}$. Dotted lines represent 95% confidence intervals.

5. UV-vis Absorption Studies

5.1. Steady-State UV-vis Absorption Spectra of $\mathbf{1}_\mathbf{s}$, at PSS and During Isomerization

Solutions of $\mathbf{1}_\mathbf{s}$ ($1.0\cdot10^{-5}$ M) in cyclohexane, toluene, MeCN and DCM were prepared, of which 2.5 mL were placed in a 1.0 cm quartz cuvette and UV-vis absorption spectra were recorded at 20 °C. The samples were then irradiated with a 528 nm or 595 nm LED until no further change was observed (5 min for the samples in MeCN and DCM). Spectra were recorded at regular intervals over the course of the irradiation. Supplementary Figure 21 shows the spectra before irradiation ($\mathbf{1}_\mathbf{s}$) and at PSS. Subsequently, the LED was switched off and in the case of the samples in cyclohexane and toluene spectra were recorded over the course of the thermal back reaction.

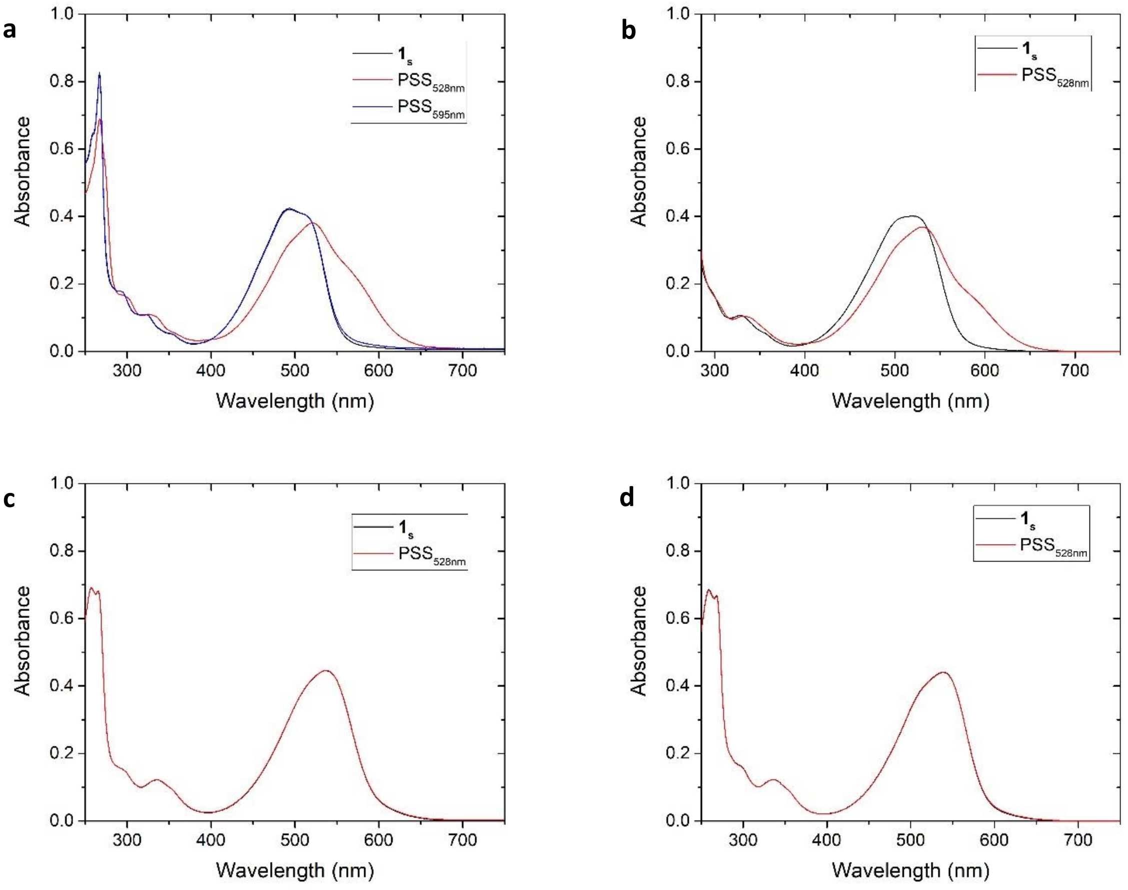

Supplementary Figure 21. Photochemical *E*/*Z* isomerization of 1 in different solvents. Steady-state UV-vis absorption spectra of a solution of $\mathbf{1_s}$ (c=1.0·10^{-5} м, 20 °C) and after irradiation to PSS with an LED with a nominal wavelength of 528 nm or 595 nm in cyclohexane (**a**), toluene (**b**), MeCN (**c**) and DCM (**d**).

Supplementary Figure 22 shows stacks of spectra recorded during the irradiation as well as thermal back-reaction of **1** in cyclohexane and toluene.

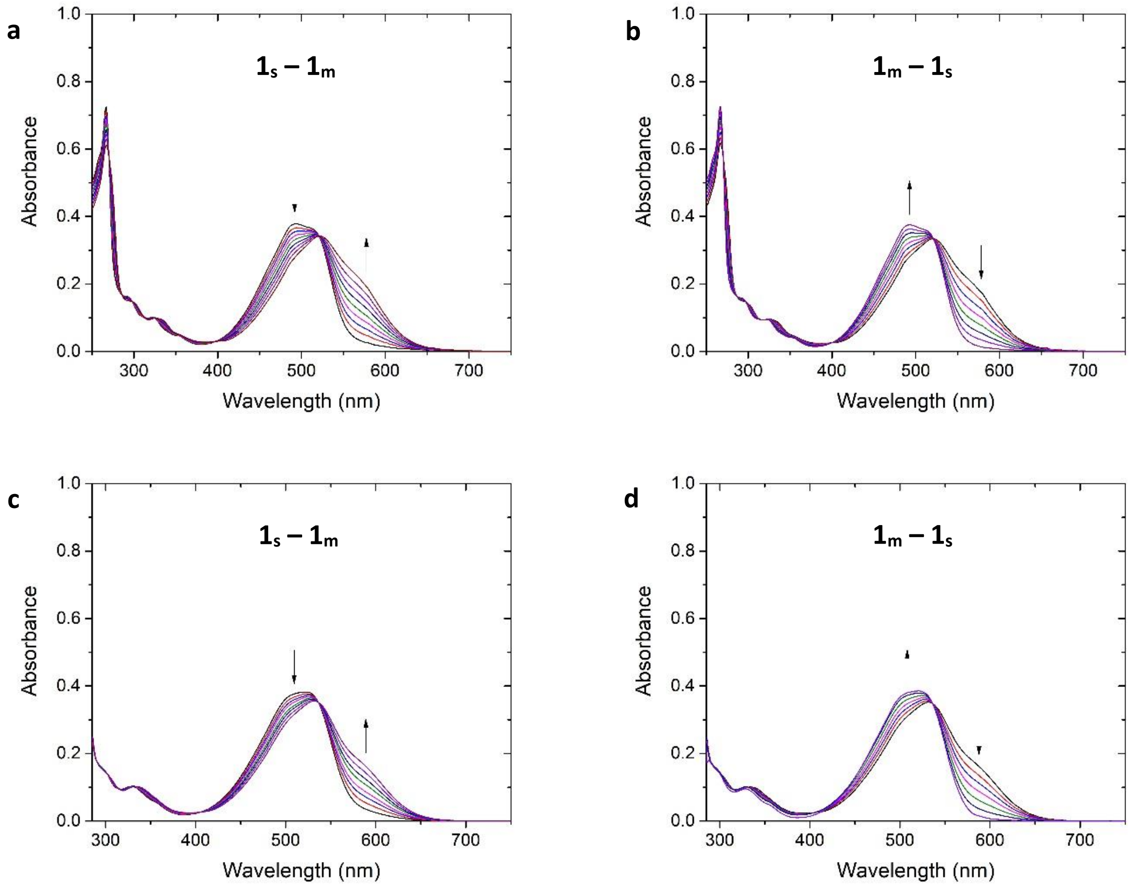

Supplementary Figure 22. Isomerization behaviour of 1 in apolar solvents. Stacks of steady-state UV-vis absorption spectra recorded during irradiation of a solution of $\mathbf{1_s}$ (c=1.0·10^{-5} M, 20 °C) with a 528 nm LED (**a**, **c**) and during subsequent THI (**b**, **d**) in cyclohexane (**a**, **b**) and toluene (**c**, **d**).

5.2. Steady-State UV-vis Absorption Spectra of $\mathbf{1_s}$ in Different Solvents

A 2.0·10^{-4} M solution of $\mathbf{1_s}$ in DCM was prepared. Aliquots of 125 µL were placed in vials, the solvent was removed *in vacuo* and the remaining solid was redissolved in 2.5 mL (1.0·10^{-5} M) of the according solvent. The solutions were placed in a 1.0 cm quartz cuvette and UV-vis absorption spectra were recorded at 20 °C. Normalized spectra of $\mathbf{1_s}$ in all solvents tested are shown in Supplementary Figure 23.

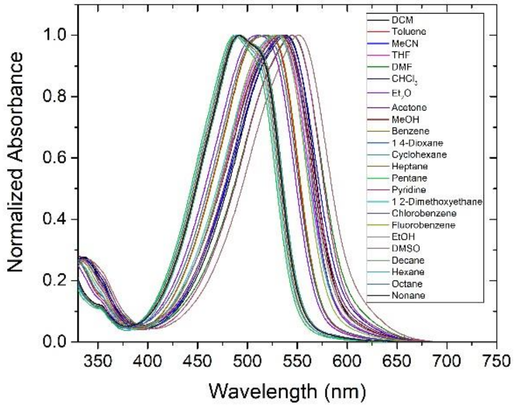

Supplementary Figure 23. Solvent-dependent UV-vis absorption properties of $\mathbf{1_s}$. Normalized steady-state UV-vis absorption spectra of $\mathbf{1_s}$ in different solvents.

5.3. Eyring Study on $\mathbf{1_m}$

A $1.0 \cdot 10^{-5}$ M solution of $\mathbf{1_s}$ in cyclohexane was prepared. Samples of 2.5 mL (1.0 cm quartz cuvette) at five different temperatures were at first irradiated to PSS using a 528 nm LED and the subsequent thermal helix inversion back to the stable isomer was followed on a UV-vis spectrophotometer. The absorbance at 565 nm was plotted over time (Supplementary Figure 24a). Rate constants, *k*, were determined by fitting a 1st order rate law. Thermodynamic parameters for the formation of the transition state were obtained by fitting the linearized form of the Eyring equation (Supplementary Figure 24b, Supplementary Table 4).

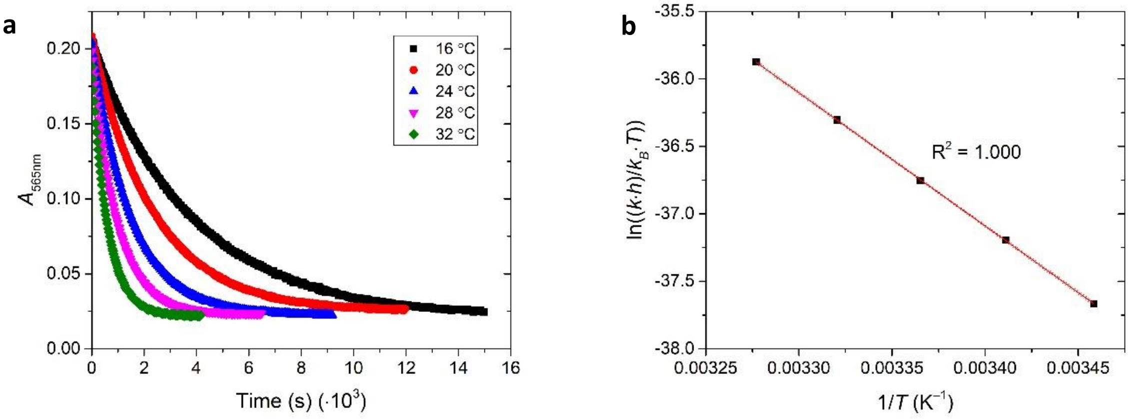

Supplementary Figure 24. Eyring study of the thermal isomerization of $\mathbf{1_m}$. a Change in absorbance at 565 nm over time of five samples of $\mathbf{1_s}$ and $\mathbf{1_m}$ at different temperatures after irradiation to PSS (c=$1.0 \cdot 10^{-5}$ M, cyclohexane, 528 nm LED). **b** Linearized Eyring plot. Dotted lines represent 95% confidence intervals.

Supplementary Table 4. Summary of thermodynamic parameters of THI of metastable isomer $\mathbf{1_m}$ in cyclohexane.

$\Delta G^‡$(20 °C) (kJ·mol^{-1})	$\Delta H^‡$ (kJ·mol^{-1})	$\Delta S^‡$ (J·mol^{-1}·K^{-1})	$t_{1/2}$ (min)
90.7±0.1	82.0	–29.5	27.1

5.4. Fatigue Study on 1

A 1.0·10^{-5} M solution of $\mathbf{1_s}$ in cyclohexane was prepared. A sample (2.5 mL) was placed in a 1.0 cm quartz cuvette. A UV-vis absorption spectrum was recorded at 20 °C before the sample was irradiated to PSS using a 528 nm LED. Another spectrum was recorded, the light source was removed, and the sample was allowed to undergo complete thermal relaxation before another spectrum was recorded. This process was repeated ten times in total. The absorbance at 492 nm at the beginning and after each step is plotted in Supplementary Figure 25.

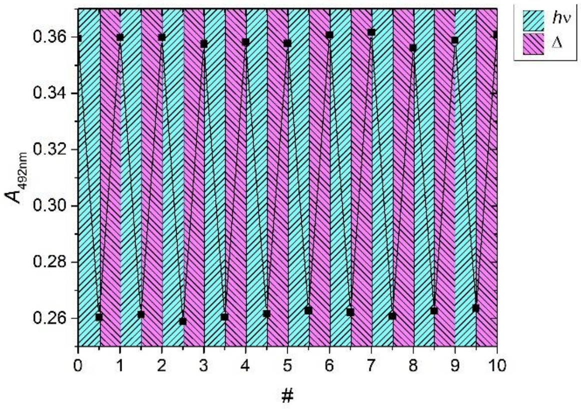

Supplementary Figure 25. Fatigue study on 1. 10 cycles of photochemical and subsequent thermal isomerizations of **1** in cyclohexane (c=1.0·10^{-5} M, 20 °C, 528 nm LED).

5.5. Protonation of $\mathbf{1_s}$

A 1.0·10^{-5} M solution of $\mathbf{1_s}$ in MeCN was prepared. A sample (2.5 mL) was placed in a quartz cuvette (1.0 cm) and aliquots of a 1.0 v/v% solution of HBF_4·OEt_2 in MeCN were added inside a glove box recording a UV-vis absorption spectrum after each addition. This was continued until no further change was observed. Supplementary Figure 26a shows a stack of spectra recorded over the course of protonation of $\mathbf{1_s}$.

In a second experiment 2.5 mL of the same stock solution of $\mathbf{1_s}$ was placed in a quartz cuvette (1.0 cm) and a UV-vis absorption spectrum was recorded. Subsequently, 5.0 µL of a 1.0 v/v% solution of HBF_4·OEt_2 was added to obtain $\mathbf{(1–H)_s^+}$ and a second spectrum was recorded. Then 1.0 eq. of NEt_3 relative to the acid was added and a third spectrum was collected. Two more protonation-deprotonation cycles were performed by adding acid and base in an alternating fashion. The obtained spectra are shown in Supplementary Figure 26b.

Note: These experiments were not performed in strictly anhydrous solvent.

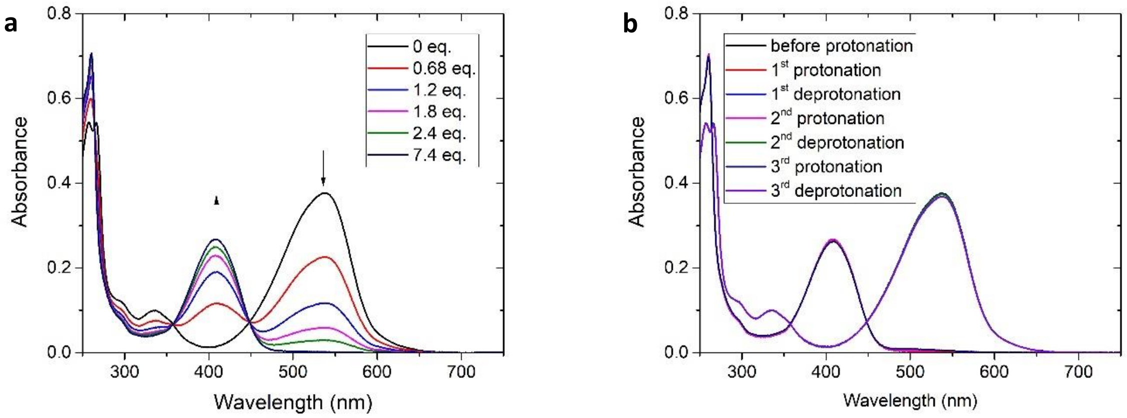

Supplementary Figure 26. Protonation of $\mathbf{1_s}$. a Stack of spectra recorded during protonation of $\mathbf{1_s}$ (c=1.0·10^{-5} M, MeCN, 20 °C) with HBF_4·OEt_2. **b** Three consecutive protonation-deprotonation cycles using HBF_4·OEt_2 and NEt_3, respectively, starting from a sample of $\mathbf{1_s}$ (c=1.0·10^{-5} M, MeCN, 20 °C)

5.6. Steady-State UV-vis Absorption Spectra of $\mathbf{(1–H)_s^+}$, at PSS and During Isomerization

Samples (2.5 mL) of 1.0·10^{-5} M solutions of $\mathbf{1_s}$ in anhydrous MeCN and DCM were placed in quartz cuvettes (1.0 cm). UV-vis absorption spectra were recorded at 20 °C before 20 µL (MeCN) and 5 µL (DCM) of a 1.0 v/v% solution of HBF_4·OEt_2 in the same solvent were added inside a glove box. Another set of UV-vis spectra was recorded at 20 °C before each sample was irradiated to PSS using a 395 nm LED. Spectra were recorded over the course of the irradiation. Subsequently, also the thermal recovery of $\mathbf{(1–H)_s^+}$ was followed over time. Supplementary Figure 27 shows the spectra of $\mathbf{1_s}$, $\mathbf{(1–H)_s^+}$ and the PSS mixture of $\mathbf{(1–H)_s^+}$ and $\mathbf{(1–H)_m^+}$ in MeCN and DCM.

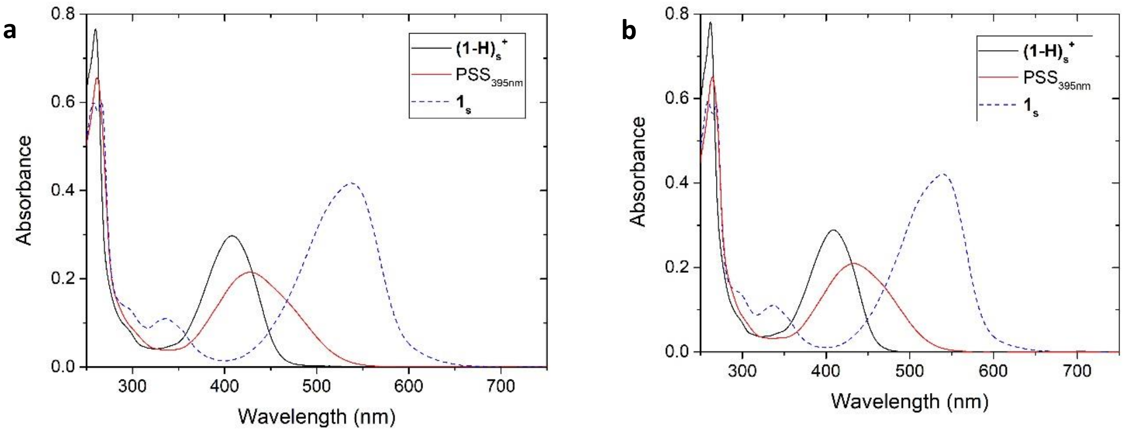

Supplementary Figure 27. Photochemical *E/Z* isomerization of $\mathbf{(1–H)^+}$. Steady-state UV-vis absorption spectra of a solution of $\mathbf{(1–H)_s^+}$ (c=1.0·10^{-5} M, 20 °C) and after irradiation to PSS with an LED of 395 nm MeCN (**a**) and DCM (**b**). Dashed spectra are those of $\mathbf{1_s}$ recorded before adding HBF_4·OEt_2.

Supplementary Figure 28 shows the stacks of spectra recorded during the irradiation as well as thermal back-reaction of **(1–H)**$^+$ in MeCN and DCM.

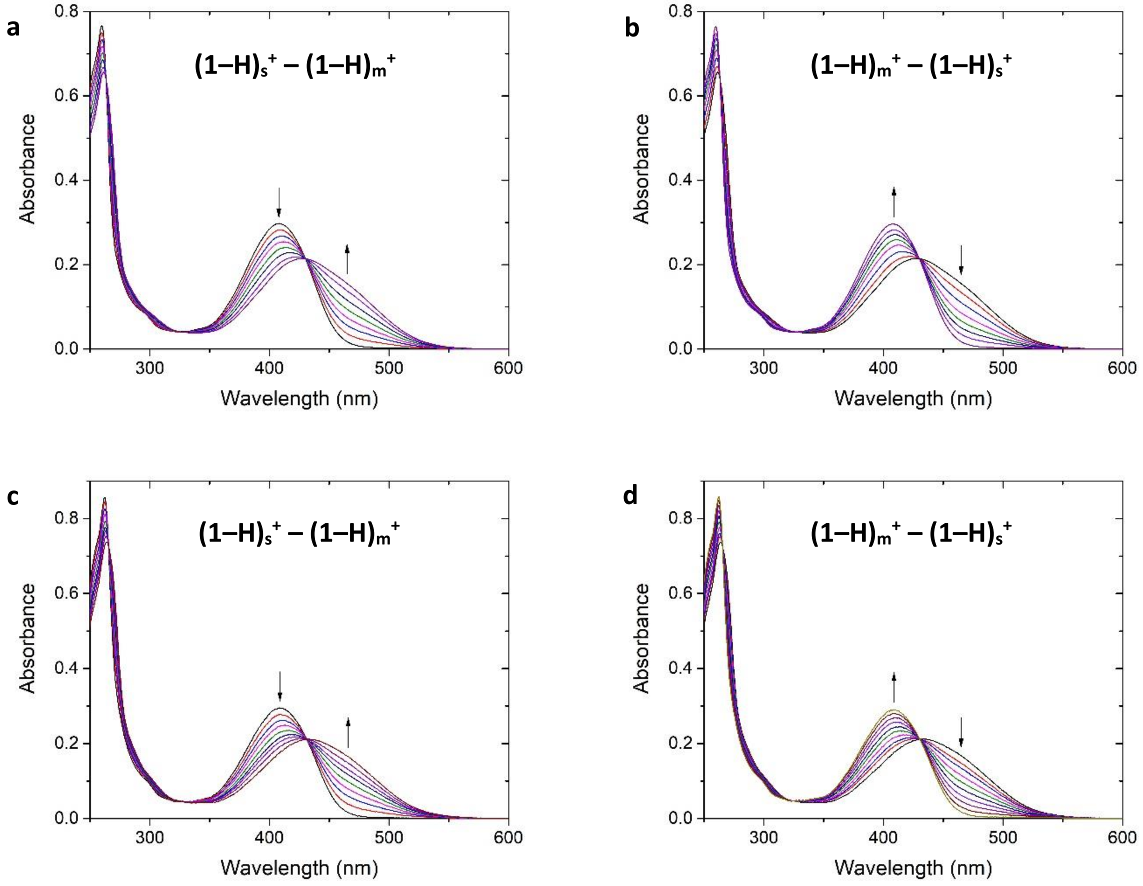

Supplementary Figure 28. Isomerization behaviour of (1–H)$^+$. Stacks of steady-state UV-vis absorption spectra recorded during irradiation of a solution of **(1–H)$_s^+$** (c=1.0·10^{-5} M, 20 °C) with a 395 nm LED (**a**, **c**) and during subsequent THI (**b**, **d**) in MeCN (**a**, **b**) and DCM (**c**, **d**).

5.7. UV-vis Spectra of (1–H)$_s^+$ in Different Solvents

A 2.0·10^{-4} M solution of **1$_s$** in anhydrous DCM was prepared. Aliquots of 125 µL were placed in vials, the solvent was removed *in vacuo* and the remaining solid was redissolved in 2.5 mL of the according anhydrous solvent. The solutions were placed in a 1.0 cm quartz cuvette and $HBF_4 \cdot OEt_2$ was added inside a glove box until no further change in absorbance was observed. The normalized UV-vis absorption spectra of **(1–H)$_s^+$** in all studied solvents are shown in Supplementary Figure 29.

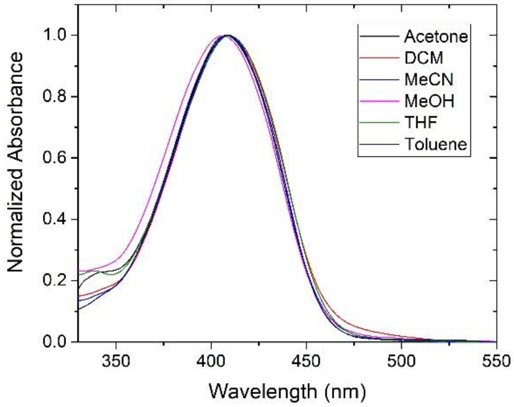

Supplementary Figure 29. UV-vis absorption properties of $(\mathbf{1{-}H})_s^+$ in different solvents. Normalized steady-state UV-vis absorption spectra of $(\mathbf{1{-}H})_s^+$ in different solvents.

5.8. Eyring Study on $(\mathbf{1{-}H})_m^+$

A $1.0{\cdot}10^{-5}$ M solution of $\mathbf{1}_s$ in anhydrous MeCN was prepared in a glove box. Samples were prepared by adding 2 µL of $HBF_4{\cdot}OEt_2$ to 2.0 mL of this stock solution which had been placed in a 1.0 cm quartz cuvette. At this point the sample was removed from the glove box. Samples at five different temperatures were at first irradiated to PSS using a 395 nm LED and the subsequent thermal isomerization back to the stable isomer was followed on a UV-vis spectrophotometer. This experiment was repeated in DCM using the same concentration of $\mathbf{1}_s$ and 20 µL of $HBF_4{\cdot}OEt_2$ for each sample. The absorbances at 466 nm was plotted over time (Supplementary Figure 30a, c). Rate constants, *k*, were determined by fitting a 1st order rate law. Thermodynamic parameters for the formation of the transition state were obtained by fitting the linearized form of the Eyring equation (Supplementary Figure 30b, d, Supplementary Table 5).

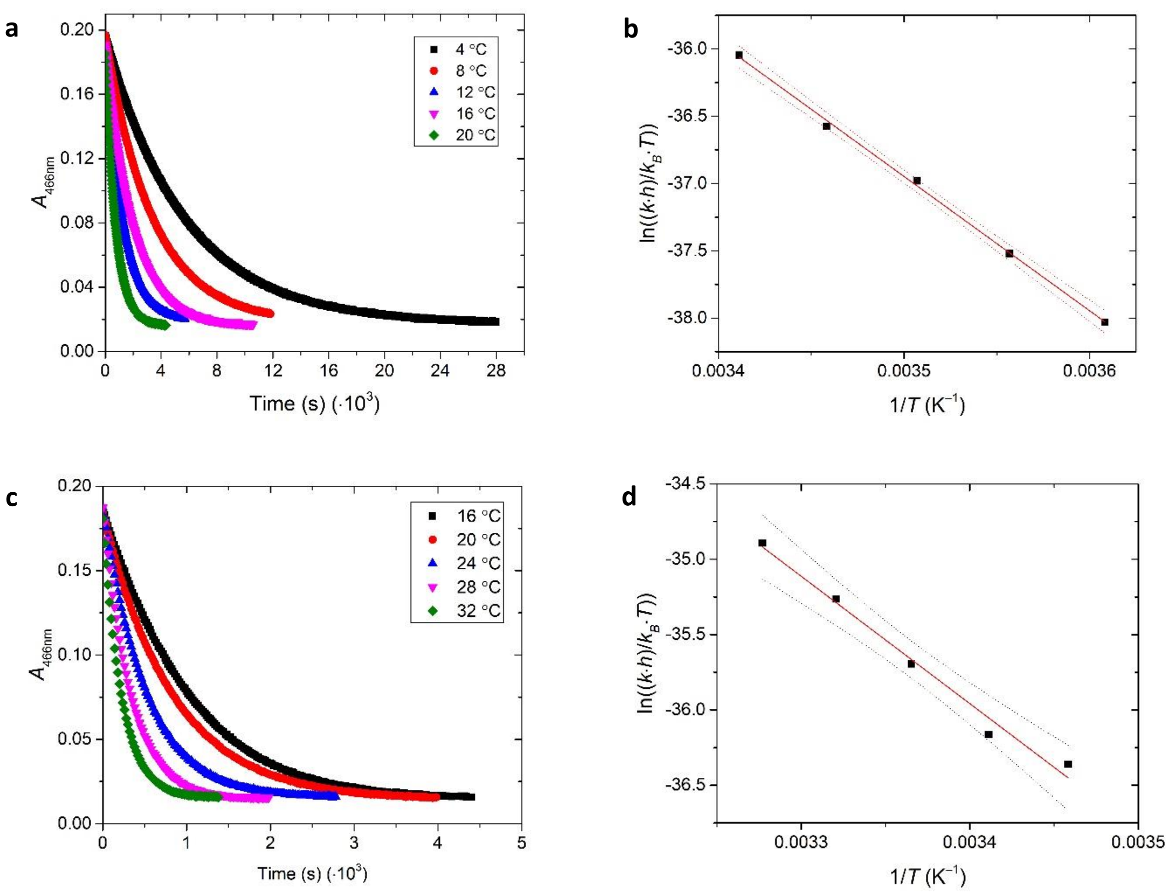

Supplementary Figure 30. Eyring study of the thermal isomerization of (1–H)$_m^+$. a Change in absorbance at 466 nm over time of five samples of **(1–H)$_s^+$** and **(1–H)$_m^+$** in MeCN at different temperatures after irradiation to PSS (c=1.0·10^{-5} M, MeCN, 395 nm LED). **b** Linearized Eyring plot of experiment in MeCN. Dotted lines represent 95% confidence intervals. **c** Change in absorbance at 466 nm over time of five samples of **(1–H)$_s^+$** and **(1–H)$_m^+$** in DCM at different temperatures after irradiation to PSS (c=1.0·10^{-5} M, DCM, 395 nm LED). **d** Linearized Eyring plot of experiment in DCM. Dotted lines represent 95% confidence intervals.

Supplementary Table 5. Summary of thermodynamic parameters of THI of metastable isomer **(1–H)$_s^+$** in MeCN and DCM.

	**Δ$G^‡$(20 °C) (kJ·mol^{-1})**	**Δ$H^‡$ (kJ·mol^{-1})**	**Δ$S^‡$ (J·mol^{-1}·K^{-1})**	**$t_{1/2}$ (min)**
MeCN	87.9±0.2	83.0	−16.8	8.67
DCM	87.9±0.4	70.3	−60.1	8.59

5.9. Fatigue Study on (1–H)$^+$

A 1.0·10^{-5} M solution of **1$_s$** in anhydrous MeCN was prepared. A sample (2.5 mL) was placed in a 1.0 cm quartz cuvette and 20 µL of a 1.0 v/v% solution of $HBF_4·OEt_2$ in MeCN were added inside a glove box. The sample was taken out of the glove box and a UV-vis absorption spectrum was recorded at 20 °C before the sample was irradiated to PSS using a 395 nm LED. Another spectrum was recorded, the light source was removed and the sample was allowed to undergo complete thermal relaxation before another

spectrum was recorded. This process was repeated ten times in total. The absorbance at 408 nm at the beginning and after each step is plotted in Supplementary Figure 31.

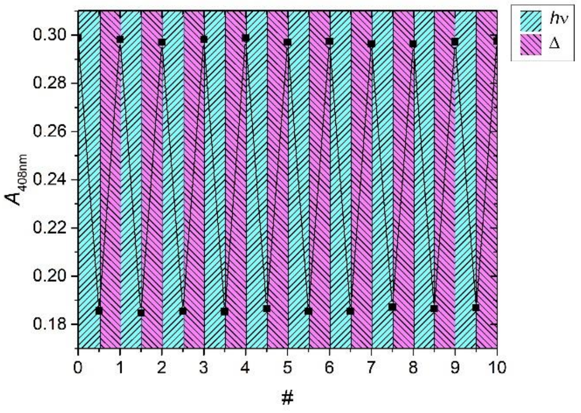

Supplementary Figure 31. Fatigue study on (1–H)⁺. 10 cycles of photochemical and subsequent thermal isomerizations of **(1–H)⁺** in MeCN (c=1.0·10^{-5} M, 20 °C, 395 nm LED).

6. Kamlet-Taft Analysis

The influence of solvent effects on the UV-vis profile of a molecule involves a great number of parameters that can be rationalized using different concepts, such as dipole-moment, polarizability, hydrogen donating effects and so on. To address the huge number of variables involved in such a complex interaction, qualitative scales such as the solvatochromic ET(30) were introduced[9]. A quantitative alternative is represented by the Linear Solvation Energy Relationship multivariate regression, that correlates λ with the Kamlet-Taft parameters α, β and π^* in the following equation:

$$\lambda_{max} = \lambda_{max,0} + a\alpha + b\beta + c(\pi^* - d\delta) \qquad \text{Supplementary Equation 13}$$

Where α is the acidity term, or the ability of the solvent to donate a proton, forming a hydrogen bond. β is the basicity term, or the solvent prowess to accept a proton in a hydrogen bond. π^* is the polarizability term, or a measure of the solvent stabilization of a dipole. δ is a correction in the polarizability term equal to 1.0 for aromatic solvents, 0.5 for the poly-chlorinated ones, 0.0 for the non-chlorinated ones[10–12].

The regressed values a, b, c and d indicate the sign (either positive or negative) and the magnitude with which a specific polarity parameter is affecting λ. The multivariate regression was performed using the lowest-energy transition maxima of both, half-chair andenvelope, conformations of $\mathbf{1_s}$ using OriginPro 2016, without furnishing any additional constraint to the analysis. To extract these absorption maxima it was assumed that the members of both sets (half-chairs and envelopes) have identical lowest-energy transition profiles. Therefore, the experimental spectrum could be fitted in the region corresponding to this transition using two Gaussians.

The most relevant parameter that influences the solvatochromism of both conformer types of $\mathbf{1_s}$ is the one related to the polarizability, π^* (see Supplementary Figure 32).

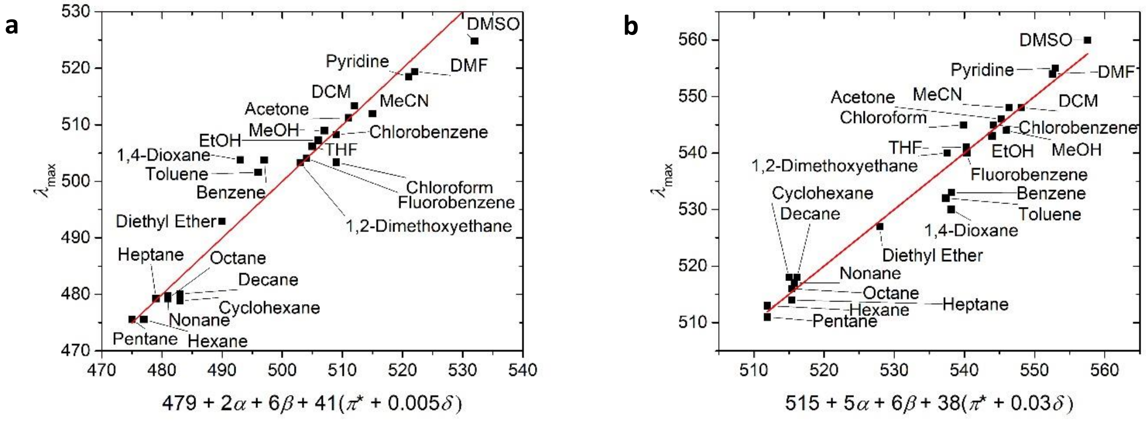

Supplementary Figure 32. Multivariate analysis of the solvatochromism of 1_S. Kamlet-Taft plot of the solvatochromism of the half-chair (**a**) and envelope (**b**) conformations of 1_S.

7. Computational Analysis

All the calculations for the thermal isomerization of **1** and for its protonated analogue **$(1–H)^+$** were carried out using the Gaussian 16 Rev. B.01 software[13]. As an approximation, the BF_4^- was excluded from the calculations of **$(1–H)^+$**. The mechanisms were analyzed at the CAM-B3LYP/6-31G(d,p) level and the results were compared to the ones obtained at the ωB97X-D/def2-SVP level. The SMD implicit solvent model was used to model acetonitrile (ε = 37.5) solvation. The stable and metastable states were connected via the thermal helix inversion transition state (TS_{THI}) and the *E*/*Z* transition state (TS_{EZ}). A broken-symmetry approach was included to take into account the possible diradical nature of the *E*/*Z* isomerization transition state. All the optimizations were confirmed to be stationary points by the number of imaginary frequencies found (0 for the minima, 1 for the transition states). The monodeterminantal nature of the transition state (due to the marked charge transfer character) was confirmed by means of a minimal CASSCF(10,10)/6-31G* single point calculation using OpenMolcas v18.09[14] on the TS_{EZ} optimized in gas phase at the CAM-B3LYP/6-31G(d,p) level. The closed shell conformation represented more than 89% of the ones possible for the transition state.

The conformational mobility of the pyrrolidine ring was probed studying different conformers (see Supplementary Figure 33b). Four envelopes and four half chairs were found. They were dubbed **1**, **1-inv**, **2** and **2-inv** (see Supplementary Figure 33b). The half-chair-1-inv and half-chair-2-inv conformations were obtained only for the TS_{EZ} *in vacuo*, both with CAM-B3LYP and ωB97X-D. In all cases the contribution to the total population of TS_{EZ} was less than 0.2%, hence we decided not to include these conformations in Supplementary Figure 33, although they were used to compute the total energy of the transition step. The energies of all the conformers were Boltzmann averaged to obtain the energies presented in Supplementary Figure 33a. The half-chair conformation is preferred, but the bias between envelope and half-chair becomes more pronounced in more polar solvents. This is because the half-chair favors the donation of the n orbital of the pyrrolidine N into the π* orbitals of the aromatic structure. Quantitative treatment of this donation was obtained through NBO analysis at the CAM-B3LYP/6-31G(d,p) level with the NBO6 software package[15]. Indeed, the donation of the most abundant half-chair in the stable form **1_S** *vs* the most abundant envelope is 223.38 vs 109.12 kJ/mol (Supplementary Figure 33b).

The marked difference between the thermal *E*/*Z* isomerization barriers (Supplementary Figure 33a) computed *in vacuo* vs in acetonitrile explains why, with more polar solvents, no more photochemical isomerization can be observed experimentally, even at low temperature. The motor loses its unidirectionality and reverts from $\mathbf{1_m}$ to $\mathbf{1_s}$ via TS_{EZ} with an extremely low barrier (ca. 55 kJ/mol). In solvents with low polarity **1** is a unidirectional motor that interconverts from $\mathbf{1_m}$ to $\mathbf{1_s}$ via TS_{THI}. The optimizations in acetonitrile for $\mathbf{(1–H)^+}$ confirm that the nitrogen n–π* interaction has a relevant contribution to the different behavior of $\mathbf{(1–H)^+}$ in different solvents. Indeed, the barrier for THI is 92 kJ/mol with SMD-CAM-B3LYP/6-31G(d) and 91 kJ/mol for ωB97X-D/def2-SVP, while that of *E*/*Z* isomerization is 120 and 113 kJ/mol, respectively.

The TD spectra of the different geometries of $\mathbf{1_s}$ obtained *in vacuo* or in acetonitrile at the CAM-B3LYP/6-31G(d,p) level were further used to compute the UV-vis spectra at the (SMD)-TD-PBE0/6-311+G(2d,p) level of theory, considering the first 25 transitions. Also, in this case, the SMD model was used to compute the effect of acetonitrile on the solvatochromism of the selected geometries. The different simulated UV-vis absorption spectra confirm the different degree of conjugation between the pyrrolidinic N and the π system (see Supplementary Figure 34), with the half-chair conformations affording more red-shifted spectra than the envelopes. Therefore, the presence of two overlapping bands in the lowest-energy transition region of the experimental UV-vis absorption spectrum can be explained by the conformational mobility of the pyrrolidine.

The cartesian coordinates (in Ångstrom) and energies (in Hartrees) for all the relevant structures are reported in the following section.

a

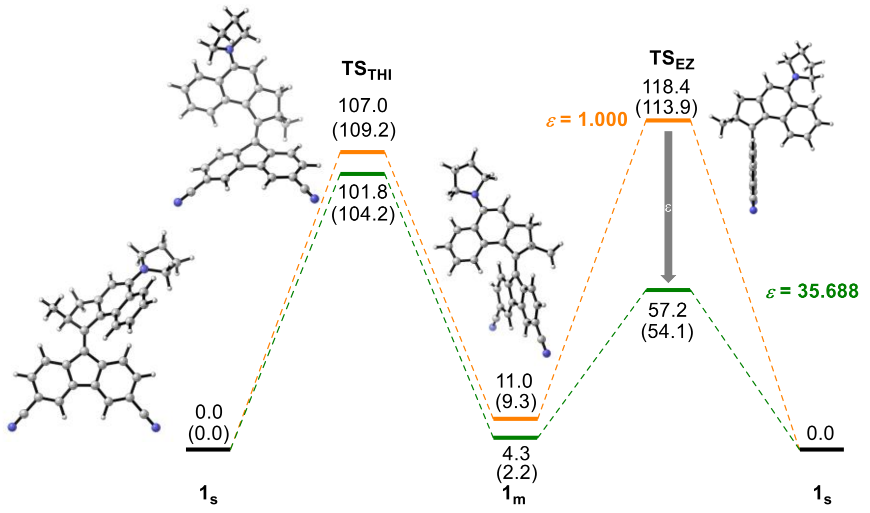

Energy profile at the (SMD)-CAM-B3LYP/6-31G(d) and (SMD)-ωB97X-D/def2-SVP (in brackets) levels. All the energies are expressed in kJ/mol.

b

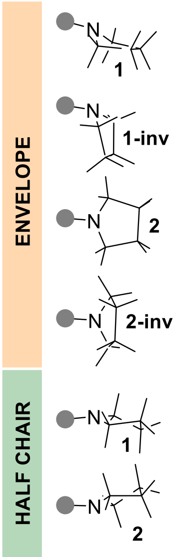

	Conformer	1_s 1.0	1_s 35.7	TS_{THI} 1.0	TS_{THI} 35.7	1_m 1.0	1_m 35.7	TS_{EZ} 1.0	TS_{EZ} 35.7
ENVELOPE	1	6.3<br>(3.7)<br>91.38	1.4<br>(0.6)<br>102.09	6.6<br>(3.8)	0.6<br>(0.2)	22.0<br>(11.4)<br>119.66	3.0<br>(2.6)<br>148.45		
ENVELOPE	1-inv	0.3<br>(0.3)<br>95.60	0.1<br>(0.1)<br>108.87	0.3<br>(0.1)	0.1<br>(0.1)	0.8<br>(0.4)<br>148.99	0.3<br>-<br>231.25		
ENVELOPE	2	30.1<br>(19.6)<br>109.12	10.7<br>(4.2)<br>124.93	28.4<br>(12.6)	8.9<br>(5.2)	3.4<br>(1.1)<br>98.03	0.3<br>(0.1)<br>115.77		
ENVELOPE	2-inv	1.0<br>(1.2)<br>113.51	0.6<br>(0.3)<br>166.73	1.0<br>(0.6)	0.5	0.2<br>(0.0)<br>123.22	0.0<br>(0.0)<br>153.39		
HALF CHAIR	1	5.8<br>(10.2)<br>211.10	5.5<br>(8.5)<br>265.47	6.5<br>(7.8)	4.9<br>(3.4)	68.2<br>(81.9)<br>239.49	93.4<br>(95.7)<br>315.22	46.1<br>(52.5)	37.5<br>(21.8)
HALF CHAIR	2	56.6<br>(65.0)<br>223.38	81.6<br>(86.3)<br>276.77	57.2<br>(75.0)	85.0<br>(91.1)	5.3<br>(5.1)<br>224.68	2.9<br>(1.7)<br>252.00	53.4<br>(47.1)	62.5<br>(78.2)

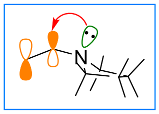

n-π* interaction

NBO6 analysis at the CAM-B3LYP/6-31g(d,p) level
Energies in kJ/mol

For every step in the path, the conformer percentage obtained from the Boltzmann distribution *in vacuo* and in acetonitrile is reported. The values are obtained at the (SMD)-CAM-B3LYP/6-31G(d) and (SMD)-ωB97X-D/def2-SVP (in brackets) levels. The second order orbital interaction $E^{(2)}$ computed at the (SMD)-CAM-B3LYP/6-31G(d) level is reported in blue.

Supplementary Figure 33. Energy barriers of thermal isomerizations of 1_m. a Profile of thermal isomerizations of 1_m. **b** Conformer analysis of the various steps of the thermal isomerization of 1_m, along with the NBO analysis of the n–π* donation of the pyrrolidine ring into the π backbone of the motor.

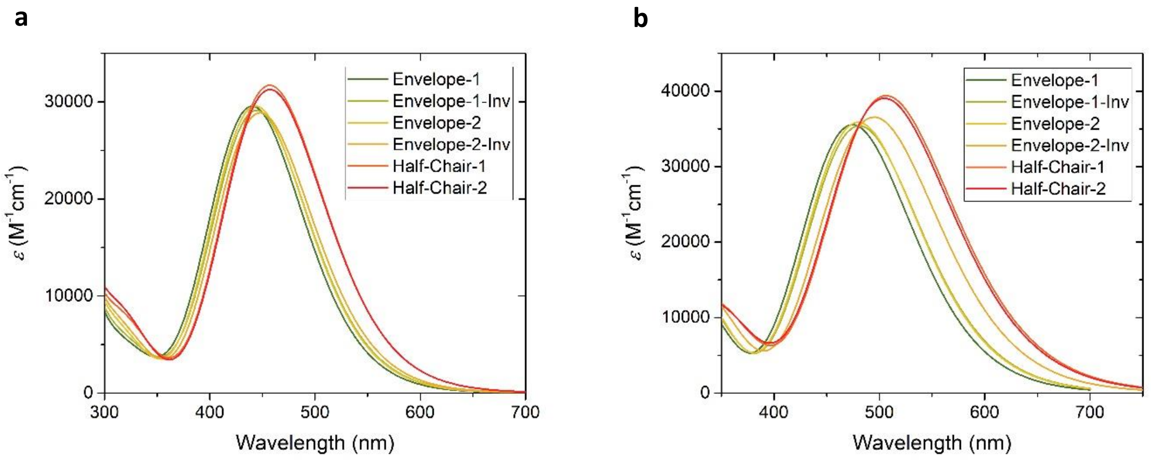

Supplementary Figure 34. Conformation-dependent UV-vis absorption properties of 1_S. Simulated absorption spectra of the different conformers of $\mathbf{1_S}$ in implicit solvent with $\varepsilon = 1.0$ (**a**) and $\varepsilon = 37.5$ (**b**). Data at the PBE0/6-311+G(2d,p)//CAM-B3LYP/6-31G(d,p) level.

TD-DFT simulated UV-vis spectra of all discussed conformers of $\mathbf{1_S}$ in acetonitrile and *in vacuo* are reported in Supplementary Figures 35–46.

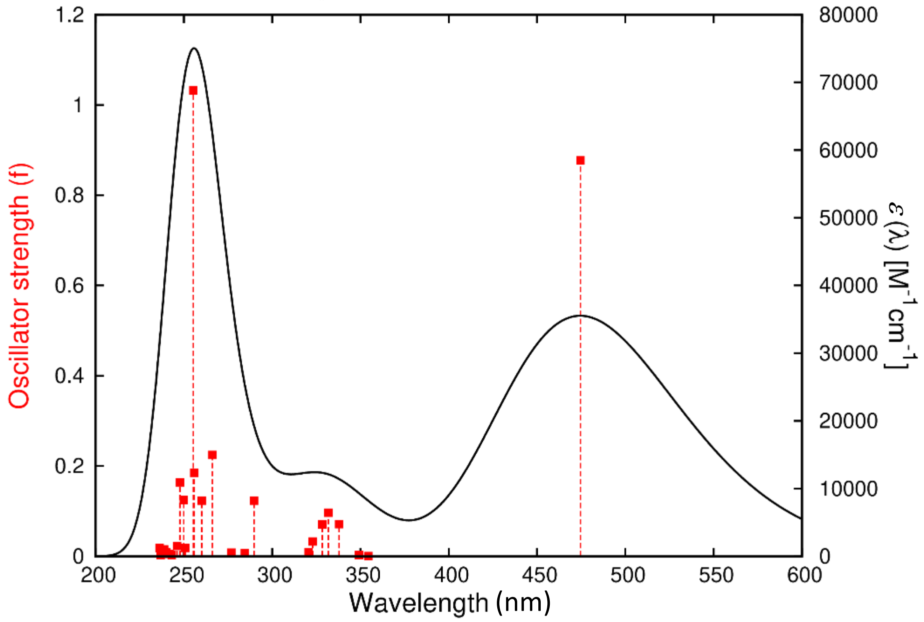

Supplementary Figure 35. UV-vis absorption properties of Envelope-1-1_S under high polarity conditions. TD-DFT simulated UV-vis spectrum of **Envelope-1-1_S** in acetonitrile.

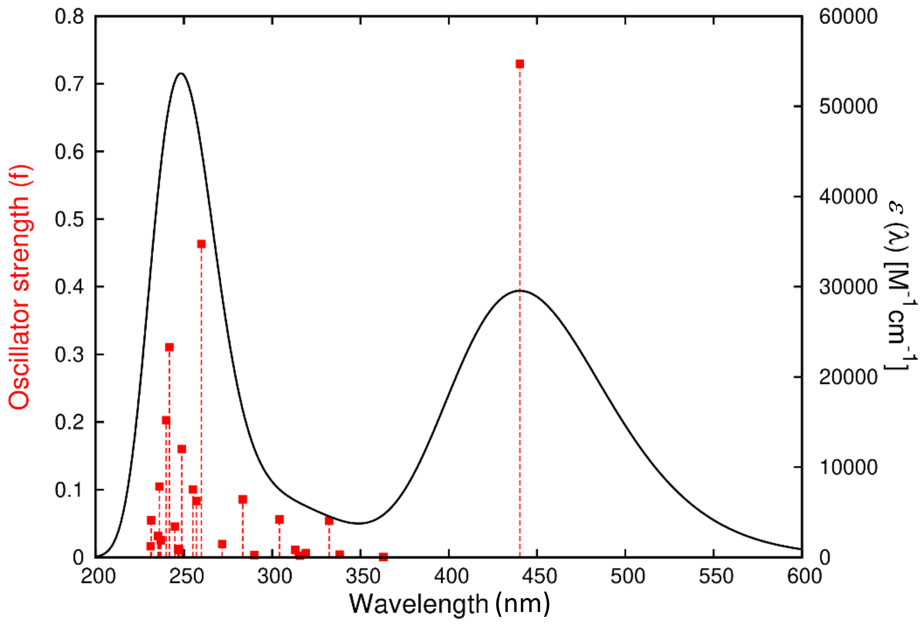

Supplementary Figure 36. UV-vis absorption properties of Envelope-1-1$_S$ under low polarity conditions. TD-DFT simulated UV-vis spectrum of **Envelope-1-1$_S$** *in vacuo*.

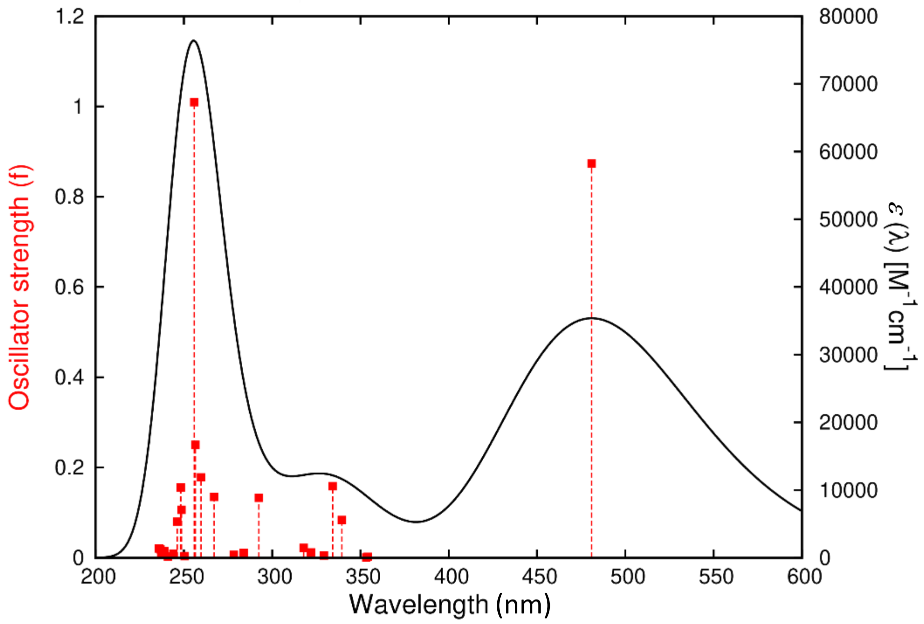

Supplementary Figure 37. UV-vis absorption properties of Envelope-1-Inv-1$_S$ under high polarity conditions. TD-DFT simulated UV-vis spectrum of **Envelope-1-Inv-1$_S$** in acetonitrile.

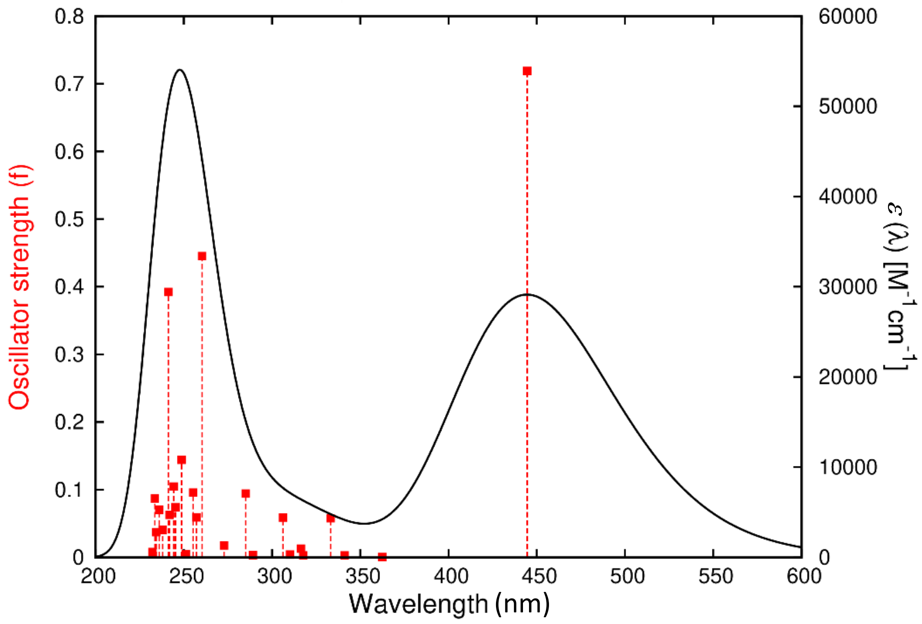

Supplementary Figure 38. UV-vis absorption properties of Envelope-1-Inv-1_S under low polarity conditions. TD-DFT simulated UV-vis spectrum of **Envelope-1-Inv-1_S** *in vacuo*.

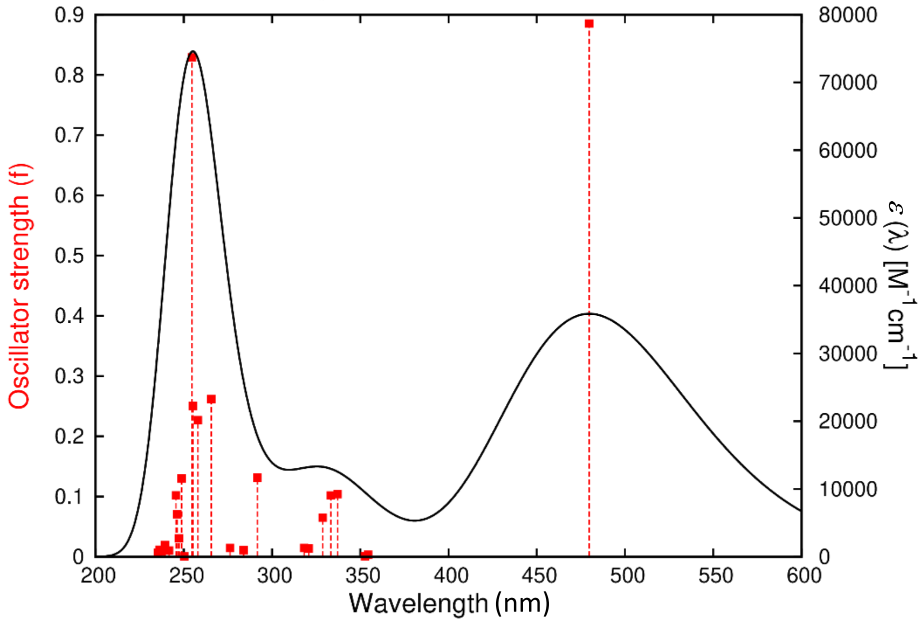

Supplementary Figure 39. UV-vis absorption properties of Envelope-2-1_S under high polarity conditions. TD-DFT simulated UV-vis spectrum of **Envelope-2-1_S** in acetonitrile.

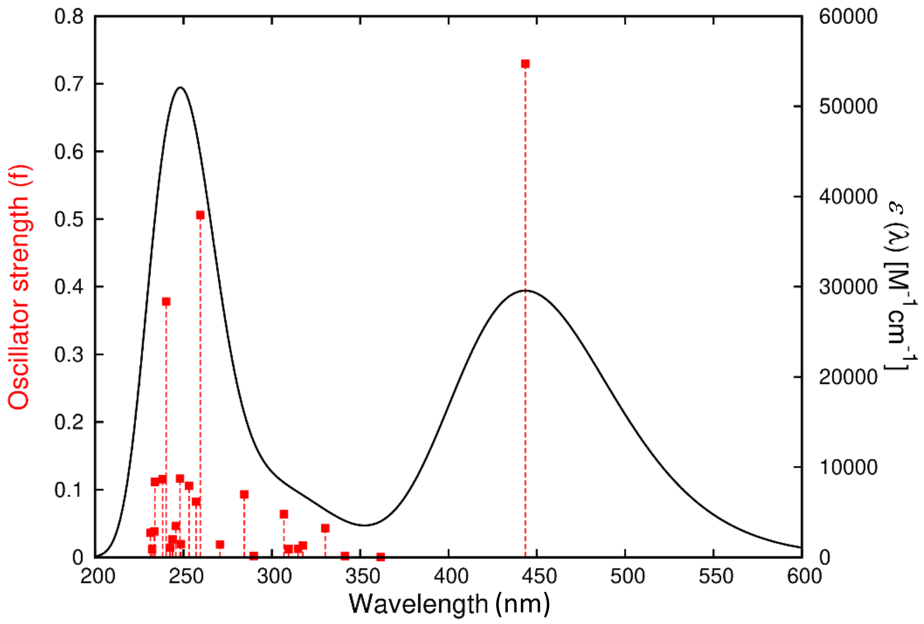

Supplementary Figure 40. UV-vis absorption properties of Envelope-2-1$_S$ under low polarity conditions. TD-DFT simulated UV-vis spectrum of **Envelope-2-1$_S$** *in vacuo*.

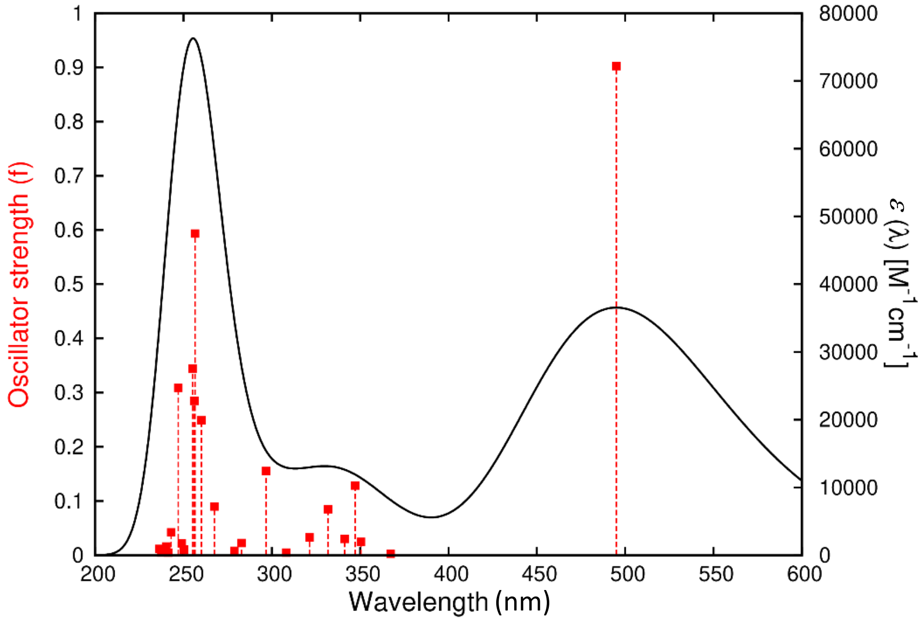

Supplementary Figure 41. UV-vis absorption properties of Envelope-2-Inv-1$_S$ under high polarity conditions. TD-DFT simulated UV-vis spectrum of **Envelope-2-Inv-1$_S$** in acetonitrile.

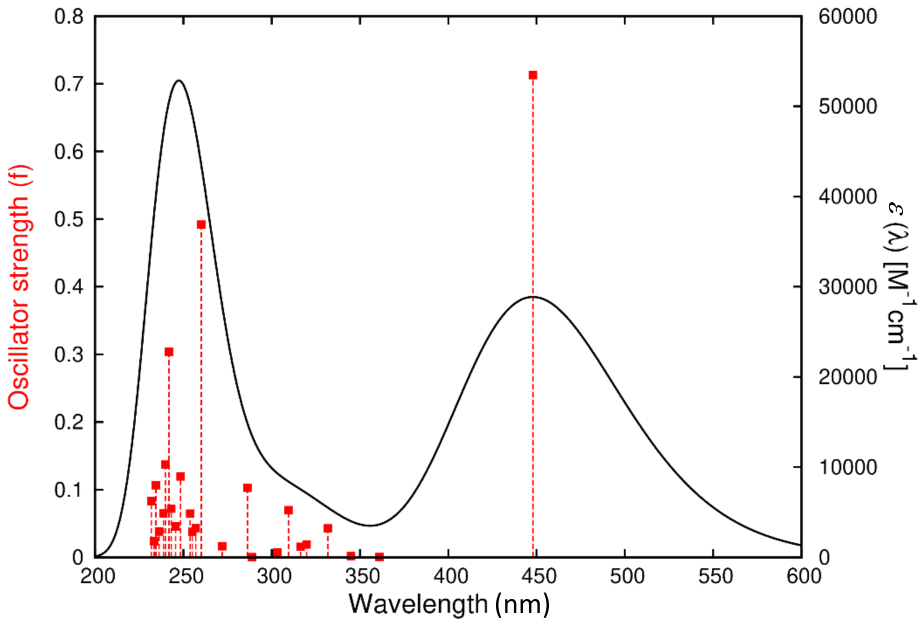

Supplementary Figure 42. UV-vis absorption properties of Envelope-2-Inv-1$_S$ under low polarity conditions. TD-DFT simulated UV-vis spectrum of **Envelope-2-Inv-1$_S$** *in vacuo*.

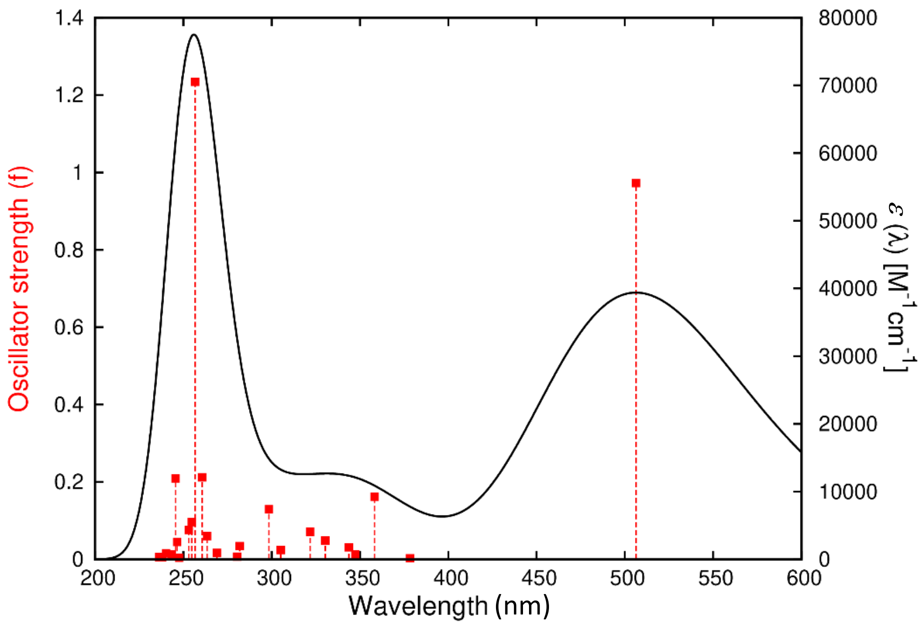

Supplementary Figure 43. UV-vis absorption properties of Half-Chair-1-1$_S$ under high polarity conditions. TD-DFT simulated UV-vis spectrum of **Half-Chair-1-1$_S$** in acetonitrile.

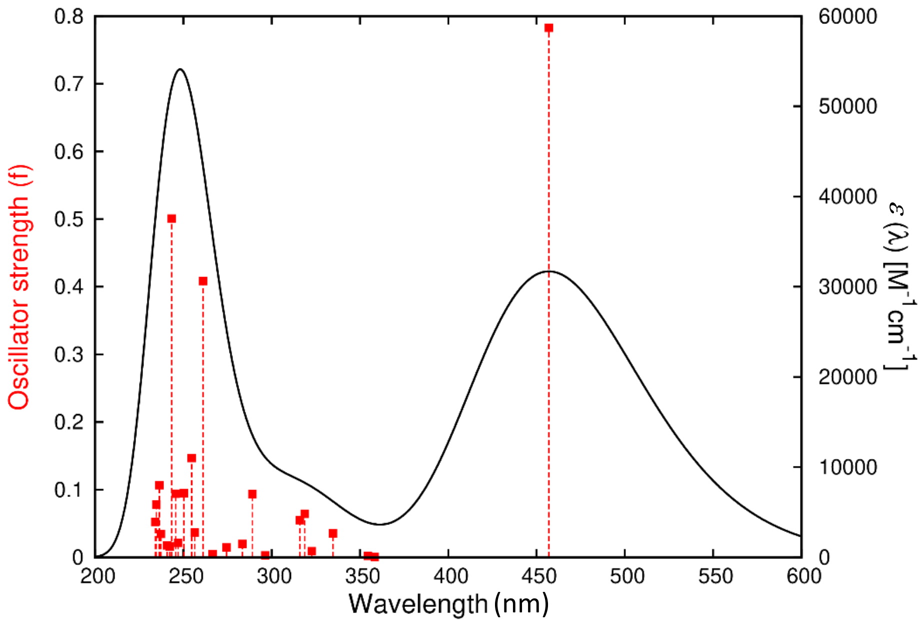

Supplementary Figure 44. UV-vis absorption properties of Half-Chair-1-1$_S$ under low polarity conditions. TD-DFT simulated UV-vis spectrum of **Half-Chair-1-1$_S$** *in vacuo*.

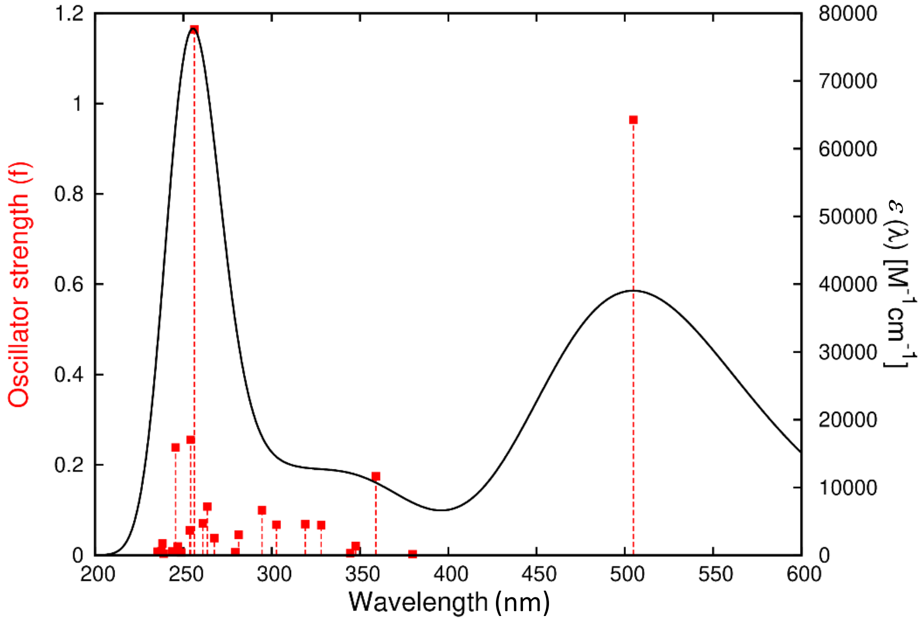

Supplementary Figure 45. UV-vis absorption properties of Half-Chair-2-1$_S$ under high polarity conditions. TD-DFT simulated UV-vis spectrum of **Half-Chair-2-1$_S$** in acetonitrile.

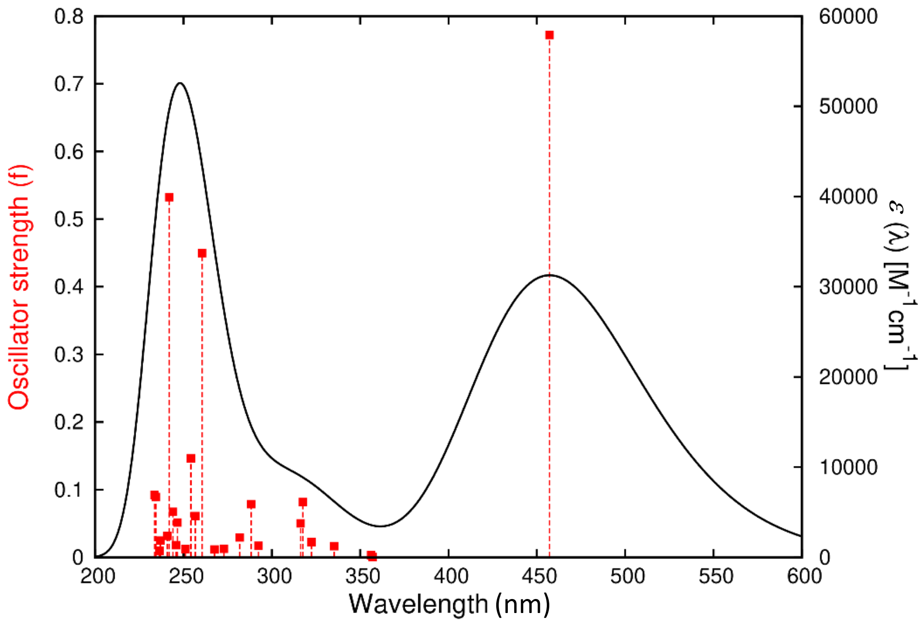

Supplementary Figure 46. UV-vis absorption properties of Half-Chair-2-1$_S$ under low polarity conditions. TD-DFT simulated UV-vis spectrum of **Half-Chair-2-1$_S$** *in vacuo*.

8. NMR Spectra

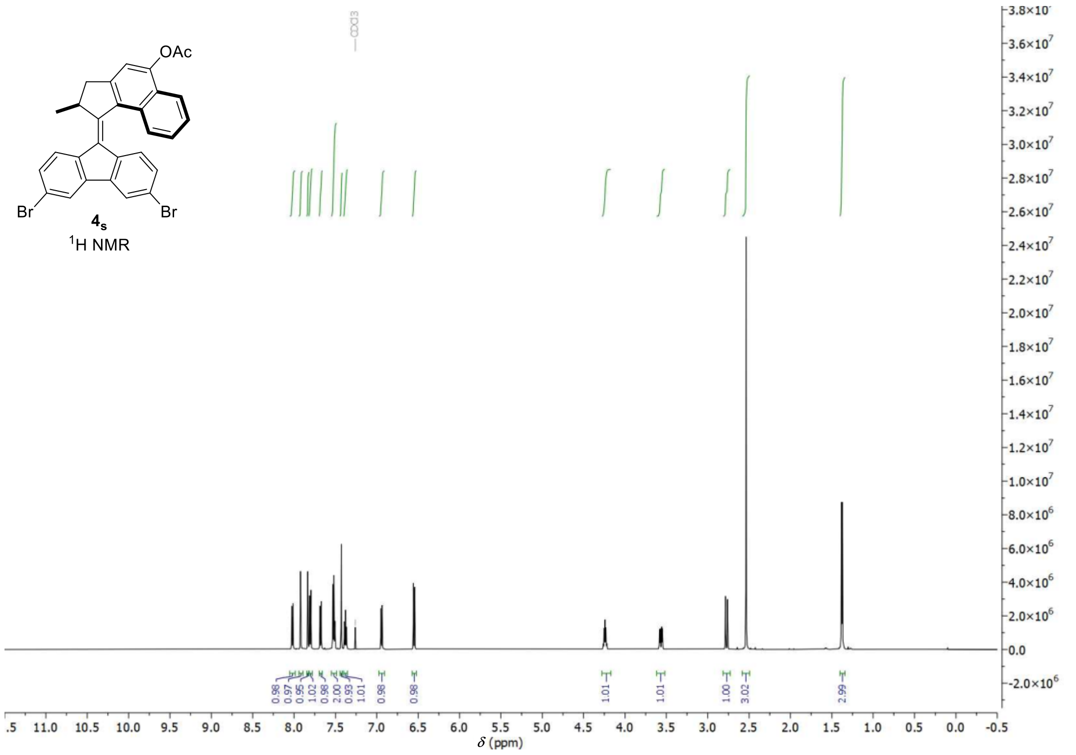

Supplementary Figure 47. ^{1}H NMR spectrum of 4_s. Conditions: 600 MHz, $CDCl_3$, 25 °C.

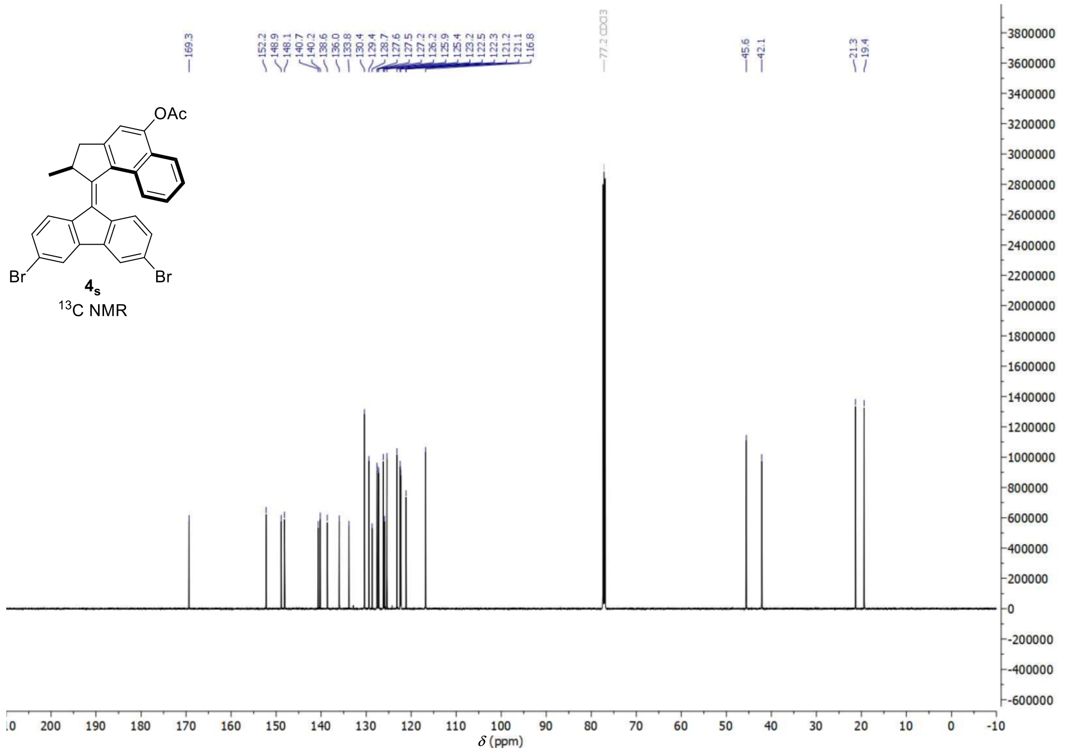

Supplementary Figure 48. ^{13}C NMR spectrum of 4_s. Conditions: 151 MHz, $CDCl_3$, 25 °C.

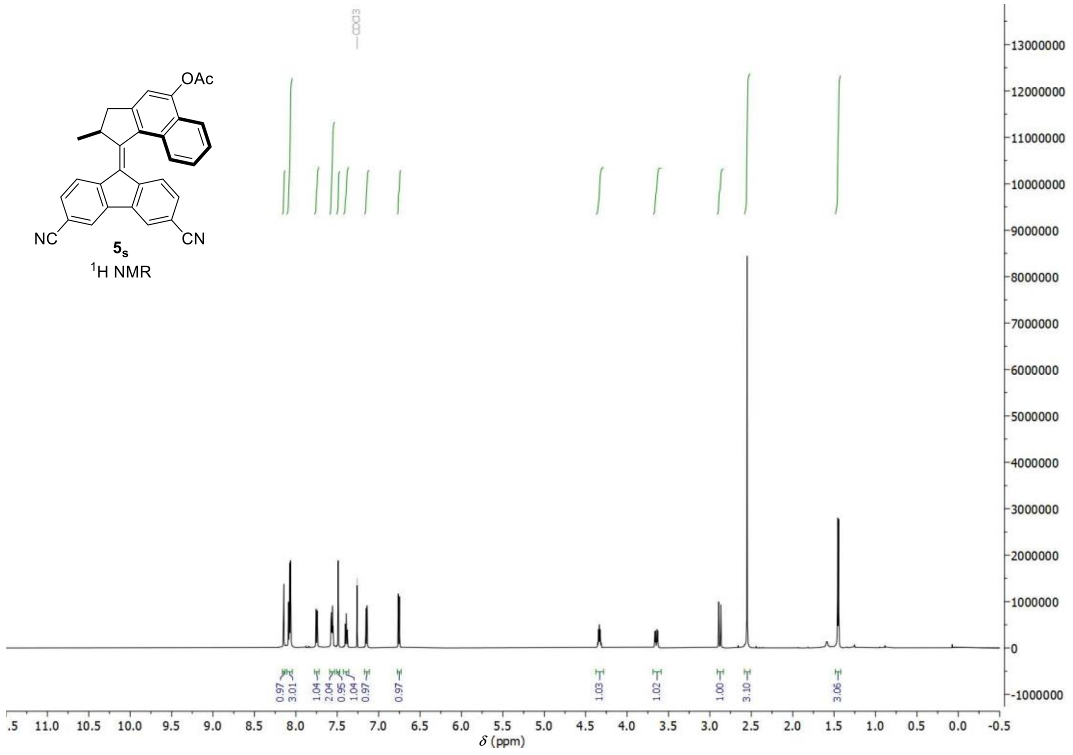

Supplementary Figure 49. ^{1}H NMR spectrum of 5_s. Conditions: 600 MHz, $CDCl_3$, 25 °C.

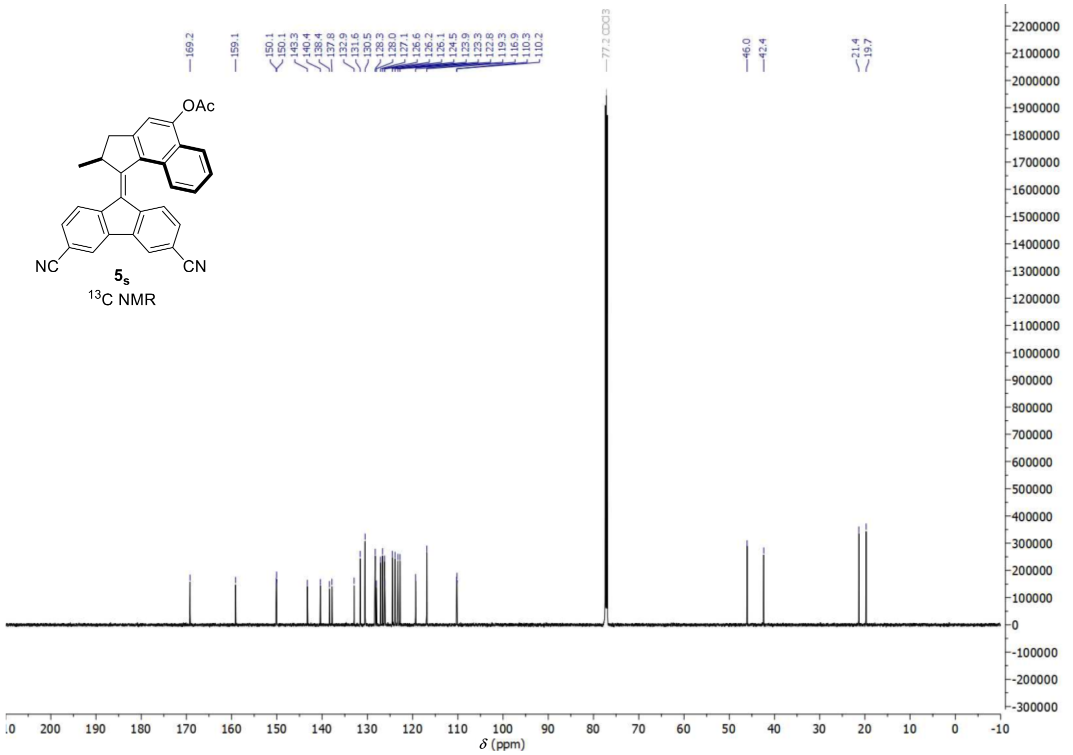

Supplementary Figure 50. ^{13}C NMR spectrum of 5_s. Conditions: 151 MHz, $CDCl_3$, 25 °C.

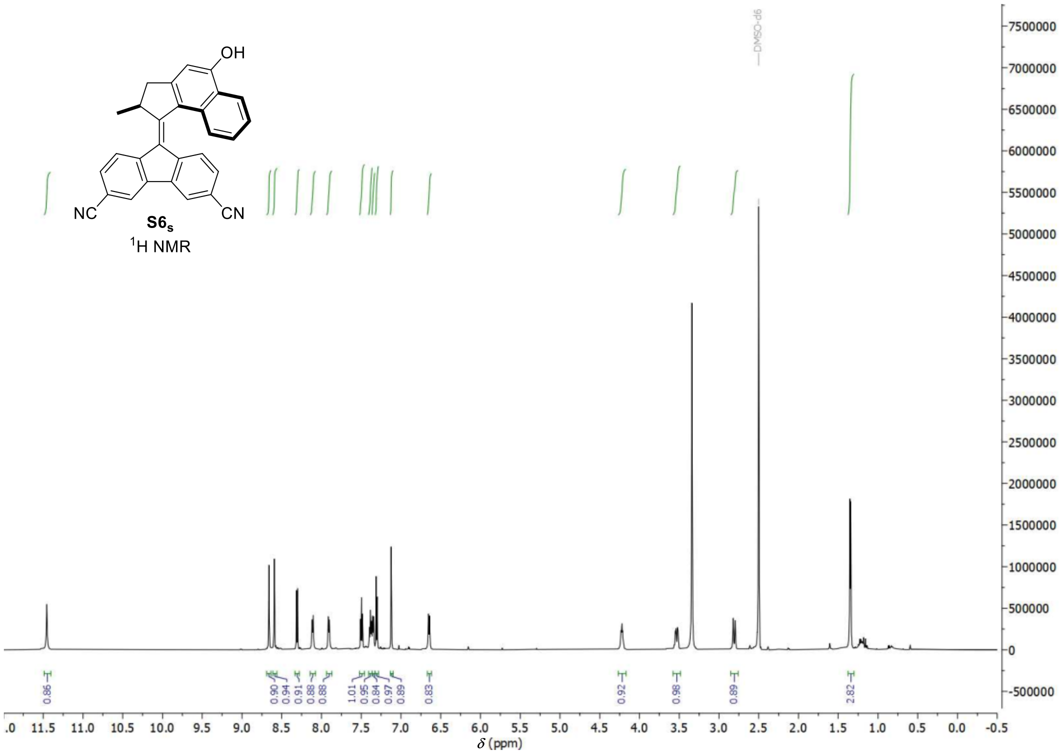

Supplementary Figure 51. ^{1}H NMR spectrum of S6$_{s}$. Conditions: 600 MHz, DMSO-d_6, 25 °C.

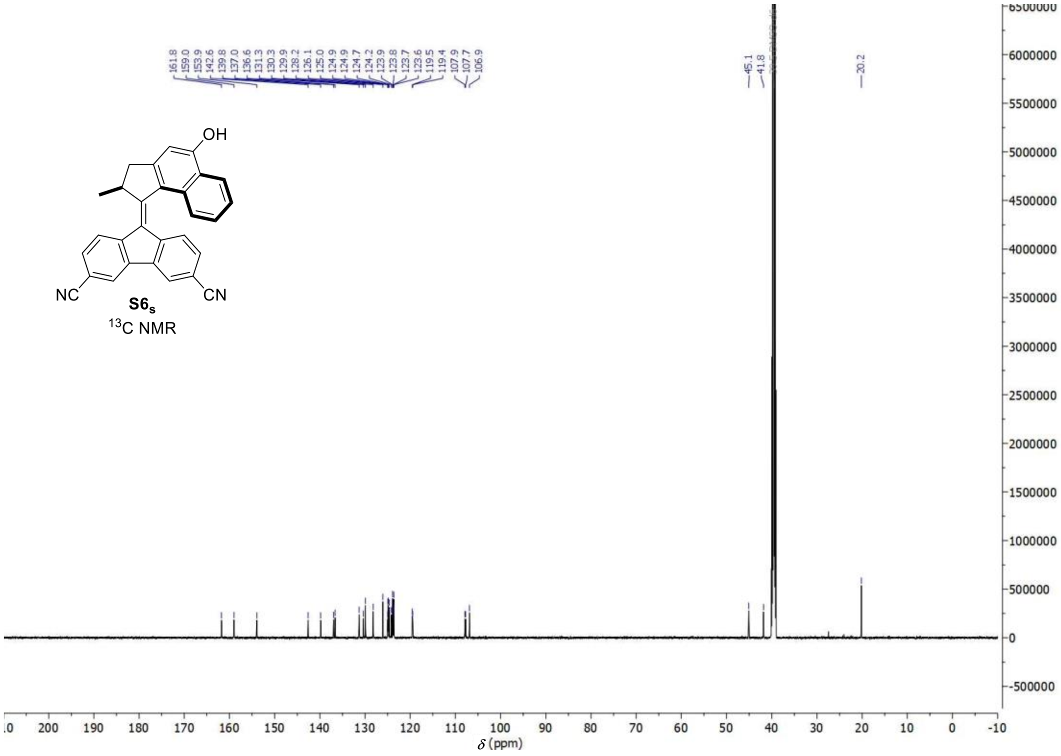

Supplementary Figure 52. ^{13}C NMR spectrum of S6$_{s}$. Conditions: 151 MHz, DMSO-d_6, 25 °C.

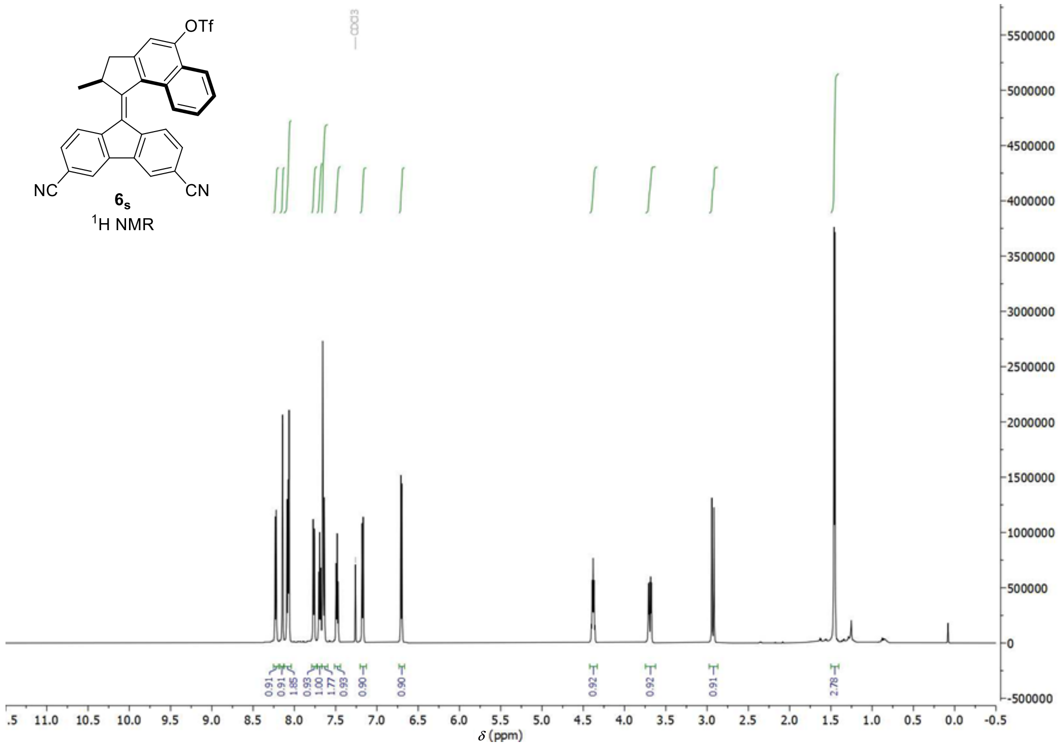

Supplementary Figure 53. ^{1}H NMR spectrum of 6_s. Conditions: 600 MHz, $CDCl_3$, 25 °C.

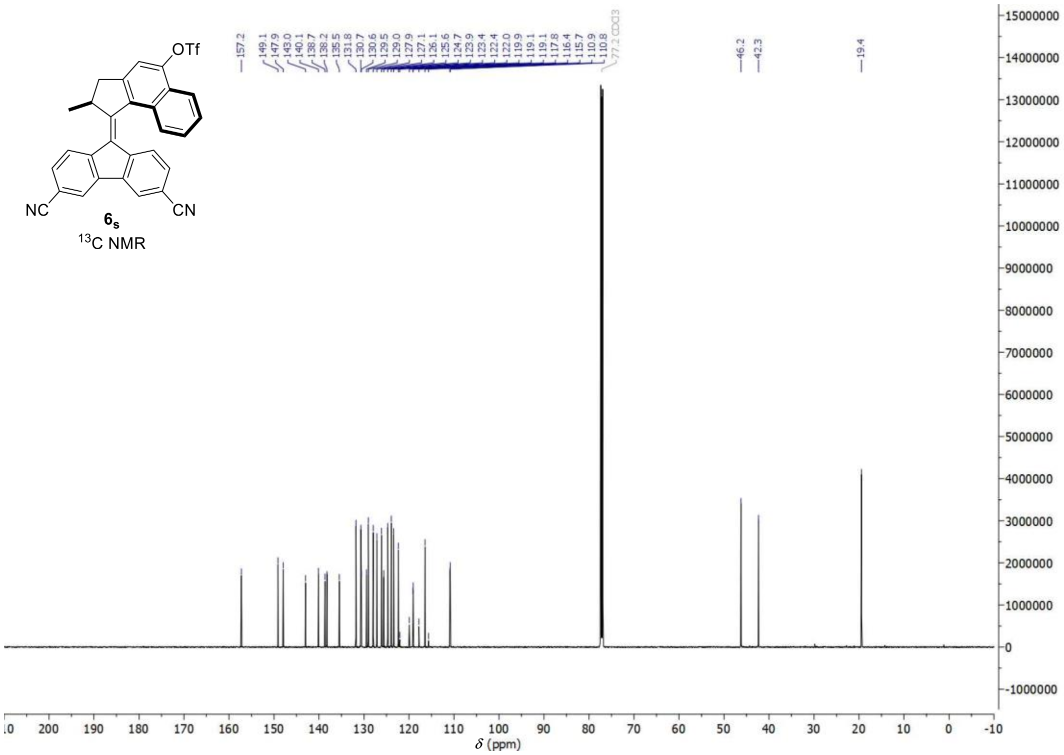

Supplementary Figure 54. ^{13}C NMR spectrum of 6_s. Conditions: 151 MHz, $CDCl_3$, 25 °C.

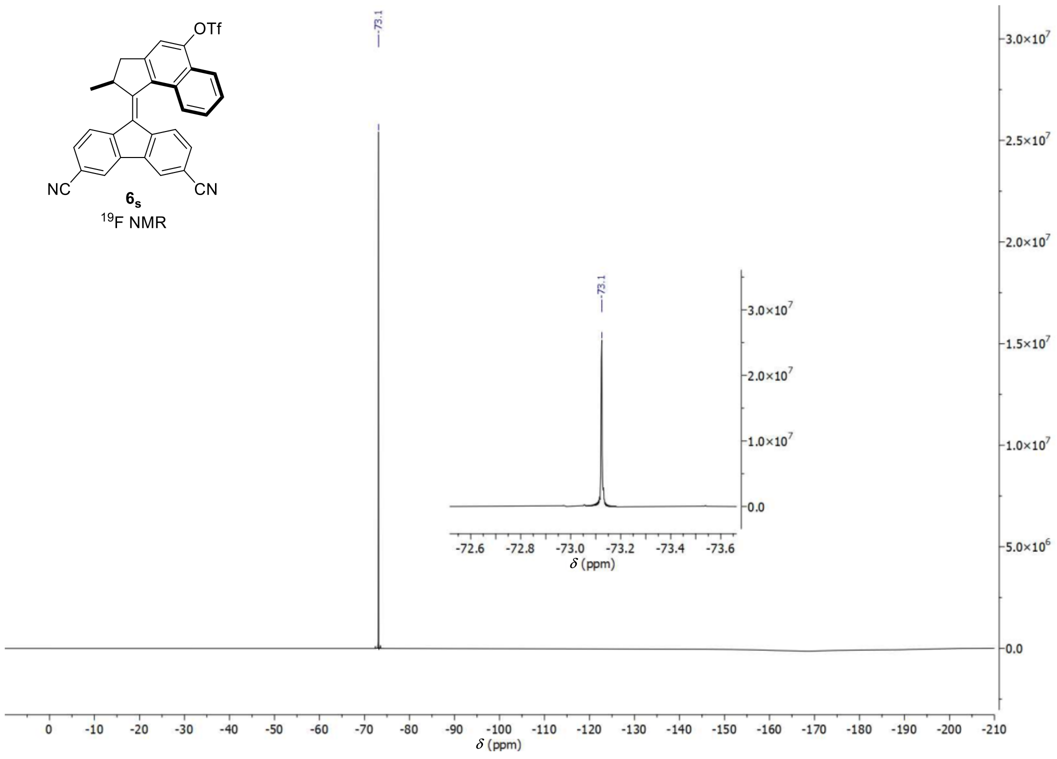

Supplementary Figure 55. ^{19}F NMR spectrum of 6_s. Conditions: 565 MHz, $CDCl_3$, 25 °C.

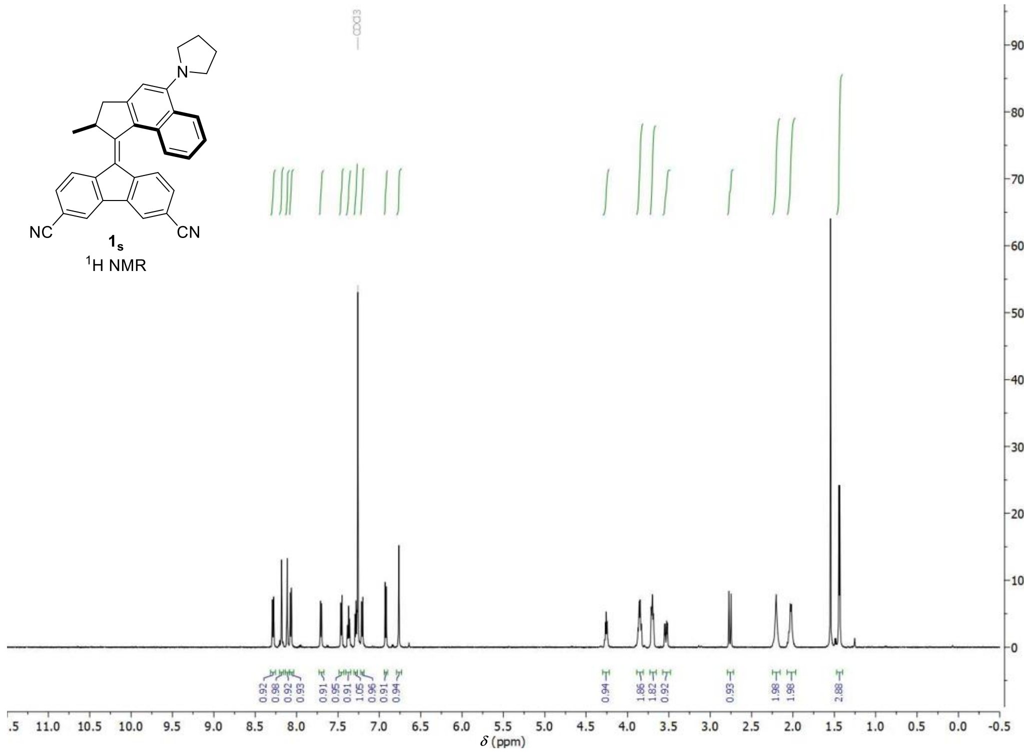

Supplementary Figure 56. ^{1}H NMR spectrum of 1_s. Conditions: 600 MHz, $CDCl_3$, 25 °C.

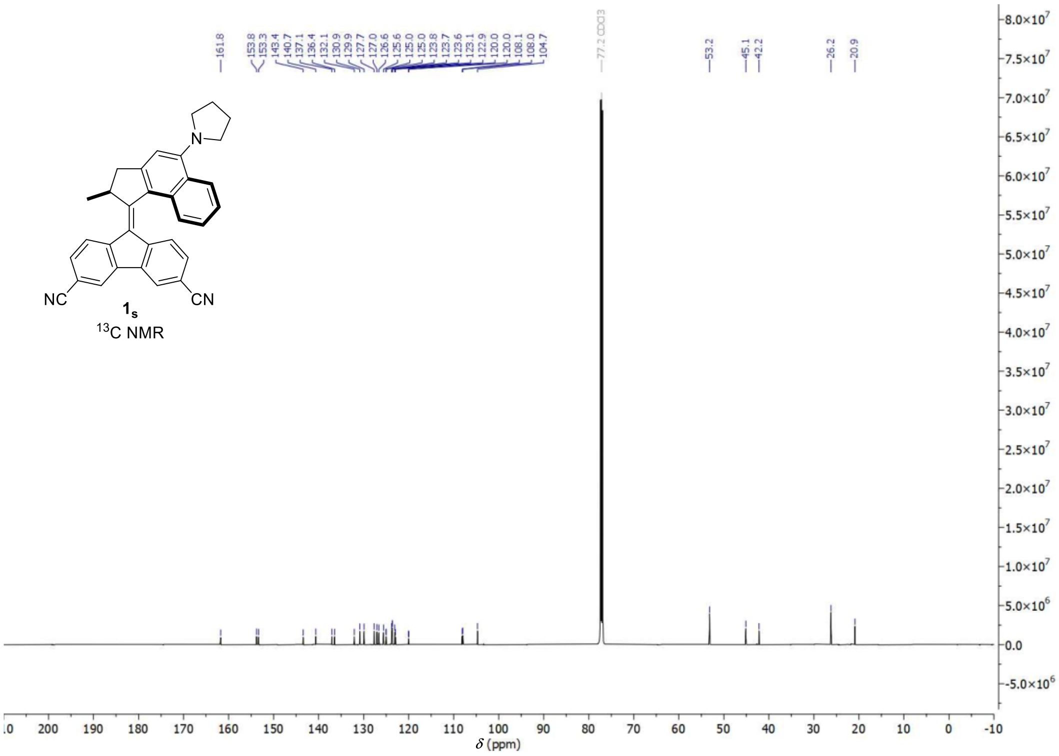

Supplementary Figure 57. ^{13}C NMR spectrum of 1_s. Conditions: 151 MHz, $CDCl_3$, 25 °C.

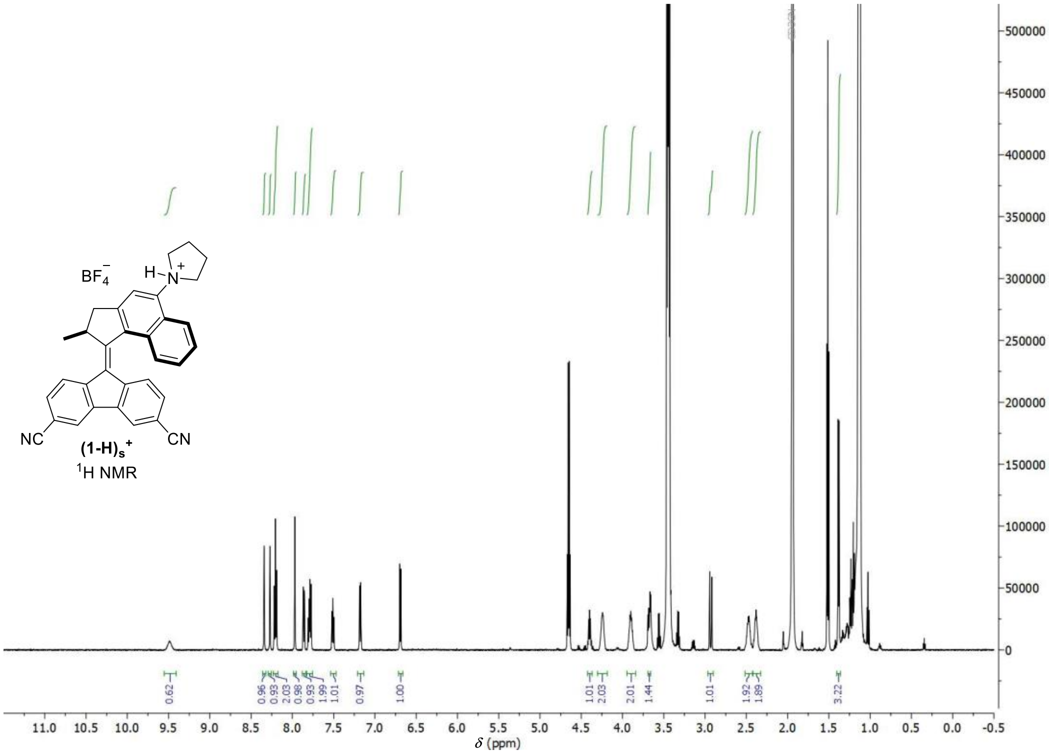

Supplementary Figure 58. ^{1}H NMR spectrum of $(1–H)_sBF_4$. Conditions: 600 MHz, MeCN-d_3, 25 °C.

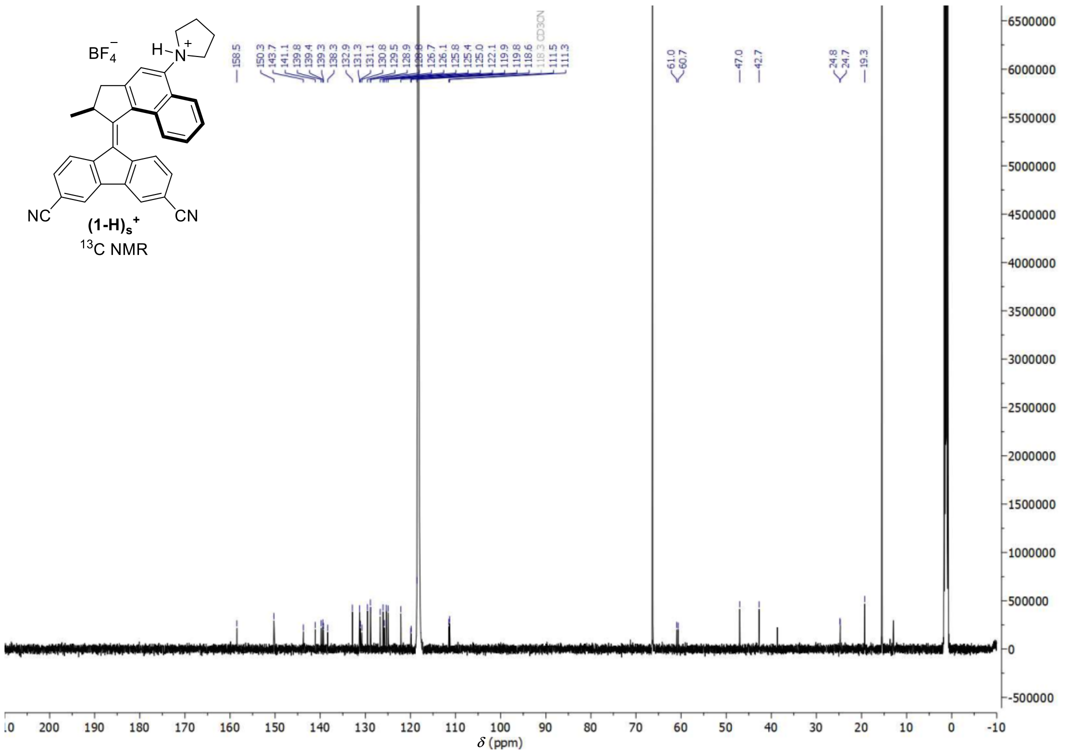

Supplementary Figure 59. ^{13}C NMR spectrum of (1–H)$_s$BF$_4$. Conditions: 151 MHz, MeCN-d_3, 25 °C.

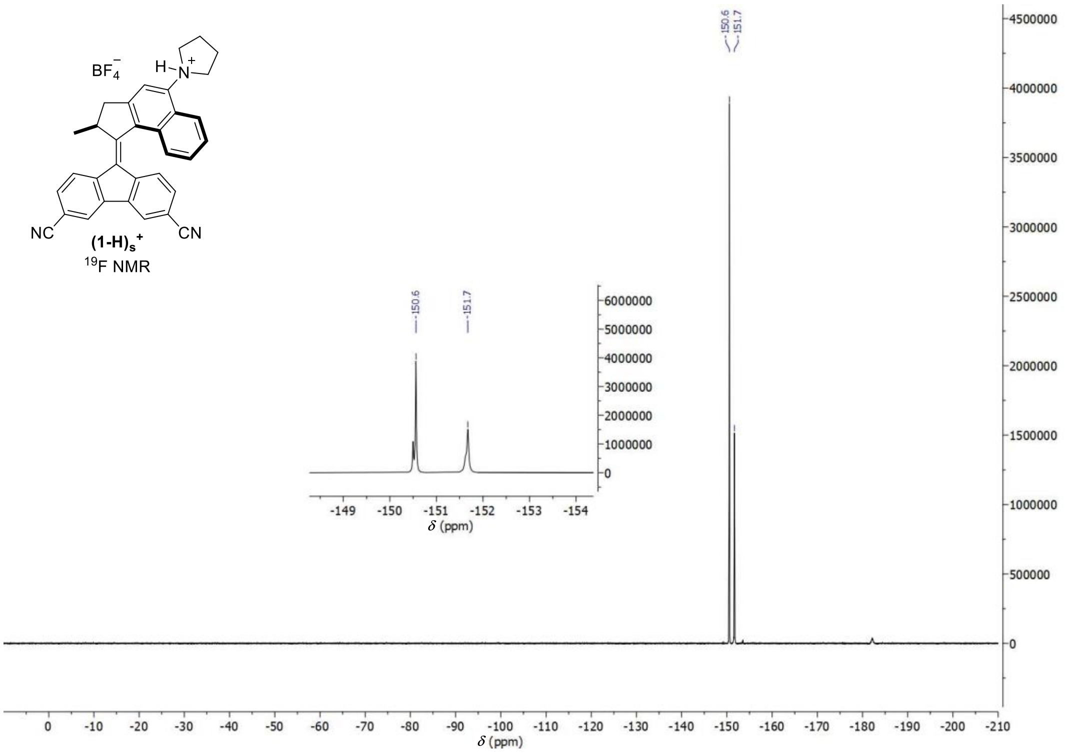

Supplementary Figure 60. ^{19}F NMR spectrum of (1–H)$_s$BF$_4$. Conditions: 565 MHz, MeCN-d_3, 25 °C.

9. Supplementary References

1. M. M. Pollard, P. V. Wesenhagen, D. Pijper, B. L. Feringa, On the effect of donor and acceptor substituents on the behaviour of light-driven rotary molecular motors. *Org. Biomol. Chem.* **6**, 1605–1612 (2008).

2. A. Cnossen, L. Hou, M. M. Pollard, P. V. Wesenhagen, W. R. Browne, B. L. Feringa, Driving Unidirectional Molecular Rotary Motors with Visible Light by Intra- And Intermolecular Energy Transfer from Palladium Porphyrin. *J. Am. Chem. Soc.* **134**, 17613–17619 (2012).

3. J. Conyard, P. Stacko, J. Chen, S. McDonagh, C. R. Hall, S. P. Laptenok, W. R. Browne, B. L. Feringa, S. R. Meech, Ultrafast Excited State Dynamics in Molecular Motors: Coupling of Motor Length to Medium Viscosity. *J. Phys. Chem. A*. **121**, 2138–2150 (2017).

4. Bruker, APEX3 (V2019.1-0), SAINT (Version 8.40A) and SADABS (Version 2016/1). Bruker AXS Inc., Madison, Wisconsin, USA.

5. G. M. Sheldrick, SHELXT - Integrated space-group and crystal-structure determination. *Acta Crystallogr. Sect. A Found. Crystallogr.* **71**, 3–8 (2015).

6. G. M. Sheldrick, A short history of SHELX. *Acta Crystallogr Sect. A*. **A64**, 112–122 (2008).

7. A. L. Spek, Single-crystal structure validation with the program PLATON. *J. Appl. Crystallogr.* **36**, 7–13 (2003).

8. K. Stott, J. Keeler, O.N. Van and A.J. Shaka, *J. Magn. Reson.* **125**, 302-324 (1997).

9. C. Reichardt, *Solvents and Solvent Effects in Organic Chemistry* (Wiley-VCH, Weinheim, ed. 3rd, 2003).

10. M. J. Kamlet, R. W. Taft, The Solvatochromic Comparison Method. I. The β-Scale Of Solvent Hydrogen-Bond Acceptor (HBA) Basicities. *J. Am. Chem. Soc.* **98**, 377–383 (1976).

11. R. W. Taft, M. J. Kamlet, The Solvatochromic Comparison Method. 2. The α-Scale of Solvent Hydrogen-Bond Donor (HBD) Acidities. *J. Am. Chem. Soc.* **98**, 2886–2894 (1976).

12. M. J. Kamlet, J. L. Abboud, R. W. Taft, The Solvatochromic Comparison Method. 6. The π* Scale of Solvent Polarities. *J. Am. Chem. Soc.* **99**, 6027–6038 (1977).

13. M. J. Frisch, G. W. Trucks, H. B. Schlegel, G. E. Scuseria, M. A. Robb, J. R. Cheeseman, G. Scalmani, V. Barone, G. A. Petersson, H. Nakatsuji, X. Li, M. Caricato, A. V. Marenich, J. Bloino, B. G. Janesko, R. Gomperts, B. Mennucci, H. P. Hratchian, J. V. Ortiz, A. F. Izmaylov, J. L. Sonnenberg, D. Williams-Young, F. Ding, F. Lipparini, F. Egidi, J. Goings, B. Peng, A. Petrone, T. Henderson, D. Ranasinghe, V. G. Zakrzewski, J. Gao, N. Rega, G. Zheng, W. Liang, M. Hada, M. Ehara, K. Toyota, R. Fukuda, J. Hasegawa, M. Ishida, T. Nakajima, Y. Honda, O. Kitao, H. Nakai, T. Vreven, K. Throssell, J. A. Montgomery Jr., J. E. Peralta, F. Ogliaro, M. J. Bearpark, J. J. Heyd, E. N. Brothers, K. N. Kudin, V. N. Staroverov, T. A. Keith, R. Kobayashi, J. Normand, K. Raghavachari, A. P. Rendell, J. C. Burant, S. S. Iyengar, J. Tomasi, M. Cossi, J. M. Millam, M. Klene, C. Adamo, R. Cammi, J. W. Ochterski, R. L. Martin, K. Morokuma, O. Farkas, J. B. Foresman, D. J. Fox, *Gaussian 16, Revision B.01* (Gaussian, Inc., Wallingford, CT, USA, 2016).

14. Aquilante, F. *et al.* Modern quantum chemistry with [Open]Molcas. *J. Chem. Phys.* **152**, 214117 (2020).

15. E. D. Glendening, C. R. Landis, F. Weinhold, NBO 6.0: Natural bond orbital analysis program. *J. Comput. Chem.* **34**, 1429–1437 (2013).
