# Supplementary material for: Controlling forward and backward rotary molecular motion on demand
Source: Nat Commun. 2022 Apr 19;13:2124. doi: 10.1038/s41467-022-29820-5 (PMC9019045; doi:10.1038/s41467-022-29820-5)
Supplement: Supplementary file 3 — Description of Additional Supplementary Files [file 41467_2022_29820_MOESM3_ESM.pdf]

Description of Additional Supplementary Files

Supplementary Data 1: Computed cartesian coordinates and energies for the structures analyzed in this work.
