# Supplementary material for: Controlling forward and backward rotary molecular motion on demand
Source: Nat Commun. 2022 Apr 19;13:2124. doi: 10.1038/s41467-022-29820-5 (PMC9019045; doi:10.1038/s41467-022-29820-5)
Supplement: Supplementary file 4 — Supplementary Data 1 [file 41467_2022_29820_MOESM4_ESM.pdf]

Coordinates_energies

1-H-cam-b3lyp-mecn
stable

C 2.82595746 3.5714366195 -0.3419554779
C 1.4605705176 3.2758864844 -0.4482869159
C 1.0154362489 1.9653282539 -0.3606309441
C 1.9335007444 0.9369016255 -0.1467414632
C 3.3084184401 1.2478926155 -0.0995530018
C 3.766724847 2.5509097146 -0.1815204514
H 0.7504434067 4.0796593317 -0.6055605017
H -0.0401630725 1.7671381976 -0.475824346
H 4.8260138143 2.7794959313 -0.1386933598
C 3.1528771531 -1.0771977053 -0.0575839375
C 3.64578668 -2.3803492488 0.0271431136
C 5.0144296146 -2.593326295 0.1084698652
C 5.903204717 -1.5110367493 0.1248231092
C 5.4295519118 -0.1976985489 0.0663181268
C 4.0634484805 0.0003567904 -0.0216332464
H 2.9922431524 -3.2401170692 0.0416694818
H 5.4013966844 -3.6043225977 0.1653541343
H 6.1198465139 0.6383584686 0.0921176008
C 1.7709099049 -0.5369037801 -0.0978897608
C -0.778460394 -0.8244229255 -0.0177421559
C -1.559965836 -1.6034501955 -0.8468916699
C 0.6238086791 -1.2659706769 -0.1584798313
C -1.3892793793 0.1012868136 0.8810340193
C -2.7932984026 0.3363689595 0.7641902932
C -0.6775351128 0.7444471546 1.9232654101
H 0.372583243 0.5173121207 2.0557227551
C -1.2995213916 1.6241032224 2.7682852384
H -0.738780021 2.0986594637 3.5665419388
C -2.6684691965 1.9158908226 2.6039374754
H -3.1518412419 2.6319566734 3.2597234054
C -3.3974090206 1.2854838974 1.6311253641
H -4.449780696 1.5323131964 1.5415604338
C -3.5283462011 -0.4274998058 -0.1925287641
C -2.9429985221 -1.3926274032 -0.9637754107
H -3.5174812101 -1.9993964028 -1.6519066952
C 0.5374246629 -2.7110168039 -0.6359463582
H 1.4064547848 -2.9735725593 -1.2389543765
C -0.7405517684 -2.6705255639 -1.512952591
H -1.2593243548 -3.6319973438 -1.5489336678
H -0.4905971623 -2.3878734371 -2.5417194941
C 0.3756788365 -3.7145817043 0.5119098627
H -0.5425167776 -3.5185374729 1.0730890692
H 0.3193281008 -4.7312307268 0.1131645143
H 1.2110014949 -3.6693122461 1.2147494472
C 7.3128101078 -1.7539920862 0.2106529985
C 3.2623882366 4.9338281683 -0.4245678276
N 8.4513747727 -1.9505969397 0.2789636619
N 3.6138112464 6.034702223 -0.4901308147
C -5.88433596 -1.3028612477 -0.6873071633
C -5.3169990448 0.9281077686 -1.325481868
C -7.2048126893 -0.5839113052 -0.9030472444
H -5.8692533842 -2.0363832488 0.1173122043
H -5.5190673113 -1.7455672818 -1.6123330017
C -6.8153825649 0.7367062948 -1.6018644742
H -4.6992637514 0.7404884395 -2.2026948384
H -5.0467955017 1.8872164566 -0.8869727358
H -7.8867746973 -1.1918578004 -1.4980865904
H -7.6813021662 -0.3857571217 0.0606421111
H -6.9797435496 0.6774605389 -2.6785748034
H -7.3989057095 1.5743379406 -1.2188026777
N -4.9675327155 -0.1613463577 -0.3254823505
H -5.3025115303 0.1592774038 0.5889517669

Energy= -1436.5405494

Zero-point correction= 0.514042 (Hartree/Particle)
Thermal correction to Gibbs Free Energy= 0.455039
Sum of electronic and zero-point Energies= -1436.026508
Sum of electronic and thermal Energies= -1435.998268
Sum of electronic and thermal Enthalpies= -1435.997324
Sum of electronic and thermal Free Energies= -1436.085511
Imaginary Frequencies 0

TS-EZ

C -4.022739 3.144129 0.084125
C -2.734918 3.468039 0.584806
C -1.810519 2.484609 0.841224
C -2.148733 1.131915 0.605276
C -3.45335 0.812612 0.091745
C -4.377114 1.815512 -0.161451
H -2.487253 4.50915 0.763147
H -0.829382 2.750494 1.222551
H -5.364918 1.579998 -0.54584
C -2.250995 -1.149271 0.375068
C -2.032453 -2.545664 0.316399
C -3.031908 -3.369235 -0.141547
C -4.279319 -2.837484 -0.560584
C -4.515993 -1.461596 -0.517942
C -3.515796 -0.618363 -0.057557
H -1.082231 -2.968339 0.627197
H -2.875017 -4.441692 -0.189744
H -5.472142 -1.06532 -0.846022
C -1.419239 -0.072695 0.775232
C 1.145438 -0.116185 0.589532
C 2.208768 -0.238849 1.50751
C -0.062905 -0.168503 1.299936
C 1.408831 0.030888 -0.826346
C 2.770715 0.035231 -1.251178
C 0.397363 0.170388 -1.789892
H -0.635484 0.164822 -1.472659
C 0.710211 0.314496 -3.12332
H -0.086126 0.421306 -3.852083
C 2.046522 0.327766 -3.544891
H 2.285431 0.448115 -4.595504
C 3.058317 0.190312 -2.627045
H 4.077916 0.21783 -2.993952
C 3.791901 -0.111111 -0.255927
C 3.539183 -0.221188 1.085438
H 4.342247 -0.305963 1.808409
C 0.1998 -0.278294 2.770237
H -0.135922 0.692806 3.168991
C 1.719536 -0.395731 2.898873
H 2.019742 -1.374672 3.290149
H 2.161599 0.354446 3.562148
C -0.599964 -1.369133 3.486882
H -0.323853 -2.36001 3.117679
H -0.384352 -1.328867 4.556822
H -1.672178 -1.22649 3.345457
C -5.295997 -3.716745 -1.034658
C -4.961173 4.186184 -0.171174
N -6.121705 -4.434637 -1.420893
N -5.722748 5.036701 -0.378295
C 6.100061 -1.189936 -0.051596
C 5.886236 1.241253 -0.439702
C 7.194612 -0.414636 0.664928
H 6.48711 -1.764303 -0.892315
H 5.497435 -1.837655 0.579395
C 7.328837 0.86366 -0.160668
H 5.410106 1.688981 0.431596
H 5.714452 1.860629 -1.318455
H 6.888525 -0.177646 1.687659
H 8.11539 -0.998097 0.705682

H 7.84031 1.667244 0.371222
H 7.862699 0.669475 -1.095249
N 5.195285 -0.1089 -0.675863
H 5.230772 -0.28263 -1.681306

Energy= -1436.4870387
Zero-point correction= 0.510567 (Hartree/Particle)
Thermal correction to Gibbs Free Energy= 0.451358
Sum of electronic and zero-point Energies= -1435.976472
Sum of electronic and thermal Energies= -1435.948469
Sum of electronic and thermal Enthalpies= -1435.947525
Sum of electronic and thermal Free Energies= -1436.035681
Imaginary Frequencies 1

TS-THI

C -3.3265208173 3.3845887751 0.0827252231
C -2.4646776147 3.3906962519 -1.018547923
C -1.8469104087 2.2165759836 -1.4304925747
C -2.0381145598 1.0365393555 -0.7159251624
C -3.0560346421 1.0219784674 0.2614178097
C -3.6677098329 2.1795204583 0.7054509074
H -2.2987874758 4.3107463654 -1.567040619
H -1.2812630064 2.2326690814 -2.3512826608
H -4.431866535 2.1541204735 1.4746643793
C -2.7166698143 -1.184284513 -0.4181463175
C -3.1458519165 -2.4999317574 -0.6049954717
C -4.1992660785 -3.005009247 0.1498461418
C -4.8489305643 -2.2048163103 1.092025949
C -4.4946112288 -0.8594212682 1.2384695032
C -3.4612719432 -0.3668059805 0.4664956385
H -2.7257033975 -3.140703438 -1.3658902457
H -4.5281964046 -4.0262228141 -0.0053606524
H -5.0380581373 -0.2173682531 1.9229899871
C -1.5690214473 -0.3624420732 -0.9443922224
C 1.0969198101 -0.46238194 -0.9505206977
C 1.8652985909 -1.5961531179 -0.7422734573
C -0.3269214878 -0.9138881138 -1.1601255669
C 1.7663453519 0.8089329313 -0.7940018009
C 3.0847476502 0.8598590603 -0.2335471929
C 1.2231335023 2.0278899644 -1.2392157815
H 0.2997748633 1.9887029623 -1.776543133
C -3.9346481499 4.607988556 0.5148777415
C -5.916826043 -2.751398409 1.8751040566
N -4.4216523231 5.5971531269 0.8668342549
N -6.7773211202 -3.1939138231 2.5102199283
C 1.8480730754 3.2343125512 -1.0587134132
H 1.3837923005 4.1431609759 -1.4271124128
C 3.0913171904 3.2866186099 -0.4098152056
H 3.585472323 4.2373404392 -0.2415508869
C 3.6984078463 2.1209993074 -0.0222481148
H 4.673927927 2.1911975119 0.4460720276
C 3.7559232089 -0.3679875801 0.0466818542
C 3.1806752247 -1.5634125204 -0.2438500463
H 3.6881555573 -2.5044996504 -0.075116219
C -0.2452688495 -2.4178649013 -1.4358931501
H -0.9550772072 -2.9463215134 -0.8063256084
C 1.1578295656 -2.8695329227 -1.0375328793
H 1.6634877228 -3.4171588749 -1.8401765199
H 1.1485408098 -3.5276806261 -0.1628234115
C -0.5623379979 -2.714069559 -2.9055159065
H 0.168234928 -2.2305668302 -3.5618441333
H -0.5214932178 -3.7916834658 -3.0902181224
H -1.5553558472 -2.3533759213 -3.1859283639
C 5.0996362878 -0.261826959 2.1702131821
C 6.0658429715 -1.4322702671 0.3362671811
C 6.5481041789 -0.5835740129 2.5597627247
H 4.3949912411 -1.0246768589 2.4997045106

H 4.7450966991 0.720905429 2.4755141701
C 7.2203175644 -1.1162307975 1.2733480702
H 6.2979230454 -1.4063744418 -0.7272615389
H 5.5914685128 -2.3755833358 0.5999969053
H 7.0616965164 0.3031833669 2.9325413046
H 6.5520279651 -1.3316985903 3.3534094351
H 7.8618185756 -0.3513778016 0.8287429688
H 7.8276436885 -2.0041104532 1.4505526167
H 5.5554349367 0.529423855 0.3184284999
N 5.0936150665 -0.3228486177 0.6524819713

Energy= -1436.5020983
Zero-point correction= 0.513651 (Hartree/Particle)
Thermal correction to Gibbs Free Energy= 0.455812
Sum of electronic and zero-point Energies= -1435.988447
Sum of electronic and thermal Energies= -1435.960961
Sum of electronic and thermal Enthalpies= -1435.960017
Sum of electronic and thermal Free Energies= -1436.046286
Imaginary Frequencies 1

unstable

C -2.8264115261 -3.2759026912 1.3006697373
C -1.4584925677 -2.9804022867 1.3964735116
C -0.9814683525 -1.7339391767 1.0238581325
C -1.8691923111 -0.7760199545 0.5309445175
C -3.2485186675 -1.0736120345 0.4837259609
C -3.7367725999 -2.3148682215 0.8533712343
H -0.7746416785 -3.7338924098 1.770389609
H 0.0740680167 -1.5213189604 1.1299593769
H -4.7972032585 -2.5387187386 0.8124805039
C -3.0240697812 1.1658438463 -0.1083557909
C -3.4456061401 2.3917411029 -0.6273397336
C -4.7869377028 2.5872648417 -0.9152931458
C -5.7145581254 1.5544779708 -0.7130711705
C -5.3038409278 0.3043570883 -0.242618931
C -3.9619634592 0.1220402168 0.0434877232
H -2.7448055368 3.1939246901 -0.8216564864
H -5.1251553511 3.5397219132 -1.3073007364
H -6.0208660089 -0.5003238965 -0.1208356367
C -1.6716162699 0.645048123 0.174623807
C 0.8590754296 0.8524509133 -0.0715761789
C 1.7735240378 1.7618584514 0.4245424645
C -0.5022637854 1.3555521193 0.165303684
C 1.3224482044 -0.2629451677 -0.8462279346
C 2.7280161472 -0.5247467923 -0.8957490604
C 0.4644271484 -1.0680131598 -1.6336192707
H -0.589662312 -0.8321324383 -1.6680553178
C 0.9469074462 -2.1128987227 -2.376698966
H 0.2683842161 -2.704520978 -2.9820657937
C 2.3220673149 -2.412914651 -2.3686503328
H 2.6998633057 -3.2469728861 -2.9499523436
C 3.1907866949 -1.6310606603 -1.6556256517
H 4.2479148943 -1.8668221119 -1.7182638723
C 3.6041839476 0.3786759419 -0.2225503231
C 3.1544106314 1.521554763 0.3753528186
H 3.8419710542 2.2381074162 0.8103568687
C -0.3744933536 2.838930733 0.5292212978
H -0.5167655652 3.4441119531 -0.3754233962
C 1.1032816945 2.9760403977 0.9756044982
H 1.1625385538 2.9870506986 2.0700328381
H 1.5721139723 3.8976466085 0.6209479774
C -1.3001692382 3.3381118685 1.6383192753
H -1.1933232348 2.7139040104 2.5303472194
H -1.0158618238 4.3592641289 1.9089210102
H -2.3520950396 3.3535164469 1.3602845638
C -7.0968273933 1.7784884389 -1.0157501355
C -3.2969212925 -4.5728663215 1.6859602988

N -8.2137404491 1.9603488674 -1.2595781412
N -3.6758109307 -5.6216956829 1.9966055067
C 5.7436539507 0.1601649158 1.1200066456
C 5.8796885743 1.0117709819 -1.1239518119
C 7.2062144217 0.0767974554 0.7228122071
H 5.3730021215 -0.6623643834 1.7300341212
H 5.4969566101 1.113274817 1.5856775492
C 7.3079587797 0.9182801215 -0.5664689491
H 5.4623414312 2.0131221432 -1.0371813507
H 5.7584276778 0.6550622892 -2.1455680799
H 7.849717371 0.4485437684 1.5207487782
H 7.4772572395 -0.9639596179 0.5270247128
H 7.6770745536 1.9219905341 -0.3512267726
H 7.9854154539 0.4589639861 -1.2867627881
N 5.048995551 0.1142508367 -0.2216883043
H 5.1936435674 -0.8416509456 -0.5563012134

Energy= -1436.5347403
Zero-point correction= 0.513502 (Hartree/Particle)
Thermal correction to Gibbs Free Energy= 0.453319
Sum of electronic and zero-point Energies= -1436.021238
Sum of electronic and thermal Energies= -1435.992814
Sum of electronic and thermal Enthalpies= -1435.991870
Sum of electronic and thermal Free Energies= -1436.081421
Imaginary Frequencies 0

1-H-wb97xd-mecn
stable

C 2.731674312 3.5673311313 -0.3199174844
C 1.3719878748 3.2404787291 -0.4645390032
C 0.9543802673 1.9157429402 -0.4026788008
C 1.8937415821 0.9045677726 -0.1734418188
C 3.262748995 1.2448909375 -0.0926108031
C 3.6945285898 2.5631299562 -0.1494820852
H 0.641245137 4.0331647291 -0.6327399065
H -0.1001292126 1.6930397636 -0.5519069658
H 4.7545662705 2.8142204011 -0.0801483264
C 3.1524383521 -1.088539958 -0.0861796186
C 3.6706815482 -2.3873421259 -0.0247530335
C 5.0457710402 -2.5756518994 0.0732746945
C 5.9159736884 -1.4735912536 0.1273029234
C 5.4153848737 -0.164907803 0.0871488344
C 4.0427138312 0.0088396272 -0.0189388691
H 3.0303333903 -3.2663762859 -0.0433096989
H 5.4527228687 -3.5873907925 0.1129885267
H 6.0905158669 0.6911629332 0.1395101018
C 1.761377964 -0.572248301 -0.1317108918
C -0.7792416978 -0.8776061382 -0.0124750209
C -1.5737827105 -1.6635762361 -0.8275952449
C 0.6199071222 -1.317056776 -0.1769379306
C -1.3712474232 0.063648818 0.8844732346
C -2.7736817274 0.3198552399 0.7686956917
C -0.6404115367 0.7101599575 1.9159350595
H 0.413625654 0.4658256372 2.0503445092
C -1.2440359765 1.6208220633 2.7480662197
H -0.6671041625 2.1034030108 3.5395853463
C -2.6120082511 1.9338492725 2.5841915242
H -3.0835074936 2.6740810555 3.2335619284
C -3.3598719878 1.2946035255 1.6252029405
H -4.4174611449 1.5540255415 1.5424928061
C -3.5263403881 -0.4447398197 -0.1790542976
C -2.9580502923 -1.4361832269 -0.9377305543
H -3.5486314767 -2.0451930544 -1.6213424911
C 0.5296774896 -2.7666262333 -0.6317564077
H 1.3933819213 -3.0365160484 -1.2517501458
C -0.7632766268 -2.7419238754 -1.4863185158

H -1.287465849 -3.7085752934 -1.5006769942
H -0.531187532 -2.4713800844 -2.5295460852
C 0.4032887592 -3.7461075439 0.5401803128
H -0.5080764321 -3.544048685 1.1246037336
H 0.3476362911 -4.7784325641 0.1640425307
H 1.262258489 -3.6740037692 1.2231926824
C 7.3326984243 -1.6898157493 0.2307432203
C 3.1406958208 4.9434852294 -0.3770445505
N 8.4743764989 -1.8652438704 0.3132290171
N 3.4682988164 6.0533344337 -0.4217893302
C -5.8945005002 -1.2539115991 -0.6792982878
C -5.2576509086 0.9418139009 -1.337212192
C -7.1936167688 -0.5035852417 -0.9158975143
H -5.9055699713 -1.9861565464 0.1361317416
H -5.5332811917 -1.7215257547 -1.6024213207
C -6.7623809227 0.8087295264 -1.6043630788
H -4.6486663551 0.7120200373 -2.2197080527
H -4.940834988 1.9035941865 -0.9178827127
H -7.8844201384 -1.0994746022 -1.5249082642
H -7.6849037854 -0.2961325873 0.0461470901
H -6.9387123631 0.7708223439 -2.6870561519
H -7.3153735519 1.6680810166 -1.2048446014
N -4.9516551986 -0.1401536777 -0.3261174324
H -5.2831290535 0.2033646193 0.5847064042

Energy= -1435.818118
Zero-point correction= 0.512474 (Hartree/Particle)
Thermal correction to Gibbs Free Energy= 0.453443
Sum of electronic and zero-point Energies= -1435.305644
Sum of electronic and thermal Energies= -1435.277404
Sum of electronic and thermal Enthalpies= -1435.276460
Sum of electronic and thermal Free Energies= -1435.364675
Imaginary Frequencies 0

TS-EZ

C -3.9729222633 3.1577450547 0.0188621607
C -2.7005562298 3.4797069004 0.5707042757
C -1.7928503159 2.4898816143 0.8803207968
C -2.1357273009 1.1342337071 0.6477528186
C -3.4191163469 0.8155865414 0.0770234819
C -4.3276913913 1.825116159 -0.2278662318
H -2.4488654978 4.527790029 0.7467973378
H -0.8193100538 2.752474284 1.3017732255
H -5.3038437582 1.5875808822 -0.6577872072
C -2.2287521013 -1.1488100761 0.413599056
C -1.9977142842 -2.5466274447 0.3606956956
C -2.9775271353 -3.3759004253 -0.1407016308
C -4.2138045253 -2.8466567766 -0.6085423166
C -4.4589058428 -1.4677932865 -0.5737677603
C -3.4768411544 -0.6184697593 -0.0716535317
H -1.0526427007 -2.9659365765 0.7141283341
H -2.8116995291 -4.4545343423 -0.184769519
H -5.4076645169 -1.0694842832 -0.9414625574
C -1.4175020383 -0.0716829886 0.8539876482
C 1.1380595163 -0.1354634646 0.6382607341
C 2.2140059917 -0.2798985707 1.541001908
C -0.060287636 -0.1729831734 1.3715157642
C 1.3745675467 0.0265360789 -0.7815046857
C 2.7316369252 0.03006174 -1.2283313916
C 0.3443030315 0.1836694379 -1.729258167
H -0.6915526165 0.1788386961 -1.3963589622
C 0.638170753 0.344728421 -3.0690689187
H -0.1745244689 0.4651476671 -3.7880572184
C 1.9714031568 0.3577669701 -3.5122387815
H 2.1963755583 0.4916788985 -4.5716388516
C 2.9999333665 0.202908119 -2.6091755146
H 4.0205522244 0.229367555 -2.9940926062

C 3.7730037329 -0.1354571215 -0.2514782176
C 3.5415732198 -0.2666270556 1.0955146521
H 4.3604942781 -0.3646902261 1.8091398277
C 0.2221908558 -0.2921015971 2.8319571649
H -0.0756559657 0.7033622495 3.2241996656
C 1.7392930659 -0.4493466839 2.9352103856
H 2.0209297586 -1.4517332283 3.3003006375
H 2.2193553325 0.2739131195 3.6123935614
C -0.6129201585 -1.3434526864 3.5633463176
H -0.3855808725 -2.351746956 3.1872477683
H -0.3801226019 -1.3121280034 4.6368230214
H -1.6870090675 -1.1516196566 3.4353975102
C -5.2114653901 -3.7319320886 -1.1245481076
C -4.8947093991 4.2065529445 -0.2904102892
N -6.0190192996 -4.4528722626 -1.5434170848
N -5.6401006196 5.060460373 -0.5405154927
C 6.0868376942 -1.18842962 -0.1111327857
C 5.8386464863 1.2333840473 -0.4796258854
C 7.1510437241 -0.4060890888 0.6388721478
H 6.5027910747 -1.7264173334 -0.9710940376
H 5.4981021486 -1.8850559774 0.4934978591
C 7.2818612495 0.8767125347 -0.1775610236
H 5.3468423181 1.6958733428 0.3844457752
H 5.67071089 1.8463389093 -1.3722926736
H 6.8116008945 -0.1790595899 1.6611433906
H 8.0846990142 -0.9791725395 0.7030474761
H 7.7776449911 1.6912693663 0.3659220573
H 7.8371071358 0.6912717885 -1.1095308848
N 5.1617649616 -0.11968092 -0.7027537033
H 5.1703081857 -0.2889526283 -1.7125894173

Energy= -1435.7663326
Zero-point correction= 0.508935 (Hartree/Particle)
Thermal correction to Gibbs Free Energy= 0.449595
Sum of electronic and zero-point Energies= -1435.257398
Sum of electronic and thermal Energies= -1435.229437
Sum of electronic and thermal Enthalpies= -1435.228493
Sum of electronic and thermal Free Energies= -1435.316738
Imaginary Frequencies 1

TS-THI

C -3.256516875 3.3982833072 0.0709875416
C -2.4266195285 3.3874877215 -1.0606866636
C -1.8333680535 2.2012263426 -1.4848351276
C -2.014494179 1.0268575822 -0.7515961195
C -3.0062181536 1.0264362568 0.2559588928
C -3.5934904871 2.1970285657 0.7128557051
H -2.2649081572 4.3077294496 -1.6242740061
H -1.2910845408 2.2032813583 -2.4279919304
H -4.3386871249 2.1824395397 1.5104886655
C -2.6982006543 -1.1893917588 -0.423281778
C -3.1344818782 -2.5089477522 -0.5916399392
C -4.172359729 -3.0072023824 0.1952254114
C -4.7992805329 -2.1964881585 1.1508665265
C -4.4351013085 -0.8474933459 1.2812414087
C -3.4170271582 -0.3613215044 0.4775673588
H -2.7289807706 -3.1611774093 -1.361112735
H -4.5082513392 -4.0357519004 0.0535172653
H -4.9603403036 -0.193566225 1.9798230057
C -1.5616992103 -0.3760519553 -0.9819316679
C 1.0980375821 -0.4786162656 -1.0019726347
C 1.8734769302 -1.6133769294 -0.7991877862
C -0.3213186507 -0.9347840308 -1.2092295694
C 1.7517760396 0.7985552794 -0.8220348855
C 3.0590174886 0.8555464592 -0.2321849334
C 1.202683269 2.0188787523 -1.2674613771
H 0.2940229463 1.973766571 -1.8433138096

C -3.8366387529 4.634448888 0.517618033
C -5.851398128 -2.7360145165 1.9666954695
N -4.2993978079 5.632174402 0.8799506307
N -6.6973186093 -3.1724308418 2.626220091
C 1.8070278931 3.2343223141 -1.0503915242
H 1.3379455538 4.1481162353 -1.4215856708
C 3.0348365345 3.2918721639 -0.3650599323
H 3.5148907359 4.2519934623 -0.1662900502
C 3.6519881829 2.1239462428 0.0160374052
H 4.6244173568 2.2008820081 0.5063750102
C 3.7430665122 -0.371387993 0.0378208392
C 3.1863017677 -1.5726954283 -0.2852012716
H 3.7062019748 -2.5158090348 -0.1197492664
C -0.2406445204 -2.4366897343 -1.4869401316
H -0.9401521494 -2.9683275917 -0.8348277229
C 1.1707587166 -2.8851776475 -1.1140873897
H 1.6781730536 -3.4117260091 -1.9385251806
H 1.1803485992 -3.5661901418 -0.2492227054
C -0.5992151627 -2.7336260236 -2.9458875096
H 0.1151084687 -2.2453526432 -3.6278575669
H -0.5647230753 -3.8179398556 -3.1324826891
H -1.6072966579 -2.3706805679 -3.1965732873
C 5.0281186996 -0.3024870682 2.1860097037
C 6.0637685483 -1.3848494222 0.3452873793
C 6.4770856276 -0.5819351348 2.5996084227
H 4.3408288428 -1.1058898423 2.4777663999
H 4.6243875122 0.661268142 2.5161851534
C 7.1887407233 -1.0783276175 1.3207086465
H 6.3209199168 -1.3183982462 -0.7180225944
H 5.6052245173 -2.3557848427 0.5633095052
H 6.9574787177 0.3229246023 2.9927774359
H 6.4911513502 -1.3398782517 3.3932036654
H 7.8384513799 -0.2931993749 0.9077575761
H 7.8055924806 -1.9687651979 1.4929982754
H 5.506620271 0.5611671527 0.3803700865
N 5.0600719362 -0.3172638537 0.6748194763

Energy= -1435.7786019
Zero-point correction= 0.512109 (Hartree/Particle)
Thermal correction to Gibbs Free Energy= 0.453674
Sum of electronic and zero-point Energies= -1435.266493
Sum of electronic and thermal Energies= -1435.238966
Sum of electronic and thermal Enthalpies= -1435.238022
Sum of electronic and thermal Free Energies= -1435.324928
Imaginary Frequencies 1

unstable

C -2.70032122 3.3786722874 -0.9362455632
C -1.3376592773 3.0918911668 -1.1416403034
C -0.8465518659 1.8110518008 -0.9226340639
C -1.7132079848 0.8078602745 -0.4729292531
C -3.0892041948 1.0976557442 -0.3209737886
C -3.5923507118 2.3738599234 -0.537152318
H -0.6656067531 3.8823227255 -1.4798862403
H 0.2070703565 1.6079536671 -1.1121733759
H -4.6548997621 2.5908354858 -0.4123410127
C -2.8351592313 -1.1917398757 0.0432019779
C -3.2327723257 -2.4690684754 0.4571322703
C -4.562643642 -2.6986436321 0.7876361272
C -5.5031066854 -1.6529091172 0.7312686871
C -5.1132905598 -0.3569190325 0.3663916897
C -3.7818637496 -0.1402415574 0.0364894768
H -2.520162185 -3.2899174742 0.5372623681
H -4.8818585726 -3.6944182025 1.0998324411
H -5.8378664329 0.45958116 0.3555836818
C -1.4986371024 -0.6370526384 -0.2516861716
C 1.0381269832 -0.8250122175 -0.0936390079

C 1.9555379854 -1.6838928563 -0.6757061926
C -0.3193714448 -1.3336953537 -0.3335232326
C 1.5001862889 0.2532650392 0.7355766532
C 2.8996410329 0.5558793448 0.7409731564
C 0.6510996593 0.9840701111 1.6067997006
H -0.3988928131 0.706678954 1.6812673742
C 1.1334401085 2.0142692304 2.3779152277
H 0.4602210459 2.553685355 3.04728281
C 2.4976830673 2.369155219 2.3159959179
H 2.8755836037 3.1986294661 2.9167795534
C 3.3615410839 1.6474585254 1.5283687567
H 4.4176989532 1.9232161437 1.5478795349
C 3.7796482984 -0.2910587493 -0.0055458148
C 3.3363257071 -1.4154240545 -0.6510529232
H 4.0267511686 -2.1008363762 -1.1438357145
C -0.1858891809 -2.776540756 -0.8232493468
H -0.3307782541 -3.4592244496 0.0333973303
C 1.291154512 -2.8761124728 -1.279663022
H 1.3530594518 -2.8342717308 -2.3798663286
H 1.7784216308 -3.8117069133 -0.9685585863
C -1.1251989227 -3.1686667896 -1.9632017748
H -1.0140578411 -2.468211243 -2.8055368253
H -0.8621850402 -4.1752146941 -2.3222913967
H -2.1826108286 -3.1876017642 -1.6760602613
C -6.8737875153 -1.9129499441 1.0738719989
C -3.1858891479 4.7119099819 -1.1593089522
N -7.9786900864 -2.1244862548 1.3492842035
N -3.5753662466 5.787984282 -1.3378444631
C 5.9686236155 -0.1408994796 -1.2866490346
C 6.0206432858 -0.7795469086 1.0145865798
C 7.4049046249 0.0521802331 -0.8343900932
H 5.5897539662 0.5965364813 -2.0035261375
H 5.7968397161 -1.1555023803 -1.6644379552
C 7.4744759394 -0.6372089901 0.544375759
H 5.6588798993 -1.8128340033 0.9607462958
H 5.8077683606 -0.3717323897 2.0094030491
H 8.1064275803 -0.3748338521 -1.5615844959
H 7.6246985745 1.126210252 -0.7439653487
H 7.9319959308 -1.6321903285 0.4701824654
H 8.0677841184 -0.0477812243 1.2545289947
N 5.2135346953 0.0057250646 0.0054360434
H 5.3205543341 0.9960212621 0.2555688737

Energy= -1435.8123889
Zero-point correction= 0.511992 (Hartree/Particle)
Thermal correction to Gibbs Free Energy= 0.452667
Sum of electronic and zero-point Energies= -1435.300397
Sum of electronic and thermal Energies= -1435.272080
Sum of electronic and thermal Enthalpies= -1435.271136
Sum of electronic and thermal Free Energies= -1435.359722
Imaginary Frequencies 0

Envelope-1-CAM-B3LYP

C 2.7588794905 3.445231289 -0.725759636
C 1.3863876955 3.1870411157 -0.8299751019
C 0.8875940004 1.908910279 -0.6292942141
C 1.7601489741 0.8709993122 -0.3028026798
C 3.1450147503 1.1334736928 -0.2568944043
C 3.6511963372 2.4068165398 -0.4511097494
H 0.7119433813 3.9993682365 -1.0750791249
H -0.172929431 1.7357353206 -0.7394133869
H 4.7166688691 2.6046374812 -0.4109704278
C 2.8939760173 -1.1697127823 -0.0117670622
C 3.3367465157 -2.4767208665 0.196315523
C 4.6936207907 -2.7340653728 0.3263966402
C 5.6281934491 -1.6937544564 0.2693211068
C 5.2022372025 -0.3766704356 0.0845415052

C 3.8477484739 -0.1297979456 -0.0514518613
H 2.647743407 -3.3055036435 0.2694451857
H 5.0405268102 -3.7494127613 0.4800920061
H 5.9289334831 0.4276321109 0.0529241716
C 1.5378049635 -0.582698126 -0.1280246933
C -1.0227077686 -0.7708172599 -0.069770901
C -1.8312978162 -1.5867810837 -0.839609955
C 0.3589149585 -1.2649092934 -0.1572356706
C -1.6173021641 0.2556885735 0.7279589112
C -3.0111353213 0.507930021 0.5750447662
C -0.8981030709 1.0056009757 1.6903681912
H 0.1431414988 0.7664219965 1.8654635337
C -1.4970791568 2.0188142904 2.3918849207
H -0.9265689799 2.5785308949 3.1256457901
C -2.8421647666 2.3543999602 2.1424818717
H -3.2933991906 3.1989554525 2.652398225
C -3.5787616824 1.6116743269 1.2578394515
H -4.6072746756 1.874571802 1.0450684868
C -3.8125836577 -0.3432260844 -0.2660614187
C -3.2088898372 -1.3701347935 -0.9638111645
H -3.8004260596 -2.040342657 -1.5758420782
C 0.2308251278 -2.739601835 -0.5247136791
H 1.0992500805 -3.0771987585 -1.0921679124
C -1.0345485821 -2.7235003414 -1.4192346841
H -1.5793093289 -3.6720973783 -1.3984597761
H -0.7620350968 -2.5235332317 -2.4620791909
C 0.0156130004 -3.6432573292 0.6961445128
H -0.9021757688 -3.363728471 1.2201671699
H -0.0745501052 -4.6881826911 0.3860854634
H 0.8375990042 -3.5694500425 1.4117688875
C 7.0262341712 -1.9830927385 0.4101558466
C 3.2505123337 4.777997351 -0.9266115536
N 8.1533028518 -2.2183731405 0.5237136181
N 3.6454807698 5.8535863551 -1.0871269774
C -5.9506205069 -0.6553105755 -1.4609322987
C -6.0183903621 -0.4100376891 0.8414642018
C -7.377667017 -0.1816848823 -1.1724326173
H -5.9226371133 -1.7583846803 -1.4772004259
H -5.5472969664 -0.2931627044 -2.4094646967
C -7.4463208622 -0.0980165066 0.3720706888
H -5.7078388308 0.1718283999 1.7084774727
H -5.9216085415 -1.4758638382 1.1050644795
H -7.541823318 0.802902824 -1.6149987376
H -8.1210135612 -0.8613501385 -1.5938428624
H -7.7500594502 0.9012426718 0.6912084378
H -8.1607346184 -0.8059362331 0.7971659043
N -5.1949629798 -0.1097508557 -0.340073718

Energy= -1436.0424571
Zero-point correction= 0.499262 (Hartree/Particle)
Thermal correction to Gibbs Free Energy= 0.440052
Sum of electronic and zero-point Energies= -1435.543195
Sum of electronic and thermal Energies= -1435.514958
Sum of electronic and thermal Enthalpies= -1435.514013
Sum of electronic and thermal Free Energies= -1435.602405
Imaginary Frequencies 0

Envelope-1-CAM-B3LYP-MeCN

C 2.7604710071 3.445977406 -0.7167172357
C 1.3867030412 3.1892797433 -0.8327849899
C 0.890016819 1.9106346273 -0.6346726382
C 1.7630432253 0.8722900672 -0.3013879637
C 3.1490073971 1.1384236067 -0.247275149
C 3.6570806286 2.4118456052 -0.4362493761
H 0.7117578282 3.9994029974 -1.0847796938
H -0.1692819738 1.7378458799 -0.7575008325
H 4.7228216848 2.6067525472 -0.3860314711

C 2.8968341106 -1.1644077126 -0.0045285879
C 3.3391647819 -2.4743095161 0.1998399822
C 4.6950727506 -2.7307937771 0.3328241172
C 5.6275122768 -1.6854580946 0.2809386649
C 5.2069605834 -0.3655507111 0.0986639908
C 3.8516761111 -0.122847476 -0.0394139217
H 2.65249117 -3.3052157228 0.2676729386
H 5.0401723061 -3.7474823958 0.4832186103
H 5.9297432829 0.4427388992 0.0688061658
C 1.5428729261 -0.5789863989 -0.1255873526
C -1.0173146828 -0.769226062 -0.0668592061
C -1.8284349217 -1.5856922584 -0.8376016223
C 0.3605712986 -1.264295298 -0.1606315462
C -1.6130760197 0.2578196389 0.731978872
C -3.0090274331 0.5075733313 0.5826687141
C -0.8939172648 1.0083856646 1.6947583399
H 0.1466507128 0.7685203033 1.874500332
C -1.4921616829 2.0218558224 2.3990571124
H -0.9213072166 2.5800231864 3.1343581111
C -2.837696266 2.3568567909 2.1514254673
H -3.2907134298 3.199660985 2.6632067555
C -3.5752047078 1.6115531218 1.2675312376
H -4.6059047118 1.8757627241 1.0673574675
C -3.8129104376 -0.3443693308 -0.2617871593
C -3.2052102741 -1.3682597954 -0.9641692644
H -3.7931359217 -2.0388924539 -1.5787684485
C 0.2352937071 -2.7368936316 -0.5342120947
H 1.1016341774 -3.0707269294 -1.1061294498
C -1.036427763 -2.7227161718 -1.4184133652
H -1.5832308126 -3.6691998043 -1.3903740158
H -0.7726169347 -2.5229215613 -2.4633039059
C 0.0313559287 -3.6470486785 0.6836344744
H -0.8914334217 -3.3865011696 1.2097842679
H -0.0436723198 -4.6898389135 0.3625627186
H 0.8538869174 -3.5691001618 1.3985684035
C 7.0230852348 -1.9745695423 0.4238797977
C 3.2510216523 4.7769696307 -0.9140922241
N 8.1509280395 -2.2090443093 0.5391879301
N 3.6468918715 5.8532873971 -1.072994783
C -5.9443860603 -0.6997834718 -1.4517077937
C -6.0262549177 -0.3777033725 0.8427238975
C -7.3725641281 -0.2192530126 -1.1856297765
H -5.9104918769 -1.800445967 -1.4244551579
H -5.5383285018 -0.3706550124 -2.411100836
C -7.4489898321 -0.0795136629 0.3536967314
H -5.7270453203 0.2279597096 1.6965778314
H -5.9302546843 -1.4361442453 1.130095789
H -7.5397451936 0.7479231547 -1.6655445433
H -8.1094156075 -0.919246034 -1.5847926269
H -7.7502157384 0.9320157046 0.6358292106
H -8.1652217301 -0.7721390652 0.8005664198
N -5.1912493739 -0.1123055944 -0.3444983396

Energy= -1436.0813484
Zero-point correction= 0.498525 (Hartree/Particle)
Thermal correction to Gibbs Free Energy= 0.439350
Sum of electronic and zero-point Energies= -1435.582823
Sum of electronic and thermal Energies= -1435.554593
Sum of electronic and thermal Enthalpies= -1435.553649
Sum of electronic and thermal Free Energies= -1435.641999
Imaginary Frequencies 0

Envelope-1-wB97X-D

C 2.6742322 3.45918896 -0.72758044
C 1.30826591 3.16227031 -0.87188945
C 0.83897227 1.86647034 -0.69067658
C 1.73392331 0.85004841 -0.34107225

C 3.1126537 1.14821192 -0.26107487
C 3.59034119 2.44015976 -0.43630533
H 0.61229553 3.96055571 -1.13387348
H -0.21980434 1.66254949 -0.83601176
H 4.65600853 2.6657423 -0.36764197
C 2.91040853 -1.16666262 -0.03302319
C 3.38012407 -2.46949444 0.16854674
C 4.74379632 -2.69748987 0.32116838
C 5.65865796 -1.63244107 0.29127626
C 5.20402724 -0.31982696 0.1096618
C 3.84245925 -0.1020386 -0.04917142
H 2.70496905 -3.32084054 0.21918329
H 5.11206676 -3.71354686 0.47090734
H 5.91511164 0.50809034 0.09673634
C 1.54395914 -0.60760511 -0.16443297
C -1.0097797 -0.82107336 -0.07609774
C -1.82561009 -1.64827462 -0.83194553
C 0.37184389 -1.30831167 -0.18135746
C -1.59232875 0.21903389 0.71493718
C -2.98653503 0.481824 0.56240416
C -0.85766333 0.98306779 1.65933816
H 0.18748725 0.73214227 1.8418388
C -1.43978453 2.03178268 2.33032317
H -0.85473945 2.60824674 3.04975917
C -2.78166374 2.38439344 2.06778078
H -3.2197162 3.26111517 2.54898005
C -3.5359301 1.62077375 1.20894767
H -4.56763254 1.89703854 0.99027575
C -3.80351829 -0.38740845 -0.25286671
C -3.20744241 -1.43176721 -0.94337937
H -3.81119386 -2.11684113 -1.53926203
C 0.24764226 -2.78682904 -0.52523813
H 1.11549222 -3.12929004 -1.10442262
C -1.02870662 -2.78939742 -1.40357101
H -1.57339028 -3.74520058 -1.36719749
H -0.76915574 -2.59563972 -2.45778521
C 0.06363853 -3.66601563 0.7175181
H -0.85443741 -3.38485748 1.25590952
H -0.01638187 -4.72553174 0.43156407
H 0.9029799 -3.56070552 1.41984821
C 7.06406129 -1.89110253 0.4550551
C 3.13559594 4.8098296 -0.90639223
N 8.19456799 -2.10189543 0.58692716
N 3.50434079 5.89789513 -1.04835629
C -5.9629661 -0.71406034 -1.40127467
C -5.99697193 -0.37076048 0.88682988
C -7.3770546 -0.19715598 -1.11318221
H -5.96060369 -1.82507418 -1.38783034
H -5.55978378 -0.39017541 -2.37181602
C -7.40921688 0.0010504 0.42113137
H -5.64495745 0.22215926 1.73977285
H -5.95256395 -1.43799364 1.18853675
H -7.54361436 0.75854536 -1.62918062
H -8.14311392 -0.89972138 -1.46885094
H -7.63428973 1.04712122 0.67265547
H -8.16799641 -0.6223648 0.91382284
N -5.18028325 -0.16140864 -0.31031961

Energy= -1435.3224738
Zero-point correction= 0.497719 (Hartree/Particle)
Thermal correction to Gibbs Free Energy= 0.438496
Sum of electronic and zero-point Energies= -1434.824755
Sum of electronic and thermal Energies= -1434.796528
Sum of electronic and thermal Enthalpies= -1434.795584
Sum of electronic and thermal Free Energies= -1434.883978
Imaginary Frequencies 0

Envelope-1-wB97X-D-MeCN

C 2.67391174 3.45291592 -0.73687331
C 1.30626668 3.15795621 -0.88808716
C 0.83861049 1.86274537 -0.70347261
C 1.73419883 0.84631182 -0.34633358
C 3.11423856 1.14755796 -0.26271048
C 3.59418764 2.43878351 -0.43849915
H 0.60978336 3.95369572 -1.15766608
H -0.21939721 1.65968647 -0.85662055
H 4.66042702 2.66048308 -0.36311373
C 2.90954015 -1.16595422 -0.02773022
C 3.37851849 -2.47134071 0.17394732
C 4.7413329 -2.69866488 0.32757223
C 5.65458721 -1.62942382 0.29710105
C 5.2059445 -0.31431748 0.11398542
C 3.84342255 -0.10034942 -0.04514646
H 2.70556779 -3.32449953 0.22365021
H 5.10705027 -3.71595639 0.47712951
H 5.91313167 0.5173059 0.09838474
C 1.54566353 -0.60796877 -0.16378814
C -1.00761341 -0.82141294 -0.07182824
C -1.8284112 -1.65037448 -0.82546299
C 0.36905129 -1.31109056 -0.18461279
C -1.58918087 0.22265247 0.71746105
C -2.98542699 0.4844822 0.56986006
C -0.8520743 0.98987441 1.65818829
H 0.19222234 0.7371003 1.84492871
C -1.43078392 2.04296329 2.32805733
H -0.84377808 2.61986547 3.04640766
C -2.7723063 2.39705887 2.06633947
H -3.20987202 3.27565561 2.54543445
C -3.53010985 1.62839986 1.2127573
H -4.56308054 1.90888666 1.00557589
C -3.80771663 -0.38820818 -0.24400006
C -3.20904131 -1.43225581 -0.93797366
H -3.81062697 -2.11987159 -1.53282294
C 0.24625286 -2.78787558 -0.532068
H 1.11080359 -3.12866535 -1.11621974
C -1.03837012 -2.79387309 -1.39658187
H -1.58572731 -3.74692466 -1.34935765
H -0.79035855 -2.6037552 -2.45403788
C 0.07564534 -3.67116884 0.70963315
H -0.84775951 -3.41020068 1.25007372
H 0.0127301 -4.72927985 0.41453019
H 0.91510761 -3.55915395 1.41104405
C 7.05779625 -1.8879688 0.46136318
C 3.13460387 4.80089303 -0.91841695
N 8.18916642 -2.09811397 0.59372782
N 3.50492516 5.88876243 -1.06408586
C -5.96295504 -0.76501176 -1.37904316
C -6.00784345 -0.31924393 0.89415648
C -7.37590354 -0.23297806 -1.11874006
H -5.95840644 -1.87191395 -1.31248191
H -5.5566628 -0.4846557 -2.36173934
C -7.41343231 0.03862642 0.40333933
H -5.6646937 0.30668517 1.72592187
H -5.96932231 -1.37423573 1.2323824
H -7.54178049 0.6970664 -1.68103794
H -8.13856315 -0.95484017 -1.4418295
H -7.63060803 1.09764862 0.60420731
H -8.17609177 -0.55838545 0.92227342
N -5.17919399 -0.16524581 -0.30812388

Energy= -1435.3613922
Zero-point correction= 0.496830 (Hartree/Particle)
Thermal correction to Gibbs Free Energy= 0.437495
Sum of electronic and zero-point Energies= -1434.864562
Sum of electronic and thermal Energies= -1434.836312
Sum of electronic and thermal Enthalpies= -1434.835368

Sum of electronic and thermal Free Energies= -1434.923898
Imaginary Frequencies 0

Envelope-1-TS-CAM-B3LYP

C 3.3645829749 3.363526391 -0.1777407371
C 2.4277064061 3.3876728701 0.8585544645
C 1.7962342684 2.2195257061 1.2658573464
C 2.0457591011 1.0160507826 0.6117704726
C 3.1349244578 0.98674739 -0.2865156542
C 3.7592362837 2.139842322 -0.7252688886
H 2.2159912128 4.3231236098 1.3632520488
H 1.1725496038 2.2587145009 2.1469650461
H 4.5783366241 2.1034656581 -1.4348160808
C 2.7773272378 -1.1970790685 0.4422807257
C 3.2236552246 -2.4885706929 0.7285311701
C 4.3529661095 -2.9969791519 0.0963761301
C 5.066449417 -2.2271854465 -0.823740316
C 4.6896826187 -0.9016345835 -1.0589622636
C 3.5811384844 -0.3993997769 -0.4054779919
H 2.7486462849 -3.102319055 1.4802807668
H 4.6948614489 -3.9987806869 0.3295785285
H 5.2814989244 -0.2802068282 -1.7218867948
C 1.5752071995 -0.3837794221 0.8426348176
C -1.093054102 -0.5265390583 0.668717181
C -1.8243925455 -1.661565412 0.346512669
C 0.3234748479 -0.9510033369 0.938687834
C -1.7942152511 0.7318849283 0.5480518217
C -3.1022056641 0.764360241 -0.0250409967
C -1.2833688568 1.9603487058 1.0047111178
H -0.3698964669 1.9392827614 1.5583862954
C 3.9874482554 4.5813491469 -0.6097547771
C 6.2134409668 -2.7791562233 -1.4848953531
N 4.4859532795 5.564992402 -0.9594771774
N 7.1363568997 -3.2267095636 -2.0200305879
C -1.9164556447 3.1577059855 0.7927813972
H -1.4719398859 4.075532664 1.1640917165
C -3.1245372045 3.1937846945 0.0792505435
H -3.5992335572 4.1410800285 -0.1529975273
C -3.7045529281 2.0136163846 -0.3050319058
H -4.6339635216 2.0145575694 -0.8602511552
C -3.8001646626 -0.4556105254 -0.3272822138
C -3.1413122139 -1.6429402791 -0.1364617887
H -3.6289141577 -2.5882690597 -0.3427552425
C 0.2472229636 -2.4653398276 1.1657720803
H 1.0604598866 -2.9647575587 0.6498052533
C -1.0693877166 -2.9290396416 0.5503245634
H -1.6152810857 -3.624294903 1.19721377
H -0.9112566497 -3.4443619554 -0.4039732923
C 0.3330000502 -2.7813447117 2.6635487315
H -0.5096899792 -2.3329819653 3.1983877144
H 0.3021901008 -3.8622469306 2.8309506727
H 1.251785535 -2.3882009252 3.1059981347
C -5.6828405504 -1.5574704981 -1.4756065317
C -7.1001372592 -1.0961748167 -1.8266703749
H -5.7321544648 -2.4322967536 -0.8047578442
H -5.0793982321 -1.8316222441 -2.3440820169
C -7.454737112 -0.0745188912 -0.7186567281
H -7.1053640391 -0.6134605584 -2.8060478825
H -7.7976825463 -1.9351120758 -1.868643408
H -7.7281992775 0.8883811013 -1.1554030135
H -8.2940787721 -0.4016095931 -0.101555885
N -5.1173060349 -0.3932140274 -0.8051243137
C -6.1737327123 0.0512739079 0.1172144928
H -5.9904453831 1.0602820184 0.4845404391
H -6.2145301701 -0.6184766521 0.9915831781

Energy= -1436.0023129

Zero-point correction= 0.498940 (Hartree/Particle)
Thermal correction to Gibbs Free Energy= 0.440587
Sum of electronic and zero-point Energies= -1435.503373
Sum of electronic and thermal Energies= -1435.475832
Sum of electronic and thermal Enthalpies= -1435.474888
Sum of electronic and thermal Free Energies= -1435.561726
Imaginary Frequencies 1

Envelope-1-TS-CAM-B3LYP-MeCN

C 3.3456409464 3.3635961471 -0.1386528447
C 2.4128899076 3.3790957293 0.904061378
C 1.7879945119 2.2050823832 1.3028344745
C 2.0402723738 1.008734035 0.6329167019
C 3.1229211648 0.9922201438 -0.2749980707
C 3.7449808573 2.1497061372 -0.7058653031
H 2.1983913152 4.3080660048 1.4199684129
H 1.1685112302 2.2340722432 2.1869842469
H 4.5591928852 2.1181933122 -1.4217853739
C 2.7645970938 -1.2034842568 0.4196547741
C 3.2063354089 -2.50623754 0.6699920063
C 4.3215418327 -3.0068707795 0.0085315424
C 5.0258256534 -2.2157366559 -0.9032606035
C 4.6593030803 -0.8812975225 -1.1051925547
C 3.5626396233 -0.3916138613 -0.4230121848
H 2.7431428779 -3.1379153291 1.4136143398
H 4.657191408 -4.017237694 0.2131897441
H 5.2414583489 -0.2430146073 -1.7609767991
C 1.5750987536 -0.3923562506 0.8548462954
C -1.0923515946 -0.5273045137 0.713487444
C -1.8326985454 -1.6636248887 0.4081853323
C 0.3212982189 -0.9588203702 0.9757751807
C -1.7870499822 0.7345412231 0.570557108
C -3.0868768728 0.7676303402 -0.0248110903
C -1.2828257537 1.9647334098 1.0346583383
H -0.3911105194 1.9421835669 1.6245196522
C 3.9628072761 4.5856712206 -0.559526122
C 6.1586761141 -2.7579929861 -1.5914377661
N 4.4580037773 5.5741297339 -0.9026442203
N 7.0725826011 -3.1974516639 -2.1498492728
C -1.903771775 3.1651494878 0.7969314642
H -1.4660730502 4.082497892 1.1782376008
C -3.0926876695 3.2013605204 0.0507653042
H -3.5582278838 4.1486796633 -0.2007892331
C -3.6730373264 2.0192298821 -0.3322026227
H -4.5954271379 2.0278680016 -0.8989983752
C -3.7917916649 -0.4546700608 -0.3182070343
C -3.143532267 -1.6436319359 -0.0917904511
H -3.6336443094 -2.5901053736 -0.2856014964
C 0.2434900147 -2.4704632233 1.2150300347
H 1.033241482 -2.9789876275 0.6724777442
C -1.0953846121 -2.9329623723 0.6498285794
H -1.6421392203 -3.5838600682 1.3402027983
H -0.9725030434 -3.4941879922 -0.2829324208
C 0.3854368975 -2.7802745024 2.7092487942
H -0.4307577351 -2.3222766063 3.277079153
H 0.3486811903 -3.8609878155 2.8763717075
H 1.3282360594 -2.4016040711 3.1129392392
C -5.6605636627 -1.5939954448 -1.4468855399
C -7.0645166175 -1.139940919 -1.8525084966
H -5.7300365566 -2.4250713655 -0.7272840017
H -5.0424863774 -1.9221380736 -2.285926787
C -7.4325177257 -0.0553677596 -0.8118381509
H -7.0469627598 -0.7138977153 -2.8584148909
H -7.7667109138 -1.9760900522 -1.8592136735
H -7.6819791501 0.8861709892 -1.3066944057
H -8.2886817884 -0.3398197659 -0.1966037596
N -5.0942460665 -0.3964889654 -0.8272741255

C -6.1726438102 0.102566263 0.0477595712
H -5.9961943506 1.1274027014 0.3702727578
H -6.2372941421 -0.5279513308 0.9479583305

Energy= -1436.0430153
Zero-point correction= 0.498646 (Hartree/Particle)
Thermal correction to Gibbs Free Energy= 0.440675
Sum of electronic and zero-point Energies= -1435.544369
Sum of electronic and thermal Energies= -1435.516955
Sum of electronic and thermal Enthalpies= -1435.516011
Sum of electronic and thermal Free Energies= -1435.602341
Imaginary Frequencies 1

Envelope-1-TS-wB97X-D

C 3.2780325132 3.3416215769 -0.2203714625
C 2.392728514 3.3551440669 0.8670392883
C 1.7982170078 2.1764933164 1.309479479
C 2.0303903195 0.9736380752 0.6402486024
C 3.0776710319 0.9538904892 -0.3103931681
C 3.6642890555 2.1179995776 -0.7849588735
H 2.1920045601 4.2945306142 1.3843188183
H 1.2149645798 2.2067703838 2.2268173541
H 4.4521066222 2.0888322417 -1.5396994903
C 2.7775810137 -1.2333015537 0.4472779603
C 3.2466534872 -2.5231248463 0.721531427
C 4.3533371527 -3.0269964959 0.0395931805
C 5.0209839714 -2.2538069951 -0.9186923181
C 4.6207839013 -0.9288046979 -1.1446839243
C 3.5353933683 -0.4304985695 -0.4419175107
H 2.8048169637 -3.1411901148 1.4998816425
H 4.7149987765 -4.0316189631 0.2640823078
H 5.1784164678 -0.3007266672 -1.8416584022
C 1.5868085266 -0.4299670379 0.8941158386
C -1.078394528 -0.5898547855 0.7757488814
C -1.8143240473 -1.7313232428 0.4709325451
C 0.3403684276 -1.0098867385 1.0234827063
C -1.7731421063 0.671372996 0.6397677875
C -3.0805184381 0.7045892969 0.0600850394
C -1.2516738913 1.9051338223 1.0804590025
H -0.3426906842 1.8818014014 1.6556914012
C 3.8585875576 4.5703292956 -0.6910855398
C 6.143682598 -2.8012293474 -1.6314785505
N 4.3212407546 5.5610112574 -1.0708578872
N 7.0449461057 -3.244590228 -2.206755978
C -1.8672864839 3.1093406028 0.8328883722
H -1.4131494144 4.035208755 1.1928288617
C -3.0673619846 3.143175107 0.0983310957
H -3.5275091626 4.0963348669 -0.1691573255
C -3.6629240948 1.9576230244 -0.2617889284
H -4.5931799784 1.9601242736 -0.8300979965
C -3.7930223883 -0.5193364997 -0.2125506899
C -3.1376632921 -1.7128302743 -0.0037838052
H -3.6360785151 -2.6644700415 -0.191340149
C 0.2757695068 -2.5215392857 1.2627763037
H 1.0822929194 -3.0233344597 0.7228029348
C -1.0534852864 -2.9948285968 0.6823526063
H -1.5923896941 -3.6824465873 1.3538389719
H -0.9162325924 -3.5265895709 -0.2733527581
C 0.4148169309 -2.8250687228 2.7583268824
H -0.4151094689 -2.3728766311 3.3237278878
H 0.397903847 -3.911408749 2.936390438
H 1.3534811218 -2.4195652714 3.1645044945
C -5.6969794408 -1.6185811537 -1.3242718731
C -7.1008392573 -1.132234603 -1.7029980833
H -5.7736653896 -2.4833521337 -0.6308969503
H -5.0881582522 -1.9324271493 -2.1846654179
C -7.4184670848 -0.0268992691 -0.6677662499

H -7.0973999849 -0.7183332375 -2.7208454576
H -7.8295270542 -1.954271737 -1.6860230956
H -7.6041563902 0.9330230007 -1.1700717758
H -8.3065323438 -0.2545639465 -0.0622133185
N -5.1080949438 -0.4613018243 -0.6746686167
C -6.1594626393 0.0587995138 0.2017706509
H -5.9397475906 1.0724801012 0.5584078247
H -6.2567277602 -0.5905296692 1.0966212597

Energy= -1435.2810395
Zero-point correction= 0.497251 (Hartree/Particle)
Thermal correction to Gibbs Free Energy= 0.438812
Sum of electronic and zero-point Energies= -1434.783789
Sum of electronic and thermal Energies= -1434.756227
Sum of electronic and thermal Enthalpies= -1434.755283
Sum of electronic and thermal Free Energies= -1434.842227
Imaginary Frequencies 1

Envelope-1-TS-wB97X-D-MeCN

C 3.2618225816 3.3793973954 -0.1410368503
C 2.3627835738 3.3778025793 0.9375358483
C 1.7689241079 2.1905928348 1.3557261472
C 2.0160216947 0.9983030486 0.6691597635
C 3.0716211992 0.9973888198 -0.2741563964
C 3.6625843873 2.1690110173 -0.7252550064
H 2.1488175754 4.3077142108 1.4670872845
H 1.1750703916 2.2061642827 2.2661970157
H 4.4574280507 2.1492322169 -1.4734548857
C 2.7580484896 -1.206728424 0.4302297107
C 3.216149786 -2.5104515795 0.6657423215
C 4.3180080515 -3.0020976465 -0.0314953454
C 4.9923265075 -2.2009219502 -0.9644148912
C 4.6076095801 -0.8651554756 -1.153766691
C 3.5244302575 -0.3838157714 -0.4358676002
H 2.7730361085 -3.152993576 1.4228232607
H 4.667657381 -4.0176169907 0.1622119195
H 5.166449565 -0.215388356 -1.8299955116
C 1.5744998958 -0.4082450344 0.8991409967
C -1.087260446 -0.5583665751 0.7719594716
C -1.826825977 -1.6986002333 0.4602872634
C 0.3241156879 -0.9881030257 1.0312058527
C -1.7729531638 0.7073811339 0.6142754874
C -3.0608949793 0.7465317568 -0.0108575825
C -1.267569477 1.9384605846 1.0853668193
H -0.3934006652 1.9089475792 1.7141238475
C 3.8438470185 4.6152992381 -0.5842358932
C 6.110650972 -2.7343056767 -1.6904898687
N 4.3090551499 5.6130787829 -0.943982661
N 7.0106560336 -3.1662182722 -2.2778490112
C -1.8666587798 3.1473055882 0.8132180602
H -1.4278848425 4.0694449105 1.2020253494
C -3.0317079897 3.1877567018 0.0234378614
H -3.4771443947 4.1432101212 -0.2621056822
C -3.620557143 2.0040114032 -0.3587034826
H -4.532273349 2.0190455303 -0.9564310052
C -3.7760751643 -0.4769623146 -0.2983632463
C -3.135007278 -1.6742419843 -0.0546039015
H -3.6332476707 -2.6250972813 -0.2461316942
C 0.2529554811 -2.4983401661 1.2710760902
H 1.0448635905 -3.0067977398 0.7164010397
C -1.0877663955 -2.9652335416 0.7136054575
H -1.6398877288 -3.6123179922 1.413626808
H -0.9692304716 -3.5370908864 -0.2205962045
C 0.4209178465 -2.8058910976 2.7622666112
H -0.3911056584 -2.3468595993 3.3487182564
H 0.3933789036 -3.8932555256 2.9319315262
H 1.3765263743 -2.4200382817 3.1484377672

C -5.6488928311 -1.6030896698 -1.4288525492
C -7.0305283596 -1.121139801 -1.8823908401
H -5.7580764349 -2.4284055762 -0.6962061954
H -5.0128109204 -1.9639901247 -2.2502032914
C -7.3760191449 0.0328820348 -0.9126976091
H -6.9849714422 -0.754952801 -2.9180144621
H -7.765763239 -1.9370364063 -1.8526034751
H -7.5320751781 0.9711055926 -1.4644525882
H -8.2865197067 -0.1615048991 -0.3290987769
N -5.0681175274 -0.4192564522 -0.8103466229
C -6.1525349783 0.1494465657 0.0005059277
H -5.9451915344 1.1773674125 0.3197935225
H -6.2876453518 -0.4635215437 0.9142449145

Energy= -1435.3220226
Zero-point correction= 0.496957 (Hartree/Particle)
Thermal correction to Gibbs Free Energy= 0.438800
Sum of electronic and zero-point Energies= -1434.825065
Sum of electronic and thermal Energies= -1434.797628
Sum of electronic and thermal Enthalpies= -1434.796684
Sum of electronic and thermal Free Energies= -1434.883222
Imaginary Frequencies 1

Envelope-1-unstable-2-CAM-B3LYP

C 2.7273184054 3.3865260836 0.2581111636
C 1.3565869025 3.1713660718 0.4606304459
C 0.8329481026 1.8891203216 0.4595126891
C 1.6764489117 0.7995294791 0.2358412796
C 3.0611683412 1.0255030868 0.0752903514
C 3.5908915261 2.3047126449 0.0755854762
H 0.706593924 4.0235468294 0.6227187049
H -0.2250764805 1.747899219 0.634407579
H 4.6545971016 2.4762407382 -0.0481519495
C 2.7537238251 -1.2791173068 0.1347691356
C 3.1333376764 -2.6140986979 -0.0206682044
C 4.4595462523 -2.9285082079 -0.2679178823
C 5.4234446073 -1.9173165162 -0.3873926787
C 5.0534833796 -0.5751980658 -0.2797870417
C 3.7265426552 -0.2688686955 -0.0304385665
H 2.4066337755 -3.4146521824 0.0394559285
H 4.7624266823 -3.9633222825 -0.379117288
H 5.7990809419 0.2025272169 -0.403979622
C 1.4283637898 -0.6532415845 0.2918794064
C -1.1133977714 -0.8418471244 0.1892487559
C -2.0415260663 -1.5795290152 0.9043148622
C 0.2354175127 -1.2947785408 0.5106163731
C -1.5948027486 0.0523869978 -0.8317499851
C -2.9939567243 0.3087127597 -0.8999759102
C -0.7681177322 0.5893105207 -1.8453573476
H 0.2885273421 0.3633148849 -1.8344639546
C -1.2859252923 1.3521474445 -2.8618316016
H -0.6273402271 1.7378909886 -3.6329607933
C -2.666717921 1.6001883556 -2.9301339192
H -3.0768487813 2.1717961066 -3.7558571886
C -3.4992907069 1.0765282185 -1.9746394378
H -4.5718588687 1.1907141523 -2.0680531056
C -3.8958431279 -0.3084679188 0.0461629733
C -3.4156561677 -1.3135569425 0.8619230734
H -4.0825696693 -1.8689872209 1.5094705026
C 0.1021597786 -2.6214648796 1.2697685111
H 0.2597581302 -3.4545351132 0.5720207515
C -1.3794707609 -2.6426446179 1.721544289
H -1.4463837608 -2.4144076612 2.7919371019
H -1.8557651531 -3.6177351476 1.5800020792
C 1.0257775342 -2.7789754232 2.4792739549
H 0.8815087253 -1.9443013931 3.170959762
H 0.779964832 -3.7020898539 3.0123421278

H 2.0814359041 -2.8143125221 2.218853382
C 6.7913035516 -2.2657766443 -0.6413304874
C 3.2444978068 4.7242885115 0.25761639
N 7.8944343685 -2.5492368228 -0.8454192504
N 3.6591217907 5.8046162874 0.2563606863
C -6.2144712624 -0.7517610247 0.7503904962
C -5.5876030591 1.4560785194 0.3951437541
C -7.5349669892 -0.0035122483 0.5306096016
H -5.9879206178 -0.8104290163 1.8282831031
H -6.2175958184 -1.7689817926 0.3516735449
C -7.1165536984 1.4690563667 0.3000412955
H -5.103866813 2.174404994 -0.2647051694
H -5.2608168619 1.675766911 1.4241624786
H -8.0522433703 -0.3961469319 -0.3469666806
H -8.2042019626 -0.1221078676 1.3849649533
H -7.4346729282 1.8074373276 -0.6884838394
H -7.5518571373 2.1502332502 1.0337741081
N -5.2377845984 0.0700549739 0.0466978319

Energy= -1436.0384699
Zero-point correction= 0.498839 (Hartree/Particle)
Thermal correction to Gibbs Free Energy= 0.439231
Sum of electronic and zero-point Energies= -1435.539631
Sum of electronic and thermal Energies= -1435.511292
Sum of electronic and thermal Enthalpies= -1435.510347
Sum of electronic and thermal Free Energies= -1435.599239
Imaginary Frequencies 0

Envelope-1-unstable-2-CAM-B3LYP-MeCN

C -2.4936457687 3.4240349555 -0.9779359169
C -1.1392528807 3.0766969542 -1.1166319486
C -0.7132490063 1.7862745868 -0.8553747153
C -1.6370355183 0.8235166866 -0.4336346113
C -3.0039084411 1.1792977108 -0.3407468799
C -3.4393821593 2.4681396768 -0.5986515142
H -0.4270773626 3.829048991 -1.4366282352
H 0.3311930018 1.5383899667 -0.9899666297
H -4.4885283202 2.7335439938 -0.5229344543
C -2.8604454953 -1.1070841922 0.0585999426
C -3.3411264936 -2.3625846824 0.4497055699
C -4.6876014301 -2.5277216073 0.7231702138
C -5.5734323398 -1.4409533199 0.6308440113
C -5.1092680991 -0.1711113347 0.279412441
C -3.7608629517 -0.0160949321 0.0057407943
H -2.6792347853 -3.2133612809 0.5512244306
H -5.066265586 -3.5000604861 1.0177786691
H -5.79308328 0.6697830336 0.2342086584
C -1.4974882368 -0.6184062796 -0.1915730078
C 1.0194549486 -0.9664978198 0.0228318931
C 1.9053153798 -1.9061818127 -0.4918007171
C -0.3441236951 -1.3755975073 -0.2506230071
C 1.554169127 0.0856945017 0.8521963614
C 2.9677438416 0.2545065189 0.902752571
C 0.7575292751 0.8698036243 1.7176868022
H -0.312825513 0.7188190862 1.727295451
C 1.3172032234 1.7799530056 2.5818911874
H 0.6789853644 2.3540781035 3.246116262
C 2.71116352 1.9372973846 2.6386008381
H 3.1540990468 2.6281877117 3.3483431613
C 3.5142890472 1.1784389977 1.8230605692
H 4.5903488948 1.2422238325 1.9239740646
C 3.8366598742 -0.6047927925 0.1197169046
C 3.2919409908 -1.7358070824 -0.4662829741
H 3.9220739885 -2.4634709479 -0.9616455957
C -0.3055644123 -2.8272297138 -0.7375674268
H -0.5725153866 -3.4883260472 0.0961492342
C 1.1805181774 -3.0651745876 -1.0929858754

H 1.3100459559 -3.0819029003 -2.1811695591
H 1.5656710102 -4.0170740912 -0.7158926555
C -1.1996048166 -3.1314736677 -1.9401154159
H -0.952040664 -2.4664760958 -2.7730105571
H -1.0273832375 -4.1599893609 -2.271019405
H -2.2623898051 -3.0254701674 -1.7276947884
C -6.9624581656 -1.6348982995 0.9163418409
C -2.9101166461 4.7662635702 -1.2483857331
N -8.0863248216 -1.7931707734 1.1466982579
N -3.2462817389 5.8528204177 -1.4667509509
C 6.1045381124 -1.3324322258 -0.4931320219
C 5.6683276262 0.9569101658 -0.4820714048
C 7.4826424786 -0.662057455 -0.4050856834
H 5.854757492 -1.5556145871 -1.5414460379
H 6.0364325319 -2.2630123275 0.0747756786
C 7.1855093331 0.8541709025 -0.3322430498
H 5.2385329756 1.8088352509 0.0400642945
H 5.3869344231 1.0321799818 -1.5431778361
H 8.0161964826 -0.993485761 0.4882866474
H 8.0955942792 -0.9250969901 -1.2693805737
H 7.4952353527 1.2588551278 0.6344468445
H 7.6986434055 1.4237127901 -1.1096192382
N 5.1917376871 -0.3308299001 0.057240655

Energy= -1436.078705
Zero-point correction= 0.497837 (Hartree/Particle)
Thermal correction to Gibbs Free Energy= 0.437959
Sum of electronic and zero-point Energies= -1435.580868
Sum of electronic and thermal Energies= -1435.552464
Sum of electronic and thermal Enthalpies= -1435.551520
Sum of electronic and thermal Free Energies= -1435.640746
Imaginary Frequencies 0

Envelope-1-unstable-2-wB97X-D

C -2.4189826195 3.4328992122 -0.9429131292
C -1.0704837968 3.0574830706 -1.0853750242
C -0.6668639833 1.7514687045 -0.8414113548
C -1.6097641113 0.8021610391 -0.4294284748
C -2.9703915845 1.1783289418 -0.3348007574
C -3.3812190117 2.4828270755 -0.5782031137
H -0.3387314485 3.8053679446 -1.3946150366
H 0.3798092528 1.4818508656 -0.9765169878
H -4.4315885194 2.7698242148 -0.5021375698
C -2.8698397143 -1.1183120637 0.0459726816
C -3.368577405 -2.3668297857 0.4384176933
C -4.7227310417 -2.5140476768 0.709772088
C -5.5972434001 -1.4159731359 0.6160106718
C -5.1063383502 -0.1510021554 0.2687249552
C -3.7512897288 -0.0120235405 -0.0032866134
H -2.7115262285 -3.2299238343 0.5456810322
H -5.1181976756 -3.4868501084 1.0060391507
H -5.7827792632 0.7046414787 0.2269810373
C -1.4942264627 -0.6493315502 -0.1985268634
C 1.0175696775 -1.0044834184 0.0218227345
C 1.9021361289 -1.9361022313 -0.5038931097
C -0.3537891113 -1.4170041134 -0.2470237564
C 1.5433673851 0.0499738778 0.8519445232
C 2.9565347728 0.238038615 0.887559567
C 0.7398754182 0.8258365005 1.7245475066
H -0.3356215237 0.6594341116 1.7446535838
C 1.2964260173 1.7542772335 2.574711339
H 0.652431017 2.3285399643 3.2437745745
C 2.6925276594 1.9333222239 2.6155441942
H 3.13386096 2.6400194161 3.3210340051
C 3.5016817884 1.1772817332 1.7990831902
H 4.585613639 1.2507978572 1.8893077101
C 3.8277574655 -0.6117684809 0.0983251811

C 3.2930711138 -1.7480197704 -0.4909185208
H 3.9322967363 -2.4701361742 -0.9987783187
C -0.3138253952 -2.8699622212 -0.7300016333
H -0.5741142628 -3.534901836 0.1133692066
C 1.1740589986 -3.1032148513 -1.0923373703
H 1.2989625053 -3.1303460645 -2.1879717355
H 1.5687990515 -4.0567304241 -0.7087072683
C -1.2173341012 -3.1744537955 -1.9262203339
H -0.9752550174 -2.4994791346 -2.7613587983
H -1.0531122867 -4.2084621208 -2.265663694
H -2.2846576303 -3.0588996958 -1.7068627471
C -6.9963279178 -1.5913224191 0.8982198907
C -2.8114754677 4.7935359513 -1.1928596608
N -8.1223885791 -1.7355582171 1.1250450998
N -3.1237987161 5.8902428766 -1.3930867515
C 6.1264490829 -1.2995755537 -0.457656315
C 5.62109722 0.9658157895 -0.5469035317
C 7.4836157167 -0.5855262065 -0.3627177626
H 5.9166241083 -1.5813203219 -1.5108371666
H 6.0685068408 -2.2145974778 0.1497382544
C 7.1376318205 0.9218677321 -0.3565082525
H 5.141832452 1.8262806262 -0.0656129026
H 5.3612937434 1.0039320149 -1.6248394193
H 8.0058526768 -0.8684265417 0.5614263314
H 8.1353607219 -0.8643646341 -1.2019505826
H 7.4095414294 1.3760382694 0.6070983157
H 7.6600522347 1.4840157133 -1.1424694921
N 5.1788065098 -0.3091169986 0.0215653608

Energy= -1435.3187297
Zero-point correction= 0.497320 (Hartree/Particle)
Thermal correction to Gibbs Free Energy= 0.437588
Sum of electronic and zero-point Energies= -1434.821410
Sum of electronic and thermal Energies= -1434.793067
Sum of electronic and thermal Enthalpies= -1434.792123
Sum of electronic and thermal Free Energies= -1434.881142
Imaginary Frequencies 0

Envelope-1-unstable-2-wB97X-D-MeCN

C -2.4109689713 3.4202659268 -0.986859874
C -1.0613347457 3.0393454758 -1.1372893732
C -0.6624292932 1.7358752626 -0.8816315401
C -1.608265095 0.7906204766 -0.4528191274
C -2.9700371077 1.1774775238 -0.3518631328
C -3.3785755374 2.481310587 -0.6046243317
H -0.3278130967 3.7789386088 -1.4633941235
H 0.3819373366 1.4624764551 -1.0283484415
H -4.4282040379 2.7699818484 -0.5196810051
C -2.8648201775 -1.1132467415 0.0567977985
C -3.370881195 -2.3657646976 0.442987107
C -4.724550183 -2.5083953747 0.7099226282
C -5.5960393541 -1.4036187029 0.6136790969
C -5.1067702574 -0.1379807548 0.2645426346
C -3.7497596852 -0.0043930709 -0.0027579221
H -2.7206666066 -3.2345110961 0.5459302534
H -5.1220644189 -3.482117602 1.0019719363
H -5.7786398621 0.7211482457 0.2129407847
C -1.4967777546 -0.6481108151 -0.1961855672
C 1.0096801592 -1.0127975755 0.0522136582
C 1.9050854357 -1.9719267809 -0.428692813
C -0.3444350751 -1.4217707991 -0.2311394616
C 1.5375418759 0.0632503836 0.8589525511
C 2.9510795674 0.2517577118 0.8926071044
C 0.7339146045 0.8469044198 1.7230503463
H -0.3429205157 0.6857350273 1.7390452448
C 1.2900236166 1.7702053309 2.5830560721
H 0.6444733508 2.3477021464 3.2487864819

C 2.6859598864 1.9319934494 2.6400842054
H 3.1308484494 2.6260917633 3.3562515502
C 3.4950795873 1.1746119361 1.820003241
H 4.5769173447 1.2475629735 1.9303298678
C 3.826206704 -0.6068246198 0.0989397781
C 3.2900248145 -1.7903269796 -0.4158868137
H 3.9259857448 -2.5358738551 -0.892113989
C -0.3133097172 -2.8757720257 -0.7008734245
H -0.6363511417 -3.5205738976 0.1350980128
C 1.1797637812 -3.1525488232 -0.9871051274
H 1.3554113207 -3.2371158041 -2.0721614701
H 1.5437975379 -4.0883419525 -0.5364432838
C -1.1759499963 -3.164152061 -1.9295181933
H -0.8784487125 -2.5113144791 -2.765212753
H -1.0324965667 -4.208262218 -2.2470456843
H -2.2476351394 -3.0155756143 -1.7488035383
C -6.9934384842 -1.5749265547 0.8910120981
C -2.7989014484 4.7765120592 -1.2493240333
N -8.1218395451 -1.7152989354 1.1142942337
N -3.1098525725 5.8726409088 -1.4609115784
C 6.0912254385 -1.2853071355 -0.5743220905
C 5.6452110643 1.025890267 -0.4396045682
C 7.4460293053 -0.5509199114 -0.6283304029
H 5.7925270817 -1.6204567745 -1.584598423
H 6.1009424867 -2.1712619122 0.0778659378
C 7.1403284028 0.8938817558 -0.1975098056
H 5.1767413219 1.8397902033 0.1212525908
H 5.4343040861 1.1927397069 -1.5139802149
H 8.1903836618 -1.0236808201 0.0266090582
H 7.8464903092 -0.5780522996 -1.6514955077
H 7.3484811619 1.0330000733 0.8745641232
H 7.7205880687 1.6410243419 -0.755890808
N 5.1481625776 -0.2994776841 -0.0523941423

Energy= -1435.35943
Zero-point correction= 0.495825 (Hartree/Particle)
Thermal correction to Gibbs Free Energy= 0.435301
Sum of electronic and zero-point Energies= -1434.863605
Sum of electronic and thermal Energies= -1434.835074
Sum of electronic and thermal Enthalpies= -1434.834130
Sum of electronic and thermal Free Energies= -1434.924129
Imaginary Frequencies 0

Envelope-1-Inv-CAM-B3LYP

C 5.464103321 -1.9346191512 0.2926227579
C 4.4641666284 -2.9114873876 0.3619932481
C 3.1270004619 -2.5690348507 0.2245334031
C 2.7696280711 -1.2392820039 -0.0031341847
C 3.7888272243 -0.2638393988 -0.0553999584
C 5.1243078417 -0.5956616523 0.0877128547
H 4.7444914474 -3.9448071273 0.5310339613
H 2.3859991728 -3.3506634132 0.3073005191
H 5.9014371707 0.1595546957 0.0463132816
C 1.7707947695 0.8657905992 -0.3272177245
C 0.9676431192 1.9534674186 -0.6696517654
C 1.5483552416 3.1937725991 -0.8865966915
C 2.9345210185 3.3641847932 -0.7825415108
C 3.7573729445 2.2742080857 -0.4912746237
C 3.1696694195 1.0390214239 -0.280965514
H -0.1017028643 1.847776895 -0.7797131365
H 0.9281871031 4.0445296499 -1.1443531872
H 4.8333188533 2.4033942557 -0.4508054208
C 1.4546627787 -0.5676015965 -0.1321717988
C -1.1122418792 -0.5873601721 -0.0801544599
C -1.9733447895 -1.3646956483 -0.8339115906
C 0.2336021652 -1.1718639078 -0.1549287582
C -1.6414669642 0.4856767469 0.7014856579

C -3.0163067294 0.8259770406 0.5408401621
C -0.878318851 1.1962602883 1.659628489
H 0.1459029345 0.8941025314 1.8377179101
C -1.4149042121 2.2473844493 2.3559816158
H -0.8112972145 2.7748158033 3.0872326078
C -2.7384011294 2.6600057111 2.1082447207
H -3.1399460032 3.5285009594 2.6194604509
C -3.5182060632 1.9612491961 1.224490478
H -4.5333562596 2.278469464 1.0220763721
C -3.8691213199 0.0246332793 -0.2999192081
C -3.3327550533 -1.0615776852 -0.964676735
H -3.9671429227 -1.7131152007 -1.5544717864
C 0.0105427474 -2.6420422424 -0.4943625987
H 0.8544648801 -3.0456309462 -1.0557041874
C -1.2531250174 -2.5637355414 -1.3875658762
H -1.8581263796 -3.4744164153 -1.3449786701
H -0.9709584332 -2.4053889229 -2.4349675191
C -0.258963062 -3.5055236847 0.7447122949
H -1.1559045872 -3.1572920514 1.2637382935
H -0.4162902358 -4.5486674327 0.4560851891
H 0.5675355016 -3.4698044225 1.4580103768
C 3.5116897975 4.6594485059 -1.0003473565
C 6.8402605679 -2.311537332 0.4413917346
N 3.9758533659 5.7048527544 -1.1745694743
N 7.9495567947 -2.6174959191 0.561418112
C -6.0623831868 0.1332605178 0.7940435684
C -6.0249910272 -0.1448194687 -1.5018868934
C -6.6773360594 -1.2596142153 0.5667545736
H -6.8464902586 0.8982383079 0.8355838789
H -5.474392281 0.2019384445 1.7082479237
C -6.7713003159 -1.3910759963 -0.9701094415
H -5.4045504184 -0.3291724202 -2.3784860246
H -6.7497776161 0.6294089073 -1.7766721136
H -6.0142607573 -2.0260022329 0.976346483
H -7.6443537294 -1.3653953166 1.0634859181
H -6.3037365956 -2.3174368597 -1.3130711304
H -7.8052705553 -1.406082859 -1.3220270194
N -5.2264441502 0.3807923115 -0.3938995371

Energy= -1436.0396847
Zero-point correction= 0.499340 (Hartree/Particle)
Thermal correction to Gibbs Free Energy= 0.440305
Sum of electronic and zero-point Energies= -1435.540345
Sum of electronic and thermal Energies= -1435.512221
Sum of electronic and thermal Enthalpies= -1435.511277
Sum of electronic and thermal Free Energies= -1435.599380
Imaginary Frequencies 0

Envelope-1-Inv-CAM-B3LYP-MeCN

C 5.4623994308 -1.9289943713 0.3040608082
C 4.4647771563 -2.9122018705 0.3599929225
C 3.1289016207 -2.5711203878 0.214887265
C 2.7718047407 -1.2376521747 -0.0050241448
C 3.7921530763 -0.2601276991 -0.0457119066
C 5.1281441025 -0.5872844549 0.1042372357
H 4.7437398399 -3.9471212113 0.5229338774
H 2.3907713776 -3.3564835059 0.284420671
H 5.9015147285 0.172425016 0.0707147106
C 1.7739531078 0.8643297136 -0.3317354053
C 0.9717961442 1.9537935804 -0.6803809656
C 1.5515351388 3.1951489445 -0.8895611273
C 2.9386775459 3.3633585833 -0.7689549005
C 3.7648651907 2.2763859355 -0.4724216739
C 3.1740053502 1.0410149888 -0.2724138315
H -0.0959754053 1.8489333757 -0.8064452756
H 0.9321249506 4.0448988981 -1.1536470583
H 4.8407613403 2.4024930025 -0.4181107857

C 1.4594034364 -0.5671054461 -0.140461046
C -1.1076778791 -0.5884660027 -0.0915868273
C -1.9714305944 -1.365231554 -0.8480666072
C 0.2340241066 -1.173572616 -0.1729393188
C -1.6388198961 0.4836383922 0.6928087905
C -3.0165844511 0.8196976607 0.537727465
C -0.87610476 1.195010432 1.6516820928
H 0.1479216376 0.8934657167 1.8333789734
C -1.4126041613 2.2450250829 2.3522724744
H -0.8087950915 2.7708799614 3.0851189207
C -2.7374153477 2.655512875 2.1080367711
H -3.1414680997 3.5212935921 2.6224794091
C -3.5178981187 1.9536268512 1.2251945601
H -4.5361156017 2.2703306564 1.0368747817
C -3.8715128496 0.0175112017 -0.3067664158
C -3.3298776347 -1.0631060923 -0.9801384369
H -3.9605429012 -1.7137801317 -1.5740821426
C 0.0141469908 -2.641181572 -0.5221959895
H 0.8564726375 -3.0400950007 -1.0882453428
C -1.255035652 -2.5622076618 -1.4060042717
H -1.8614605975 -3.4709273694 -1.3596906151
H -0.980614421 -2.3999524636 -2.4546451389
C -0.2467223993 -3.5145332318 0.7117408697
H -1.1507858153 -3.1858208636 1.2322421385
H -0.3888413658 -4.5559626353 0.4094084037
H 0.5790508967 -3.4771585073 1.426272685
C 3.5155112838 4.6575361727 -0.9769642905
C 6.8358232173 -2.3037859257 0.4602315323
N 3.9810836617 5.7042986734 -1.1443124452
N 7.9458986154 -2.6074195606 0.5863123611
C -6.0712754318 0.1741558214 0.787293243
C -6.0295628631 -0.1886548836 -1.4995608849
C -6.7154178553 -1.2117607522 0.6013964617
H -6.8380162867 0.9562258927 0.8008327984
H -5.4872455104 0.2541389472 1.7025684697
C -6.7605098134 -1.421036611 -0.9289886391
H -5.4180442381 -0.3934166652 -2.3776153011
H -6.7622090122 0.5756676343 -1.780766325
H -6.0940271006 -1.9763802208 1.0749419752
H -7.7032939039 -1.257672157 1.0656474298
H -6.2521523729 -2.3464711865 -1.210736433
H -7.7819573884 -1.4795308842 -1.3117842488
N -5.2245380858 0.3693902284 -0.4078013623

Energy= -1436.0789993
Zero-point correction= 0.498538 (Hartree/Particle)
Thermal correction to Gibbs Free Energy= 0.439328
Sum of electronic and zero-point Energies= -1435.580462
Sum of electronic and thermal Energies= -1435.552302
Sum of electronic and thermal Enthalpies= -1435.551358
Sum of electronic and thermal Free Energies= -1435.639671
Imaginary Frequencies 0

Envelope-1-Inv-wB97X-D

C 5.608298987 -1.4954953193 0.0446422346
C 4.7321172536 -2.590300839 0.1211782251
C 3.3544057565 -2.4042962163 0.0752351455
C 2.8300311546 -1.1141885177 -0.0642506518
C 3.724489675 -0.0194486914 -0.1287552692
C 5.1003229526 -0.1955003347 -0.0763497386
H 5.1421993558 -3.5962683238 0.2225416253
H 2.7122786254 -3.2782622345 0.1586244952
H 5.7822311675 0.6552690458 -0.1258756256
C 1.5712213062 0.8679515044 -0.2442728484
C 0.6210736609 1.8610356222 -0.5040324169
C 1.0346034365 3.1746434984 -0.692885757
C 2.397668213 3.5133396269 -0.6452105482

C 3.3644498651 2.519632548 -0.4443453772
C 2.9420257849 1.2096823558 -0.2608768816
H -0.4387684824 1.6252334587 -0.5731168958
H 0.2961979715 3.9544322392 -0.8851016889
H 4.4246503667 2.778832407 -0.4504220866
C 1.4407911386 -0.5979367016 -0.0831102459
C -1.0911895778 -0.8945682972 0.1887420349
C -1.9368372074 -1.7409741977 -0.5122149828
C 0.2926347781 -1.3355625116 -0.0265875194
C -1.6460980805 0.1099455807 1.0422417108
C -3.056193171 0.3289367845 0.9995166798
C -0.8679783501 0.8746101499 1.9505524125
H 0.1962666359 0.6579317104 2.0455485843
C -1.4338720969 1.8782606569 2.699806396
H -0.8153160408 2.4546478192 3.3907801903
C -2.8056099022 2.1818230536 2.5596709343
H -3.2381832126 3.0194097269 3.1104802103
C -3.5964102479 1.4187143502 1.733819583
H -4.6549631648 1.6523339857 1.6183592035
C -3.9050544499 -0.543081645 0.2200730015
C -3.328007753 -1.5687573061 -0.5153776738
H -3.9518410208 -2.2764119483 -1.0630606401
C 0.189793106 -2.8133839414 -0.3806901408
H 1.020111989 -3.1201744606 -1.0305798335
C -1.1509259983 -2.8509087954 -1.1557304377
H -1.6599349543 -3.8244867849 -1.0866203756
H -0.9820322011 -2.6406117675 -2.225067989
C 0.1337140009 -3.711019639 0.8613152226
H -0.7478494673 -3.4663554927 1.4735684146
H 0.0663356305 -4.7697610164 0.5691448581
H 1.0217974949 -3.5844142629 1.4970158007
C 2.8018322109 4.8813975019 -0.8298808961
C 7.0294446947 -1.7107592935 0.0982071501
N 3.12474413 5.9833143923 -0.9766168447
N 8.1727175293 -1.8866615151 0.1415061451
C -5.9830789598 -0.6303900626 1.517013548
C -6.1418689257 -0.9057615949 -0.7693905366
C -6.5059345824 -2.0689118858 1.339380583
H -6.8208392441 0.0765964859 1.6434586907
H -5.3152564292 -0.5181574232 2.3797484599
C -6.7165129636 -2.2165121679 -0.1838277576
H -5.600271745 -1.0276658676 -1.7155020347
H -6.9607739258 -0.1922011823 -0.9593763946
H -5.7483073872 -2.7827583152 1.6952468552
H -7.4215028722 -2.2484511522 1.9202861473
H -6.1853774696 -3.0980400044 -0.5719277497
H -7.7755848866 -2.3384115416 -0.4512699484
N -5.2877351511 -0.3285332429 0.2605022843

Energy= -1435.320368
Zero-point correction= 0.497945 (Hartree/Particle)
Thermal correction to Gibbs Free Energy= 0.438900
Sum of electronic and zero-point Energies= -1434.822423
Sum of electronic and thermal Energies= -1434.794332
Sum of electronic and thermal Enthalpies= -1434.793387
Sum of electronic and thermal Free Energies= -1434.881468
Imaginary Frequencies 0

Envelope-1-Inv-wB97X-D-MeCN

C 5.4813572804 -1.9073343334 0.341855167
C 4.4955754373 -2.9105827669 0.3443141952
C 3.1551944637 -2.5874887068 0.1671063582
C 2.7813053244 -1.2506170049 -0.0291179254
C 3.7880269147 -0.2530187889 -0.021222655
C 5.1289518154 -0.5629920219 0.1614584031
H 4.7873415609 -3.9520270448 0.4903913872
H 2.4248021147 -3.3930769841 0.1931749367

H 5.8930598549 0.2167050164 0.1663424166
C 1.7565978024 0.8411975029 -0.3530267034
C 0.9413033766 1.9212368625 -0.7166896368
C 1.5021929423 3.1815224234 -0.8825634962
C 2.8844583753 3.3782066438 -0.7052433266
C 3.7252974243 2.2995274747 -0.3991805603
C 3.1526835518 1.0441244741 -0.2425979224
H -0.1256426087 1.7942539097 -0.8888517635
H 0.8684458879 4.0264689971 -1.1573124845
H 4.8028457981 2.4455518514 -0.3026752155
C 1.4628232938 -0.5966701826 -0.183446269
C -1.1005549473 -0.6291376608 -0.1261782977
C -1.9725527741 -1.4042024248 -0.8823790093
C 0.2385160695 -1.2136924019 -0.2237333505
C -1.6191875049 0.4493951943 0.6598828324
C -2.9942345547 0.8042573203 0.5004514083
C -0.8435621922 1.1589088696 1.6148716128
H 0.1807625201 0.8384676165 1.8079192391
C -1.3610696638 2.236939498 2.2951043435
H -0.7445881189 2.7664639967 3.0252208555
C -2.677869145 2.6737824575 2.0341330066
H -3.0666000026 3.5665217933 2.5289243855
C -3.47455555 1.9661593784 1.1632295563
H -4.491801545 2.3054629315 0.9658494773
C -3.8660544823 -0.0032113165 -0.3303093385
C -3.3323984709 -1.0951229594 -1.0061484947
H -3.97492057 -1.7516836209 -1.5936662162
C 0.017262006 -2.6793188706 -0.5701059397
H 0.8598853833 -3.0799546686 -1.1481178478
C -1.2593314543 -2.6022878583 -1.4420346139
H -1.870834448 -3.5154584595 -1.3936710308
H -0.9930659525 -2.4351200329 -2.4989130648
C -0.220501157 -3.5445696446 0.6734608923
H -1.1280644941 -3.2197288305 1.2061233506
H -0.3528897509 -4.5971486008 0.3814614471
H 0.619366892 -3.4873025907 1.3810565178
C 3.4424924873 4.6914760744 -0.866819689
C 6.8596085773 -2.2637259858 0.5312150813
N 3.8908099835 5.7517346475 -0.9961737654
N 7.9708671298 -2.5526679912 0.6840363816
C -6.0454771867 0.2383678961 0.794224483
C -6.0495484464 -0.2147732786 -1.4739816013
C -6.7658399642 -1.1187731082 0.6659201391
H -6.7728877828 1.0666423923 0.7986129351
H -5.4408575515 0.3127947982 1.7055399625
C -6.7966199991 -1.4088898522 -0.851073712
H -5.4621686754 -0.4610926403 -2.3665980114
H -6.7717054533 0.5665108534 -1.7635658112
H -6.1970044904 -1.8956106765 1.1977956436
H -7.7688659429 -1.0808722261 1.1141503999
H -6.2822803843 -2.3540006463 -1.0795531136
H -7.8189543309 -1.4871689248 -1.2468833381
N -5.2126162529 0.3406143911 -0.4140416706

Energy= -1435.3597471
Zero-point correction= 0.497026 (Hartree/Particle)
Thermal correction to Gibbs Free Energy= 0.437619
Sum of electronic and zero-point Energies= -1434.862721
Sum of electronic and thermal Energies= -1434.834556
Sum of electronic and thermal Enthalpies= -1434.833612
Sum of electronic and thermal Free Energies= -1434.922128
Imaginary Frequencies 0

Envelope-1-Inv-TS-CAM-B3LYP

C 3.500500537 3.2602251922 -0.097507119
C 2.5229061058 3.3298115793 0.8985519072
C 1.8144360486 2.1951039749 1.2718093751

C 2.0282263853 0.9825814729 0.6215362619
C 3.1507247794 0.899382714 -0.2309677457
C 3.8528777001 2.0197347717 -0.6358182611
H 2.3394339484 4.2732589346 1.3994169429
H 1.1565693613 2.2633209551 2.1259187563
H 4.6979034113 1.9431449645 -1.3109313261
C 2.6469395209 -1.2659747722 0.4670967553
C 3.010261654 -2.582652078 0.7571316594
C 4.133682116 -3.1478723695 0.164029346
C 4.9242149764 -2.4118657052 -0.7200361872
C 4.6300895877 -1.0659004495 -0.9568611592
C 3.5260363762 -0.508031144 -0.3420835501
H 2.4741992696 -3.1754190738 1.4840995617
H 4.410412387 -4.1688554233 0.4004803639
H 5.2802161966 -0.4731937482 -1.5908418377
C 1.4763976724 -0.3905367997 0.8265974353
C -1.1841991128 -0.3901030442 0.5433017924
C -1.9655448366 -1.4858817692 0.2015765333
C 0.1927032349 -0.8892564386 0.8745232488
C -1.809492179 0.9032722083 0.3844101284
C -3.0831743262 1.0035480522 -0.2561178504
C -1.2601440417 2.1025589858 0.8721321266
H -0.3774669652 2.033541983 1.4703214324
C 4.2044457468 4.4451312362 -0.4951232308
C 6.0642346571 -3.0214135154 -1.3414041387
N 4.7685274491 5.4024731925 -0.8171476377
N 6.9815048838 -3.5153646841 -1.8444950465
C -1.822356437 3.331102582 0.6388475073
H -1.3510592172 4.2236894837 1.0377289496
C -2.9940739651 3.430792206 -0.1266284065
H -3.4136117381 4.4015364069 -0.3680005604
C -3.6123191011 2.2831526674 -0.5489716459
H -4.521149816 2.3352985013 -1.1347580688
C -3.8274676179 -0.1782983973 -0.6007610279
C -3.2528728466 -1.398158401 -0.3465370286
H -3.7873441686 -2.3183357642 -0.5533336817
C 0.0271569716 -2.3957663686 1.1054934757
H 0.8234913608 -2.940408574 0.608676606
C -1.2982846017 -2.7918564795 0.4615322365
H -1.9063343199 -3.4316097242 1.1105348498
H -1.1469288665 -3.3445334954 -0.4725907778
C 0.0632617844 -2.7095343054 2.6056672348
H -0.7646452367 -2.2125207942 3.1202947242
H -0.0315680552 -3.7862804172 2.7760120159
H 0.9925060723 -2.3661043196 3.06706482
N -5.100264218 -0.0262077704 -1.1778447732
C -5.7059593173 -1.1668347026 -1.8660051225
H -6.300641774 -0.7678307261 -2.6950763591
H -4.9430059194 -1.8126431724 -2.2997283728
C -6.1728297244 0.4612143358 -0.2925514496
H -5.774382052 1.1127546636 0.4838556305
H -6.8834151462 1.0418856775 -0.89216893
C -6.8436172357 -0.8094653096 0.2607354527
H -7.8964394588 -0.6440679871 0.5001993315
H -6.3437158693 -1.1185900783 1.182261839
C -6.636345936 -1.8664347567 -0.8476235537
H -6.1798584986 -2.773130036 -0.442853229
H -7.576332456 -2.1642036366 -1.3177282824

Energy= -1435.9994071
Zero-point correction= 0.499000 (Hartree/Particle)
Thermal correction to Gibbs Free Energy= 0.440757
Sum of electronic and zero-point Energies= -1435.500407
Sum of electronic and thermal Energies= -1435.472976
Sum of electronic and thermal Enthalpies= -1435.472031
Sum of electronic and thermal Free Energies= -1435.558650
Imaginary Frequencies 1

Envelope-1-Inv-TS-CAM-B3LYP-MeCN

C 3.4755852962 3.2637355569 -0.0507961392
C 2.4973489618 3.3207136732 0.9483051038
C 1.7974679128 2.1777543245 1.309832672
C 2.0209696577 0.9728298 0.6445060573
C 3.1414955312 0.906992163 -0.2138463344
C 3.8393381218 2.0342506309 -0.6078769845
H 2.304997007 4.2569143901 1.4598808064
H 1.1385997914 2.2345212224 2.1634349456
H 4.6811778206 1.9656809528 -1.2882770571
C 2.637108776 -1.2730799353 0.4394510671
C 2.9944647027 -2.6032638087 0.6836582687
C 4.1066869729 -3.1560206908 0.0603147884
C 4.8934596321 -2.3924184489 -0.806459935
C 4.6103593627 -1.0369035558 -1.0009579478
C 3.5138820043 -0.4963244002 -0.3577874973
H 2.466712057 -3.2202698362 1.3955819141
H 4.375652456 -4.1869547797 0.2609295256
H 5.2544257603 -0.42305726 -1.6211923084
C 1.477926649 -0.4029095498 0.8379718852
C -1.1824508851 -0.4009353256 0.5948037592
C -1.9737775864 -1.5002509197 0.2780400678
C 0.191796997 -0.9032755321 0.9160271937
C -1.8006914591 0.8936667594 0.4055215985
C -3.0621874174 0.9885362741 -0.2624894131
C -1.2632354739 2.0977465466 0.8992888979
H -0.4089071403 2.0326702806 1.5396881285
C 4.1696294173 4.4560187516 -0.4357416576
C 6.0230156223 -2.9875797497 -1.454800343
N 4.7270160868 5.4207703903 -0.749826599
N 6.9344535105 -3.4698846671 -1.9810310465
C -1.8133499408 3.3266616828 0.6331988126
H -1.3541458147 4.2216566717 1.0414783743
C -2.960217869 3.4202011684 -0.170961835
H -3.3702763904 4.3889031209 -0.4372276043
C -3.5745908246 2.2674937981 -0.590015008
H -4.4732982229 2.3225405415 -1.1912965379
C -3.8103571988 -0.1985961034 -0.5958688825
C -3.2509676226 -1.4165333733 -0.2937726436
H -3.7878474025 -2.3383664414 -0.4849878567
C 0.0300314282 -2.4066745017 1.1631927718
H 0.7932867577 -2.9575711284 0.6230784341
C -1.32860362 -2.8031000306 0.5934855493
H -1.9308481164 -3.3767921496 1.3062253201
H -1.23235745 -3.4205355764 -0.305916471
C 0.1525516381 -2.7173383315 2.6587045484
H -0.640478384 -2.2146522046 3.2215807287
H 0.0582622246 -3.7938498825 2.8306133386
H 1.112263603 -2.386482026 3.0642525687
N -5.0602072599 -0.0568742108 -1.2156807271
C -5.666702491 -1.2243840433 -1.8646685858
H -6.2390850462 -0.8535008876 -2.7218768855
H -4.9054134296 -1.9025860961 -2.2489011611
C -6.1596926083 0.4979305127 -0.3986045047
H -5.7815460542 1.1747976859 0.3656857468
H -6.8281148302 1.0637174716 -1.0565001877
C -6.8855624208 -0.7278199952 0.1846015299
H -7.9487413295 -0.5272537464 0.336267744
H -6.4576961819 -0.9844772378 1.1573003024
C -6.6267571687 -1.8580675327 -0.8368510479
H -6.1705033879 -2.7247660575 -0.3522102582
H -7.5443395917 -2.1999738436 -1.3211370495

Energy= -1436.0404718
Zero-point correction= 0.498217 (Hartree/Particle)
Thermal correction to Gibbs Free Energy= 0.439766
Sum of electronic and zero-point Energies= -1435.542255
Sum of electronic and thermal Energies= -1435.514802

Sum of electronic and thermal Enthalpies= -1435.513858
Sum of electronic and thermal Free Energies= -1435.600706
Imaginary Frequencies 1

Envelope-1-Inv-TS-wB97X-D

C 3.4296969111 3.2804594658 -0.0887985122
C 2.4788685645 3.3305114836 0.9408928699
C 1.7957617637 2.1812122435 1.3287082156
C 2.0060653098 0.9745417511 0.6589116729
C 3.1080884503 0.9082043356 -0.2251581478
C 3.7848820769 2.0439560163 -0.645527638
H 2.2961022223 4.2739728575 1.457417421
H 1.1576579128 2.232715765 2.207813966
H 4.6159554833 1.9804765355 -1.350099796
C 2.6434376828 -1.26791856 0.4808965934
C 3.0227400193 -2.5863377103 0.7593180137
C 4.1382923824 -3.1425988869 0.135159652
C 4.9045528459 -2.3953987877 -0.7683962749
C 4.5927602932 -1.0470131718 -0.9950006821
C 3.4966872658 -0.4976271533 -0.3492562098
H 2.502402261 -3.1896824405 1.4996089299
H 4.429094287 -4.1691811375 0.3632473227
H 5.2247608767 -0.4420494821 -1.64753233
C 1.4745710078 -0.4046829931 0.8685910279
C -1.1806842447 -0.4180960494 0.5864781601
C -1.9619383407 -1.5177407141 0.2415127029
C 0.19255518 -0.9158137722 0.9214447797
C -1.7941441581 0.8788761825 0.4065587098
C -3.052392457 0.9837657975 -0.2673780418
C -1.2439768667 2.0798371199 0.8994039775
H -0.379762323 2.0049687822 1.5362194259
C 4.1042253275 4.4808200212 -0.5041168999
C 6.035885247 -2.9956799351 -1.4225223552
N 4.6428515228 5.4490257126 -0.8391315651
N 6.9441374393 -3.4816004403 -1.950531573
C -1.7831222375 3.3159022856 0.6307989019
H -1.310513081 4.2134673719 1.0359027475
C -2.9309152162 3.4183346243 -0.1768002669
H -3.3307243095 4.3964818806 -0.4509373239
C -3.555878253 2.2686549996 -0.5987033978
H -4.4533282979 2.3262042133 -1.2146982546
C -3.8060662065 -0.1989332623 -0.6064737167
C -3.2438625851 -1.4257261617 -0.3261522782
H -3.7868872934 -2.3497216805 -0.5302146637
C 0.0328019582 -2.4213369401 1.1536529834
H 0.8312151044 -2.9654426927 0.6426420403
C -1.2963119675 -2.8217168654 0.5190711268
H -1.9112840423 -3.4528359172 1.1809309983
H -1.1509769275 -3.3889864535 -0.4145688104
C 0.0969794287 -2.7349421388 2.6520749102
H -0.7277069304 -2.2375425149 3.1863713911
H 0.0118760729 -3.8189091299 2.8253016473
H 1.0401519447 -2.3835847753 3.0963140477
N -5.067716909 -0.0473589674 -1.1919751414
C -5.6875065916 -1.1767981277 -1.8729201064
H -6.268555327 -0.7746775919 -2.7194014887
H -4.9332451997 -1.8512587971 -2.2965002319
C -6.140033752 0.4962536021 -0.3500413203
H -5.7401933065 1.1551127212 0.430188097
H -6.8225415997 1.0927848995 -0.9792172466
C -6.8625026733 -0.7424682766 0.2149516537
H -7.9250322516 -0.5428976567 0.4130479245
H -6.4018230613 -1.0350395324 1.1700339547
C -6.6414033364 -1.8379605433 -0.8518584402
H -6.1914084368 -2.7370008842 -0.4057208182
H -7.578713589 -2.1535145139 -1.3313737434

Energy= -1435.2787924
Zero-point correction= 0.497747 (Hartree/Particle)
Thermal correction to Gibbs Free Energy= 0.439801
Sum of electronic and zero-point Energies= -1434.781046
Sum of electronic and thermal Energies= -1434.753700
Sum of electronic and thermal Enthalpies= -1434.752756
Sum of electronic and thermal Free Energies= -1434.838992
Imaginary Frequencies 1

Envelope-1-Inv-TS-wB97X-D-MeCN

C 3.3961387263 3.2833009879 -0.0454897352
C 2.4474467904 3.3191060917 0.989545768
C 1.7772133204 2.1603210246 1.3688678641
C 1.9985787193 0.9609881406 0.6851266232
C 3.0951383842 0.91456945 -0.2088317255
C 3.7636695349 2.0586388009 -0.6211421336
H 2.2537250424 4.2552954152 1.5158626999
H 1.1405313431 2.2001125012 2.2489562984
H 4.5893858036 2.0044702788 -1.3333020506
C 2.6332527141 -1.2781579228 0.4496960782
C 3.006131274 -2.6115196888 0.6740091893
C 4.1065248166 -3.1524717192 0.012355712
C 4.865930399 -2.3740447335 -0.8733878018
C 4.5653273905 -1.0154963487 -1.0508811073
C 3.4807560478 -0.4862585856 -0.3695780179
H 2.4970575182 -3.2435281666 1.3974307347
H 4.3885990192 -4.1902411326 0.198312665
H 5.1877709294 -0.38624987 -1.6899046201
C 1.4791363388 -0.4215433311 0.8851581644
C -1.1757976954 -0.4333464732 0.6548308884
C -1.9670850844 -1.536891926 0.3335535395
C 0.1949896106 -0.9346067759 0.9758745804
C -1.781553172 0.8644090621 0.4442114642
C -3.0230325487 0.9634217296 -0.2637436954
C -1.2480314476 2.0698390704 0.9492209941
H -0.4203790338 1.9990780713 1.6357514532
C 4.057146777 4.4921505304 -0.4505733363
C 5.9831351859 -2.9569291129 -1.5617694015
N 4.5860645863 5.4686781925 -0.7794268833
N 6.8825520619 -3.4285425968 -2.1187895024
C -1.7721777818 3.3056698771 0.644675144
H -1.3164877686 4.2059251042 1.0641666345
C -2.8859902154 3.4012501924 -0.2110595885
H -3.2724926819 4.376969051 -0.5134142346
C -3.5048634116 2.2468243201 -0.6341006188
H -4.3859646513 2.3073551313 -1.2733581213
C -3.7808674667 -0.2244735085 -0.5933242042
C -3.2359568565 -1.4492028999 -0.2623160603
H -3.7819462141 -2.3745078227 -0.4509574295
C 0.0394740818 -2.4364609855 1.225574278
H 0.7998354221 -2.9877387188 0.6662763644
C -1.3258114569 -2.8362021235 0.6730886601
H -1.9340325098 -3.3912083927 1.4054805783
H -1.2422975303 -3.4770804203 -0.2186071643
C 0.1994236789 -2.7476925549 2.7164744605
H -0.5843218472 -2.243360617 3.3041861852
H 0.1137159674 -3.8313539657 2.8907668801
H 1.1756282242 -2.4121903994 3.0979824009
N -5.0152254078 -0.0846390781 -1.225681134
C -5.6371647151 -1.2399101882 -1.8675349237
H -6.1828074344 -0.8686482088 -2.7506798156
H -4.8864554966 -1.9547282516 -2.2258273656
C -6.1148329038 0.5407357296 -0.4738161143
H -5.7378918069 1.2229030565 0.2970706831
H -6.7364845512 1.1249886538 -1.1719578707
C -6.9155817907 -0.6390373629 0.1114845821
H -7.9849183561 -0.3964983161 0.1900052313

H -6.5549997856 -0.8681120801 1.1252452791
C -6.6348710427 -1.8186497233 -0.8455216366
H -6.1932024837 -2.6675930908 -0.3032237189
H -7.5437394198 -2.1845028608 -1.3437001269

Energy= -1435.320175
Zero-point correction= 0.496607 (Hartree/Particle)
Thermal correction to Gibbs Free Energy= 0.438077
Sum of electronic and zero-point Energies= -1434.823568
Sum of electronic and thermal Energies= -1434.796121
Sum of electronic and thermal Enthalpies= -1434.795177
Sum of electronic and thermal Free Energies= -1434.882098
Imaginary Frequencies 1

Envelope-1-Inv-unstable-CAM-B3LYP

C -5.5498450889 -1.4357972141 0.488858445
C -4.664367153 -2.5129386242 0.6379956835
C -3.3064428254 -2.3457300065 0.422774115
C -2.8137507761 -1.0988635847 0.0304731683
C -3.7106976907 -0.0127339462 -0.0746388923
C -5.0690264069 -0.1718716512 0.1412153446
H -5.0541500437 -3.4805702844 0.9325060822
H -2.6434973771 -3.1888531255 0.5723103424
H -5.7558318461 0.6633268738 0.057640927
C -1.5706842264 0.8364614153 -0.4109371715
C -0.6320811114 1.7984494852 -0.7892606712
C -1.0504584961 3.0860624639 -1.0805990806
C -2.4083505436 3.4308607336 -1.0175290968
C -3.3640886917 2.4700290203 -0.6817295357
C -2.9396197041 1.1844848831 -0.3914564927
H 0.4188550056 1.5526091166 -0.86080568
H -0.3267812207 3.841430546 -1.3647563493
H -4.4156138564 2.7351577536 -0.6659511543
C -1.4373161465 -0.6096032003 -0.1599050484
C 1.0716075371 -0.9509748061 0.1491890499
C 1.9747938885 -1.880225805 -0.3431071689
C -0.2886329322 -1.362482228 -0.1687833139
C 1.5734291029 0.0950108812 1.0004627497
C 2.984854872 0.2595109737 1.1130136144
C 0.7413818215 0.8884825382 1.8231919977
H -0.3279715767 0.7385323186 1.7849081243
C 1.2642163009 1.8105085174 2.6946392704
H 0.5987880154 2.3935254291 3.32275883
C 2.6544759615 1.9757650745 2.8018500329
H 3.0659760895 2.6836525838 3.5133242613
C 3.4911342981 1.206269089 2.0348046533
H 4.5621469949 1.2803627539 2.1694185639
C 3.8860962387 -0.6062943409 0.3826350536
C 3.3589943725 -1.7145689963 -0.2571979079
H 4.0014682756 -2.4322224917 -0.7519844822
C -0.2251805033 -2.8194301106 -0.6459120265
H -0.4820697755 -3.4841118203 0.1895190128
C 1.2703968714 -3.030846896 -0.9889398623
H 1.4084190478 -3.014805374 -2.0766426399
H 1.6577961524 -3.9925527745 -0.6387884235
C -1.0982554351 -3.1518373745 -1.8573049771
H -0.8575585485 -2.4814375926 -2.6871180952
H -0.8982171083 -4.1761694665 -2.1852353438
H -2.1648681735 -3.0659699175 -1.6611567276
C -2.8155321112 4.7727882963 -1.3178762481
C -6.9523646925 -1.6322363178 0.7147886067
N -3.1409795157 5.8566654436 -1.5595843714
N -8.0837090248 -1.7933030042 0.8967629397
C 5.7245585335 0.9840552167 -0.0635034126
C 6.2259327505 -1.3033482989 -0.0569742371
H 6.3294823676 1.4739236176 0.7066033119
H 4.8747449985 1.6317084647 -0.2768182345

C 6.575817787 -0.8385728808 -1.4703500321
H 5.8497776819 -2.3219127651 0.0180175252
H 7.1106499034 -1.2382125582 0.5888296113
H 5.7933609449 -1.1538823452 -2.1669754169
H 7.5265170898 -1.2386166073 -1.8294246598
N 5.2493107024 -0.3278499501 0.4216956279
C 6.5862692564 0.693059233 -1.3226026294
H 7.6043080338 1.0621862673 -1.1753953176
H 6.1875559764 1.1922213392 -2.2078521834

Energy= -1436.0351778
Zero-point correction= 0.498879 (Hartree/Particle)
Thermal correction to Gibbs Free Energy= 0.439087
Sum of electronic and zero-point Energies= -1435.536299
Sum of electronic and thermal Energies= -1435.507998
Sum of electronic and thermal Enthalpies= -1435.507054
Sum of electronic and thermal Free Energies= -1435.596091
Imaginary Frequencies 0

Envelope-1-Inv-unstable-CAM-B3LYP-MeCN

C -5.5684343357 -1.4016254936 0.5272442544
C -4.6941950866 -2.4928944113 0.6749259106
C -3.3374752981 -2.3429799778 0.451949077
C -2.8293794773 -1.0977354204 0.0573828051
C -3.7212411723 -0.0018051178 -0.0548446363
C -5.0806060859 -0.1428948284 0.1683435269
H -5.0920324182 -3.4567103704 0.9725720728
H -2.6878218736 -3.1972731389 0.5947048991
H -5.7560304785 0.7012420058 0.0777372747
C -1.5759157464 0.8076752191 -0.4357875214
C -0.6283881286 1.7561499307 -0.8416545477
C -1.0326961572 3.0436899879 -1.1442571785
C -2.3886173391 3.4064451765 -1.0620499684
C -3.3560096719 2.4661916604 -0.6984064365
C -2.9431525423 1.1787261705 -0.3993601785
H 0.4180684096 1.496820379 -0.9321062521
H -0.3025510965 3.7836442692 -1.4527386711
H -4.4045547043 2.7427984893 -0.6650020456
C -1.4581411618 -0.6247705031 -0.154131688
C 1.0434722584 -0.9935771627 0.163378755
C 1.9473825893 -1.9579960214 -0.2873466425
C -0.3028970205 -1.3927419612 -0.1560638255
C 1.5619777169 0.0736071107 0.9828299924
C 2.975666616 0.2333069569 1.0794257862
C 0.7409557761 0.8827045141 1.7998923466
H -0.3300316853 0.7397601644 1.7695575998
C 1.2734062916 1.8052100011 2.6687368888
H 0.614194328 2.3971295725 3.2958966499
C 2.6638446046 1.9529642932 2.7746629532
H 3.0876853144 2.6551652878 3.4848217429
C 3.490436387 1.1731202163 2.0019686557
H 4.559880875 1.2469138238 2.1425114366
C 3.8725020377 -0.6513943485 0.3452001186
C 3.3254584058 -1.8089881945 -0.21064783
H 3.9516957708 -2.5627448503 -0.6686105915
C -0.2639347448 -2.8525167208 -0.6180610091
H -0.584532825 -3.4935870204 0.2122902146
C 1.2326927601 -3.1215564368 -0.8939805286
H 1.4145083202 -3.1667215359 -1.9737271443
H 1.5840926682 -4.0693274181 -0.4758659499
C -1.106369214 -3.1618809389 -1.8558444585
H -0.8076604421 -2.517064326 -2.6879361196
H -0.9405936122 -4.1994666177 -2.1608654117
H -2.175354281 -3.0306139486 -1.6932994916
C -2.7815700945 4.7463924341 -1.3730275226
C -6.9683191039 -1.5800070963 0.7623278712
N -3.0985813862 5.8318283985 -1.6242630407

N -8.1016088808 -1.7257276631 0.9523154029
C 5.7100959977 0.9995008437 -0.0057842825
C 6.2019621066 -1.301955399 -0.2482662906
H 6.2058181718 1.4282330711 0.8692350975
H 4.8749259998 1.6473858562 -0.2716191191
C 6.6546282391 -0.6516484433 -1.5489470443
H 5.8069441509 -2.3080840859 -0.3545729988
H 7.0367612578 -1.3493307621 0.4618152084
H 5.900722489 -0.8221514273 -2.3237054389
H 7.60947605 -1.0421414957 -1.9073416781
N 5.2093548892 -0.3635669244 0.2849016459
C 6.7208617202 0.8327490961 -1.1665974544
H 7.7253487429 1.0920932663 -0.8229183263
H 6.4790493897 1.4903058352 -2.0039068038

Energy= -1436.0764713
Zero-point correction= 0.497865 (Hartree/Particle)
Thermal correction to Gibbs Free Energy= 0.437869
Sum of electronic and zero-point Energies= -1435.578606
Sum of electronic and thermal Energies= -1435.550192
Sum of electronic and thermal Enthalpies= -1435.549248
Sum of electronic and thermal Free Energies= -1435.638603
Imaginary Frequencies 0

Envelope-1-Inv-unstable-wB97X-D

C 5.3852596672 -1.9633249574 -0.2351694504
C 4.4160335536 -2.971065477 -0.0768486365
C 3.0810617536 -2.645314409 0.1236339761
C 2.6959318012 -1.3001538961 0.1914398964
C 3.6727797737 -0.295851168 -0.0118301806
C 5.0095507331 -0.614241814 -0.2151034136
H 4.7228252541 -4.0172682329 -0.1203483041
H 2.3491945087 -3.4479223471 0.2158324098
H 5.7596335838 0.1637281251 -0.3688320776
C 1.6125783754 0.7785765225 0.1620912022
C 0.7577498963 1.8775588646 0.3111559908
C 1.2753802864 3.1637715667 0.2380087621
C 2.6517032377 3.3758694909 0.0355954425
C 3.5257223216 2.2863516287 -0.067676999
C 3.0013017418 1.002047153 0.0054865521
H -0.308340097 1.739398353 0.4869564093
H 0.613112231 4.0247587275 0.3409165205
H 4.5971762426 2.4547746381 -0.1906123423
C 1.3689338055 -0.6687216582 0.2882590345
C -1.1644351766 -0.8548313041 0.1100838698
C -2.1282964074 -1.5488163116 0.831925454
C 0.1639541263 -1.3000281362 0.5017395728
C -1.6003099222 0.0040528677 -0.9604322052
C -2.99370743 0.2938115279 -1.0715788718
C -0.7315612004 0.479666101 -1.9743788051
H 0.3249560238 0.2219634801 -1.9305168599
C -1.2022372169 1.2257584031 -3.0306228184
H -0.5090875559 1.5677905515 -3.8018380233
C -2.5757096237 1.5149857835 -3.1390030421
H -2.9519302173 2.0781297049 -3.9952147588
C -3.4492875622 1.0442620409 -2.1860927989
H -4.5190802216 1.2057207169 -2.3123795104
C -3.940637881 -0.2696815698 -0.1255598166
C -3.4952855075 -1.261927469 0.7409655113
H -4.1848121634 -1.7774225884 1.4093218623
C -0.0008221553 -2.57789916 1.3300967997
H 0.1932739387 -3.4513936335 0.6815738572
C -1.501332322 -2.5822388881 1.7141333969
H -1.6212336886 -2.3125929108 2.7770428048
H -1.9788543823 -3.5656740888 1.5843004559
C 0.8797301827 -2.6550919492 2.5782909411
H 0.7069668852 -1.7742057353 3.2153838397

H 0.6241367768 -3.5527003554 3.161741433
H 1.9514634863 -2.6949804453 2.3534952148
C 3.1627190791 4.7174750351 -0.045310385
C 6.7625406953 -2.322966676 -0.4385537184
N 3.5701928391 5.7991829675 -0.1116035361
N 7.870883867 -2.6154307183 -0.6011103148
C -5.5712758271 1.5870833648 -0.041046867
C -6.3111693582 -0.5512460722 0.5442560514
H -6.1689352655 1.9294929106 -0.901810821
H -4.6463853867 2.177371318 -0.0274829658
C -6.5477102583 0.2892078437 1.7989983791
H -6.0529384492 -1.5996372233 0.7286679876
H -7.2157285998 -0.5521326833 -0.0896181124
H -5.7728535014 0.0591667166 2.5470048119
H -7.5267981218 0.1066783832 2.2631711525
N -5.2578279147 0.1562114074 -0.1691241329
C -6.3869065629 1.726108998 1.2731284454
H -7.3664473207 2.1813556626 1.0667479096
H -5.8771973497 2.3744348234 1.9986424805

Energy= -1435.3159979
Zero-point correction= 0.497624 (Hartree/Particle)
Thermal correction to Gibbs Free Energy= 0.437957
Sum of electronic and zero-point Energies= -1434.818374
Sum of electronic and thermal Energies= -1434.790126
Sum of electronic and thermal Enthalpies= -1434.789182
Sum of electronic and thermal Free Energies= -1434.878041
Imaginary Frequencies 0

Envelope-2-CAM-B3LYP

C 2.4604520005 3.5300049687 -0.7024946774
C 1.1041102218 3.1818307262 -0.7259990545
C 0.7042853862 1.8704588273 -0.5192748857
C 1.6618536246 0.88751964 -0.2686495538
C 3.0264974904 1.242639724 -0.300540314
C 3.4343631021 2.5498138419 -0.50165382
H 0.3635951075 3.95105295 -0.9125961643
H -0.3475857224 1.6293090204 -0.5639069279
H 4.484905315 2.8180734095 -0.5214894193
C 2.9447095513 -1.0745425897 -0.0731436564
C 3.4873971958 -2.3503974196 0.088797805
C 4.863843089 -2.5157641579 0.1339169533
C 5.7212736037 -1.4138682418 0.035573657
C 5.1967406516 -0.1273974427 -0.1050321648
C 3.8228836 0.0278838875 -0.1558266591
H 2.8626497806 -3.2255955127 0.190639593
H 5.2868971729 -3.5067045425 0.2521448214
H 5.8640616495 0.7249804482 -0.1694030852
C 1.548040345 -0.5794445701 -0.1035052279
C -0.9860860266 -0.9346427761 0.087146381
C -1.7856263601 -1.8160394797 -0.6166470778
C 0.4160714459 -1.3380199015 -0.073614467
C -1.6047154952 0.0436703364 0.9298084849
C -3.018597713 0.1950863574 0.860460169
C -0.8914050089 0.7812581706 1.9046925084
H 0.179696902 0.6449373944 1.9813449833
C -1.5371013774 1.6313488351 2.7647389654
H -0.9695903042 2.1776130824 3.5108850194
C -2.936300939 1.768528845 2.7082282679
H -3.4467626534 2.4116908757 3.4171980524
C -3.6565625556 1.0582033555 1.7832373578
H -4.7384394113 1.1022098295 1.7855130226
C -3.7896397985 -0.600782639 -0.0649876554
C -3.1721408952 -1.6442882558 -0.7262636464
H -3.7426398901 -2.3147919512 -1.3569681676
C 0.3668129648 -2.8219891577 -0.4209343548
H 1.2158545548 -3.1054052203 -1.0446208621

C -0.9556243079 -2.913124174 -1.2231357098
H -1.4335615557 -3.8939939737 -1.1408717112
H -0.7712299221 -2.7267703376 -2.2875835647
C 0.3037633876 -3.718625126 0.8225945692
H -0.5936855836 -3.4966423952 1.4060567189
H 0.2686909933 -4.7724122083 0.5319639978
H 1.165642585 -3.5735263196 1.4777165866
C 7.1415618842 -1.6086384439 0.0879958079
C 2.8491422257 4.895445989 -0.9089018528
N 8.2867850052 -1.7675879455 0.1301647584
N 3.1605337803 5.9974290676 -1.0743476689
C -6.0120152944 -1.3216817637 -0.8509268296
C -5.5679790951 0.9543845314 -0.7732506114
C -7.3944646211 -0.6608137417 -0.8207166868
H -5.9711802088 -2.2756803829 -0.3197935905
H -5.702844924 -1.500557785 -1.8948614482
C -7.0978624176 0.8585719033 -0.8033544062
H -5.1656980671 1.0582374804 -1.7938565056
H -5.1960504111 1.7928131719 -0.1858941055
H -7.9995310734 -0.9631009137 -1.6777320335
H -7.9339278788 -0.9549187048 0.0818030295
H -7.5071355944 1.3761066143 -1.6732012932
H -7.5294792743 1.3239654786 0.0854169404
N -5.1560236059 -0.3371091088 -0.2019164714

Energy= -1436.043885
Zero-point correction= 0.499298 (Hartree/Particle)
Thermal correction to Gibbs Free Energy= 0.439998
Sum of electronic and zero-point Energies= -1435.544587
Sum of electronic and thermal Energies= -1435.516322
Sum of electronic and thermal Enthalpies= -1435.515378
Sum of electronic and thermal Free Energies= -1435.603887
Imaginary Frequencies 0

Envelope-2-CAM-B3LYP-MeCN

C 2.4459640042 3.5204207142 -0.7183413782
C 1.0878948726 3.1711926793 -0.7441578743
C 0.6932439593 1.859968686 -0.5314169325
C 1.6548730327 0.8793916942 -0.2740779099
C 3.0200567711 1.2410306215 -0.3048136874
C 3.4271120816 2.5476974041 -0.5110428181
H 0.3441693448 3.9359084192 -0.9378008456
H -0.3582228905 1.6180838562 -0.5828383358
H 4.4783955727 2.8141211846 -0.5289774844
C 2.9426691678 -1.0752393836 -0.0707041814
C 3.4889571045 -2.3525708617 0.0869237391
C 4.86516903 -2.5136531016 0.1311506523
C 5.7171133721 -1.4044092919 0.0347313421
C 5.1940731457 -0.1165922121 -0.1047423016
C 3.8192327461 0.0307518757 -0.1548068761
H 2.8684410408 -3.231195604 0.1842833254
H 5.2896136693 -3.5046439847 0.246185553
H 5.8545184833 0.7412730136 -0.1699397212
C 1.5471145804 -0.5844013141 -0.1014458128
C -0.9840945231 -0.9428817477 0.100737764
C -1.7897019568 -1.8295524323 -0.595125654
C 0.4123527634 -1.3479646592 -0.0685452299
C -1.6009321143 0.0423761993 0.9390820743
C -3.0149085405 0.2000095883 0.8665694712
C -0.8869134652 0.7774079627 1.9160113494
H 0.1833428135 0.6357398853 1.999238588
C -1.5306628101 1.6279190185 2.7793244488
H -0.9621757223 2.169402465 3.5288227289
C -2.9294079349 1.7692238633 2.7217493757
H -3.4399286259 2.4122473338 3.4312269305
C -3.6503578608 1.0628199992 1.7920508541
H -4.7316410695 1.1201207827 1.7945688343

C -3.7901640298 -0.5963460237 -0.0613784974
C -3.1744590682 -1.6536258122 -0.7081643836
H -3.7446281773 -2.3286918016 -1.3340383658
C 0.3652514967 -2.8317966966 -0.4129709885
H 1.2088034555 -3.1151746856 -1.0431633052
C -0.9665198548 -2.931722171 -1.1972629477
H -1.4451553093 -3.9100265904 -1.0979167793
H -0.7962274762 -2.7545789063 -2.2653273461
C 0.3206002076 -3.725818113 0.8331677917
H -0.5786913448 -3.5205073008 1.4209505131
H 0.3006004138 -4.7787415855 0.5380807138
H 1.1845340886 -3.5703205907 1.4837359452
C 7.1358129908 -1.5932060117 0.0854908494
C 2.8299587458 4.8833014697 -0.9326783964
N 8.2826748497 -1.746441866 0.1259805332
N 3.1396608084 5.9854484115 -1.105504612
C -5.994461651 -1.3034968588 -0.9004665362
C -5.5726589062 0.9771294132 -0.7414371245
C -7.3810701892 -0.6519375728 -0.8733608694
H -5.9568397592 -2.2722931303 -0.3969358844
H -5.6629786388 -1.4474268287 -1.94082489
C -7.0992768592 0.8666763538 -0.7799238966
H -5.168011297 1.1081200907 -1.7566318226
H -5.2153378406 1.8036108467 -0.1297623051
H -7.9577904711 -0.9192326945 -1.7610671245
H -7.9435384491 -0.9912299541 -0.0005960659
H -7.5098930013 1.423293552 -1.6247805414
H -7.5343890391 1.2830192032 0.131737414
N -5.1495391062 -0.3317752203 -0.2086889457

Energy= -1436.0830023
Zero-point correction= 0.498464 (Hartree/Particle)
Thermal correction to Gibbs Free Energy= 0.439109
Sum of electronic and zero-point Energies= -1435.584538
Sum of electronic and thermal Energies= -1435.556266
Sum of electronic and thermal Enthalpies= -1435.555322
Sum of electronic and thermal Free Energies= -1435.643893
Imaginary Frequencies 0

Envelope-2-wB97X-D

C 2.4023965889 3.5287743983 -0.6827792941
C 1.0499091313 3.1537080544 -0.7547096137
C 0.6683623715 1.8292471251 -0.5754558788
C 1.6396332045 0.8603113345 -0.3018860717
C 3.0007802651 1.2395987745 -0.2911025859
C 3.3912089647 2.5608261497 -0.4650096024
H 0.2946278645 3.914324589 -0.9579882418
H -0.383720104 1.566142612 -0.6612004581
H 4.4440181563 2.8482856138 -0.4500054139
C 2.9483049683 -1.0860943899 -0.0918948384
C 3.5067715818 -2.3608267375 0.0592658419
C 4.8879169315 -2.5078599111 0.132615062
C 5.7346544265 -1.389149621 0.0713565732
C 5.1926092115 -0.1043714882 -0.0620649021
C 3.8136017224 0.0327925864 -0.1414124196
H 2.8889874194 -3.253082219 0.1303784435
H 5.3237932019 -3.5019104646 0.2435233107
H 5.8510173897 0.7652842969 -0.1002691238
C 1.5461904678 -0.6083382622 -0.1420406432
C -0.9819984795 -0.9698757211 0.0661591934
C -1.7911966287 -1.8593400476 -0.6228357829
C 0.4165909018 -1.3762047485 -0.1070814334
C -1.5868268683 0.0235659501 0.9031838068
C -3.0008980029 0.192293476 0.8322323371
C -0.8596308626 0.7626084291 1.8716450228
H 0.2174540049 0.6142702225 1.9502644029
C -1.4948784467 1.6339238798 2.7249142192

H -0.9150473079 2.1856361755 3.4676373245
C -2.8961330304 1.7857710668 2.6706346362
H -3.4014558498 2.4445965456 3.379614252
C -3.6293756327 1.070288223 1.7530914409
H -4.7180958752 1.1285157723 1.7601763568
C -3.7846960458 -0.606337753 -0.0874312012
C -3.1796721017 -1.6766449604 -0.7301278678
H -3.7614080931 -2.3554304493 -1.3538389846
C 0.3640756856 -2.8635232737 -0.4293288022
H 1.2112167523 -3.1582107384 -1.0628722072
C -0.9661810781 -2.967932642 -1.2157945693
H -1.4492040259 -3.9519117259 -1.1143964818
H -0.7925986369 -2.7946033224 -2.2910230185
C 0.3254433013 -3.7304994562 0.8353138353
H -0.570940922 -3.5004981308 1.4314365677
H 0.2977027136 -4.7981159664 0.5702916869
H 1.2014067974 -3.5565105269 1.4765252134
C 7.1599402494 -1.5643024389 0.152776263
C 2.7726743432 4.9074652525 -0.8587960126
N 8.30667359 -1.707874796 0.2184061981
N 3.0676469479 6.0180433172 -0.9990585512
C -6.023394906 -1.2931959477 -0.8545788289
C -5.5497253527 0.9761868334 -0.7814296334
C -7.3964049649 -0.6075956156 -0.81446359
H -5.9910802155 -2.246127481 -0.3063159729
H -5.7328235466 -1.5000577237 -1.906448587
C -7.0776738461 0.9040283136 -0.7338745699
H -5.1938364559 1.0774728784 -1.8276117786
H -5.1317219528 1.8154377251 -0.2125482753
H -7.9959534949 -0.8671755154 -1.6976696027
H -7.9621074524 -0.9320428755 0.0697123293
H -7.5330847385 1.4804247819 -1.550700882
H -7.4482073031 1.3270720636 0.2107175534
N -5.1372089331 -0.3206254915 -0.2415421495

Energy= -1435.3238817
Zero-point correction= 0.497710 (Hartree/Particle)
Thermal correction to Gibbs Free Energy= 0.438336
Sum of electronic and zero-point Energies= -1434.826172
Sum of electronic and thermal Energies= -1434.797893
Sum of electronic and thermal Enthalpies= -1434.796948
Sum of electronic and thermal Free Energies= -1434.885546
Imaginary Frequencies 0

Envelope-2-wB97X-D-MeCN

C 2.3804359954 3.51673224 -0.7027689276
C 1.0266762964 3.1384649033 -0.7734830462
C 0.6521307952 1.8140081181 -0.5851920489
C 1.6291238634 0.8488913989 -0.3057494046
C 2.9902708708 1.2368295368 -0.2971712938
C 3.3778489712 2.5580627771 -0.4786634087
H 0.2666878509 3.8929484036 -0.9837293414
H -0.3992494788 1.5487151206 -0.6748328487
H 4.4310991161 2.8448742586 -0.4642438141
C 2.9452219362 -1.0876658829 -0.0882676439
C 3.5095177415 -2.3631112671 0.0570473035
C 4.890713545 -2.5038799122 0.1257274808
C 5.7303763791 -1.3769910416 0.0636496228
C 5.1879801925 -0.0916089927 -0.0675991864
C 3.8075681073 0.0358942093 -0.1424927649
H 2.8977097387 -3.2597995368 0.1247365145
H 5.3294568292 -3.4974836804 0.2323333522
H 5.8380374646 0.7843975749 -0.1090721251
C 1.5437950473 -0.6161134771 -0.1366532697
C -0.9801664009 -0.9828599728 0.0863608583
C -1.796918938 -1.8803423153 -0.5905040275
C 0.4115457783 -1.3903880332 -0.0967747142

C -1.5831278497 0.020592822 0.915409958
C -2.9969197179 0.196906993 0.8394184753
C -0.8554283894 0.7576326366 1.8854575597
H 0.2208007202 0.6037591892 1.9706736114
C -1.4890450149 1.6292562962 2.7420896715
H -0.9086019355 2.1764423466 3.4885079296
C -2.8897503837 1.7840320692 2.6870775165
H -3.3957904251 2.4416940942 3.397230211
C -3.6233774104 1.0731032772 1.7637760732
H -4.7113446098 1.1441489251 1.7726034843
C -3.784806529 -0.6019969826 -0.0844607946
C -3.1827344253 -1.6928454665 -0.7035532494
H -3.7636008752 -2.3782541679 -1.3206862932
C 0.3624531521 -2.8780336372 -0.4133129273
H 1.2026882104 -3.1733447127 -1.054797879
C -0.9794625336 -2.9957399126 -1.1760413457
H -1.4620265653 -3.9766721811 -1.0516481534
H -0.8244313223 -2.8370611498 -2.256091283
C 0.3483686927 -3.7393281517 0.8557716973
H -0.5482462315 -3.527047733 1.4590162964
H 0.3374343212 -4.8066146493 0.5884756052
H 1.227993843 -3.5521437913 1.488736192
C 7.154541087 -1.5447852235 0.1390388561
C 2.744145345 4.8931258204 -0.8905611728
N 8.3031757814 -1.6818707402 0.1995253911
N 3.0356910483 6.0040547095 -1.0414359465
C -6.0011151741 -1.2664193647 -0.92297606
C -5.5509835698 1.0089313468 -0.7424918292
C -7.3776725774 -0.5863684335 -0.894632605
H -5.9792681127 -2.2347959569 -0.4024089979
H -5.6806184074 -1.4392319552 -1.9698571904
C -7.0751029609 0.9167319125 -0.6991930907
H -5.1962071703 1.150802614 -1.7828441934
H -5.1471447871 1.8299523009 -0.139583512
H -7.9296748783 -0.7861298685 -1.8233114796
H -7.9840723733 -0.9726611399 -0.0634017793
H -7.5365023608 1.5497997124 -1.4695246243
H -7.4435253581 1.2613826151 0.2781271189
N -5.1249453236 -0.3103673117 -0.2606363873

Energy= -1435.363147
Zero-point correction= 0.496793 (Hartree/Particle)
Thermal correction to Gibbs Free Energy= 0.437412
Sum of electronic and zero-point Energies= -1434.866354
Sum of electronic and thermal Energies= -1434.838069
Sum of electronic and thermal Enthalpies= -1434.837124
Sum of electronic and thermal Free Energies= -1434.925735
Imaginary Frequencies 0

Envelope-2-TS-CAM-B3LYP

C 3.2376644504 3.3642620265 -0.2672651265
C 2.3949452962 3.397409136 0.8479932216
C 1.806168257 2.2327263098 1.3208269637
C 2.0027227022 1.0233070186 0.6587155195
C 3.001418071 0.9865740209 -0.3377594314
C 3.5825269098 2.1371388337 -0.8394447431
H 2.2261573992 4.3379939684 1.3593453116
H 1.2612610812 2.2754829482 2.2526938328
H 4.3319545376 2.0967059349 -1.6220793284
C 2.697338194 -1.1971849399 0.4227936032
C 3.1406376372 -2.5067350221 0.6267015897
C 4.1772428297 -3.026002153 -0.1402844159
C 4.8037943697 -2.2528030301 -1.1190364719
C 4.433164898 -0.9156272243 -1.2861052767
C 3.4172824671 -0.4027549869 -0.5032330996
H 2.7429730317 -3.1297000054 1.4138160705
H 4.5164829595 -4.0409078967 0.0325120413

H 4.9572261511 -0.2941144456 -2.0037791435
C 1.5646261512 -0.3679874291 0.9594395019
C -1.0913577149 -0.4900336101 1.0510223961
C -1.8675903459 -1.6326026092 0.9121617597
C 0.3284676868 -0.9162565088 1.2327258447
C -1.7872163484 0.7662519259 0.8579642258
C -3.1013514494 0.7638809867 0.3003223706
C -1.2973165974 2.0028391084 1.311748839
H -0.3592860301 2.0085145664 1.8252586858
C 3.8173264935 4.5789206711 -0.7632748057
C 5.8555503565 -2.8156486026 -1.9151833486
N 4.2805256632 5.5601875978 -1.1646268823
N 6.7014045306 -3.2720508029 -2.5592498122
C -1.9973986495 3.1763161527 1.1827301398
H -1.5719616918 4.1008789934 1.5594572352
C -3.2766015629 3.1694828423 0.6111827848
H -3.8517503154 4.0863141511 0.5384842254
C -3.8168725861 1.978084948 0.1994578726
H -4.840264597 1.9390298026 -0.151371214
C -3.7506753237 -0.4764735777 -0.0464139234
C -3.1646466342 -1.6442304045 0.3738157546
H -3.6503283463 -2.6000599016 0.2202510733
C 0.2767199497 -2.4088916998 1.5733200452
H 0.9484777649 -2.952247607 0.9133578052
C -1.1477123812 -2.8818827386 1.2854883821
H -1.6134060756 -3.3478015462 2.1618491985
H -1.184125915 -3.6239483995 0.4811301949
C 0.694526107 -2.6480835782 3.0279873006
H 0.0027542005 -2.1440776162 3.7097359227
H 0.6784889751 -3.7168170828 3.2628358715
H 1.6965599268 -2.2631706114 3.231113818
C -5.0158982486 0.1008411222 -2.0820497023
C -5.7846644039 -1.6556824344 -0.7743039294
C -6.4837873389 -0.0702428871 -2.4883429494
H -4.3591793468 -0.489311863 -2.7410879645
H -4.6721123006 1.1337500868 -2.1144869538
C -7.0026931728 -1.2282678056 -1.6013234547
H -6.0371762971 -1.9967474541 0.2325725827
H -5.249628225 -2.4739391908 -1.2855441516
H -7.0452383813 0.8455162608 -2.2907446604
H -6.5748309279 -0.2845217176 -3.5550054218
H -7.8020844662 -0.8784715732 -0.9450663749
H -7.3991181389 -2.0614570535 -2.1849608663
N -4.9754295958 -0.4452381437 -0.7165725121

Energy= -1436.0036931
Zero-point correction= 0.498913 (Hartree/Particle)
Thermal correction to Gibbs Free Energy= 0.440592
Sum of electronic and zero-point Energies= -1435.504780
Sum of electronic and thermal Energies= -1435.477235
Sum of electronic and thermal Enthalpies= -1435.476291
Sum of electronic and thermal Free Energies= -1435.563101
Imaginary Frequencies 1

Envelope-2-TS-CAM-B3LYP-MeCN

C 3.2168142804 3.3597791514 -0.2516770276
C 2.3719626164 3.3922021554 0.8648566914
C 1.7868389108 2.2253689798 1.3342646402
C 1.9895707841 1.0166360791 0.6679244675
C 2.9853393471 0.9863109767 -0.3337523277
C 3.5668883976 2.1380316064 -0.8332604866
H 2.1982072342 4.3302860509 1.3796725803
H 1.2413328377 2.2655009013 2.265027162
H 4.3125674821 2.0967328722 -1.6199120269
C 2.6633060636 -1.2060576696 0.3951610377
C 3.0851993567 -2.5311557381 0.5597740512
C 4.1037991874 -3.0474986413 -0.2309842485

C 4.7352811801 -2.2550757632 -1.1943216548
C 4.3891828865 -0.9072743127 -1.3281003381
C 3.3875456255 -0.4018064446 -0.5221377087
H 2.6866475094 -3.1730928421 1.3304395237
H 4.4235481669 -4.0735292038 -0.0879067492
H 4.9127062273 -0.2714230146 -2.0338695001
C 1.5549267254 -0.37286838 0.9667707
C -1.0930167352 -0.4753700563 1.1105450608
C -1.8834988091 -1.6148525192 0.9979635466
C 0.31868406 -0.914124821 1.2777347792
C -1.7804753717 0.7834668541 0.8902204643
C -3.0785554155 0.7789653869 0.2935534929
C -1.3087022092 2.0189683464 1.3695907133
H -0.3993394815 2.0211555184 1.9351186272
C 3.7952002505 4.5749416105 -0.7409984322
C 5.7703963804 -2.8130764745 -2.010983982
N 4.258983696 5.5582497179 -1.1389150917
N 6.6054865353 -3.265485063 -2.6731264691
C -2.0028517495 3.1947477193 1.2139980715
H -1.5943986045 4.1172234206 1.6146838662
C -3.2570610794 3.1892303943 0.5886587505
H -3.8276862882 4.1068424611 0.4899326928
C -3.7853582536 1.9959310516 0.1611471736
H -4.7944796902 1.9668415341 -0.2296783754
C -3.7309814904 -0.4682020012 -0.0428361375
C -3.1708875367 -1.6290531967 0.4381735007
H -3.6642589932 -2.5841325854 0.3075425673
C 0.2621218314 -2.4016958547 1.6354446712
H 0.8846602867 -2.9615603021 0.9414813292
C -1.1816585916 -2.8552703257 1.4251599907
H -1.6295518238 -3.2430173886 2.3476018838
H -1.2661924789 -3.6488983567 0.6768285849
C 0.7563569466 -2.6416524236 3.0649006326
H 0.11629297 -2.1180789732 3.7822088736
H 0.7252693533 -3.7094515902 3.3021260518
H 1.7798857339 -2.2869145876 3.2090872669
C -4.9663203346 0.1030948083 -2.1072342153
C -5.7270041791 -1.6764840027 -0.8170970536
C -6.4271567601 -0.0799735549 -2.5245981352
H -4.3005254317 -0.4838835907 -2.7580735146
H -4.634565503 1.1394042523 -2.1365897298
C -6.9311574512 -1.271199914 -1.6752317804
H -5.999444885 -2.0192498061 0.183818056
H -5.1624686146 -2.4829089614 -1.3106024754
H -7.0015496639 0.8218907898 -2.2995768325
H -6.5082819723 -0.2635318493 -3.597857728
H -7.7659383628 -0.964205259 -1.041211991
H -7.2738474836 -2.1077792754 -2.2874044321
N -4.9331019791 -0.451532635 -0.7412570377

Energy= -1436.0447386
Zero-point correction= 0.498164 (Hartree/Particle)
Thermal correction to Gibbs Free Energy= 0.439880
Sum of electronic and zero-point Energies= -1435.546574
Sum of electronic and thermal Energies= -1435.519038
Sum of electronic and thermal Enthalpies= -1435.518094
Sum of electronic and thermal Free Energies= -1435.604858
Imaginary Frequencies 1

Envelope-2-TS-wB97X-D

C 3.1765221577 3.3303748006 -0.2915006755
C 2.3374388728 3.3515333022 0.8332674222
C 1.7615009958 2.17689951 1.3066644818
C 1.9656283736 0.97023456 0.6341916392
C 2.9621975421 0.9432694989 -0.3680468625
C 3.5316410446 2.1046438556 -0.8709196663
H 2.160832495 4.2944909138 1.3529061081

H 1.2164968789 2.2083014482 2.2472000214
H 4.2811722667 2.071813029 -1.6636043575
C 2.6663427009 -1.2486941583 0.3866384623
C 3.1050838764 -2.5657324759 0.5781818269
C 4.1388906794 -3.083352953 -0.2000928889
C 4.7672864168 -2.301297721 -1.1776681088
C 4.3977762548 -0.9579041748 -1.333480408
C 3.3836659006 -0.44646495 -0.5396229652
H 2.7022468105 -3.1997551269 1.3637620326
H 4.4758425814 -4.1081325079 -0.0362329215
H 4.9210878295 -0.326920778 -2.0541112204
C 1.5372033998 -0.4230831074 0.930855523
C -1.1112045757 -0.5383834121 0.9636169681
C -1.8931859996 -1.6797814976 0.8091057733
C 0.294974251 -0.9734761965 1.1890661831
C -1.7803125052 0.7236128872 0.715890345
C -3.0558386391 0.7270817796 0.070691805
C -1.3087593011 1.9602005616 1.1988186652
H -0.4081137754 1.9570182038 1.7899591089
C 3.7403200813 4.5554769746 -0.7908709898
C 5.8158609631 -2.8620974615 -1.9861292617
N 4.189270517 5.543391703 -1.1938308229
N 6.6573418473 -3.3163479516 -2.638459693
C -1.987480562 3.143450026 1.013134549
H -1.5780585856 4.0720174746 1.4168300797
C -3.2274603724 3.1435700244 0.3527010421
H -3.7916292483 4.0706687015 0.2343480506
C -3.7541660049 1.949850431 -0.0849351342
H -4.7595365891 1.9209702628 -0.5052977271
C -3.6978234473 -0.5165775194 -0.2979314714
C -3.160274143 -1.6868722165 0.1959143084
H -3.6525126505 -2.6457391023 0.0322511861
C 0.2297552104 -2.4623952072 1.5362166673
H 0.8962291506 -3.0138939062 0.8639367011
C -1.1998447317 -2.9232319979 1.2498034782
H -1.6924192226 -3.3341548502 2.147149731
H -1.2432375628 -3.708670337 0.479493418
C 0.664438704 -2.7049893132 2.9840740999
H -0.0166781417 -2.1901305387 3.6796719511
H 0.6442224172 -3.7801891752 3.2207595868
H 1.679221792 -2.3250412019 3.1725635565
C -4.8300702261 0.0712249705 -2.4124829966
C -5.6991081038 -1.6692082738 -1.1463232697
C -6.2878851292 -0.0373078404 -2.863861064
H -4.1713854567 -0.5439229985 -3.0594466721
H -4.438728122 1.0954778684 -2.4303376808
C -6.8562613101 -1.2261673003 -2.0535799975
H -6.022488327 -1.9875781842 -0.14444714
H -5.1554343358 -2.5194182289 -1.6099885285
H -6.8290148935 0.8885601342 -2.6218171518
H -6.3632636579 -0.1855344244 -3.9497461159
H -7.7182625016 -0.9084125214 -1.4510828387
H -7.1947431269 -2.0511662297 -2.6952488692
N -4.8582865727 -0.4890911014 -1.0600853923

Energy= -1435.2825434
Zero-point correction= 0.497409 (Hartree/Particle)
Thermal correction to Gibbs Free Energy= 0.439200
Sum of electronic and zero-point Energies= -1434.785134
Sum of electronic and thermal Energies= -1434.757619
Sum of electronic and thermal Enthalpies= -1434.756675
Sum of electronic and thermal Free Energies= -1434.843343
Imaginary Frequencies 1

Envelope-2-TS-wB97X-D-MeCN

C 3.1244459129 3.3667937112 -0.2674388283
C 2.3162127561 3.3903097103 0.8827205077

C 1.7615949707 2.2146561778 1.3753417626
C 1.9558370945 1.0050390106 0.6997478535
C 2.9169795937 0.9829414741 -0.3390760908
C 3.4689473534 2.1448057068 -0.8617198289
H 2.1467406994 4.3326863787 1.4064643241
H 1.2442390059 2.247471569 2.3301652395
H 4.1893850301 2.1088495019 -1.6813094727
C 2.62325148 -1.2168256165 0.3944905737
C 3.040228634 -2.5516392087 0.5279498705
C 4.0262256493 -3.0673836093 -0.3090192077
C 4.6310651099 -2.2659114806 -1.2893289915
C 4.2869872731 -0.9110005777 -1.3969442397
C 3.316694888 -0.405613218 -0.545771256
H 2.6594427736 -3.2067503909 1.3067856146
H 4.3428693814 -4.1048805695 -0.1886052786
H 4.7878037896 -0.2657185379 -2.1212751052
C 1.5437211474 -0.3851389118 1.0144762766
C -1.0909384108 -0.4789894396 1.2057485671
C -1.8930288482 -1.6185396583 1.1138478235
C 0.3122505561 -0.9261112027 1.364212843
C -1.762192419 0.7822205527 0.9454483718
C -3.0377718909 0.7748033029 0.2981893179
C -1.3033424888 2.0206822233 1.4393293144
H -0.4174974591 2.0206347258 2.0555880691
C 3.669824599 4.591391432 -0.7816764636
C 5.6326117302 -2.8228052656 -2.1538895211
N 4.1056066213 5.5805722399 -1.1981329257
N 6.4388064676 -3.2740550472 -2.8527364477
C -1.9884924747 3.2015776846 1.2475571025
H -1.5914934583 4.1311369507 1.6623854249
C -3.2208659494 3.1939627947 0.5730629822
H -3.7901202769 4.1175067693 0.4473176133
C -3.7397988817 1.9947092804 0.13469342
H -4.7426020144 1.9700621696 -0.2919744609
C -3.6872228397 -0.480958286 -0.0395312311
C -3.166155115 -1.6365086121 0.5170806292
H -3.668812363 -2.5964623829 0.3990568107
C 0.2553680752 -2.4065071853 1.742432764
H 0.8374639233 -2.9808125298 1.0130388816
C -1.2020240739 -2.8445715265 1.5988450496
H -1.6371480535 -3.1566667201 2.5632651577
H -1.3260798828 -3.6901561542 0.9063808525
C 0.8279588019 -2.6437849501 3.1412287652
H 0.2356076446 -2.1016330513 3.895548248
H 0.7974362002 -3.7161419414 3.3888108468
H 1.8701354529 -2.3005589323 3.2214223357
C -4.8523769126 0.109282204 -2.1492802705
C -5.6520107145 -1.6839542118 -0.8928267776
C -6.3172946591 -0.0199185375 -2.5574619137
H -4.2032478783 -0.4860084223 -2.8216913872
H -4.4847399475 1.1414140114 -2.1613230208
C -6.814093467 -1.2801269872 -1.8141959911
H -5.9765611458 -1.9999608645 0.1092428633
H -5.0794197073 -2.5208379322 -1.3396989456
H -6.8752280316 0.8685343383 -2.2268355332
H -6.4267416682 -0.0944942174 -3.6480677441
H -7.7178845753 -1.0586004188 -1.2296601137
H -7.0630641014 -2.0987590286 -2.5033410199
N -4.8328072664 -0.4828230332 -0.8068479896

Energy= -1435.3240398
Zero-point correction= 0.496471 (Hartree/Particle)
Thermal correction to Gibbs Free Energy= 0.437929
Sum of electronic and zero-point Energies= -1434.827568
Sum of electronic and thermal Energies= -1434.800021
Sum of electronic and thermal Enthalpies= -1434.799077
Sum of electronic and thermal Free Energies= -1434.886110
Imaginary Frequencies 1

Envelope-2-unstable-CAM-B3LYP

C -5.4540156922 -1.6504604876 0.8586964378
C -4.4954817097 -2.6716769256 0.9243903483
C -3.1803820342 -2.4289228326 0.5629338278
C -2.8101036291 -1.163350453 0.1041738691
C -3.7739706175 -0.1320062115 0.0869515279
C -5.0895845799 -0.3660520431 0.4492036166
H -4.7932104324 -3.6547071373 1.2706912028
H -2.4535527643 -3.2269923578 0.6523442923
H -5.8298272076 0.426383973 0.4331595387
C -1.7333930682 0.8359471159 -0.4758372802
C -0.896593417 1.842454889 -0.9601156769
C -1.4175388521 3.0999632575 -1.2172527914
C -2.7790904704 3.3670013966 -1.015139704
C -3.6376519469 2.358301766 -0.5733273159
C -3.1107129582 1.1038207634 -0.3175725226
H 0.1534834851 1.6553580665 -1.1407865698
H -0.7724836536 3.8907822316 -1.5825347339
H -4.6956700067 2.5629868309 -0.4513623381
C -1.4908022008 -0.599032155 -0.2330324434
C 1.0537912855 -0.7982849005 -0.1810186476
C 1.9440411668 -1.6564179881 -0.8045697769
C -0.3116480409 -1.286483648 -0.3645813025
C 1.5756840089 0.2717725376 0.6246615718
C 2.9793814055 0.5172025822 0.5984479699
C 0.7799143519 1.0690904306 1.481231442
H -0.2695466091 0.8343397202 1.5894477135
C 1.3143994874 2.1230623958 2.1771679205
H 0.6800136276 2.7139201886 2.8295283714
C 2.6723163318 2.4570571673 2.0256811187
H 3.0757037692 3.3342827671 2.5202052088
C 3.4840253448 1.6633713253 1.2584289461
H 4.5280408903 1.9160961953 1.1235333973
C 3.8625395367 -0.3875181632 -0.0933789808
C 3.3279545655 -1.4555511431 -0.7844881487
H 3.9747991406 -2.1679776783 -1.2819824709
C -0.2102697023 -2.7338009763 -0.8653498634
H -0.2868140511 -3.4183421766 -0.0096069954
C 1.2336326994 -2.8197098027 -1.4204754505
H 1.2143226431 -2.7304036207 -2.5134325944
H 1.7234181594 -3.7708427789 -1.1914626108
C -1.2139148949 -3.1398142924 -1.9453062611
H -1.1622791924 -2.4425965988 -2.7861567822
H -0.9638866107 -4.1363717372 -2.3209342752
H -2.2444790737 -3.163818651 -1.5982775237
C -3.2937671528 4.6788349961 -1.2825764625
C -6.8103817321 -1.9242196937 1.2361964908
N -3.7070662444 5.7380862375 -1.4973462571
N -7.9041819817 -2.1475874486 1.5404086692
C 6.1128837096 -0.7987586934 -1.0181898244
C 5.9374535814 -0.358419258 1.2500287805
C 7.5043657369 -0.299119459 -0.6196928459
H 5.8153854962 -0.5176760773 -2.0310525851
H 6.0785114611 -1.8991626858 -0.9453053587
C 7.40711327 -0.0767575805 0.9093996452
H 5.8109464012 -1.3995295267 1.5883226699
H 5.5382532149 0.2903278278 2.0282130502
H 8.2825079296 -1.0135399443 -0.8955635257
H 7.724611806 0.6415395204 -1.1284316779
H 8.0717254116 -0.7364964705 1.4708139672
H 7.6744670073 0.9501739376 1.1673950948
N 5.2443365316 -0.1642630627 -0.0338240918

Energy= -1436.0368386
Zero-point correction= 0.498817 (Hartree/Particle)
Thermal correction to Gibbs Free Energy= 0.439358
Sum of electronic and zero-point Energies= -1435.538021

Sum of electronic and thermal Energies= -1435.509725
Sum of electronic and thermal Enthalpies= -1435.508781
Sum of electronic and thermal Free Energies= -1435.597481
Imaginary Frequencies 0

Envelope-2-unstable-CAM-B3LYP-MeCN

C -5.435099382 -1.6651983963 0.8805746896
C -4.4737503638 -2.688717513 0.9232671159
C -3.16452031 -2.4387430856 0.550191014
C -2.8024637094 -1.162378019 0.1056031549
C -3.7735267814 -0.1337108553 0.1071552704
C -5.0851532674 -0.3730570008 0.4808306158
H -4.7637665523 -3.6781520598 1.2586797664
H -2.4376323503 -3.2387154989 0.6173998716
H -5.8255713289 0.4198161433 0.4767694277
C -1.744481324 0.8384448391 -0.4847097791
C -0.9169722483 1.851483279 -0.9799274511
C -1.4440381233 3.1078023793 -1.2243956287
C -2.8055227967 3.3674962356 -0.9955967252
C -3.6584652411 2.357644763 -0.5428921032
C -3.1222135878 1.1041344007 -0.3023551424
H 0.1297903828 1.6687862468 -1.1840231685
H -0.8065409269 3.9004583573 -1.5998121074
H -4.7152583383 2.5551961336 -0.3985039841
C -1.4942534819 -0.5900498945 -0.2440915469
C 1.0516087259 -0.7895290559 -0.1936734022
C 1.9461971932 -1.6530687364 -0.812840224
C -0.3064568889 -1.2751971717 -0.3878491566
C 1.5750028897 0.2832891893 0.611391424
C 2.9811339733 0.5208820512 0.5959839971
C 0.7777632727 1.0891981121 1.458702204
H -0.2725658035 0.8574523014 1.5673495237
C 1.3108129441 2.1473821197 2.1525496773
H 0.674300451 2.7433242223 2.7989174707
C 2.6699791753 2.4765985236 2.0059183452
H 3.0750769298 3.3556155938 2.4963536174
C 3.4838700375 1.6716207306 1.2497511275
H 4.5296355322 1.9238106036 1.1287246783
C 3.8683748883 -0.394174893 -0.0871263617
C 3.3291719238 -1.4580880113 -0.7866613764
H 3.9724645488 -2.175998485 -1.2800448754
C -0.2103531354 -2.714830733 -0.9036032195
H -0.3156378025 -3.4029346525 -0.0549908043
C 1.2402040871 -2.8148135549 -1.4312722187
H 1.244643418 -2.7299406531 -2.524405149
H 1.7228744376 -3.764502635 -1.1847888877
C -1.2036733271 -3.0955591938 -2.0007818737
H -1.1323326441 -2.3937750296 -2.8371390467
H -0.9589527496 -4.0924100493 -2.379618448
H -2.2387773852 -3.1160763719 -1.6639581653
C -3.3275048297 4.67603373 -1.2486214597
C -6.7835450449 -1.9467144549 1.2697708309
N -3.748461558 5.7353776881 -1.4528437488
N -7.8744105198 -2.1753068197 1.5840392864
C 6.1154481477 -0.8780244522 -0.975150479
C 5.9422143878 -0.3179562187 1.2700997166
C 7.5095203017 -0.3709921029 -0.5984811863
H 5.8239180951 -0.6467934452 -2.0021507322
H 6.0655695815 -1.9698870502 -0.8415466138
C 7.410386919 -0.0586748313 0.9137881518
H 5.8115101116 -1.3428343664 1.649250853
H 5.5525808536 0.367360791 2.0200336297
H 8.2769608648 -1.1129094998 -0.8280017009
H 7.745797938 0.5352547053 -1.1609642962
H 8.0712416933 -0.6865743215 1.51487583
H 7.676032475 0.9824060553 1.1114638559
N 5.2455385522 -0.1850866227 -0.0249230831

Energy= -1436.0766911
Zero-point correction= 0.497834 (Hartree/Particle)
Thermal correction to Gibbs Free Energy= 0.438233
Sum of electronic and zero-point Energies= -1435.578857
Sum of electronic and thermal Energies= -1435.550524
Sum of electronic and thermal Enthalpies= -1435.549580
Sum of electronic and thermal Free Energies= -1435.638458
Imaginary Frequencies 0

Envelope-2-unstable-wB97X-D

C 5.330396078 2.2853090501 0.4749501395
C 4.2822658981 3.2232500172 0.4408934637
C 2.9850768764 2.8249228985 0.1442861632
C 2.7218125098 1.4814266849 -0.1485345547
C 3.7740240814 0.5392905794 -0.0638187805
C 5.0733255645 0.9294235363 0.2346053541
H 4.4967564371 4.2705949653 0.6589695197
H 2.1861948191 3.5668919149 0.1560079957
H 5.8836602345 0.2007293109 0.2961814467
C 1.8147934141 -0.6591971471 -0.4618214944
C 1.0575213757 -1.7857782573 -0.8028833038
C 1.6794747456 -3.0231304323 -0.9058397608
C 3.0640764648 -3.1527288192 -0.6926059459
C 3.8422880888 -2.0255144288 -0.3996305132
C 3.2135774394 -0.7911968365 -0.2965237278
H -0.0126412178 -1.7082505975 -0.9915043526
H 1.093298852 -3.9081740141 -1.1578014607
H 4.9214949231 -2.1247726173 -0.2697102538
C 1.4538413156 0.7687119435 -0.384204562
C -1.0910776024 0.7299577039 -0.2660735552
C -2.0812418016 1.4089743862 -0.9629689583
C 0.2096632469 1.3280932065 -0.5543069103
C -1.4729274035 -0.2933041399 0.6703984725
C -2.8385012376 -0.7077536634 0.699352939
C -0.5747612483 -0.8937606701 1.5899187592
H 0.4443294191 -0.5167803877 1.6581590831
C -0.9666089565 -1.9400048737 2.3922463683
H -0.2533595085 -2.3834819608 3.08988934
C -2.2738706584 -2.4595189701 2.2929749614
H -2.5596501316 -3.3393928374 2.8727414058
C -3.1894643942 -1.8461538479 1.47095963
H -4.2001449583 -2.2446872471 1.3813986588
C -3.83481649 0.0149396074 -0.0596758467
C -3.4357933914 1.0574090612 -0.8816849395
H -4.1727946296 1.637929509 -1.4371359882
C -0.0471690552 2.6819631412 -1.2237672751
H -0.012897975 3.4741315726 -0.4531713059
C -1.508423723 2.5621842816 -1.7253734726
H -1.5158223635 2.3505005268 -2.8082919466
H -2.0928993202 3.483391217 -1.579830491
C 0.8911812431 3.0381400968 -2.3768199488
H 0.8937414042 2.2302338162 -3.1243605007
H 0.5404042515 3.9570825064 -2.8706128386
H 1.9282039875 3.2023869612 -2.0644195398
C 3.6845562855 -4.4459340634 -0.7961883772
C 6.6666616179 2.7195771867 0.7813337892
N 4.181352819 -5.4884474805 -0.8780502595
N 7.7415872131 3.0721672843 1.0271100521
C -6.1361535849 0.065090577 -0.943673553
C -5.8658785546 -0.159638848 1.3446127143
C -7.444206138 -0.57464766 -0.4632565519
H -5.8173450269 -0.2705141666 -1.9411727124
H -6.2490080374 1.1696169464 -0.9784233292
C -7.2708055957 -0.7051719337 1.0687197727
H -5.9072791372 0.9210686572 1.5930538204
H -5.3530223064 -0.6632923062 2.1724965724

H -8.3152382654 0.033415049 -0.7433067829
H -7.5741474372 -1.5641767949 -0.9228224753
H -8.0322281056 -0.1480463074 1.631624649
H -7.3415653171 -1.7573633029 1.3787205666
N -5.175309033 -0.3435425841 0.0658336313

Energy= -1435.3170372
Zero-point correction= 0.497473 (Hartree/Particle)
Thermal correction to Gibbs Free Energy= 0.438130
Sum of electronic and zero-point Energies= -1434.819564
Sum of electronic and thermal Energies= -1434.791323
Sum of electronic and thermal Enthalpies= -1434.790378
Sum of electronic and thermal Free Energies= -1434.878907
Imaginary Frequencies 0

Envelope-2-unstable-wB97X-D-MeCN

C -5.4627738759 -1.6418005557 0.8667369983
C -4.5169782508 -2.6869340454 0.8955955941
C -3.1996991468 -2.4567303188 0.5250252763
C -2.8126905452 -1.1771586076 0.0982126953
C -3.7653950585 -0.1267504902 0.1138425469
C -5.0860649754 -0.3470827662 0.4850945225
H -4.8260206697 -3.6827672408 1.2182570383
H -2.4832186319 -3.2767099725 0.5807260952
H -5.8136403579 0.4670821975 0.4905735539
C -1.7151470996 0.807626855 -0.4779290538
C -0.8624941474 1.8089640192 -0.9661424227
C -1.3591875873 3.0863359451 -1.1826492215
C -2.7154369658 3.3798400156 -0.9340665774
C -3.5939351644 2.3807297342 -0.4930477231
C -3.0874009158 1.1047697296 -0.2799426689
H 0.1842777293 1.600748024 -1.1863235794
H -0.6979322005 3.872224073 -1.5519988361
H -4.6511248917 2.6019971807 -0.3335151628
C -1.4959957254 -0.6263444581 -0.2487961062
C 1.0426260865 -0.8490845769 -0.1769275469
C 1.941791161 -1.7105156111 -0.8022314939
C -0.3123498825 -1.3289323402 -0.3871586974
C 1.5578730592 0.230192005 0.6274567475
C 2.9619467694 0.4906690526 0.5950423178
C 0.7526046123 1.0311427428 1.4774826542
H -0.2981351698 0.7766189263 1.6079833903
C 1.2720239967 2.1196842988 2.142385467
H 0.6273563863 2.7172450791 2.7909874983
C 2.622578166 2.4802885224 1.9621147713
H 3.0161804961 3.3882842045 2.4239990064
C 3.447981967 1.6724343033 1.2127095948
H 4.4919756629 1.9519235713 1.0715275523
C 3.8605558014 -0.4274348802 -0.0816036644
C 3.3256230588 -1.5045294489 -0.7793175732
H 3.9773773884 -2.2269915815 -1.270452246
C -0.224264257 -2.7591415661 -0.9204804562
H -0.3595850582 -3.4570344677 -0.0745624018
C 1.2355264133 -2.8741141882 -1.4177704606
H 1.2667607099 -2.8001907289 -2.5179120144
H 1.7138567061 -3.8280194077 -1.1505028701
C -1.2108915512 -3.1052648243 -2.0343614992
H -1.1172874358 -2.3869905414 -2.8639323692
H -0.9834825142 -4.1079130696 -2.4275704516
H -2.2569580471 -3.1090527779 -1.7058890595
C -3.205606827 4.7104151083 -1.155680027
C -6.8208389766 -1.9037918679 1.2507498241
N -3.5993639246 5.7854707616 -1.3340333243
N -7.9168707977 -2.1173285951 1.5599021196
C 6.1129987803 -0.9043645762 -0.9528283253
C 5.9433909093 -0.2398114171 1.2636778683
C 7.4975588482 -0.3447321933 -0.6064623028

H 5.8044154923 -0.7119633385 -1.9904162961
H 6.0952723391 -2.0005550182 -0.7905037303
C 7.3814909022 0.1013662795 0.8694348024
H 5.887126288 -1.260000539 1.6923578608
H 5.5182206856 0.4515715662 1.9990228926
H 8.2787860135 -1.1015616972 -0.7608321432
H 7.7362548584 0.5122303792 -1.2521775893
H 8.102038018 -0.4060373444 1.5255576541
H 7.5543013651 1.1833049358 0.9626478508
N 5.2277229112 -0.2200309978 -0.0193952986

Energy= -1435.3570718
Zero-point correction= 0.496311 (Hartree/Particle)
Thermal correction to Gibbs Free Energy= 0.436544
Sum of electronic and zero-point Energies= -1434.860760
Sum of electronic and thermal Energies= -1434.832434
Sum of electronic and thermal Enthalpies= -1434.831490
Sum of electronic and thermal Free Energies= -1434.920528
Imaginary Frequencies 0

Envelope-2-Inv-CAM-B3LYP

C 5.6302344737 -1.4571405042 -0.0605489754
C 4.7651424483 -2.5494945727 0.0721832163
C 3.3898282683 -2.3697566357 0.0735701179
C 2.8551491989 -1.08887106 -0.0754644082
C 3.7415271571 0.0038615202 -0.1931852579
C 5.1146371983 -0.1658071038 -0.1888685913
H 5.1816598557 -3.5443474138 0.1803931149
H 2.7597375529 -3.2377801479 0.2008388339
H 5.7882355164 0.6791778483 -0.2799072399
C 1.5868605411 0.8853115762 -0.2351113247
C 0.6314200417 1.8771618861 -0.4575881161
C 1.0372603825 3.1832544913 -0.6846895467
C 2.3971225012 3.5174522044 -0.7097983914
C 3.3672517669 2.5280764568 -0.53769103
C 2.9532660135 1.2261709615 -0.3159595646
H -0.4236957686 1.6464569996 -0.46464343
H 0.2987477499 3.9593745249 -0.8494561093
H 4.4191827944 2.785245134 -0.5952503374
C 1.4637060293 -0.5793565027 -0.0598335206
C -1.0658963801 -0.9104519785 0.2205137755
C -1.8981173815 -1.7842747256 -0.4556056065
C 0.3253334454 -1.326433279 0.0105467633
C -1.6469905645 0.0773156788 1.0775043194
C -3.0619753448 0.2403491469 1.0572658827
C -0.8931432731 0.8196227822 2.0176486809
H 0.1782565219 0.6715655027 2.0600734978
C -1.499157868 1.6913208622 2.8848097507
H -0.9001675544 2.2412735124 3.6031722385
C -2.8969678927 1.8505257851 2.8685797388
H -3.3748748408 2.5175163822 3.578183964
C -3.6569778658 1.1334740692 1.98131994
H -4.7368873589 1.2049577858 2.0093284291
C -3.8765700672 -0.5621792518 0.1745026735
C -3.2849024891 -1.6030763572 -0.5156802196
H -3.8762021351 -2.2638532511 -1.1380798584
C 0.2501957963 -2.80955917 -0.3360600685
H 1.0756204388 -3.1008596565 -0.9872457185
C -1.0984924819 -2.8854357009 -1.0950116285
H -1.5817446271 -3.862725771 -1.0021408406
H -0.9468024074 -2.6946679312 -2.1638488962
C 0.2178602474 -3.7076916988 0.9075707091
H -0.6579943191 -3.4777014603 1.519994863
H 0.1628830879 -4.7605782629 0.616705548
H 1.1017056865 -3.5725173759 1.5349528068
C 2.7922500981 4.8777496263 -0.9366828057
C 7.0493426944 -1.6667586993 -0.0560928575
N 3.108644706 5.975686018 -1.1187868035

N 8.1936138996 -1.8377441992 -0.0526751731
C -6.1594867465 -1.2519320137 -0.4954326483
C -5.6601448374 1.0026069782 -0.4528562741
C -6.3347761284 -0.8039121682 -1.960106943
H -7.1174314977 -1.1774496272 0.0316091345
H -5.8043769474 -2.2740405139 -0.3698681207
C -5.9946787706 0.7049320653 -1.9305805635
H -4.8771929095 1.7495390398 -0.331389521
H -6.5508622552 1.3562970452 0.0785045683
H -5.648125938 -1.3412318305 -2.6189158789
H -7.3473835923 -1.0004783483 -2.318981634
H -5.129489454 0.9169439955 -2.5630750971
H -6.8171593576 1.3290959263 -2.2868967322
N -5.2504618278 -0.2895792136 0.1240036061

Energy= -1436.0404633
Zero-point correction= 0.499340 (Hartree/Particle)
Thermal correction to Gibbs Free Energy= 0.439805
Sum of electronic and zero-point Energies= -1435.541123
Sum of electronic and thermal Energies= -1435.512926
Sum of electronic and thermal Enthalpies= -1435.511981
Sum of electronic and thermal Free Energies= -1435.600658
Imaginary Frequencies 0

Envelope-2-Inv-CAM-B3LYP-MeCN

C 5.6609239852 -1.4402947022 0.0100919423
C 4.8073204653 -2.5452296308 0.1407671913
C 3.4312930879 -2.3804579366 0.1226559089
C 2.8843912952 -1.103609902 -0.0421638622
C 3.7633627025 -0.0020101431 -0.1629734034
C 5.138550723 -0.1534024191 -0.139123714
H 5.2311588932 -3.5358081274 0.2617068215
H 2.8108105769 -3.2560543336 0.2444680647
H 5.8000837661 0.7010388072 -0.2323514626
C 1.5995731893 0.8497500894 -0.2543607159
C 0.6355970289 1.828893415 -0.5117539713
C 1.0292733731 3.1345446857 -0.7564570108
C 2.3886721987 3.4810096939 -0.7618643237
C 3.3710744729 2.5093663233 -0.5554334609
C 2.9652469118 1.2075634038 -0.3182594111
H -0.4171469821 1.588529213 -0.5388820263
H 0.2839182188 3.8975788665 -0.9507621822
H 4.4224407988 2.77289316 -0.597960729
C 1.4913607361 -0.6085334864 -0.0520834834
C -1.0344571857 -0.9545946252 0.2174671723
C -1.8655874116 -1.8532002169 -0.437772543
C 0.3517390864 -1.3666874867 0.0183404824
C -1.6293398462 0.0522309367 1.0441038279
C -3.0450552398 0.2164992757 0.999810281
C -0.8890349811 0.8071386724 1.9855602828
H 0.1817248494 0.659003057 2.0474140999
C -1.5058592671 1.6844908559 2.8415450326
H -0.9159020118 2.2399695512 3.5637995355
C -2.9034196541 1.8384105998 2.8090738929
H -3.3935336376 2.5074718772 3.508712089
C -3.6500283265 1.1137573717 1.9138791606
H -4.7288025283 1.195678355 1.9349301375
C -3.8526697414 -0.6011627452 0.1133038094
C -3.2477709448 -1.6792945294 -0.5200460641
H -3.8237554192 -2.3640323221 -1.1292340277
C 0.2925628628 -2.8557180451 -0.3020605738
H 1.1180318845 -3.1511657669 -0.9505514549
C -1.0607000708 -2.9656291551 -1.046404343
H -1.5370894614 -3.9419016133 -0.9200965309
H -0.9204161274 -2.8031451537 -2.1211149642
C 0.2801391417 -3.7297923921 0.9591279919
H -0.6042439188 -3.5149096915 1.5657975031

H 0.2531471527 -4.7874008848 0.6817607291
H 1.1596960101 -3.5639967142 1.5856855447
C 2.7718404606 4.8386199106 -1.00728447
C 7.0796192796 -1.6327562807 0.0351386598
N 3.0808712002 5.9368066303 -1.2052568952
N 8.2266915814 -1.7890563659 0.0550160427
C -6.1149501756 -1.2462824879 -0.6819996314
C -5.6366759302 1.0418901679 -0.3972711697
C -6.332047914 -0.6222171776 -2.056342033
H -7.0594689495 -1.2572130139 -0.1240223115
H -5.7316511993 -2.2640509435 -0.6909566764
C -6.4247790789 0.8736791737 -1.7218575126
H -4.7657807938 1.686725757 -0.5134050712
H -6.2766123777 1.4786742817 0.3750255506
H -5.4657773117 -0.8262962638 -2.6933668047
H -7.2236479542 -1.0021767707 -2.5602681343
H -6.0152765428 1.5031125997 -2.5146759308
H -7.4671990639 1.1676358732 -1.574293832
N -5.2048906558 -0.3202518973 -0.0027045831

Energy= -1436.0801855
Zero-point correction= 0.498432 (Hartree/Particle)
Thermal correction to Gibbs Free Energy= 0.439002
Sum of electronic and zero-point Energies= -1435.581753
Sum of electronic and thermal Energies= -1435.553513
Sum of electronic and thermal Enthalpies= -1435.552569
Sum of electronic and thermal Free Energies= -1435.641184
Imaginary Frequencies 0

Envelope-2-Inv-wB97X-D

C -5.6427014186 -1.3749905036 0.1592507945
C -4.7983921884 -2.4948315066 0.0848643855
C -3.4156281737 -2.3454448739 0.0800519188
C -2.852076067 -1.0667212163 0.1646817281
C -3.7155342517 0.0529728222 0.2281164377
C -5.0965707135 -0.0867998185 0.22656342
H -5.2378374168 -3.4917428115 0.0257396483
H -2.8007845228 -3.2391136403 0.0011911337
H -5.753570996 0.7834333692 0.2747832244
C -1.5374608251 0.8847008387 0.2471079694
C -0.5543861416 1.8611392901 0.4404552821
C -0.9276860243 3.1900720744 0.6027643476
C -2.2826987556 3.5629971011 0.5934302687
C -3.2803046482 2.5892618444 0.4565520338
C -2.8978868291 1.2636256577 0.2990812826
H 0.5010554054 1.6000801149 0.4767463378
H -0.1638130242 3.9562499485 0.7434756755
H -4.3328345479 2.8761593209 0.4903880578
C -1.4503688527 -0.5876454977 0.125737543
C 1.0637757969 -0.9581696165 -0.2059401771
C 1.9094044843 -1.8228686408 0.4720128058
C -0.3226077454 -1.356607547 0.0557193589
C 1.6241381481 0.0093084038 -1.1007068523
C 3.0407973475 0.178417721 -1.1095194629
C 0.8458515215 0.7275411917 -2.0448700451
H -0.232990328 0.5733912928 -2.0665151738
C 1.4324645506 1.5882696437 -2.9422524996
H 0.8129106805 2.1246197912 -3.6639098323
C 2.8331904415 1.7534253382 -2.9561965716
H 3.2979061342 2.4109577867 -3.6935367602
C 3.616143965 1.0539049694 -2.0680275026
H 4.7021105044 1.1315505614 -2.1218176288
C 3.8768109704 -0.6022459953 -0.2199738451
C 3.3000110676 -1.6408008185 0.4981924451
H 3.9077975265 -2.2937283813 1.1252051929
C -0.2515247172 -2.8314452025 0.4284108911
H -1.0654118448 -3.1031876978 1.1137347952

C 1.1166476519 -2.9055390187 1.1508997257
H 1.5944868134 -3.8935819503 1.0658630677
H 0.9963444123 -2.68781632 2.2253542719
C -0.273458654 -3.7446723043 -0.803602943
H 0.5916511708 -3.5366183918 -1.4517098378
H -0.2303473653 -4.8017294329 -0.5009518814
H -1.1804917019 -3.5949623906 -1.4066050981
C -2.6449088907 4.9460520877 0.7507462581
C -7.0699760214 -1.5527022253 0.1591599637
N -2.9332738033 6.0601573489 0.8760443319
N -8.2183574194 -1.698199401 0.1591011428
C 6.174600941 -1.2417599927 0.4412243024
C 5.6511210962 1.0010180197 0.3275145492
C 6.370625105 -0.743194852 1.8863478789
H 7.126398676 -1.1724267449 -0.1117862217
H 5.8334740178 -2.2802192778 0.3554226937
C 6.0262466359 0.7619111897 1.8070496997
H 4.8531498555 1.7424723835 0.201726876
H 6.5276405258 1.3491950777 -0.2444525634
H 5.6881491151 -1.2628716303 2.5748122283
H 7.3934379231 -0.9284892534 2.242894597
H 5.1715953675 0.9998329186 2.4563868283
H 6.8599690301 1.4062988079 2.1195665213
N 5.2407604769 -0.3133156214 -0.1795707984

Energy= -1435.321076
Zero-point correction= 0.497786 (Hartree/Particle)
Thermal correction to Gibbs Free Energy= 0.438198
Sum of electronic and zero-point Energies= -1434.823290
Sum of electronic and thermal Energies= -1434.795092
Sum of electronic and thermal Enthalpies= -1434.794147
Sum of electronic and thermal Free Energies= -1434.882878
Imaginary Frequencies 0

Envelope-2-Inv-wB97X-D-MeCN

C 5.6775727627 -1.4190669391 0.037755988
C 4.8334546772 -2.5402344221 0.1395450274
C 3.452130845 -2.3937476159 0.0990124815
C 2.8895378102 -1.1179845275 -0.0568354875
C 3.7570201387 -0.0001221166 -0.1522697845
C 5.1382327033 -0.1338378685 -0.1060148172
H 5.26966023 -3.5340326338 0.2546443435
H 2.8384038457 -3.2864823978 0.1956150338
H 5.7915498833 0.7376531844 -0.1794922005
C 1.5793768174 0.8188106829 -0.2849533503
C 0.600803941 1.7838180151 -0.5627446828
C 0.9769524264 3.1021110407 -0.7858254449
C 2.3338115953 3.4758066831 -0.7506376258
C 3.331423878 2.5169212662 -0.5284033218
C 2.9426190216 1.2011196658 -0.3131068525
H -0.4532852031 1.5212303858 -0.6244155943
H 0.2160172503 3.8559024669 -0.9954324296
H 4.3858638451 2.7995237956 -0.5413525194
C 1.4911877535 -0.6401107834 -0.084306494
C -1.0277362498 -0.9928863745 0.2069565455
C -1.8702712578 -1.903802487 -0.4261970852
C 0.3528996787 -1.4075167607 -0.0066094415
C -1.6092245146 0.0310975903 1.0239789159
C -3.0258025927 0.2109152856 0.9808562559
C -0.8533982943 0.7903036847 1.9547213214
H 0.2236450453 0.631405675 2.0157864523
C -1.4578949106 1.689559548 2.8035234679
H -0.854115237 2.2525017048 3.5191650122
C -2.8573708059 1.85553533 2.7771545655
H -3.341536246 2.5407747843 3.476283517
C -3.6185446801 1.1239110646 1.892803175
H -4.703027709 1.2191834276 1.9258841192

C -3.8469988459 -0.6110467001 0.1012057831
C -3.2534326081 -1.7229542768 -0.5020165093
H -3.8411213124 -2.4228106432 -1.0950912085
C 0.2925004241 -2.9001332515 -0.29851881
H 1.114482826 -3.207907651 -0.957741483
C -1.0704830682 -3.028011912 -1.0199192786
H -1.548955365 -4.0072734749 -0.8695739389
H -0.9466829921 -2.8821482132 -2.1057636561
C 0.3108660173 -3.7404186729 0.9847201171
H -0.5719867899 -3.5197214758 1.6050130768
H 0.2960572247 -4.8121023816 0.7356625777
H 1.2040402569 -3.5413237231 1.5944945335
C 2.6995693625 4.8462522842 -0.9728745303
C 7.1020020667 -1.5931488367 0.0847739445
N 2.9927611039 5.9526915348 -1.1516673922
N 8.2510990483 -1.7352123895 0.1223984207
C -6.1137953554 -1.2108877226 -0.7151209426
C -5.6106072392 1.0668199602 -0.3903884578
C -6.3734138667 -0.5499182875 -2.063799532
H -7.0440607029 -1.2384387515 -0.1206672414
H -5.7353950947 -2.2362216896 -0.7694686515
C -6.4496955057 0.9353258268 -1.6862422518
H -4.7329164786 1.711844681 -0.5281323999
H -6.2197506374 1.4982960237 0.4190873575
H -5.5212139503 -0.7420062772 -2.7351668697
H -7.2855223209 -0.9175462421 -2.5543559518
H -6.0690762242 1.5907401701 -2.4816251989
H -7.4917329357 1.2271441423 -1.4891046335
N -5.1789482549 -0.3035130246 -0.0554395232

Energy= -1435.3610684
Zero-point correction= 0.497005 (Hartree/Particle)
Thermal correction to Gibbs Free Energy= 0.437733
Sum of electronic and zero-point Energies= -1434.864064
Sum of electronic and thermal Energies= -1434.835865
Sum of electronic and thermal Enthalpies= -1434.834920
Sum of electronic and thermal Free Energies= -1434.923335
Imaginary Frequencies 0

Envelope-2-Inv-TS-CAM-B3LYP

C -3.2797171559 3.2865101636 0.4057168392
C -2.4322276944 3.4137064111 -0.699055061
C -1.7957463688 2.3005663054 -1.230669423
C -1.9487396891 1.0493072265 -0.6382331789
C -2.9524375195 0.9193053053 0.3456173815
C -3.5806209271 2.0170191186 0.9056022543
H -2.2969470924 4.3869373121 -1.1564323289
H -1.2473799529 2.416764613 -2.154192882
H -4.333185067 1.9041404958 1.6780688262
C -2.5612956785 -1.2047082961 -0.5336243791
C -2.9572361159 -2.5139249808 -0.8202926147
C -3.9821413974 -3.1132017981 -0.0972218377
C -4.6438958827 -2.4216026779 0.918796738
C -4.3222985087 -1.0845467024 1.1682141825
C -3.3179642437 -0.4914359552 0.4282227253
H -2.5307919006 -3.074337052 -1.6388906678
H -4.2845182393 -4.1268930711 -0.3338203878
H -4.8750716343 -0.5248930801 1.9146774164
C -1.454232754 -0.3063173668 -1.0094943145
C 1.2074490814 -0.3256932193 -1.0825430151
C 2.0244597888 -1.4444270305 -0.9855029845
C -0.1951251071 -0.7943114168 -1.293366845
C 1.8558472921 0.9441197427 -0.8292792871
C 3.1719118552 0.9669129523 -0.2746493981
C 1.3125751934 2.1818273605 -1.2136788029
H 0.3744566038 2.1743544348 -1.7265972406
C -3.9087069937 4.448551399 0.9637580024

C -5.6821009423 -3.066758022 1.6689414224
N -4.4117066685 5.3874295409 1.4154543955
N -6.516792879 -3.5896802275 2.2759410271
C 1.9591550384 3.3758614358 -1.0153735708
H 1.4927048761 4.300678982 -1.339356256
C 3.2349274994 3.3936434194 -0.4363131714
H 3.7654822935 4.3301356629 -0.3017754428
C 3.8288844427 2.2058499008 -0.0942761328
H 4.847264734 2.195641594 0.2722770776
C 3.8779119058 -0.2608168344 0.0022453444
C 3.3217782544 -1.4313113532 -0.4519148163
H 3.8315490288 -2.379185093 -0.3272301366
C -0.0843299248 -2.266544398 -1.7028034739
H -0.7533525392 -2.8645083451 -1.089591275
C 1.3481240636 -2.7046105921 -1.4016677892
H 1.842008507 -3.1377569631 -2.279221023
H 1.391546696 -3.462995232 -0.6127735255
C -0.4564749996 -2.4460554933 -3.1784608127
H 0.2351889207 -1.8867497242 -3.8157345076
H -0.3992674445 -3.5007073974 -3.4648611529
H -1.4653479596 -2.0835261957 -3.3890808812
N 5.1202702898 -0.1864051281 0.6438918604
C 5.1277963103 0.2878227341 2.0389974722
H 6.0490261873 0.8561008471 2.2097926726
H 4.2837877886 0.9483852547 2.2308973554
C 5.9902960872 -1.3606039338 0.669428699
H 5.866841832 -1.9675437387 -0.2266771974
H 7.024106243 -0.9970951657 0.6718622565
C 5.6922161579 -2.0935649269 1.992431496
H 6.5929472022 -2.5521934724 2.4062112792
H 4.9624616973 -2.8927933722 1.8412352746
C 5.1109289615 -0.9892523644 2.9065953131
H 4.0869908228 -1.2317072909 3.2001590089
H 5.6880092855 -0.8553354511 3.8242460334

Energy= -1436.0003308
Zero-point correction= 0.498962 (Hartree/Particle)
Thermal correction to Gibbs Free Energy= 0.440348
Sum of electronic and zero-point Energies= -1435.501369
Sum of electronic and thermal Energies= -1435.473879
Sum of electronic and thermal Enthalpies= -1435.472935
Sum of electronic and thermal Free Energies= -1435.559983
Imaginary Frequencies 1

Envelope-2-Inv-TS-CAM-B3LYP-MeCN

C -3.2454994404 3.2775698962 0.3968828984
C -2.3896264283 3.4091145752 -0.7046294675
C -1.7646454414 2.2945842121 -1.2431392422
C -1.9373867688 1.0369892147 -0.6626838074
C -2.9416203925 0.9114323287 0.3236880622
C -3.5633763913 2.0097553479 0.891102153
H -2.2391106684 4.3842114716 -1.1539343161
H -1.2106685433 2.4141345688 -2.1616610001
H -4.3145660246 1.8931640648 1.6647954254
C -2.5364059937 -1.2188957505 -0.5343887516
C -2.9092617469 -2.5472978531 -0.7792363358
C -3.9139231879 -3.1455624274 -0.0303866782
C -4.5821201709 -2.4345515704 0.9715442401
C -4.2865870349 -1.0855189593 1.1868653609
C -3.2977138015 -0.4974425462 0.4218433715
H -2.481965729 -3.1285531169 -1.5817562781
H -4.1945412005 -4.1724557599 -0.2361475526
H -4.8378266224 -0.5118310948 1.9241603535
C -1.4573984534 -0.3147528015 -1.0472711847
C 1.1909305174 -0.3220190925 -1.1855905171
C 2.0225190428 -1.4403919823 -1.143299933
C -0.200090419 -0.7933417377 -1.3880444218

C 1.8357751387 0.9366676086 -0.8662782088
C 3.122884906 0.9304347604 -0.2443154492
C 1.3345051423 2.1862561749 -1.2736677659
H 0.4414458098 2.1968625797 -1.8651161886
C -3.8655523163 4.4392918379 0.9592444065
C -5.6015451355 -3.0769458667 1.7441031972
N -4.363165629 5.3795298702 1.416237652
N -6.4241431095 -3.5977632136 2.3708151175
C 1.9832417175 3.3707729279 -1.0199111474
H 1.5536606848 4.304766102 -1.3685544316
C 3.2186853835 3.3628770781 -0.359296566
H 3.7526063542 4.2895685046 -0.1763048254
C 3.7789015233 2.1601474606 -0.0042662858
H 4.7735454152 2.1455641569 0.420947662
C 3.8191811607 -0.3164721742 0.0103652825
C 3.2991528653 -1.4560783969 -0.5707525973
H 3.8071210649 -2.4091896808 -0.4957619602
C -0.0950785392 -2.2519061732 -1.8426603483
H -0.69174243 -2.8760592969 -1.1815146093
C 1.3649827309 -2.6677835082 -1.6691117381
H 1.8239553859 -2.9609327054 -2.6205823864
H 1.4806112196 -3.5166767434 -0.9889201083
C -0.5918184315 -2.4182393005 -3.2815086281
H 0.0248044534 -1.8281804711 -3.9670563371
H -0.5252522223 -3.4670703522 -3.5865422227
H -1.6278740694 -2.0911703247 -3.3986825907
N 4.9991133287 -0.2953910833 0.7339515988
C 5.0138166939 0.3095134856 2.0872525935
H 5.7294838062 1.1358748958 2.1293595055
H 4.0279250604 0.7092642762 2.3239539029
C 5.860135667 -1.4786368867 0.8062790549
H 5.7028983449 -2.1498481816 -0.0349792557
H 6.9021888563 -1.1383939161 0.7638664877
C 5.5646050306 -2.076599672 2.1775247174
H 6.3441912584 -2.7604121061 2.5209093547
H 4.6186168725 -2.6258792761 2.1423213695
C 5.4370663357 -0.8239821465 3.0564901171
H 4.712207495 -0.9536163194 3.8628288782
H 6.3989987358 -0.5809260611 3.5152469147

Energy= -1436.0418831
Zero-point correction= 0.498182 (Hartree/Particle)
Thermal correction to Gibbs Free Energy= 0.439821
Sum of electronic and zero-point Energies= -1435.543701
Sum of electronic and thermal Energies= -1435.516212
Sum of electronic and thermal Enthalpies= -1435.515268
Sum of electronic and thermal Free Energies= -1435.602062
Imaginary Frequencies 1

Envelope-2-Inv-TS-wB97X-D

C -3.1965726285 3.2916596021 0.414518422
C -2.3893303853 3.4115214502 -0.7272034682
C -1.7796433561 2.2906489165 -1.2817295777
C -1.9167647448 1.0399424564 -0.6757092144
C -2.8860127507 0.9147714016 0.3459853795
C -3.4875940618 2.0210452244 0.9292999147
H -2.2645054878 4.3889754098 -1.1955927517
H -1.2617952333 2.3989477659 -2.2318670599
H -4.2147486897 1.9118646907 1.7358660135
C -2.5255634356 -1.215057708 -0.5445095001
C -2.9224526065 -2.5324468909 -0.8101959667
C -3.9195160272 -3.1348997087 -0.0452207852
C -4.5523517216 -2.4386482355 0.9926026819
C -4.2276188535 -1.0946122631 1.2243844284
C -3.2505961333 -0.4981050139 0.4433997619
H -2.5155431198 -3.1006151608 -1.6427907874
H -4.2242313841 -4.1589610714 -0.2666023392

H -4.7572918111 -0.5289206278 1.9929666819
C -1.4399079127 -0.3161694227 -1.0610553297
C 1.2123535162 -0.3257530137 -1.1612105199
C 2.0403655437 -1.442425685 -1.0780053454
C -0.1829130974 -0.8032606181 -1.3712799445
C 1.8410865743 0.9467189033 -0.8713044859
C 3.1402577416 0.9718833115 -0.2737026879
C 1.2971302087 2.1861740012 -1.2619460094
H 0.3765740645 2.1738985702 -1.8209436667
C -3.794854537 4.4618814288 0.9982396753
C -5.5613456815 -3.086714767 1.7860261889
N -4.2714967804 5.4057632081 1.4692454323
N -6.3706152615 -3.6113077307 2.4262321702
C 1.9250138487 3.3874645491 -1.0237046691
H 1.4586155191 4.3177953732 -1.3551884081
C 3.1828085474 3.408281767 -0.3978657123
H 3.7028804103 4.3534865962 -0.2309473262
C 3.7808031888 2.2175281378 -0.0545320743
H 4.7937724479 2.2160141749 0.3475517433
C 3.8520673516 -0.2586380636 0.0003168688
C 3.3276995618 -1.4270733174 -0.5135689057
H 3.8498127481 -2.3776672989 -0.4002691448
C -0.0691567649 -2.2697034059 -1.7949057332
H -0.7121122677 -2.8798328899 -1.1518205973
C 1.3773625183 -2.6942277154 -1.5413213345
H 1.868367268 -3.0704513401 -2.4543992564
H 1.4543071614 -3.4953703384 -0.7897443006
C -0.5035409367 -2.448540921 -3.2523333794
H 0.1563358859 -1.8748210107 -3.9218665121
H -0.4497046076 -3.508259342 -3.546559959
H -1.5313423506 -2.0925638565 -3.4156936681
N 5.0536415062 -0.1946264809 0.7017229896
C 5.0162324398 0.2858236759 2.0880449315
H 5.9251667304 0.877979397 2.2872668777
H 4.1489065907 0.9340044528 2.260563687
C 5.9403069455 -1.3483542041 0.7473583949
H 5.8647459219 -1.955958979 -0.1621466419
H 6.9749323484 -0.9687873444 0.792234914
C 5.596853004 -2.0925352433 2.0518739648
H 6.483200111 -2.5710018767 2.4912795499
H 4.8588319064 -2.8861756882 1.8639932174
C 4.9970010946 -0.9919547605 2.9568957375
H 3.9645418111 -1.2409696406 3.2404208382
H 5.5639125521 -0.8519550002 3.8880377976

Energy= -1435.2797982
Zero-point correction= 0.497672 (Hartree/Particle)
Thermal correction to Gibbs Free Energy= 0.439293
Sum of electronic and zero-point Energies= -1434.782126
Sum of electronic and thermal Energies= -1434.754723
Sum of electronic and thermal Enthalpies= -1434.753778
Sum of electronic and thermal Free Energies= -1434.840505
Imaginary Frequencies 1

Envelope-2-Inv-unstable-CAM-B3LYP

C 5.3132128853 -1.8765374406 -0.865227025
C 4.2983523747 -2.8432572635 -0.90900545
C 3.0024472869 -2.5229652405 -0.5393606547
C 2.7068160034 -1.2326032356 -0.0946479837
C 3.7278434122 -0.2570504989 -0.0979481055
C 5.0248486808 -0.5690390991 -0.4686131961
H 4.5375292486 -3.8458126873 -1.2446132661
H 2.2321149969 -3.2809767364 -0.6109277097
H 5.808447391 0.1807487524 -0.468280186
C 1.7508433037 0.8290763035 0.4744211094
C 0.9767005003 1.8867237918 0.9546970724
C 1.5697961126 3.1156842781 1.192085544

C 2.9420662703 3.30402882 0.9733576082
C 3.7381256795 2.2439703048 0.5347940067
C 3.1394553977 1.0180649935 0.2990364986
H -0.0802234741 1.7606060036 1.1478787436
H 0.9738231133 3.945399725 1.5545147032
H 4.8045367705 2.3878724174 0.3995856245
C 1.425598933 -0.5912107646 0.2486341223
C -1.1259233259 -0.6475893599 0.2225271299
C -2.0610399022 -1.4540984503 0.8530050664
C 0.2091694389 -1.209088534 0.3979850054
C -1.5972232822 0.441205383 -0.5875600608
C -2.9860953829 0.766542842 -0.5517295791
C -0.765007106 1.1811392065 -1.4602706023
H 0.2682174229 0.8845181046 -1.5723956827
C -1.2431957574 2.2545913403 -2.1672628173
H -0.5807774372 2.7993525818 -2.831817586
C -2.5789489274 2.6649049408 -2.0136934188
H -2.9379685405 3.5541001241 -2.5207463183
C -3.4284327486 1.9288259843 -1.2295744038
H -4.4563652082 2.2424339832 -1.1071580576
C -3.9152061749 -0.075490824 0.1630607566
C -3.4285173354 -1.1787087948 0.8425729085
H -4.1062704945 -1.8689327344 1.3303385459
C 0.0336407779 -2.6452013571 0.9106580902
H 0.0802688114 -3.3395813887 0.0608315064
C -1.413402367 -2.6569890639 1.4628223257
H -1.392043029 -2.5750026904 2.5562323479
H -1.9532167168 -3.5792015035 1.2276514245
C 1.0158150024 -3.0889224673 1.9956788118
H 0.9924422108 -2.3853877272 2.8325415922
H 0.7226057119 -4.0718134705 2.3761547679
H 2.0450435622 -3.1585139659 1.6505788629
C 3.5318350743 4.5879187043 1.2200842015
C 6.6484028475 -2.2305276502 -1.2510665003
N 4.0056047327 5.6249344428 1.4180692378
N 7.7250098022 -2.5187740735 -1.5621475219
C -6.1941246563 -0.3637587588 1.0909644239
C -5.9782892563 0.2442710313 -1.1724361151
C -6.8970235894 -1.4756085098 0.3139602933
H -6.9240803672 0.3986065613 1.3922034833
H -5.6730616768 -0.6835869792 1.9920156999
C -7.0923430616 -0.8313566751 -1.0668828203
H -5.2703420723 0.0331497241 -1.9744316763
H -6.4181835102 1.2270353514 -1.3709438763
H -6.2444593948 -2.3511871918 0.2425792366
H -7.8363393085 -1.7926286457 0.7724586822
H -7.0300195088 -1.5598908111 -1.8776185439
H -8.076102835 -0.3592168853 -1.1319991078
N -5.2747540882 0.2419542131 0.128914762

Energy= -1436.0341234
Zero-point correction= 0.498866 (Hartree/Particle)
Thermal correction to Gibbs Free Energy= 0.439278
Sum of electronic and zero-point Energies= -1435.535257
Sum of electronic and thermal Energies= -1435.506997
Sum of electronic and thermal Enthalpies= -1435.506053
Sum of electronic and thermal Free Energies= -1435.594845
Imaginary Frequencies 0

Envelope-2-Inv-unstable-CAM-B3LYP-MeCN

C 5.3082492922 -1.879150007 -0.8753308168
C 4.2951793484 -2.8530188045 -0.8984252463
C 3.0025638595 -2.5314847571 -0.5235592895
C 2.7067425018 -1.2310185673 -0.0975967485
C 3.7307702471 -0.253838365 -0.1163146477
C 5.0265349468 -0.5656320324 -0.491861575
H 4.5329642918 -3.8608646327 -1.2199395447

H 2.2360865444 -3.2950426828 -0.5743336268
H 5.8069933083 0.187806396 -0.5009166526
C 1.7583538494 0.825453578 0.4782817033
C 0.9869542991 1.8862989237 0.9661063563
C 1.5789891266 3.1163563324 1.1923869909
C 2.9506633158 3.3043082327 0.9515062189
C 3.7476656443 2.2471768355 0.5045780587
C 3.1470768498 1.0196474616 0.2827302966
H -0.0662384607 1.7594151149 1.1792943416
H 0.9855323089 3.9447649119 1.5629765041
H 4.8119601731 2.3895123311 0.3500818042
C 1.4342106468 -0.5883999041 0.2535363137
C -1.1172624813 -0.6585526878 0.22693125
C -2.0556708064 -1.4733332667 0.8549969749
C 0.2103753283 -1.2096040334 0.415019915
C -1.5947161865 0.4313979535 -0.5826162587
C -2.9867164789 0.747524997 -0.5534359852
C -0.7636622207 1.1787612674 -1.4498598036
H 0.270961781 0.8865035684 -1.5633953333
C -1.2433516205 2.2533968513 -2.1575567898
H -0.5807628513 2.8019542191 -2.8195706069
C -2.5807684989 2.6566195009 -2.0074656346
H -2.9446042968 3.5446896561 -2.5135844438
C -3.4300327314 1.9106518356 -1.2288269158
H -4.4596734018 2.2229166254 -1.1197061112
C -3.9175093488 -0.1023969166 0.1627451539
C -3.4207052938 -1.2043730594 0.8466782149
H -4.0930445843 -1.9007274797 1.3318604677
C 0.0489856879 -2.6372953871 0.9474903046
H 0.1353185824 -3.3389379222 0.1081343789
C -1.4074263869 -2.673221961 1.4650481639
H -1.416081915 -2.5995385355 2.5588290637
H -1.9337588356 -3.5960372412 1.205759163
C 1.0208435149 -3.042310729 2.055432092
H 0.9674818099 -2.3312443815 2.8853155605
H 0.7410277064 -4.0272634249 2.4410850935
H 2.0567345618 -3.0993582399 1.7248799012
C 3.539998526 4.5869721036 1.186849463
C 6.6384256315 -2.2337156663 -1.2665123837
N 4.0158297032 5.6255989934 1.3771887088
N 7.7148013577 -2.5213994585 -1.5826156579
C -6.1945909217 -0.4419185192 1.0796235477
C -5.9920824548 0.2950358107 -1.1524065105
C -6.9135094085 -1.4954918711 0.2448314252
H -6.9109797239 0.3178555264 1.4163339412
H -5.6789855436 -0.8173633663 1.9612736378
C -7.1253632029 -0.760446845 -1.0860471815
H -5.3019138313 0.1071229675 -1.9747205976
H -6.4112445964 1.2963651845 -1.28715997
H -6.2642972409 -2.3662325544 0.1099972156
H -7.8484215007 -1.832642788 0.6981492574
H -7.0956445612 -1.4346789347 -1.9444880008
H -8.0977738787 -0.2612119219 -1.0934549733
N -5.2698482608 0.2007881956 0.1403057535

Energy= -1436.0748461
Zero-point correction= 0.498051 (Hartree/Particle)
Thermal correction to Gibbs Free Energy= 0.438677
Sum of electronic and zero-point Energies= -1435.576795
Sum of electronic and thermal Energies= -1435.548564
Sum of electronic and thermal Enthalpies= -1435.547620
Sum of electronic and thermal Free Energies= -1435.636169
Imaginary Frequencies 0

Envelope-2-Inv-unstable-wB97X-D

C 5.0711315868 -2.4991200145 -0.539414564
C 3.9816789358 -3.3521567321 -0.2835665467

C 2.7360128478 -2.8315784508 0.0421835258
C 2.5674693226 -1.4449363162 0.1414682137
C 3.6576719518 -0.595685181 -0.1634431726
C 4.9058001114 -1.1089820921 -0.492676308
H 4.1228211511 -4.4319674407 -0.3514979108
H 1.9017902134 -3.5143608105 0.205465322
H 5.7454912433 -0.4512651791 -0.7246097655
C 1.8040807341 0.7717004279 0.1920661193
C 1.1377558085 1.9830914078 0.4110660167
C 1.8299646311 3.1794097246 0.2762794829
C 3.1969287467 3.186654473 -0.0568546406
C 3.8879939809 1.9806218893 -0.2305523726
C 3.1893854505 0.7876634862 -0.0958234429
H 0.0845445471 2.0031881866 0.6889125596
H 1.3137786315 4.1278651545 0.4324685704
H 4.9562304265 1.9898658492 -0.4546369862
C 1.3633175905 -0.6249122676 0.3586677819
C -1.1765560371 -0.4384186696 0.4244769967
C -2.1455058528 -0.9514268712 1.2787328761
C 0.107923446 -1.07045635 0.7023347383
C -1.5825625299 0.4588967196 -0.6232302523
C -2.9238939174 0.9511999675 -0.615184189
C -0.7322705412 0.856374223 -1.6867828796
H 0.2570904182 0.4094575974 -1.7673326173
C -1.1326086558 1.7907461017 -2.6132539401
H -0.4554242864 2.0772934905 -3.4204001039
C -2.4006123031 2.3969227169 -2.5059577997
H -2.6900378475 3.1903811116 -3.1976902642
C -3.2771997941 1.9745207836 -1.5343828472
H -4.2582849386 2.4407665015 -1.451814904
C -3.8934717022 0.4171230671 0.3183288878
C -3.4795127446 -0.5336182115 1.2440137642
H -4.1971188749 -1.0012994917 1.9187946659
C -0.1626300559 -2.2948017995 1.5826692142
H -0.2178472035 -3.1916884649 0.938446841
C -1.5768742745 -2.024217372 2.1540154507
H -1.4981144266 -1.6698474166 3.1959116075
H -2.2151043257 -2.9208038903 2.1730935772
C 0.8407559379 -2.5282493392 2.7120949802
H 0.9275588022 -1.6229003671 3.3321086777
H 0.491695437 -3.3495156107 3.3563389889
H 1.8458400171 -2.7842248148 2.3592903564
C 3.8905209574 4.4382434462 -0.199619213
C 6.352977293 -3.059503845 -0.8721826896
N 4.4459891844 5.4473777626 -0.3160201543
N 7.383914194 -3.5138199782 -1.1387801441
C -6.1399240177 0.626776977 1.3413347347
C -5.986114664 0.6599913268 -0.9852596518
C -6.9843022863 -0.5693720664 0.8940957381
H -6.7781432956 1.5226060626 1.4432037685
H -5.6186814208 0.4960353744 2.2969367269
C -7.1012381976 -0.3597510515 -0.627682137
H -5.3358668224 0.3053005524 -1.7944100242
H -6.4313424827 1.6148028135 -1.3112387375
H -6.4519506498 -1.5072586876 1.1160048671
H -7.9592319115 -0.6150116746 1.3988631188
H -6.973043351 -1.3023495169 -1.1770514477
H -8.0878431865 0.0404315962 -0.9022183925
N -5.2096423608 0.8576851025 0.2457486499

Energy= -1435.3148904
Zero-point correction= 0.497133 (Hartree/Particle)
Thermal correction to Gibbs Free Energy= 0.438913
Sum of electronic and zero-point Energies= -1434.817757
Sum of electronic and thermal Energies= -1434.790309
Sum of electronic and thermal Enthalpies= -1434.789365
Sum of electronic and thermal Free Energies= -1434.875977
Imaginary Frequencies 1

Envelope-2-Inv-unstable-wB97X-D-MeCN

C 5.3286415598 -1.8694953181 -0.8872570661
C 4.3260011626 -2.8615519693 -0.8825151383
C 3.0295084006 -2.5529942278 -0.4974331639
C 2.7179244796 -1.245547331 -0.090176255
C 3.7291623492 -0.2506459081 -0.1348726527
C 5.02972668 -0.550267255 -0.5214767947
H 4.575469819 -3.8785262872 -1.1906197381
H 2.2695049503 -3.3344083849 -0.525779779
H 5.801586523 0.2215504498 -0.5499931133
C 1.7447489469 0.8019769113 0.4787365001
C 0.9569351706 1.8574090477 0.9645839685
C 1.5273031485 3.1074176372 1.1552327565
C 2.8941836561 3.3218159303 0.8815864014
C 3.7080414139 2.2693358474 0.4405653091
C 3.1285024279 1.0207505752 0.2534115966
H -0.0961967635 1.7101952033 1.2031541468
H 0.9170773153 3.9340855143 1.5235686448
H 4.7729166912 2.4297954295 0.2603430364
C 1.4424683376 -0.6170228736 0.269832517
C -1.1053042019 -0.7049364905 0.2399827101
C -2.0462622879 -1.5192829489 0.8767371834
C 0.2196411275 -1.250527337 0.4375926814
C -1.5795855776 0.3898872199 -0.5677838665
C -2.9699301631 0.722930077 -0.522395159
C -0.7440807648 1.136166939 -1.4371866822
H 0.2910225889 0.8261606033 -1.5718341329
C -1.2124502858 2.2390968034 -2.1166131937
H -0.5433247569 2.7920188296 -2.7799648216
C -2.5415038343 2.6671815154 -1.9352963485
H -2.8967247511 3.5819102871 -2.4144893477
C -3.3994004178 1.9137052783 -1.1649197555
H -4.4274687077 2.2480121442 -1.0375454479
C -3.9103396872 -0.1355797458 0.1839758608
C -3.4114348099 -1.2451273907 0.871816516
H -4.0874589093 -1.9477846761 1.3578620564
C 0.0691682951 -2.6677149995 0.9916800382
H 0.1815485925 -3.3818513121 0.1563434014
C -1.3942770786 -2.7183687223 1.4852324839
H -1.4255990829 -2.6506488018 2.5856363617
H -1.9163346696 -3.6469153749 1.2103994963
C 1.0415644834 -3.0347832826 2.1115253379
H 0.9704486347 -2.3050058377 2.9333172492
H 0.7819314874 -4.0254690954 2.5150565011
H 2.0871684665 -3.0748947701 1.7833514357
C 3.4611610105 4.6253523657 1.0771761191
C 6.6633799776 -2.2115271671 -1.2880660263
N 3.9168042201 5.6791263976 1.2348948357
N 7.7408806528 -2.4895157772 -1.6113294252
C -6.2047829671 -0.4961006731 1.0526795261
C -5.9693452025 0.3595023741 -1.1315013813
C -6.9730630333 -1.4631524787 0.160665124
H -6.8851670498 0.2825106198 1.4407867825
H -5.7101893227 -0.9531568502 1.9157350006
C -7.1511069475 -0.6420693042 -1.1226733839
H -5.2868628481 0.1861567196 -1.9736608385
H -6.3433687167 1.3921664913 -1.2082876414
H -6.3579022 -2.3578143824 -0.0265183389
H -7.9265214191 -1.7881619471 0.5998330356
H -7.1557375594 -1.2678511035 -2.0256527137
H -8.1045873702 -0.0943282163 -1.0960530403
N -5.2564779632 0.1439594589 0.1431365617

Energy= -1435.3559707
Zero-point correction= 0.496307 (Hartree/Particle)
Thermal correction to Gibbs Free Energy= 0.436209
Sum of electronic and zero-point Energies= -1434.859663

Sum of electronic and thermal Energies= -1434.831327
Sum of electronic and thermal Enthalpies= -1434.830383
Sum of electronic and thermal Free Energies= -1434.919762
Imaginary Frequencies 0

Half-Chair-1-CAM-B3LYP

C 2.8058509743 3.4195029695 -0.8101261776
C 1.4303153044 3.1743941408 -0.9105721584
C 0.9165225486 1.9073613124 -0.681558862
C 1.7766452423 0.8662511482 -0.330738157
C 3.1650136312 1.1141975695 -0.289223769
C 3.6857131768 2.3771504048 -0.5112247293
H 0.7657283671 3.9887709351 -1.1752397343
H -0.145585088 1.7429443731 -0.7900201901
H 4.7534022049 2.5635541712 -0.4741440677
C 2.8838972264 -1.1791202138 0.0059848847
C 3.3119254838 -2.4874510438 0.2394195926
C 4.6651821717 -2.7585851698 0.3749221415
C 5.6130735745 -1.73119341 0.297619926
C 5.2030417568 -0.4132796957 0.0857535939
C 3.8516641358 -0.1521599666 -0.0555736574
H 2.6131958964 -3.3065763704 0.3273122755
H 4.9995844497 -3.7749711491 0.5484900128
H 5.9395213546 0.3812893249 0.0372520645
C 1.5368385051 -0.5781256602 -0.1233388072
C -1.0259022096 -0.7375591485 -0.05639924
C -1.8508094437 -1.560650958 -0.8029415026
C 0.3458333523 -1.2450159148 -0.1407032689
C -1.614717988 0.3121590188 0.7120152851
C -3.0101452147 0.5866868948 0.5617763906
C -0.8681079494 1.0706450415 1.6457751391
H 0.1656221934 0.8002632512 1.8189520065
C -1.4215963253 2.1292641654 2.3152213407
H -0.826620275 2.6946354861 3.0247215547
C -2.752683888 2.4978901419 2.05612524
H -3.1771978621 3.3768285356 2.5292873882
C -3.5204815298 1.7405363791 1.2095033435
H -4.5338637941 2.0551355953 1.0180225644
C -3.836082846 -0.2895865383 -0.2480977446
C -3.2188854316 -1.3367214279 -0.9282442645
H -3.8013336532 -2.0229782072 -1.5267782242
C 0.2026397927 -2.7251544866 -0.4822027083
H 1.0647668778 -3.0810797665 -1.0482885273
C -1.0684641661 -2.7147000906 -1.3676003847
H -1.6213898475 -3.6579574111 -1.3273083737
H -0.8018240512 -2.5334495632 -2.4152832337
C -0.0136800617 -3.6040705922 0.7565494347
H -0.9278610682 -3.3078412772 1.277728917
H -0.1118751197 -4.6542803055 0.4671229745
H 0.810802398 -3.5221111931 1.4683371052
C 7.0071912854 -2.0349697089 0.4448052811
C 3.3124300981 4.7417763442 -1.0397336309
N 8.1312658319 -2.282145198 0.5636494688
N 3.7189367425 5.8094320699 -1.2234866163
C -6.0974806243 0.3103232568 0.7303172025
C -5.955734904 -0.9210139107 -1.3316418628
C -7.396439008 -0.454931433 0.4821075668
H -6.3004072259 1.3859985825 0.676761237
H -5.6538762174 0.1004223441 1.7064216976
C -7.4122146159 -0.5870019961 -1.0391336141
H -5.7924028767 -1.9970848918 -1.1725280797
H -5.64294808 -0.6840257697 -2.3528947268
H -7.3495117719 -1.4466213956 0.9434090874
H -8.2651940215 0.066155968 0.8894749242
H -8.0948141748 -1.3559078497 -1.4058364345
H -7.6890105367 0.3648945771 -1.5031185265
N -5.2038012064 -0.1191500585 -0.3624127166

Energy= -1436.0431903
Zero-point correction= 0.499625 (Hartree/Particle)
Thermal correction to Gibbs Free Energy= 0.440862
Sum of electronic and zero-point Energies= -1435.543565
Sum of electronic and thermal Energies= -1435.515492
Sum of electronic and thermal Enthalpies= -1435.514548
Sum of electronic and thermal Free Energies= -1435.602329
Imaginary Frequencies 0

Half-Chair-1-CAM-B3LYP-MeCN

C 2.7775131192 3.4204910359 -0.812088823
C 1.4004478499 3.1687666229 -0.9094870294
C 0.8978970177 1.8988676665 -0.6775670796
C 1.7676609862 0.8604577643 -0.3299585588
C 3.157002139 1.1209469924 -0.2939401737
C 3.6700617647 2.3869806165 -0.5163734654
H 0.7281267654 3.9773170976 -1.1736926216
H -0.1634760598 1.7293285642 -0.7862911705
H 4.7373891462 2.576778162 -0.4801270196
C 2.88759391 -1.1724322993 0.0031670816
C 3.3255752889 -2.4822397912 0.2271346951
C 4.6800663442 -2.7454847191 0.3522236692
C 5.6206529014 -1.7079928734 0.2716613517
C 5.2069017629 -0.3894685647 0.0665014746
C 3.8520541507 -0.1388578779 -0.0635635953
H 2.6345530669 -3.3076425381 0.3145935198
H 5.0195802726 -3.7617263497 0.5181851737
H 5.9351902228 0.4127120656 0.0123327717
C 1.5404505247 -0.5806201465 -0.1192434404
C -1.0194133938 -0.7590378736 -0.0330441227
C -1.8546747003 -1.5898596386 -0.7704710845
C 0.3455020095 -1.2574761688 -0.1391200751
C -1.6087174315 0.2883508728 0.7428828441
C -3.0095606991 0.5576565135 0.6130161257
C -0.8540376843 1.0422589757 1.6738749594
H 0.1823138305 0.773640276 1.8352723815
C -1.4023409287 2.0912652776 2.3658255744
H -0.7998233369 2.6497077126 3.075254505
C -2.7390611079 2.4522628785 2.1331551934
H -3.166563036 3.3183014601 2.6274892267
C -3.5154125254 1.6984518856 1.287300307
H -4.5349258916 2.0099497872 1.1313948342
C -3.8429275578 -0.3179764988 -0.204258306
C -3.2187335371 -1.3692712039 -0.8883800512
H -3.8024390327 -2.0592106586 -1.4810112628
C 0.2138496285 -2.7358090941 -0.4916671865
H 1.0699203341 -3.0791602792 -1.0738870864
C -1.0752306268 -2.7372115205 -1.3481733361
H -1.6212632242 -3.6830960299 -1.2952742568
H -0.8337633389 -2.5517293932 -2.4009795469
C 0.031389655 -3.6265135877 0.7444912289
H -0.8875290987 -3.3623227417 1.2756339818
H -0.0404413012 -4.6754264197 0.4428289366
H 0.8599623018 -3.5314534523 1.4502100412
C 7.0148490981 -2.0043699746 0.4069574476
C 3.2737905537 4.7431443036 -1.0438918028
N 8.1422224737 -2.2449822639 0.5162038153
N 3.6743376755 5.8134346964 -1.2309259033
C -6.1254864735 0.4090137027 0.6620946079
C -5.9436026275 -0.9968092607 -1.2906805716
C -7.4439371215 -0.3168431086 0.4083173276
H -6.2630428126 1.4823689066 0.4930203132
H -5.7510141106 0.2643785854 1.6772165392
C -7.3935573404 -0.5792532329 -1.0936460806
H -5.8278617581 -2.0632937999 -1.0559623468
H -5.5735267616 -0.8340977684 -2.3068095262
H -7.4655097923 -1.2651273507 0.9547933158

H -8.3021196535 0.2801876435 0.7234006822
H -8.0893339596 -1.3513769068 -1.4274708452
H -7.6063405501 0.3400165333 -1.6487520771
N -5.19752582 -0.1678709818 -0.33124983

Energy= -1436.0836999
Zero-point correction= 0.498983 (Hartree/Particle)
Thermal correction to Gibbs Free Energy= 0.440444
Sum of electronic and zero-point Energies= -1435.584717
Sum of electronic and thermal Energies= -1435.556691
Sum of electronic and thermal Enthalpies= -1435.555746
Sum of electronic and thermal Free Energies= -1435.643256
Imaginary Frequencies 0

Half-Chair-1-wB97X-D

C 2.7346469163 3.4256494327 -0.7689455585
C 1.3637321551 3.1496498844 -0.9112938662
C 0.8723471536 1.8646696827 -0.7144950611
C 1.7496276694 0.8367203327 -0.3516683089
C 3.1336906808 1.1130557076 -0.2739933218
C 3.6329735185 2.3947173289 -0.4644533182
H 0.6817533264 3.9564242266 -1.1840679567
H -0.1893147412 1.6757961094 -0.8591197762
H 4.7022725519 2.6031223965 -0.3971692244
C 2.8889031729 -1.1941362567 -0.0169496275
C 3.337287765 -2.5030833525 0.1979612114
C 4.6964365745 -2.7530212845 0.353102343
C 5.6304233028 -1.7045642769 0.3119914307
C 5.198493551 -0.3866100654 0.115409462
C 3.8408573559 -0.1463738432 -0.0458626803
H 2.648027928 -3.3425214096 0.2562625251
H 5.0468636719 -3.7738176465 0.5133962005
H 5.9239003171 0.4286375672 0.092447233
C 1.5342413121 -0.6127395708 -0.1561756677
C -1.0234412621 -0.784872524 -0.0621108559
C -1.8589839523 -1.6096893707 -0.8008484378
C 0.3460273342 -1.2916705882 -0.1671945189
C -1.5966892352 0.2753417225 0.7058603653
C -2.9902334004 0.5721353499 0.5528885443
C -0.8320699561 1.0317936824 1.6318813844
H 0.2023068338 0.740185149 1.8143108616
C -1.3616828016 2.1209647251 2.2795519584
H -0.7498075675 2.6901250186 2.9821733252
C -2.6857646903 2.5169077353 2.0074297087
H -3.092646797 3.4225894823 2.4612734765
C -3.4746646058 1.7544717802 1.1774063242
H -4.4872262192 2.0929777109 0.9830541469
C -3.8347087573 -0.3093300213 -0.2414403369
C -3.2291581977 -1.3759032196 -0.9158501111
H -3.8251695944 -2.0704965358 -1.5042588456
C 0.2000567032 -2.772142281 -0.4964252692
H 1.0597069382 -3.1323927946 -1.0772317541
C -1.082195842 -2.7681247638 -1.3647804364
H -1.639475229 -3.7160727571 -1.3149542418
H -0.8281144445 -2.5872669609 -2.4225223951
C 0.0120645938 -3.6349819373 0.757303963
H -0.9014656195 -3.3376284463 1.2947626984
H -0.0795885721 -4.6970994526 0.4843775603
H 0.8542403316 -3.5299086465 1.4562187147
C 7.0308775193 -1.9861208603 0.4788146408
C 3.2181866494 4.7661975134 -0.9630262831
N 8.1575500814 -2.2155327546 0.6132968975
N 3.6042607099 5.8466475387 -1.1174558052
C -6.0810327315 0.3829222101 0.7097542333
C -5.9675575899 -0.9390205339 -1.2929789704
C -7.4014436549 -0.3528503132 0.4923381906
H -6.2491993803 1.4673119443 0.5932805322

H -5.6529337342 0.2145561297 1.7089016055
C -7.4153371574 -0.5530405872 -1.0204173503
H -5.8348711848 -2.0227859592 -1.1087570955
H -5.6415188788 -0.7369921186 -2.3254453523
H -7.384797287 -1.3290921441 1.0026940149
H -8.2615872845 0.213355066 0.8750022635
H -8.1237206739 -1.3208223389 -1.3595390625
H -7.6666165441 0.3902890768 -1.5308758913
N -5.1985250319 -0.1422068879 -0.3399004349

Energy= -1435.3243953
Zero-point correction= 0.498247 (Hartree/Particle)
Thermal correction to Gibbs Free Energy= 0.439466
Sum of electronic and zero-point Energies= -1434.826148
Sum of electronic and thermal Energies= -1434.798119
Sum of electronic and thermal Enthalpies= -1434.797175
Sum of electronic and thermal Free Energies= -1434.884929
Imaginary Frequencies 0

Half-Chair-1-wB97X-D-MeCN

C 2.7180106253 3.4290646275 -0.7564016805
C 1.3459552288 3.1494920529 -0.9048498659
C 0.8619847369 1.8622296904 -0.7115275794
C 1.7449288906 0.8344693737 -0.3497088074
C 3.1299697695 1.1203921824 -0.2686146256
C 3.6252453599 2.4047859746 -0.4524641433
H 0.6594293163 3.9524108952 -1.1790219192
H -0.1985234626 1.6709594617 -0.8626414745
H 4.6942139069 2.6139358837 -0.3786280334
C 2.8904180379 -1.1870754222 -0.017775895
C 3.3451731319 -2.4989011413 0.1854355918
C 4.7043146307 -2.7437226784 0.3374223438
C 5.6331851518 -1.6869683083 0.3026585712
C 5.2013556005 -0.3671234237 0.1146193736
C 3.8414571937 -0.1344835361 -0.0430279846
H 2.6624142357 -3.3441097151 0.2356444728
H 5.0565923781 -3.7656097936 0.4885141838
H 5.9198951181 0.4547077065 0.0931366846
C 1.5376251279 -0.6115126842 -0.156341706
C -1.0180738638 -0.7973413025 -0.0534622917
C -1.8633467118 -1.6274382549 -0.7872181092
C 0.3437107197 -1.2975871247 -0.177380456
C -1.5920406144 0.2615913553 0.7209009738
C -2.9903833926 0.5533570875 0.5851013445
C -0.8225231396 1.0149239082 1.645373656
H 0.2143222281 0.7261147059 1.8196614401
C -1.35020706 2.0962322019 2.3116323365
H -0.7336024602 2.6598707588 3.015584412
C -2.67891781 2.4860113309 2.0598221918
H -3.0899553396 3.381391369 2.5308994001
C -3.4734327117 1.7250544826 1.2298908303
H -4.4912779628 2.0604526117 1.0616555507
C -3.8423603417 -0.3293205751 -0.2136670338
C -3.2297083013 -1.3968931107 -0.896436865
H -3.8258029425 -2.0940137258 -1.4816022764
C 0.2077933667 -2.7748147261 -0.5222822158
H 1.0626605389 -3.1227590108 -1.1168752102
C -1.0896910117 -2.7788698104 -1.3647131909
H -1.6414004219 -3.7288079103 -1.3062433689
H -0.8581723487 -2.5920696367 -2.4263900183
C 0.0487982514 -3.6521239299 0.7258572777
H -0.8723552703 -3.3901420994 1.2696907141
H -0.0131650243 -4.7122809774 0.4375547599
H 0.890841066 -3.5344153052 1.4231814438
C 7.0324591061 -1.9642336298 0.4656069814
C 3.1950225281 4.7697283826 -0.9458689149
N 8.1611752879 -2.1897444941 0.5970851899

N 3.5782991438 5.8524393501 -1.0979952861
C -6.0983527641 0.4516832245 0.658503425
C -5.9623017611 -1.003986843 -1.2554089997
C -7.4318002139 -0.2572531823 0.4427219955
H -6.2172122521 1.5279353476 0.4511664428
H -5.7170561919 0.3368957936 1.6827928865
C -7.4038250055 -0.5609421402 -1.051049993
H -5.8585370012 -2.0769453085 -1.0108836392
H -5.5990536309 -0.8591274847 -2.2844093547
H -7.4617885519 -1.1954505932 1.0194814186
H -8.2817565035 0.364822185 0.7548006531
H -8.1200872411 -1.3344605906 -1.3592786773
H -7.6121568483 0.3520199885 -1.6315205529
N -5.1947210207 -0.181451233 -0.3142607272

Energy= -1435.3651782
Zero-point correction= 0.497508 (Hartree/Particle)
Thermal correction to Gibbs Free Energy= 0.438768
Sum of electronic and zero-point Energies= -1434.867670
Sum of electronic and thermal Energies= -1434.839653
Sum of electronic and thermal Enthalpies= -1434.838709
Sum of electronic and thermal Free Energies= -1434.926410
Imaginary Frequencies 0

Half-Chair-1-thermal_isomerization-TS-CAM-B3LYP

C 4.3840163008 -2.7836778437 -0.0161362229
C 3.1790745777 -3.2680875082 0.5466718872
C 2.1491407248 -2.4125441665 0.8588169346
C 2.294401959 -1.0295495739 0.6173716707
C 3.5152701882 -0.5428218915 0.0408235439
C 4.544906703 -1.4211802217 -0.2666263478
H 3.0791345214 -4.3329037853 0.7277463322
H 1.2305595036 -2.8073126079 1.2847379028
H 5.4730510466 -1.0624715458 -0.7006172728
C 2.081677852 1.2462028843 0.3823369759
C 1.6809567092 2.5990240232 0.3292007248
C 2.5412307649 3.5439536325 -0.1770083189
C 3.8245889911 3.1825765109 -0.652428394
C 4.2364443918 1.8508813118 -0.6169169825
C 3.3810783056 0.8832577726 -0.1087363599
H 0.6970031615 2.8983049241 0.6790954638
H 2.2434688107 4.5861145849 -0.2209913511
H 5.2206826458 1.5845351824 -0.9894930071
C 1.4181960998 0.0701225315 0.8236300786
C -1.1191855515 -0.2279836084 0.78966033
C -2.1691680796 -0.2498257655 1.7527455316
C 0.0927000805 -0.0171045418 1.4166735263
C -1.4470835429 -0.4577820708 -0.6083623563
C -2.8132679424 -0.6009242976 -0.973494445
C -0.4552450118 -0.6153814714 -1.5876333344
H 0.5787692163 -0.4789187648 -1.3010254721
C -0.7852906501 -0.9695828205 -2.8787662658
H -0.0005256398 -1.0948061168 -3.6168735624
C -2.1170392478 -1.2081121008 -3.2216674128
H -2.3743366242 -1.5468466369 -4.219182329
C -3.1103492598 -1.02341594 -2.2852275284
H -4.1246536248 -1.2598062305 -2.5655934641
C -3.8561756449 -0.3823949301 0.0337268668
C -3.486042618 -0.3567878254 1.4042031375
H -4.2421337125 -0.3206297214 2.1748217884
C -0.0976978448 0.0716215939 2.9163324955
H 0.4416120815 -0.7914540535 3.3307463013
C -1.6131982186 -0.0783761953 3.1334563227
H -2.0451793803 0.8034977279 3.6178568718
H -1.8687830541 -0.9370780489 3.7628245335
C 0.5115078316 1.3327519602 3.5332077047
H -0.0023568106 2.2304419345 3.1773532421

H 0.426088442 1.3000619908 4.6221355228
H 1.5658453163 1.4167162064 3.2672035655
C 4.6919214384 4.191126473 -1.1764849568
C 5.4333554571 -3.702095659 -0.3321033953
N 5.3879263047 5.0145932822 -1.6018119211
N 6.278120735 -4.4532263165 -0.5880627081
C -5.6992600917 0.2136621628 -1.5924327771
C -6.1798354013 -0.0448264536 0.7643858154
C -6.9509799069 1.0042201849 -1.2285941714
H -5.9772267759 -0.665864515 -2.1821130164
H -4.9619570584 0.7886319833 -2.1522414414
C -7.4637799076 0.2553698426 -0.0014232908
H -5.9155660188 0.790927921 1.4219677858
H -6.2408262951 -0.9513273491 1.3714221043
H -6.6869972306 2.033586223 -0.9676764144
H -7.6657461874 1.0353837252 -2.0524306794
H -8.1693243439 0.8308215093 0.599545895
H -7.9562863833 -0.6743460415 -0.3016537034
N -5.1478136615 -0.1932162797 -0.2829202523

Energy= -1435.9965294
Zero-point correction= 0.497351 (Hartree/Particle)
Thermal correction to Gibbs Free Energy= 0.437678
Sum of electronic and zero-point Energies= -1435.499178
Sum of electronic and thermal Energies= -1435.471225
Sum of electronic and thermal Enthalpies= -1435.470281
Sum of electronic and thermal Free Energies= -1435.558851
Imaginary Frequencies 1

Half-Chair-1-thermal_isomerization-TS-CAM-B3LYP-MeCN

C 4.3471495685 -2.7729192091 -0.1143400301
C 3.1170232542 -3.255208738 0.4060295026
C 2.1008193387 -2.3891267191 0.7288554684
C 2.2799117586 -0.9976919546 0.542343845
C 3.5282826799 -0.5198010647 0.008399277
C 4.5470472257 -1.4033952245 -0.3120690533
H 2.989034867 -4.3236965707 0.5451779718
H 1.1658002662 -2.7747421982 1.1239947907
H 5.4911856799 -1.0466663272 -0.7130343841
C 2.120848884 1.2778452411 0.4062265146
C 1.7480381283 2.6442264549 0.4036697024
C 2.6336715778 3.5924723975 -0.0459447851
C 3.9218652039 3.2226278841 -0.5162397673
C 4.3110472369 1.8802606986 -0.5351360032
C 3.4260402483 0.9140489263 -0.0833300669
H 0.7648675854 2.9449686977 0.7514199241
H 2.3550124486 4.6411956349 -0.0491012829
H 5.2956340552 1.6073283119 -0.9032981985
C 1.4225843412 0.1046731004 0.7841888645
C -1.1122582799 -0.2496933289 0.7692509788
C -2.168225885 -0.2758339443 1.7492892188
C 0.0829500076 0.0164562938 1.3814070952
C -1.4594261852 -0.5305257899 -0.6221894178
C -2.8283620073 -0.6847397685 -0.9675098047
C -0.4867299545 -0.7292594084 -1.6101583026
H 0.5536319702 -0.5893606733 -1.3528503175
C -0.8324966669 -1.1297084886 -2.8872862891
H -0.0574968895 -1.2826928668 -3.6312128024
C -2.1666079612 -1.3753036185 -3.2020095808
H -2.4427648479 -1.7447993017 -4.1835966185
C -3.1466618705 -1.1526605671 -2.253976289
H -4.1671782218 -1.3874767469 -2.5129481388
C -3.8678337243 -0.4252838321 0.0462685178
C -3.4780964941 -0.4041482916 1.4230691079
H -4.2274744071 -0.3721469138 2.2007410191
C -0.0961155586 0.15490616 2.8824936448
H 0.4958163232 -0.6496568661 3.3380855199

C -1.5979274459 -0.0805800366 3.1185871007
H -2.0768160199 0.7642618646 3.6223818006
H -1.7849846632 -0.964063624 3.7371511999
C 0.4095517013 1.4801337149 3.4552859289
H -0.1529207778 2.3241413061 3.0452471031
H 0.2835184818 1.4851800461 4.5414611768
H 1.4685627114 1.630357122 3.2364358498
C 4.8192496013 4.2275867865 -0.9774836096
C 5.3834755408 -3.6945546068 -0.4391543203
N 5.5486967829 5.0487797526 -1.3530077891
N 6.2263133303 -4.4476857706 -0.7031387513
C -5.7065400962 0.1546002141 -1.5803245601
C -6.1637309202 0.0083108149 0.7971379348
C -6.9327865142 0.9879709591 -1.2343937294
H -6.0107966708 -0.7574387839 -2.1011520089
H -4.9693736879 0.6834337794 -2.1830090839
C -7.4456700369 0.3064717875 0.0300751692
H -5.8710697347 0.8571815278 1.4219031332
H -6.2299489621 -0.8817826158 1.4250326721
H -6.639964731 2.0208322512 -1.0228480478
H -7.655137068 0.9923790095 -2.052222406
H -8.1250243469 0.927278064 0.6160284005
H -7.9621006403 -0.6237726747 -0.224126015
N -5.1356910892 -0.1877670962 -0.2565752787

Energy= -1436.0623501
Zero-point correction= 0.497613 (Hartree/Particle)
Thermal correction to Gibbs Free Energy= 0.439067
Sum of electronic and zero-point Energies= -1435.564737
Sum of electronic and thermal Energies= -1435.537144
Sum of electronic and thermal Enthalpies= -1435.536200
Sum of electronic and thermal Free Energies= -1435.623283
Imaginary Frequencies 1

Half-Chair-1-thermal_isomerization-TS-wB97X-D

C 4.3485568894 2.6696777302 0.847727108
C 3.152795075 3.3137148926 0.4330369946
C 2.1364072821 2.6038580232 -0.1713381727
C 2.2894011787 1.213429405 -0.3840800559
C 3.4942350615 0.5603590535 0.0498265738
C 4.5122855619 1.2938407973 0.6543298725
H 3.0474518811 4.3872111175 0.6022645728
H 1.2206693436 3.1172815108 -0.4781274096
H 5.4329463378 0.8065717693 0.9836312369
C 2.0665451336 -1.0233576908 -0.8672704508
C 1.6503381244 -2.3247709951 -1.2362896808
C 2.4924024986 -3.3948250617 -1.0191690585
C 3.769657871 -3.2128482173 -0.4255664349
C 4.1929469974 -1.9356121072 -0.043445343
C 3.3544816269 -0.8435233241 -0.253512689
H 0.6678858174 -2.4875038601 -1.6875075917
H 2.1826712859 -4.4029890616 -1.3018008986
H 5.1737520566 -1.806313179 0.4200165761
C 1.4222943827 0.2401033137 -0.9498971501
C -1.1050195132 0.4899118428 -0.7352397659
C -2.1927297087 0.7996667474 -1.605554284
C 0.0811717559 0.5002548581 -1.4464145177
C -1.36771132 0.2765860114 0.6803055407
C -2.7194365366 0.2947658228 1.1290171913
C -0.3313462845 0.131852059 1.6208852665
H 0.6970544773 0.0886796431 1.2651998631
C -0.6056742911 0.0824540743 2.9753601051
H 0.2159841066 -0.0191322942 3.6866396504
C -1.9230809005 0.2073935889 3.4305931955
H -2.1369837341 0.233933149 4.5005196541
C -2.9592686525 0.3072120112 2.5218447901
H -3.9650579431 0.4451086885 2.9071679412

C -3.8077479906 0.3766378253 0.1440934627
C -3.4979938398 0.7819142113 -1.1856347698
H -4.2888912254 0.9782338689 -1.9057938845
C -0.1668950316 0.8717579971 -2.8890947792
H 0.3598632969 1.8337780966 -3.032689878
C -1.6892526951 1.0646315424 -2.99155292
H -2.1468909892 0.3645601039 -3.7088044142
H -1.9729937393 2.0774710631 -3.3185600575
C 0.4295797741 -0.1320089055 -3.8773386685
H -0.0638158514 -1.1120078383 -3.7869313052
H 0.3024323289 0.2249199602 -4.9094675748
H 1.5018859021 -0.270492686 -3.6820703195
C 4.6190347496 -4.3483404166 -0.2116398721
C 5.3846044297 3.4372568008 1.4756793502
N 5.2985724913 -5.2721889685 -0.0401352414
N 6.2162115969 4.0642413263 1.9855141897
C -5.5594527074 -0.7291753292 1.5853551659
C -6.1583908964 0.2237993217 -0.549496832
C -6.8243701444 -1.3965664415 1.0588299919
H -5.8104404884 -0.0738991421 2.4349052416
H -4.7881545136 -1.4385669769 1.9115588959
C -7.4016156576 -0.3202677326 0.1449659417
H -5.9235949751 -0.3676920917 -1.4508985266
H -6.2546794908 1.2773236021 -0.850054475
H -6.5674399362 -2.2977828657 0.4802184874
H -7.4996520643 -1.6968446045 1.8706226696
H -8.1405312778 -0.6987824903 -0.5730644442
H -7.8859556596 0.4686769651 0.7412891904
N -5.0792304267 0.063547876 0.4399403158

Energy= -1435.2789866
Zero-point correction= 0.495877 (Hartree/Particle)
Thermal correction to Gibbs Free Energy= 0.436195
Sum of electronic and zero-point Energies= -1434.783110
Sum of electronic and thermal Energies= -1434.755203
Sum of electronic and thermal Enthalpies= -1434.754259
Sum of electronic and thermal Free Energies= -1434.842791
Imaginary Frequencies 1

Half-Chair-1-thermal_isomerization-TS-wB97X-D-MeCN

C 4.3148384447 -2.7831099794 -0.1680614708
C 3.1039749314 -3.2679408779 0.404887629
C 2.1005772472 -2.3994561486 0.7765603133
C 2.2770908083 -1.0046380696 0.5877970987
C 3.4999092187 -0.5232634305 -0.0044269166
C 4.5075452108 -1.4093964634 -0.3723435225
H 2.9780041544 -4.3438030901 0.5457279061
H 1.1753073038 -2.7858167265 1.2120272762
H 5.4366439663 -1.0471860724 -0.8197118736
C 2.095848818 1.2706738139 0.4393218824
C 1.7049280421 2.6354192935 0.440209834
C 2.5642089653 3.5908594745 -0.0563628909
C 3.8402828954 3.2282818015 -0.5766702021
C 4.2442050632 1.8860907115 -0.5992609295
C 3.3846445347 0.9120679112 -0.1012798345
H 0.7277796047 2.9292614365 0.8306942383
H 2.2712039918 4.6431691245 -0.0573395431
H 5.221055498 1.614677435 -1.0075460999
C 1.4252617666 0.0953849792 0.8602860511
C -1.1026584072 -0.2603985544 0.8226010646
C -2.1702257992 -0.2914166804 1.7927904455
C 0.0864020527 0.0001964505 1.4543798116
C -1.4246220777 -0.5364396621 -0.5768230921
C -2.7913097137 -0.6918230377 -0.9426494648
C -0.433260762 -0.7313375586 -1.5532122776
H 0.610928172 -0.5870880744 -1.2819473613
C -0.7608466849 -1.1361645159 -2.8380277979

H 0.0304811682 -1.2877913748 -3.5752535164
C -2.0933299953 -1.3879320046 -3.1714009798
H -2.3560033998 -1.7626223969 -4.1625633264
C -3.090999206 -1.1646050128 -2.2359589494
H -4.1138433215 -1.4020884959 -2.5138151288
C -3.8473365414 -0.4268561667 0.0581211611
C -3.4798694714 -0.4180507544 1.4454462848
H -4.2435326499 -0.3883365083 2.2195349504
C -0.1093951979 0.140178496 2.9490860664
H 0.4862727312 -0.6670570027 3.4119913533
C -1.6121871251 -0.1035245185 3.1679144506
H -2.1068206144 0.7372240032 3.6780395056
H -1.8048972328 -0.9995574123 3.7785959924
C 0.3942921542 1.4700478721 3.5115499205
H -0.1651678747 2.3151992074 3.0810929866
H 0.2598107739 1.4904519403 4.6032490714
H 1.4631841359 1.6146599093 3.2975740049
C 4.7106671756 4.2409356981 -1.0856362174
C 5.3384507559 -3.707308169 -0.5434207861
N 5.415613153 5.0661679216 -1.4985698393
N 6.1678769096 -4.4607295694 -0.8478015667
C -5.6412513605 0.1827031663 -1.5981247345
C -6.1480986904 0.0356158169 0.7622731427
C -6.8715340183 1.0203589552 -1.2798809038
H -5.9372782671 -0.7311453093 -2.135215235
H -4.8848077886 0.7156781219 -2.1869559748
C -7.4134042775 0.3402176339 -0.0281940032
H -5.8616339558 0.8884531282 1.3978979007
H -6.2325442831 -0.857433448 1.3959294168
H -6.5771758402 2.0584405399 -1.0593429595
H -7.5795719392 1.0315980779 -2.1187650334
H -8.1079884656 0.9649622664 0.5481253645
H -7.9317189453 -0.5934677408 -0.2959257633
N -5.1045573009 -0.1654651801 -0.2672452281

Energy= -1435.3445919
Zero-point correction= 0.496228 (Hartree/Particle)
Thermal correction to Gibbs Free Energy= 0.437894
Sum of electronic and zero-point Energies= -1434.848364
Sum of electronic and thermal Energies= -1434.820826
Sum of electronic and thermal Enthalpies= -1434.819881
Sum of electronic and thermal Free Energies= -1434.906698
Imaginary Frequencies 1

Half-Chair-1-TS-CAM-B3LYP

C 3.4323323507 3.3217868699 -0.1825388362
C 2.4734277556 3.3753902498 0.8327281025
C 1.8141053321 2.2241999542 1.2425514309
C 2.0566087112 1.0053668642 0.6124174069
C 3.1651289274 0.9474513068 -0.261428566
C 3.8177882066 2.0839644206 -0.7029800556
H 2.2663574491 4.3211808107 1.3198713931
H 1.1721865002 2.2896315832 2.1087068868
H 4.6507585585 2.023167962 -1.3945236409
C 2.7523362861 -1.2211119498 0.4804248388
C 3.1713985117 -2.5176346841 0.7883917899
C 4.3054765553 -3.0510661342 0.187115294
C 5.0533430888 -2.3026466738 -0.7238266815
C 4.7046326131 -0.9734180763 -0.9791008524
C 3.5901181926 -0.446181592 -0.3557636338
H 2.6697978697 -3.1164101379 1.5348406874
H 4.6247232677 -4.0561813319 0.4376926459
H 5.3218579483 -0.3680756904 -1.6337382605
C 1.5578907945 -0.3839089736 0.8494580111
C -1.1093689814 -0.4928539702 0.6320379305
C -1.8563712049 -1.616460876 0.3023303481
C 0.2929538724 -0.9315090842 0.9228438144

C -1.793566409 0.7692385919 0.4846097203
C -3.0939720733 0.827639219 -0.1126530497
C -1.2537804833 1.9852336105 0.9384604785
H -0.3597924301 1.9312712488 1.5211372671
C 4.083702933 4.5230147747 -0.6183721381
C 6.2051202892 -2.880481052 -1.3534455791
N 4.6048484947 5.4937961204 -0.9712906262
N 7.1319698194 -3.3491914406 -1.8632509789
C -1.8270110764 3.2036146821 0.6853436944
H -1.3581324526 4.1106793747 1.0529891789
C -3.005744966 3.2667586893 -0.0699005834
H -3.4360137694 4.2233779896 -0.3458488991
C -3.6239970221 2.0997617441 -0.4381797615
H -4.5308926979 2.1550129002 -1.0198329057
C -3.8247652769 -0.3882089883 -0.3951245278
C -3.1607626948 -1.5836057443 -0.1939553462
H -3.6429873376 -2.5293907246 -0.3996885075
C 0.1916769639 -2.4444630799 1.1561130286
H 1.0064834743 -2.9583919208 0.6574464246
C -1.1204377089 -2.8931974549 0.5199875744
H -1.684929861 -3.5815724872 1.1580467253
H -0.9524769743 -3.4101332894 -0.4316753836
C 0.2454051776 -2.7531297652 2.656855062
H -0.6018474268 -2.2910864008 3.1725094175
H 0.1980593266 -3.8326346392 2.8298442353
H 1.1601455873 -2.3686251024 3.1149171998
C -5.7435396263 -1.6359783646 -1.2924708274
C -6.1731143413 0.5518309568 -0.3876021567
C -7.2083322873 -1.2780904226 -1.5045890708
H -5.668949125 -2.4066202811 -0.5110739684
H -5.2467021595 -2.0204261131 -2.1882209567
C -7.4492584424 -0.288507261 -0.366505554
H -6.3051065415 1.3870266002 -1.0845130531
H -5.9152035955 0.96936182 0.5884451328
H -7.3480210339 -0.7884751236 -2.4735165646
H -7.8582344882 -2.154508298 -1.4689755957
H -8.3460198875 0.3213808098 -0.4938747263
H -7.5374816196 -0.8257156752 0.5833307726
N -5.1278014229 -0.3786854803 -0.8599983451

Energy= -1436.002925
Zero-point correction= 0.499219 (Hartree/Particle)
Thermal correction to Gibbs Free Energy= 0.441219
Sum of electronic and zero-point Energies= -1435.503706
Sum of electronic and thermal Energies= -1435.476307
Sum of electronic and thermal Enthalpies= -1435.475362
Sum of electronic and thermal Free Energies= -1435.561706
Imaginary Frequencies 1

Half-Chair-1-TS-CAM-B3LYP-MeCN

C 3.343729137 3.3276207701 -0.1450880003
C 2.4226503293 3.3510682336 0.9094863827
C 1.7901443733 2.1850481875 1.3147933874
C 2.0209246076 0.9822550621 0.6440765912
C 3.0903227999 0.9585846754 -0.2807065394
C 3.7199852551 2.1102923068 -0.7182705919
H 2.224434981 4.281842992 1.4288125805
H 1.1802302497 2.2242757835 2.2042870558
H 4.5214738566 2.0699245871 -1.4480671262
C 2.698795518 -1.2394394561 0.3861792544
C 3.1141914221 -2.5621610697 0.5864536643
C 4.2004567397 -3.0717543197 -0.112918222
C 4.9082989713 -2.2739251223 -1.0174675508
C 4.5702621236 -0.9266945816 -1.1746474307
C 3.5006427866 -0.4282660326 -0.456062089
H 2.6535561537 -3.2057780124 1.320679766
H 4.5130545874 -4.0960300637 0.0564820217

H 5.1525592574 -0.2850678078 -1.8271849118
C 1.5454143348 -0.4116508746 0.8721530172
C -1.1189348489 -0.5112773177 0.8075526998
C -1.8934092703 -1.6354995699 0.5275118001
C 0.2805392361 -0.958654798 1.0373753489
C -1.7922428624 0.7563617809 0.6407898406
C -3.0736781307 0.8283032552 -0.0002927502
C -1.2689536623 1.9593883824 1.1493786861
H -0.4190767075 1.8842298522 1.7965085505
C 3.9687814257 4.5428429507 -0.5722237071
C 6.012651754 -2.8246195169 -1.7430397458
N 4.4703512284 5.5265908103 -0.9202541053
N 6.9041141321 -3.2711291926 -2.3317251291
C -1.8219873397 3.1879620826 0.8912891269
H -1.3725162577 4.084146401 1.307604819
C -2.9606860048 3.2698888792 0.0791907091
H -3.3766161296 4.2327619739 -0.1982513245
C -3.5752143385 2.1116760074 -0.3303365057
H -4.4639547211 2.1958730271 -0.9340285644
C -3.812479953 -0.3899399962 -0.2939315902
C -3.1800953323 -1.5930458893 -0.0021143527
H -3.6801007366 -2.535239294 -0.1781415957
C 0.1929273831 -2.4683397053 1.29014764
H 0.9239930422 -2.9884936455 0.6793554167
C -1.1948483991 -2.913396802 0.8348117408
H -1.7298122434 -3.4709001709 1.6114117684
H -1.1511076981 -3.5630352927 -0.0453252481
C 0.4584264817 -2.7821190499 2.7661616164
H -0.2985263376 -2.3092129466 3.4000945721
H 0.4154550984 -3.862030434 2.9377927471
H 1.4382509505 -2.4212330636 3.0896585298
C -5.6785629619 -1.6849217223 -1.2291557848
C -6.1281335287 0.6114649512 -0.6403085884
C -7.0834700193 -1.3081713645 -1.6760877733
H -5.7327604461 -2.371718708 -0.373614636
H -5.0822805813 -2.1670188105 -2.0090817712
C -7.4297791768 -0.183679365 -0.7047154955
H -6.1149110651 1.3509434474 -1.4483339089
H -5.9980274726 1.1357999155 0.3082900547
H -7.0709078214 -0.933564384 -2.704691494
H -7.7691195495 -2.1562981055 -1.6272736675
H -8.26811023 0.4366645519 -1.0283124947
H -7.6694816673 -0.5990712376 0.279377117
N -5.0723827629 -0.4045702141 -0.8343310903

Energy= -1436.0448709
Zero-point correction= 0.498619 (Hartree/Particle)
Thermal correction to Gibbs Free Energy= 0.440577
Sum of electronic and zero-point Energies= -1435.546252
Sum of electronic and thermal Energies= -1435.518890
Sum of electronic and thermal Enthalpies= -1435.517946
Sum of electronic and thermal Free Energies= -1435.604294
Imaginary Frequencies 1

Half-Chair-1-unstable-CAM-B3LYP

C -5.6039812783 -1.3767603499 0.5217539625
C -4.7332962444 -2.4680021708 0.6570269312
C -3.3756349837 -2.3215409818 0.4273423239
C -2.8660473112 -1.0805571542 0.0356665917
C -3.748955092 0.0190194654 -0.0590721188
C -5.107144628 -0.1199681652 0.1713339334
H -5.1348371498 -3.4308293943 0.9516325292
H -2.7260260412 -3.1769462992 0.5640826361
H -5.7818249877 0.7258525489 0.0956499711
C -1.6016639101 0.8339511583 -0.427131188
C -0.6540372571 1.7825318635 -0.8186010629
C -1.056928745 3.0760238035 -1.1043619947

C -2.4084722351 3.4421209425 -1.0214472871
C -3.3735900999 2.4959196772 -0.6714370568
C -2.9649853727 1.203812032 -0.3873824002
H 0.3918323691 1.5207141375 -0.9055234653
H -0.3260000663 3.820180422 -1.3995027367
H -4.4205770238 2.777259778 -0.6396823516
C -1.4863831094 -0.6114482184 -0.1730111161
C 1.0209262811 -0.9855991867 0.097932284
C 1.9062324338 -1.942031883 -0.3793741039
C -0.3454624737 -1.3799011217 -0.1922614596
C 1.5552420158 0.058389512 0.9324488179
C 2.9715265632 0.2166072243 1.0167236931
C 0.7406011252 0.8444104497 1.7783627626
H -0.3298015654 0.7000269614 1.7489938019
C 1.2776121598 1.7422173629 2.6662742173
H 0.6234616092 2.3166190705 3.3137860089
C 2.6686430574 1.885829744 2.7645535258
H 3.0985209026 2.5662204038 3.4917315188
C 3.4888326054 1.1313097548 1.9636017772
H 4.5563871879 1.2003619573 2.1082064495
C 3.8464478174 -0.6460154239 0.2392275486
C 3.2891187201 -1.7910584091 -0.3260403736
H 3.9132471908 -2.5466707857 -0.782778373
C -0.3144554006 -2.8433958324 -0.6526252362
H -0.608737989 -3.490079377 0.1841336832
C 1.1775063793 -3.1070833039 -0.9706774146
H 1.3270991397 -3.1539193801 -2.0556812386
H 1.5410674643 -4.0562101787 -0.5645937304
C -1.1828731331 -3.1552621814 -1.8732737438
H -0.8990223304 -2.509000345 -2.7086499859
H -1.0265486346 -4.1926207046 -2.1836347151
H -2.2468660417 -3.0156949909 -1.6931752218
C -2.7988451425 4.7902308949 -1.3155558921
C -7.0065959942 -1.552225234 0.7627678764
N -3.110276271 5.879404559 -1.5522937818
N -8.1382205629 -1.6965475109 0.9571505211
C 5.7920490793 0.9513602749 0.0679389388
C 6.0473479006 -1.353236042 -0.5917448263
C 6.9163564855 0.8235807467 -0.9573885003
H 6.2163214801 1.2277190317 1.0400179313
H 5.0451717232 1.7021602218 -0.1975086138
C 7.373264435 -0.6205408138 -0.7615193858
H 5.6236648007 -1.5987061046 -1.5758404768
H 6.1348245957 -2.2851372524 -0.0248464167
H 6.5244622013 0.9606774588 -1.9702622989
H 7.7060854516 1.5602254346 -0.7971537549
H 7.956678847 -1.0131462447 -1.5962909368
H 7.9786429632 -0.7083137096 0.146120143
N 5.1898953899 -0.3940221715 0.1114675302

Energy= -1436.0406725
Zero-point correction= 0.499172 (Hartree/Particle)
Thermal correction to Gibbs Free Energy= 0.440366
Sum of electronic and zero-point Energies= -1435.541501
Sum of electronic and thermal Energies= -1435.513367
Sum of electronic and thermal Enthalpies= -1435.512423
Sum of electronic and thermal Free Energies= -1435.600306
Imaginary Frequencies 0

Half-Chair-1-unstable-CAM-B3LYP-MeCN

C -5.5929770601 -1.3851394356 0.5534316995
C -4.7171332664 -2.4786840286 0.6795101618
C -3.3635721663 -2.327790473 0.4417769777
C -2.857733211 -1.078201879 0.054783285
C -3.7516960033 0.0192888481 -0.0387354237
C -5.1083173486 -0.123718909 0.1997613926
H -5.1122189908 -3.4452755613 0.9719544206

H -2.7130143234 -3.184293724 0.5664838168
H -5.7845409662 0.7213063619 0.1238909967
C -1.6107219256 0.8260897737 -0.444775251
C -0.6664936078 1.7775845639 -0.8543491463
C -1.0725536302 3.0674290381 -1.1423618071
C -2.4272635678 3.4322774623 -1.0405785289
C -3.3913003737 2.4909296527 -0.6706526318
C -2.9775251365 1.2004105776 -0.386298524
H 0.3784417204 1.5171997476 -0.9591282849
H -0.345158292 3.8085753881 -1.4546482332
H -4.438776205 2.7694182653 -0.620988207
C -1.4916488616 -0.6051846093 -0.172524752
C 1.0124527899 -0.9867474476 0.1048453402
C 1.9087851716 -1.9600434697 -0.3528981099
C -0.3339933693 -1.376407544 -0.1977166294
C 1.5487045376 0.0718201871 0.9275489443
C 2.9648218039 0.2337270239 1.0114781077
C 0.7335715634 0.864299778 1.7646143586
H -0.337065881 0.7180437095 1.7383142205
C 1.2684199578 1.7706731463 2.6496855853
H 0.6118105909 2.3490761254 3.2919970851
C 2.6573153852 1.9160547081 2.748477627
H 3.0880996868 2.6038656045 3.4683943582
C 3.4800813473 1.1544146671 1.9512237967
H 4.5463555579 1.235575719 2.0962243584
C 3.8445148795 -0.639667706 0.2346995856
C 3.2827902613 -1.8140794683 -0.2939211385
H 3.9096434146 -2.5799321216 -0.7290402922
C -0.3130495709 -2.8326585974 -0.6722711402
H -0.6574789572 -3.4743049118 0.1475923808
C 1.1800150912 -3.1274850513 -0.9356534897
H 1.3676849957 -3.2021447152 -2.0125358104
H 1.5181171202 -4.0686263558 -0.4920675604
C -1.1525029104 -3.1112420889 -1.9196294416
H -0.8297725225 -2.4689130329 -2.7447311639
H -1.0138834878 -4.1508929565 -2.2310990797
H -2.2185238859 -2.9502710336 -1.7625434102
C -2.821842658 4.7745702765 -1.3372577539
C -6.9896888069 -1.5649173431 0.8036235302
N -3.1402691197 5.8623007722 -1.5771905418
N -8.1208125083 -1.7120081608 1.0059209662
C 5.8301523219 0.9266528243 0.1334003261
C 6.0153800195 -1.3503199821 -0.6622644778
C 6.9489201669 0.8247377888 -0.8975521961
H 6.2600376001 1.0872939043 1.1276473236
H 5.1230545671 1.7310659229 -0.0729141972
C 7.3582584734 -0.6417623379 -0.7927265927
H 5.5893281255 -1.5586323944 -1.6513143138
H 6.0757948419 -2.2945332234 -0.1144411837
H 6.5609580162 1.0399416781 -1.898245864
H 7.76095249 1.5227048268 -0.6847909213
H 7.9234831704 -1.0011968638 -1.654467414
H 7.9629555236 -0.8025861233 0.1052507324
N 5.1616067226 -0.387400823 0.0553867845

Energy= -1436.0832247
Zero-point correction= 0.498121 (Hartree/Particle)
Thermal correction to Gibbs Free Energy= 0.439244
Sum of electronic and zero-point Energies= -1435.585104
Sum of electronic and thermal Energies= -1435.556944
Sum of electronic and thermal Enthalpies= -1435.555999
Sum of electronic and thermal Free Energies= -1435.643980
Imaginary Frequencies 0

Half-Chair-1-unstable-wB97X-D

C 5.6212063164 1.3494529153 0.5172061134
C 4.7631661434 2.4576695795 0.6444141619

C 3.3998055518 2.3292713194 0.4152449405
C 2.8711306838 1.0887970514 0.0333235752
C 3.738935496 -0.027413893 -0.0536385683
C 5.1035653066 0.0936000962 0.1764439691
H 5.1799738637 3.4239877411 0.9327324894
H 2.7588588894 3.2011412072 0.5456786674
H 5.7677908945 -0.7697433341 0.1056558183
C 1.5767614237 -0.8085034413 -0.4287861161
C 0.6104864986 -1.743496252 -0.821675188
C 0.9904186454 -3.0515719717 -1.089185064
C 2.3377297027 -3.4459361526 -0.988211353
C 3.3216224947 -2.5114068357 -0.6417125897
C 2.9351406633 -1.2039328873 -0.3750517726
H -0.4358026828 -1.4587970618 -0.9249334114
H 0.2409302071 -3.7874135087 -1.3847792807
H 4.3698859376 -2.8125645073 -0.5968103397
C 1.4856932356 0.6383225106 -0.1765027768
C -1.0136517862 1.0229121901 0.1164649646
C -1.9041535114 1.9843787463 -0.3508752796
C 0.3490646511 1.4194322225 -0.1819717169
C -1.5393442625 -0.0351061015 0.9406801767
C -2.9563904352 -0.2158076561 1.0094821035
C -0.7154253557 -0.8173536764 1.7870198012
H 0.3605201128 -0.6539625198 1.7712725013
C -1.2458044429 -1.7402958829 2.6591815278
H -0.5830393134 -2.3161426218 3.3081920796
C -2.6385699681 -1.9083652908 2.7424255242
H -3.0666565339 -2.610567408 3.4602675054
C -3.4674676218 -1.153006117 1.9434297882
H -4.5409629276 -1.2475140915 2.081902326
C -3.8377418396 0.6477005392 0.2306323238
C -3.2889273177 1.8184168122 -0.3061971092
H -3.9205767681 2.5813454262 -0.7573959263
C 0.3202331938 2.8808572139 -0.6401710019
H 0.6477870931 3.5231455407 0.1967989499
C -1.1763662576 3.1632070573 -0.9172785512
H -1.355085431 3.2491540176 -2.0021040736
H -1.5309356135 4.1030789067 -0.4664167301
C 1.170391449 3.1747823229 -1.8776413309
H 0.8575874454 2.5267197575 -2.7106904062
H 1.0322657666 4.2213036208 -2.1894343655
H 2.2417474481 3.0102891647 -1.7151716237
C 2.7046642652 -4.8090351769 -1.2611588275
C 7.0302807928 1.5059433478 0.7563564974
N 2.9955738202 -5.9081971432 -1.4801108011
N 8.1646674062 1.6352703016 0.948726711
C -5.7641628404 -0.9703727682 0.0402890718
C -6.0356406785 1.3267647675 -0.6239855766
C -6.8871414478 -0.8574285103 -0.9871746546
H -6.1912322886 -1.2382765396 1.0218609678
H -5.0129786827 -1.7305910108 -0.2171695731
C -7.355014095 0.5829999494 -0.7974803259
H -5.6146936423 1.5935347034 -1.6120796738
H -6.1329331932 2.2578980883 -0.0436632808
H -6.488762506 -0.9969392779 -2.0047801679
H -7.6757311761 -1.6051417097 -0.8270983938
H -7.9444459091 0.9716870278 -1.63862239
H -7.9689608861 0.6688921375 0.1131277564
N -5.1667578639 0.3708992451 0.0601941485

Energy= -1435.32199
Zero-point correction= 0.497772 (Hartree/Particle)
Thermal correction to Gibbs Free Energy= 0.438989
Sum of electronic and zero-point Energies= -1434.824218
Sum of electronic and thermal Energies= -1434.796136
Sum of electronic and thermal Enthalpies= -1434.795191
Sum of electronic and thermal Free Energies= -1434.883001
Imaginary Frequencies 0

Half-Chair-1-unstable-wB97X-D-MeCN

C -5.6124819157 -1.3593805183 0.5137813251
C -4.7515090419 -2.4712323014 0.6365118243
C -3.3898858538 -2.3392378741 0.4126845623
C -2.859747774 -1.0893030007 0.0448829316
C -3.7368815685 0.0261963769 -0.0462816254
C -5.1026863259 -0.0982504775 0.1783252442
H -5.1657704071 -3.4420773405 0.9148949141
H -2.7499870761 -3.2133761893 0.5334196812
H -5.7665980443 0.7654471017 0.1029648683
C -1.5769272317 0.7965728723 -0.4409246181
C -0.6095446424 1.7350149322 -0.8410918063
C -0.9886433857 3.0409858438 -1.1096917735
C -2.3392062764 3.4359732431 -0.9989787958
C -3.3260589692 2.5065107982 -0.6432693894
C -2.93867126 1.1986964064 -0.3779217131
H 0.4364412525 1.4515874167 -0.9545126923
H -0.2390190012 3.773625768 -1.4144892287
H -4.374643783 2.8061055557 -0.5869587411
C -1.4859245239 -0.6364968196 -0.17258134
C 1.007602807 -1.0329128649 0.1427723343
C 1.910924083 -2.0129759493 -0.3000030932
C -0.3328579783 -1.4228354602 -0.176568416
C 1.5326711301 0.0411976188 0.9550440822
C 2.9488988405 0.2254170062 1.0240949504
C 0.7081951565 0.8307751177 1.7912624948
H -0.3683748371 0.6680992122 1.7766678718
C 1.2374200073 1.7612444139 2.6612944841
H 0.5725968445 2.342292437 3.3046096096
C 2.6281776431 1.928652959 2.7470105605
H 3.0571638407 2.6369712814 3.4587703417
C 3.4595581694 1.1664361057 1.9512051845
H 4.5322534122 1.2687343091 2.0900387852
C 3.8351423697 -0.6465770714 0.2436656703
C 3.2867181372 -1.8508382225 -0.2488209261
H 3.9227139544 -2.6245609082 -0.6750141767
C -0.3132147897 -2.8762996178 -0.6494011511
H -0.7033806544 -3.51212059 0.1640769354
C 1.1835391553 -3.1953733539 -0.8547148137
H 1.4102614059 -3.3227570087 -1.9257181726
H 1.5033495925 -4.121944576 -0.3540148406
C -1.1207268594 -3.1332215224 -1.9218290784
H -0.7574154742 -2.4913561328 -2.7399650106
H -1.0015745944 -4.1818737902 -2.2345319347
H -2.1937829762 -2.9432378231 -1.7928815148
C -2.7057879292 4.7952868412 -1.2727839522
C -7.0183816272 -1.5200283571 0.7481527055
N -2.9996461859 5.8945495083 -1.4936856397
N -8.1542671604 -1.6520009137 0.9372482115
C 5.7744049784 0.9628751914 0.0855413461
C 6.0016410234 -1.3123178855 -0.6881375779
C 6.8806013082 0.8801469218 -0.9595697671
H 6.2158645978 1.137337782 1.0802192259
H 5.045053793 1.7588560361 -0.1156549351
C 7.3252950723 -0.5745004946 -0.847458696
H 5.5663307806 -1.5536968922 -1.673865739
H 6.0925969488 -2.2509873155 -0.1213619184
H 6.4705043121 1.0786396678 -1.9626325977
H 7.6828921346 1.6046614462 -0.7653027173
H 7.8907387055 -0.9322326825 -1.7182125016
H 7.9516179388 -0.7127496422 0.0479949772
N 5.1334080521 -0.3598706049 0.0173004219

Energy= -1435.3651241
Zero-point correction= 0.496552 (Hartree/Particle)
Thermal correction to Gibbs Free Energy= 0.437575
Sum of electronic and zero-point Energies= -1434.868572

Sum of electronic and thermal Energies= -1434.840437
Sum of electronic and thermal Enthalpies= -1434.839493
Sum of electronic and thermal Free Energies= -1434.927549
Imaginary Frequencies 0

Half-Chair-1-Inv-EZ_isomerization-TS-CAM-B3LYP

C -3.831901034 -3.1582327709 -0.7293838907
C -2.5557870473 -3.5332053818 -0.2452870632
C -1.6973615897 -2.6006403897 0.2864056276
C -2.0929101036 -1.2472156711 0.3570649082
C -3.3849030651 -0.8703187183 -0.1423949711
C -4.2384653973 -1.8255593201 -0.67648881
H -2.2620266118 -4.5758139328 -0.3030665372
H -0.7188101039 -2.910227049 0.6424568772
H -5.2172053074 -1.5486849567 -1.0557710534
C -2.2993200008 1.025512257 0.6289282238
C -2.1515107023 2.4035524078 0.8958034969
C -3.1745269438 3.2684479713 0.5864314901
C -4.3745883997 2.7985686447 0.0015446338
C -4.5377513025 1.4412412524 -0.2741946674
C -3.5151556026 0.5535422599 0.0299480613
H -1.2365924557 2.787517218 1.3391603618
H -3.0725933931 4.3295777019 0.7869119756
H -5.4621548256 1.0937331976 -0.7249272473
C -1.4300373119 -0.0814150415 0.826230317
C 1.1075310126 0.1985603561 0.8196648419
C 2.1509053215 0.1899701748 1.7901722965
C -0.1115907474 -0.0111543779 1.4351536864
C 1.4451325656 0.4535928858 -0.571413304
C 2.8156200294 0.5633092717 -0.9162233499
C 0.4719564143 0.6875896802 -1.5557667056
H -0.570750035 0.5728073502 -1.2917352581
C 0.8361231311 1.0956282746 -2.8214990621
H 0.0684217121 1.2818606269 -3.5646425162
C 2.180226899 1.3258237787 -3.1292657276
H 2.458759778 1.7263021378 -4.0978050797
C 3.1518627673 1.0632165793 -2.1898646184
H 4.1807658654 1.3099100881 -2.416100096
C 3.8424655899 0.2721034438 0.0857536781
C 3.4769715141 0.2646089821 1.452138042
H 4.2307798224 0.1772639697 2.2220304225
C 0.0669256725 -0.1243434353 2.9338181139
H -0.4644494965 0.739726241 3.3566716049
C 1.5824154693 0.0038765366 3.1635864073
H 2.0002549785 -0.8895821165 3.6390553848
H 1.844468465 0.8506891452 3.8065184384
C -0.5634137619 -1.3861936979 3.5278209268
H -0.0588743399 -2.2853526762 3.1626488478
H -0.4856624714 -1.3701398199 4.6176340452
H -1.6166776647 -1.4520586052 3.2527067212
C -5.4167444951 3.726465251 -0.3106806151
C -4.6975348981 -4.1541449982 -1.2798974384
N -6.2556420915 4.4851782249 -0.5634190669
N -5.3922174209 -4.9673391378 -1.7265306289
C 5.6003737451 -0.6604654461 -1.4724910132
C 7.0680247507 -0.2698414957 -1.5179609646
H 5.0368105969 -0.3983659806 -2.358971247
H 5.4924106067 -1.7423063543 -1.3146756937
C 7.4807608781 -0.3391662778 -0.0422565263
H 7.654826344 -0.9333663275 -2.1550851683
H 7.1671137626 0.7489228715 -1.9039716491
H 7.8221365532 -1.3468815513 0.2056584925
H 8.2922078566 0.3482746607 0.2003237027
N 5.1213884797 0.0315508539 -0.2588814955
C 6.198532704 -0.0112691987 0.7462389418
H 5.9947358793 -0.7786290991 1.5007831886
H 6.2482586958 0.9546676926 1.2551298778

Energy= -1435.9900109
Zero-point correction= 0.496778 (Hartree/Particle)
Thermal correction to Gibbs Free Energy= 0.436124
Sum of electronic and zero-point Energies= -1435.493233
Sum of electronic and thermal Energies= -1435.464975
Sum of electronic and thermal Enthalpies= -1435.464031
Sum of electronic and thermal Free Energies= -1435.553887
Imaginary Frequencies 1

Half-Chair-1-Inv-EZ_isomerization-TS-wB97X-D

C -4.0578150394 -2.8399430037 -0.7469680124
C -2.8594588063 -3.3500001162 -0.1808576005
C -1.9319600336 -2.5091025788 0.3970041132
C -2.1796085342 -1.1161456473 0.4318031914
C -3.3873274056 -0.6007088945 -0.1539991754
C -4.3140356003 -1.4649930005 -0.7320040717
H -2.6799295481 -4.4265703866 -0.2116395732
H -1.0121007118 -2.9214697953 0.820227925
H -5.2348257122 -1.0808544322 -1.1770351426
C -2.1304987011 1.1715962467 0.6597728593
C -1.8181391268 2.530940151 0.8976680344
C -2.7028241114 3.5105171676 0.4975012377
C -3.9202924419 3.1754565529 -0.1520023175
C -4.2402091702 1.8370728347 -0.4040739836
C -3.3563466729 0.834915325 -0.0112733271
H -0.8828525819 2.8101206164 1.3912472986
H -2.4738525908 4.5630874681 0.6757598887
H -5.1759138112 1.5902830952 -0.9110396709
C -1.417789293 -0.0269545669 0.9338057944
C 1.1283159151 -0.0423288394 0.9148468278
C 2.1724715333 -0.1561296801 1.8815638549
C -0.101913712 -0.0980141703 1.5457830751
C 1.4735287669 0.1572792699 -0.4847434782
C 2.8473828957 0.1098976078 -0.8420110955
C 0.5202175967 0.4889026343 -1.4664787905
H -0.5351453594 0.4899269732 -1.1972507689
C 0.9187206037 0.8515659459 -2.7400338774
H 0.1643696148 1.1172749626 -3.4828238069
C 2.2812144198 0.9357828706 -3.0579724264
H 2.5965665934 1.304582706 -4.0356647022
C 3.2286735226 0.569496578 -2.1221157835
H 4.2834074348 0.6980592175 -2.3624657054
C 3.8459377124 -0.2944001275 0.1550186664
C 3.4993797875 -0.2363408163 1.5302485524
H 4.2494803046 -0.4003328962 2.3016483189
C 0.0718473246 -0.2164284865 3.0405532328
H -0.3575196815 0.7152444488 3.4544954317
C 1.5934746897 -0.258308321 3.2594582787
H 1.9158090562 -1.1921911365 3.7472018228
H 1.959395134 0.5664825372 3.8914696641
C -0.7052859337 -1.388839026 3.6422024153
H -0.3151770213 -2.3493264116 3.2717912644
H -0.6216130686 -1.3813426909 4.7384134725
H -1.7675500985 -1.3238995941 3.3692379797
C -4.8165922436 4.2191215415 -0.5574612888
C -4.9995758881 -3.7427551082 -1.3425142143
N -5.5344065547 5.0688137894 -0.8850739013
N -5.7546545419 -4.4793672962 -1.8240504157
C 5.4493902901 -1.4364333668 -1.4236134585
C 6.9492993887 -1.2104689802 -1.498740709
H 4.8967999769 -1.1125195842 -2.3060681564
H 5.221944825 -2.5070931108 -1.2663584408
C 7.3769917693 -1.3346221006 -0.0325125201
H 7.4512748941 -1.9320922755 -2.1563559443
H 7.1526455698 -0.199499241 -1.8853070782
H 7.5906677386 -2.3857632232 0.2093170938
H 8.2815947005 -0.7576487878 0.198935827

N 5.0745472369 -0.7057850771 -0.2019639457
C 6.1616339736 -0.8463532847 0.7762399158
H 5.8912924091 -1.5696010866 1.5631699697
H 6.3341942678 0.125687861 1.2623694166

Energy= -1435.2722244
Zero-point correction= 0.495369 (Hartree/Particle)
Thermal correction to Gibbs Free Energy= 0.434689
Sum of electronic and zero-point Energies= -1434.776855
Sum of electronic and thermal Energies= -1434.748672
Sum of electronic and thermal Enthalpies= -1434.747728
Sum of electronic and thermal Free Energies= -1434.837536
Imaginary Frequencies 1

Half-Chair-2-CAM-B3LYP

C 2.3928006082 3.537867962 -0.7161409485
C 1.039732835 3.1750718086 -0.7135507935
C 0.6574508559 1.8603086978 -0.4976265204
C 1.6296740081 0.8864688359 -0.2651205649
C 2.9901873672 1.2566997499 -0.3222459269
C 3.3802292538 2.5679669059 -0.5322577785
H 0.2879851101 3.9365605585 -0.8868231487
H -0.3924074241 1.6078308919 -0.5214930572
H 4.427427059 2.8471673445 -0.5713434761
C 2.934363947 -1.0601438595 -0.0878212404
C 3.4946027841 -2.3305890571 0.0627303567
C 4.8729315775 -2.4816380546 0.0813078766
C 5.7176640884 -1.3711294507 -0.0337292187
C 5.1770087097 -0.0905756066 -0.1653871164
C 3.800840002 0.0509866072 -0.1895861892
H 2.8816245467 -3.2127183384 0.1750560876
H 5.3082100757 -3.4682894147 0.1911478945
H 5.8340340958 0.7686231391 -0.2442006958
C 1.5341392439 -0.5791150441 -0.0942889623
C -0.9912873532 -0.9577773512 0.1420285037
C -1.8031000707 -1.8604645398 -0.524422218
C 0.4061363043 -1.3471823349 -0.0440366001
C -1.6091857612 0.0146009572 0.9901731663
C -3.0287433518 0.1594528249 0.9503230758
C -0.8764743059 0.744665918 1.9558511042
H 0.1969204971 0.6126198629 1.9984186039
C -1.5002672136 1.5713659086 2.8533729586
H -0.9148492202 2.1079363455 3.5926658001
C -2.8995880347 1.6914647215 2.8404642893
H -3.4000491366 2.3113472755 3.5765983104
C -3.6379083777 0.9965432415 1.9166480328
H -4.7148615662 1.0507063222 1.9695588347
C -3.8118072402 -0.6331704478 0.0189368862
C -3.18606637 -1.7074003299 -0.6093104376
H -3.7526822612 -2.4094333447 -1.2053085142
C 0.3675349179 -2.8355133256 -0.3757354964
H 1.2018885911 -3.1147011119 -1.0210152602
C -0.9767194072 -2.9582831073 -1.1344515256
H -1.439600359 -3.943284654 -1.020784762
H -0.8280281862 -2.7878683251 -2.2070290502
C 0.3552450175 -3.7160314716 0.8810711296
H -0.5298850579 -3.500707597 1.4855421495
H 0.3304503571 -4.7743666118 0.6059410934
H 1.2317078956 -3.5471270576 1.5105259782
C 7.1404010852 -1.551514879 -0.0085947631
C 2.7626075373 4.9070035513 -0.931174503
N 8.2878368236 -1.6991086611 0.011620423
N 3.0580411731 6.0123854434 -1.1037913917
C -5.9264531185 -1.2870236393 -1.0503597902
C -5.753703181 0.9605646279 -0.2007756382
C -7.2448241108 -0.5546494041 -1.2696550995
H -6.0447702278 -2.2663076594 -0.5763956048
H -5.4203821527 -1.4410524866 -2.0143740919

C -6.7909942513 0.9028152724 -1.3203937594
H -4.9921756464 1.7280984831 -0.3548551442
H -6.256941445 1.1736588636 0.7493240707
H -7.7555970787 -0.8868821473 -2.1753287485
H -7.9182416865 -0.7148292554 -0.4217580788
H -6.3211852497 1.1165166047 -2.285782478
H -7.5996326095 1.6211509668 -1.1712356485
N -5.1498691023 -0.3851652143 -0.1954814244

Energy= -1436.0454829
Zero-point correction= 0.499593 (Hartree/Particle)
Thermal correction to Gibbs Free Energy= 0.441000
Sum of electronic and zero-point Energies= -1435.545890
Sum of electronic and thermal Energies= -1435.517810
Sum of electronic and thermal Enthalpies= -1435.516866
Sum of electronic and thermal Free Energies= -1435.604483
Imaginary Frequencies 0

Half-Chair-2-CAM-B3LYP-MeCN

C 2.3796512571 3.5270478387 -0.7267843489
C 1.0246911439 3.1615179208 -0.7343101004
C 0.6474864062 1.8469784727 -0.5149969731
C 1.6234042171 0.8747814554 -0.2715389992
C 2.9850718776 1.2535253243 -0.3208517834
C 3.373879358 2.5649553428 -0.5323454846
H 0.2699559795 3.9179174448 -0.9183826328
H -0.4018272834 1.5932259919 -0.5506991388
H 4.4217757163 2.8435863854 -0.5638354024
C 2.9313138561 -1.0620752279 -0.0820870874
C 3.4964507985 -2.3347190066 0.060824754
C 4.8739774384 -2.4813309037 0.0822075492
C 5.714198457 -1.3629756356 -0.0245172044
C 5.1748110355 -0.0810385024 -0.1535095959
C 3.7977876477 0.053021318 -0.1807976352
H 2.8879562487 -3.2211939412 0.1620232767
H 5.3104201891 -3.4683714186 0.1865456569
H 5.8251877068 0.7836962837 -0.2294395158
C 1.5341087823 -0.5854230566 -0.0932659131
C -0.9873222547 -0.9682563532 0.1511894883
C -1.8108486112 -1.8805200657 -0.5022444435
C 0.3987815264 -1.3586623052 -0.0456390165
C -1.6028385645 0.0160322646 0.9918011981
C -3.0220986399 0.1720825687 0.9480578846
C -0.8687245709 0.7444849914 1.9574562605
H 0.2033742043 0.6027737021 2.0093497131
C -1.4888366234 1.5790201454 2.8534277458
H -0.9008063081 2.1116324481 3.5942544343
C -2.8862272408 1.7108963976 2.8353062567
H -3.3856785591 2.3387341906 3.5657205801
C -3.627257065 1.0174961665 1.9089391219
H -4.7031896985 1.0901524892 1.9576968853
C -3.8116342381 -0.6240021279 0.0146105504
C -3.1884585487 -1.7250466584 -0.587091886
H -3.7597259107 -2.4327997911 -1.1717661847
C 0.3648705394 -2.8466727899 -0.3776174161
H 1.1914040541 -3.1233458847 -1.0332923289
C -0.9912652674 -2.9827670801 -1.1096228592
H -1.4510782547 -3.9660641057 -0.9760112386
H -0.863370957 -2.8221797521 -2.1860792317
C 0.3789553391 -3.7251169517 0.8810107388
H -0.5093428799 -3.533698615 1.4899054256
H 0.377255009 -4.7823652854 0.6006894749
H 1.2549590045 -3.5398903677 1.5069779573
C 7.1348139978 -1.5372285009 0.0030120989
C 2.7452347517 4.8936201292 -0.9457237232
N 8.2838751963 -1.6789466278 0.0248654134
N 3.0400472836 5.999456591 -1.1224556448

C -5.8995903269 -1.2619424343 -1.1108907709
C -5.7797163652 0.955076583 -0.1566666016
C -7.2269837512 -0.5406230927 -1.3078155409
H -6.0057867202 -2.2510979675 -0.6564261986
H -5.3863543551 -1.3866493449 -2.0732398616
C -6.8048485112 0.925967925 -1.2860481543
H -5.045556149 1.7558053136 -0.2613734905
H -6.2944536478 1.0827487481 0.8015179478
H -7.7196322391 -0.8412242711 -2.234386988
H -7.9045089375 -0.752606142 -0.4745734112
H -6.3301102828 1.1961043003 -2.2347956829
H -7.6297230967 1.6179538684 -1.1045822697
N -5.126507353 -0.3670524836 -0.2360446939

Energy= -1436.086205
Zero-point correction= 0.498754 (Hartree/Particle)
Thermal correction to Gibbs Free Energy= 0.440396
Sum of electronic and zero-point Energies= -1435.587451
Sum of electronic and thermal Energies= -1435.559413
Sum of electronic and thermal Enthalpies= -1435.558469
Sum of electronic and thermal Free Energies= -1435.645809
Imaginary Frequencies 0

Half-Chair-2-wB97X-D

C 2.3418339691 3.5352672457 -0.6949012169
C 0.990960461 3.1487007806 -0.7366084388
C 0.6240829775 1.8217625151 -0.547720928
C 1.6089460787 0.8597167883 -0.2961478222
C 2.9673058477 1.2510861957 -0.3142755882
C 3.3427345793 2.5754584597 -0.4975579538
H 0.2253941723 3.9032616847 -0.9236161032
H -0.4273147065 1.5493644188 -0.6091503633
H 4.393280152 2.8715496615 -0.5047919492
C 2.9356848131 -1.073912187 -0.1085797813
C 3.5090249264 -2.3442914459 0.0296414667
C 4.8922336403 -2.4799598998 0.0731848922
C 5.729090642 -1.3545482233 -0.0065689837
C 5.173632414 -0.0746513132 -0.1296315898
C 3.7920746153 0.0517245149 -0.1790257857
H 2.9007993321 -3.2420302517 0.1125103993
H 5.3383005823 -3.4705526951 0.1745195841
H 5.8238781675 0.8003474258 -0.1837203487
C 1.530739061 -0.6072580435 -0.131583348
C -0.9890906739 -0.9875830223 0.1266518499
C -1.812381809 -1.8966983571 -0.5235381355
C 0.4037350791 -1.3822663025 -0.0745129434
C -1.5909438584 -0.0002777564 0.9709077651
C -3.0113381106 0.1642241953 0.9337639943
C -0.8398254662 0.7299973646 1.9273160335
H 0.2392654308 0.5824130065 1.9691507867
C -1.447933476 1.5818885424 2.8182490418
H -0.8466013684 2.1228411375 3.5516930419
C -2.8486682787 1.7226148486 2.809506354
H -3.3402531507 2.3635875425 3.5438580537
C -3.6044513339 1.023403162 1.8963746786
H -4.6865690515 1.0995490068 1.9580916079
C -3.8093633762 -0.6312714767 0.009680666
C -3.197346461 -1.7317093521 -0.6013756462
H -3.775923298 -2.442547538 -1.1884002605
C 0.3594044627 -2.8735958889 -0.3812539216
H 1.1902861395 -3.1656038133 -1.0374029174
C -0.993391149 -3.0069792591 -1.1216048246
H -1.4620697847 -3.9941275882 -0.9882326832
H -0.857254714 -2.8495384118 -2.204532824
C 0.3722322629 -3.7236979943 0.8956006497
H -0.5105426745 -3.4990314394 1.5137215759
H 0.3542080415 -4.7953748986 0.6461986265

H 1.2629366039 -3.5267768138 1.5092115964
C 7.1569715699 -1.5183643627 0.044211896
C 2.696259351 4.9167346882 -0.8800574197
N 8.3060339619 -1.6529911437 0.0852260993
N 2.9777805715 6.0299272549 -1.0279159672
C -5.9297202743 -1.259284607 -1.0584507218
C -5.7408167381 0.9778100461 -0.1955520319
C -7.2465202093 -0.5192235686 -1.2625343292
H -6.0502415272 -2.2409392584 -0.5733290406
H -5.4365339906 -1.4299633346 -2.0348181795
C -6.7882482656 0.9362041075 -1.3053172113
H -4.9809517855 1.7588472714 -0.3435367683
H -6.2387258843 1.1734858856 0.769689048
H -7.7716509524 -0.8452509089 -2.1703460987
H -7.9187856589 -0.6839686632 -0.4054787985
H -6.3226286248 1.1586682026 -2.2786882353
H -7.5970767187 1.661740859 -1.1436322385
N -5.1332345355 -0.3585659932 -0.22901131

Energy= -1435.3265311
Zero-point correction= 0.498304 (Hartree/Particle)
Thermal correction to Gibbs Free Energy= 0.439855
Sum of electronic and zero-point Energies= -1434.828227
Sum of electronic and thermal Energies= -1434.800213
Sum of electronic and thermal Enthalpies= -1434.799269
Sum of electronic and thermal Free Energies= -1434.886676
Imaginary Frequencies 0

Half-Chair-2-wB97X-D-MeCN

C 2.321402214 3.5258761521 -0.7030248335
C 0.9691931824 3.1353838218 -0.7505259669
C 0.6089325899 1.8083200591 -0.5572974168
C 1.598832782 0.8484230213 -0.2985302452
C 2.9577069294 1.2497180668 -0.3124202859
C 3.3301338549 2.5748238946 -0.4977730319
H 0.1993677878 3.8839926011 -0.9460239151
H -0.4416750918 1.5341337777 -0.6270801412
H 4.3809611116 2.8710104554 -0.5001814752
C 2.9308462631 -1.0740296187 -0.1024978408
C 3.510749068 -2.345997796 0.0262802511
C 4.8932896535 -2.4756908884 0.0689183526
C 5.7241764144 -1.3419470323 -0.0048329314
C 5.1686511575 -0.0611578535 -0.1233900753
C 3.7859474527 0.0564449414 -0.1720380312
H 2.9088476497 -3.2489092002 0.0990274255
H 5.3416411574 -3.4662981904 0.1634055403
H 5.8110164627 0.8199861262 -0.1763080565
C 1.5285562925 -0.6132242867 -0.12778621
C -0.9863416456 -1.0009971793 0.1404299621
C -1.8215388137 -1.9206808329 -0.4957784505
C 0.3947349642 -1.3950028856 -0.0737740367
C -1.5858116257 -0.0026999449 0.9783392785
C -3.00581452 0.1704628078 0.9385023738
C -0.8343411384 0.7266003646 1.9348915162
H 0.2440203417 0.5729366968 1.9846983946
C -1.4407007236 1.5830337284 2.8262185662
H -0.8377785491 2.1207473827 3.5617007189
C -2.8397945158 1.7313985598 2.8139918167
H -3.3313905026 2.3775620588 3.5443380317
C -3.5971855992 1.0339317468 1.8971025518
H -4.6786926365 1.1230786184 1.9548471679
C -3.8096204763 -0.6266843653 0.0101569045
C -3.2014071498 -1.7548042407 -0.5725983528
H -3.7846520005 -2.4721279955 -1.147359275
C 0.3567873116 -2.885484088 -0.3828506537
H 1.1792087667 -3.1735163268 -1.0506364851
C -1.0088974551 -3.0339312447 -1.092620564

H -1.4725562816 -4.0197893919 -0.9385940203
H -0.896899103 -2.8866548561 -2.1794668114
C 0.4001539 -3.7343939502 0.8945637731
H -0.4850697039 -3.5369195459 1.519370979
H 0.4075561606 -4.8045423055 0.6384206115
H 1.2906553077 -3.5203021298 1.5031187567
C 7.1501788293 -1.4991498 0.043849413
C 2.6701098363 4.9051235786 -0.8941279458
N 8.3009281211 -1.627732151 0.0828318192
N 2.949517538 6.0189823078 -1.0479338649
C -5.9027047038 -1.2329022144 -1.1160765497
C -5.7532466708 0.9772055182 -0.1619684115
C -7.2235410755 -0.4975140066 -1.3051675741
H -6.0180245065 -2.2222864598 -0.6475509799
H -5.3987485181 -1.3791580588 -2.0886851908
C -6.7875909429 0.9640449592 -1.2822312907
H -5.0133933558 1.7830262136 -0.2661726292
H -6.2615406036 1.0990019538 0.808623954
H -7.7295915265 -0.7938153864 -2.2338181365
H -7.9030755736 -0.7056596673 -0.463409387
H -6.3143463005 1.2351128754 -2.239575152
H -7.6085767947 1.6687824749 -1.0923009614
N -5.1081591864 -0.3407349598 -0.2655800192

Energy= -1435.3676383
Zero-point correction= 0.497326 (Hartree/Particle)
Thermal correction to Gibbs Free Energy= 0.439038
Sum of electronic and zero-point Energies= -1434.870312
Sum of electronic and thermal Energies= -1434.842342
Sum of electronic and thermal Enthalpies= -1434.841398
Sum of electronic and thermal Free Energies= -1434.928600
Imaginary Frequencies 0

Half-Chair-2-EZ-TS-CAM-B3LYP

C 3.439468206 3.4835150677 -0.1247199056
C 2.134423506 3.5814365962 -0.6642065555
C 1.3902509804 2.45582213 -0.9260739469
C 1.9341021546 1.1818984297 -0.6560298731
C 3.2561066865 1.0866896219 -0.1062976319
C 3.993290206 2.2339082697 0.1518842324
H 1.7271625803 4.5660132134 -0.8678025545
H 0.3877764941 2.5548756746 -1.3339726882
H 4.9944720804 2.1719403233 0.5669538873
C 2.3965733166 -1.0500978407 -0.3685128161
C 2.4059278636 -2.4590501675 -0.2735445303
C 3.5142625336 -3.0995562453 0.2276494857
C 4.6480758726 -2.3685579031 0.6559695371
C 4.6555900434 -0.976140487 0.5793425699
C 3.5453040342 -0.3121293201 0.076946491
H 1.5439599415 -3.040414868 -0.5887436595
H 3.5320670086 -4.1814784206 0.3035450093
H 5.5287086902 -0.4260002734 0.9162134899
C 1.4105527405 -0.1295631235 -0.8136655528
C -1.1083423593 -0.5286959691 -0.7108769502
C -2.1577320535 -0.7549254883 -1.6487635972
C 0.1016155892 -0.4385179131 -1.3681624048
C -1.4403874872 -0.4391874728 0.7016013497
C -2.8098750605 -0.4495097121 1.0803008471
C -0.452630802 -0.420525912 1.6970489436
H 0.5854848372 -0.3914258279 1.3940967823
C -0.7935944457 -0.4732926644 3.0322289277
H -0.0127184639 -0.4678743064 3.7849326029
C -2.1331738862 -0.5766447287 3.4106621025
H -2.4010347637 -0.678309498 4.456552176
C -3.1210089079 -0.562631619 2.4504512797
H -4.1435170727 -0.6928032737 2.7687110943
C -3.8446072511 -0.4198540249 0.0417359421

C -3.47720227 -0.7279091838 -1.2954676873
H -4.2333034409 -0.846406566 -2.0577635565
C -0.0972242635 -0.612608986 -2.8599353524
H 0.1182373006 0.3731743577 -3.2967219723
C -1.5899451268 -0.9464181501 -3.0214048895
H -1.726367785 -1.9891340943 -3.3314863677
H -2.0983732577 -0.3280493265 -3.7665025426
C 0.8622840909 -1.6188857722 -3.4949444508
H 0.6860909008 -2.6269877953 -3.1088063046
H 0.7259521735 -1.6408252596 -4.5790376229
H 1.8964227345 -1.3487384829 -3.2778439619
C 5.7822721873 -3.0668780563 1.175761235
C 4.1833899897 4.6755394894 0.1394821254
N 6.6968077029 -3.6398981321 1.5984294744
N 4.7781973405 5.6471875959 0.3528838471
C -6.1513940141 -0.1460164754 -0.7641285949
C -5.6508568689 0.6103227219 1.4790464372
C -7.4154948572 0.3854046405 -0.0967487425
H -6.2615881509 -1.1623995645 -1.1499057797
H -5.8458396 0.5035769436 -1.5919197294
C -6.8608507812 1.3623808182 0.9366305286
H -4.8834227129 1.2572231036 1.9033775882
H -5.9717799129 -0.1009861832 2.2468926981
H -8.0909310829 0.8477746349 -0.8179153145
H -7.9561479183 -0.4262413137 0.3992319405
H -6.5443091828 2.2923998245 0.45479082
H -7.5708350533 1.6135662026 1.7261700906
N -5.1242871628 -0.1051159476 0.2977483403

Energy= -1435.996805
Zero-point correction= 0.497307 (Hartree/Particle)
Thermal correction to Gibbs Free Energy= 0.437815
Sum of electronic and zero-point Energies= -1435.499498
Sum of electronic and thermal Energies= -1435.471548
Sum of electronic and thermal Enthalpies= -1435.470604
Sum of electronic and thermal Free Energies= -1435.558990
Imaginary Frequencies 1

Half-Chair-2-EZ-TS-CAM-B3LYP-MeCN

C 3.4052120266 3.4950275133 -0.110671608
C 2.080819534 3.5788078637 -0.6178514231
C 1.3493643148 2.4439176264 -0.8687113785
C 1.9194884212 1.1721837093 -0.6198248134
C 3.2617585813 1.0987966773 -0.1031337402
C 3.9902130748 2.2514297118 0.1453141343
H 1.6516018421 4.5577318836 -0.8048945838
H 0.3370390033 2.5265265332 -1.2528069654
H 5.0029452928 2.1998671816 0.5340635805
C 2.4186789337 -1.0428795582 -0.3673365849
C 2.4501173441 -2.4567273096 -0.2921066207
C 3.5787407307 -3.0898360156 0.1676399027
C 4.7179239907 -2.3450230743 0.5740925286
C 4.7094223636 -0.9482367429 0.5191638669
C 3.5758807923 -0.2974551507 0.0579939406
H 1.5859313222 -3.0412905918 -0.5923525625
H 3.6113476819 -4.1727937569 0.2275858827
H 5.5840738383 -0.3889621538 0.8380061866
C 1.4085063495 -0.1391516862 -0.7773891364
C -1.1082159755 -0.571085748 -0.6685397776
C -2.1675189575 -0.8194121915 -1.6137887503
C 0.0867386856 -0.4738407313 -1.3259503568
C -1.4582080683 -0.4758880331 0.7462216151
C -2.8303928694 -0.4533667074 1.1113225109
C -0.4866035452 -0.4923208293 1.7541970383
H 0.5571743897 -0.5018843488 1.4717125024
C -0.8407187963 -0.534074573 3.0897841876
H -0.0680725547 -0.5545811293 3.8515029175

C -2.1844183039 -0.5900574567 3.4508191728
H -2.4718039773 -0.6733794952 4.4932860007
C -3.1610386357 -0.5504515769 2.4739470901
H -4.1903533707 -0.6380018509 2.7837374331
C -3.8607148783 -0.4109942112 0.0552657779
C -3.4792706114 -0.7772899867 -1.2763250675
H -4.2330258767 -0.9162135907 -2.0375497496
C -0.1057359079 -0.6574389622 -2.8209569077
H 0.0596339386 0.331850141 -3.2705628112
C -1.5845038083 -1.0513527741 -2.971233119
H -1.6764514267 -2.1141445274 -3.2248510882
H -2.1095775369 -0.4910700603 -3.7484298441
C 0.8701240372 -1.634023277 -3.4727692192
H 0.7566751945 -2.6397561069 -3.0573613752
H 0.6778443802 -1.6893528946 -4.5479418332
H 1.9050980598 -1.3161229563 -3.3302924328
C 5.8747373005 -3.0286922743 1.0459346877
C 4.140881278 4.6884914775 0.1394539591
N 6.8161488015 -3.5886079494 1.4302033825
N 4.7383326554 5.66285999 0.3423157555
C -6.1304253658 -0.0629997117 -0.8114563738
C -5.6694296872 0.6452059993 1.4614647501
C -7.397396113 0.4751786294 -0.1590516254
H -6.2392538926 -1.0771155618 -1.1990497265
H -5.7957654027 0.5892331345 -1.6233741383
C -6.8546182682 1.4224730352 0.9058185506
H -4.9105748617 1.2731699235 1.925863765
H -6.016409395 -0.0977394356 2.1849607992
H -8.0443027856 0.9634401626 -0.8893648342
H -7.9591582409 -0.3384722486 0.3089280911
H -6.512842588 2.3572658224 0.4512833235
H -7.5767868776 1.6604626744 1.688375099
N -5.1128117899 -0.0378691401 0.2710363549

Energy= -1436.0629656
Zero-point correction= 0.497495 (Hartree/Particle)
Thermal correction to Gibbs Free Energy= 0.439201
Sum of electronic and zero-point Energies= -1435.565470
Sum of electronic and thermal Energies= -1435.537862
Sum of electronic and thermal Enthalpies= -1435.536918
Sum of electronic and thermal Free Energies= -1435.623764
Imaginary Frequencies 1

Half-Chair-2-EZ-TS-wB97X-D

C 3.183293869 -3.5998761056 0.9262037215
C 1.8801497819 -3.4982872775 1.4817240797
C 1.2225327598 -2.287402989 1.534075074
C 1.8573234113 -1.1276628113 1.030038926
C 3.1720718204 -1.2341548333 0.4587178611
C 3.8225353204 -2.4649618312 0.4161382452
H 1.4017102731 -4.4003751491 1.8684417227
H 0.2168638507 -2.2312524924 1.9605079704
H 4.8224908408 -2.5544615368 -0.0146460288
C 2.4584650281 0.9689461993 0.3034261311
C 2.5440111946 2.3288889973 -0.0791737287
C 3.678770109 2.7853789242 -0.7162742569
C 4.7615583816 1.9109991224 -0.9984045531
C 4.6893082236 0.5609930009 -0.6388370269
C 3.5492419522 0.0815532861 0.001553021
H 1.7184482441 3.0167670275 0.1230209022
H 3.7566575872 3.8327331272 -1.014785707
H 5.5237625215 -0.1057783842 -0.8681769876
C 1.4287314217 0.2238526831 0.9373888339
C -1.0416940725 0.7451641967 0.610126332
C -2.1114703791 1.2432957977 1.4135152326
C 0.1257539827 0.7184068326 1.3498441499
C -1.3086254127 0.3645954789 -0.7685531342

C -2.6569928092 0.3823917434 -1.2261074556
C -0.2766952077 0.0561954868 -1.673569904
H 0.7484865004 0.0190872745 -1.3073859201
C -0.5506273444 -0.1600019788 -3.0118215009
H 0.2673856468 -0.3871248853 -3.697876643
C -1.8612769031 -0.0450205834 -3.4889500603
H -2.0718475894 -0.1538325293 -4.5543248475
C -2.8943381903 0.2180202252 -2.6095681743
H -3.8933021613 0.3439360037 -3.0167446913
C -3.7413597271 0.6436610175 -0.268359319
C -3.4158868693 1.2234136587 0.9919372583
H -4.1984767409 1.5589061755 1.6685015869
C -0.1239472039 1.2256854513 2.751106234
H -0.0200651404 0.3334725791 3.3981715717
C -1.5871561766 1.698552366 2.7403327349
H -1.6443977397 2.7991633518 2.7925578946
H -2.1864269044 1.3116388942 3.5779399113
C 0.9015498192 2.2582939124 3.2154627205
H 0.8475388372 3.1699884941 2.6008470738
H 0.7190780649 2.5368418125 4.2633924487
H 1.9186264517 1.8515922067 3.130427348
C 5.9261400168 2.4180155569 -1.6642832029
C 3.8365442012 -4.8758351864 0.8838622533
N 6.8631505575 2.8347370373 -2.2055568896
N 4.3560795631 -5.9120386598 0.8509668107
C -6.095555141 0.6762557039 0.4281339849
C -5.532449642 -0.5846148311 -1.5515688299
C -7.3577978321 0.0844076501 -0.1889361075
H -6.1542784607 1.7643046527 0.5772525145
H -5.8807321721 0.2095392255 1.4045709908
C -6.819916862 -1.1275204053 -0.9430393892
H -4.7875739091 -1.3594496583 -1.7734013714
H -5.7602490455 -0.0438441587 -2.4841663667
H -8.1085997821 -0.1650768593 0.5718979977
H -7.8150941606 0.7995643486 -0.8904035465
H -6.5946209444 -1.9486956159 -0.2445578374
H -7.5062037668 -1.5129816394 -1.7082757875
N -5.0231812018 0.3405171792 -0.5241152

Energy= -1435.2792483
Zero-point correction= 0.495990 (Hartree/Particle)
Thermal correction to Gibbs Free Energy= 0.436560
Sum of electronic and zero-point Energies= -1434.783258
Sum of electronic and thermal Energies= -1434.755381
Sum of electronic and thermal Enthalpies= -1434.754437
Sum of electronic and thermal Free Energies= -1434.842688
Imaginary Frequencies 1

Half-Chair-2-EZ-TS-wB97X-D-MeCN

C 3.3432133651 3.5237009715 -0.1022809659
C 2.0328986819 3.5966893695 -0.6581577997
C 1.3177786548 2.4522379139 -0.9358570109
C 1.8940405765 1.1832662239 -0.6669977081
C 3.2179138951 1.1188855581 -0.0975349488
C 3.9306635404 2.2816734221 0.1770407753
H 1.5978711563 4.577795598 -0.8618053004
H 0.3124812414 2.5246210284 -1.3590991573
H 4.9343957557 2.2351201657 0.6070467578
C 2.3927113765 -1.0303293787 -0.3831604794
C 2.4161475338 -2.4464226502 -0.2939771747
C 3.528475293 -3.0777565469 0.2185328862
C 4.6552265974 -2.3273269905 0.6631145719
C 4.6507889578 -0.9270231805 0.5952319312
C 3.5320604764 -0.2780576205 0.0823220539
H 1.5572030878 -3.0347783244 -0.6265064649
H 3.5559602494 -4.167373729 0.2905812491
H 5.5161766226 -0.359009694 0.9460284074

C 1.397101667 -0.1321283959 -0.8397674654
C -1.1050133246 -0.6041030709 -0.7117527937
C -2.171853778 -0.8651177035 -1.6489239626
C 0.080034511 -0.4794673305 -1.386941639
C -1.431571612 -0.5189368045 0.7100159152
C -2.801933312 -0.5111794589 1.0906092172
C -0.4452878434 -0.5359515079 1.7098507672
H 0.6038515408 -0.5281099964 1.4180471975
C -0.7882517093 -0.6027089887 3.0517042333
H -0.0029518342 -0.6239663794 3.8104468113
C -2.1314330615 -0.685972683 3.4255517994
H -2.4083613035 -0.7944427686 4.4759374349
C -3.1215295936 -0.6397531865 2.4566825463
H -4.1546287824 -0.7491096171 2.7748284437
C -3.8444679579 -0.4499698202 0.0436212311
C -3.4848275071 -0.8293974829 -1.2946609646
H -4.2516840132 -0.9726061525 -2.0529440705
C -0.1227342012 -0.6690653413 -2.8757429548
H 0.015149411 0.3319493475 -3.3263525089
C -1.5944405969 -1.0939650692 -3.0090283332
H -1.6677346449 -2.1685562287 -3.2489852382
H -2.1443226474 -0.5535251832 -3.7923970943
C 0.8789004001 -1.6195444162 -3.5254253039
H 0.8018799652 -2.6292517369 -3.0926305144
H 0.6814549919 -1.6948465839 -4.6052700003
H 1.9105873124 -1.2627483947 -3.3922084627
C 5.7960746879 -3.0091542474 1.188369358
C 4.06205533 4.7271739496 0.1752587765
N 6.721344667 -3.5663265506 1.614758978
N 4.6434673807 5.7070477916 0.3995410003
C -6.1132367023 -0.0332146293 -0.7808715047
C -5.5840907796 0.6709696953 1.4722063066
C -7.3490982515 0.5502982568 -0.1090456178
H -6.2637635151 -1.0507863337 -1.1657487268
H -5.7707534662 0.6063815447 -1.6097507649
C -6.7539306005 1.4847271841 0.9379297953
H -4.7891160963 1.2795406126 1.9198523986
H -5.9407380936 -0.0575551857 2.2157844322
H -8.0001482584 1.0565422934 -0.8334217779
H -7.9315368652 -0.2464639033 0.378785536
H -6.3882177212 2.4107405321 0.4671812743
H -7.4549630815 1.755816765 1.738032865
N -5.079233982 -0.0352116478 0.2777180977

Energy= -1435.3450665
Zero-point correction= 0.495777 (Hartree/Particle)
Thermal correction to Gibbs Free Energy= 0.437162
Sum of electronic and zero-point Energies= -1434.849290
Sum of electronic and thermal Energies= -1434.821682
Sum of electronic and thermal Enthalpies= -1434.820737
Sum of electronic and thermal Free Energies= -1434.907905
Imaginary Frequencies 1

Half-Chair-2-TS-CAM-B3LYP

C 3.2330016493 3.3227690248 -0.3876144711
C 2.406149764 3.4104026884 0.7376270093
C 1.8092001305 2.2740852133 1.2634741081
C 1.9805662482 1.0354242956 0.6477194976
C 2.9599919266 0.9476762554 -0.3650692739
C 3.5500808382 2.0703053578 -0.918130354
H 2.2575491522 4.3724951723 1.2141746898
H 1.2783792792 2.3609918138 2.2000020339
H 4.2840344921 1.9891980393 -1.7122666278
C 2.616641377 -1.2069309866 0.4597347162
C 3.0199687819 -2.5304485617 0.6674911398
C 4.0161703627 -3.0957539396 -0.1188734644
C 4.6452003548 -2.3587226077 -1.1243352983

C 4.31483538 -1.012904589 -1.3018767971
C 3.3375281425 -0.4538220951 -0.5013548447
H 2.623262917 -3.1313929955 1.4714554344
H 4.3231250761 -4.1200426171 0.0592529432
H 4.8380054756 -0.4202494492 -2.0442746421
C 1.5296947661 -0.3329910072 1.0114519616
C -1.1152228544 -0.3948183455 1.1834930605
C -1.9266405479 -1.5239272775 1.1395190857
C 0.2893621521 -0.8390791242 1.3567246907
C -1.7924606949 0.8555127161 0.9121293718
C -3.0966528089 0.8459083319 0.3251281313
C -1.2974177449 2.0972570265 1.3439385078
H -0.3739732527 2.1000920367 1.8852237013
C 3.8217189674 4.5091166748 -0.9378312767
C 5.6555111992 -2.9690391329 -1.9384553563
N 4.2917200636 5.4681353547 -1.3830225221
N 6.4677629091 -3.4641680914 -2.5972271275
C -1.9790946616 3.2764170956 1.1744478812
H -1.5515918757 4.2047103167 1.5393173092
C -3.2453381419 3.266182785 0.5797621112
H -3.8126566956 4.1844256334 0.4729217164
C -3.789856946 2.0693579332 0.1857669984
H -4.8049718738 2.0588335768 -0.1817924815
C -3.7519832788 -0.4108749542 0.0178077912
C -3.210496531 -1.5504351506 0.5878403781
H -3.7165751985 -2.5040615278 0.5243623304
C 0.2202213476 -2.3093485796 1.7822368493
H 0.8113720749 -2.9054641199 1.089584864
C -1.2385829122 -2.7471023813 1.640252499
H -1.6676451671 -3.055503263 2.6011856505
H -1.3571723652 -3.5945225138 0.9579603084
C 0.7592748552 -2.4977580686 3.2033757506
H 0.1490175595 -1.9355552175 3.9167283048
H 0.7258377859 -3.5529262102 3.492124916
H 1.7880720311 -2.1441825097 3.2998021394
C -5.1695446461 0.4078921253 -1.8952153233
C -5.5815946964 -1.7548907368 -0.9145520839
C -5.8388160502 -0.5105942648 -2.9156948088
H -4.2476598933 0.8643878867 -2.2614631915
H -5.8594162378 1.2156900384 -1.6254038466
C -6.5936166207 -1.4949745947 -2.0242639311
H -6.037580206 -2.0831038464 0.0245656655
H -4.8709988518 -2.5321267421 -1.2308656113
H -6.4812629012 0.039003004 -3.6066171635
H -5.0827484704 -1.0402530985 -3.5040315469
H -7.496039218 -1.0269276205 -1.6184370768
H -6.889203664 -2.4125715801 -2.5363014676
N -4.8984046523 -0.4703712764 -0.740615778

Energy= -1436.0053107
Zero-point correction= 0.499123 (Hartree/Particle)
Thermal correction to Gibbs Free Energy= 0.441547
Sum of electronic and zero-point Energies= -1435.506188
Sum of electronic and thermal Energies= -1435.478825
Sum of electronic and thermal Enthalpies= -1435.477881
Sum of electronic and thermal Free Energies= -1435.563764
Imaginary Frequencies 1

Half-Chair-2-TS-CAM-B3LYP-MeCN

C 3.2173595675 3.3180304844 -0.3579648627
C 2.3708248581 3.4035037098 0.7573152612
C 1.7697616968 2.2658757247 1.2699842777
C 1.9561440783 1.024714731 0.6546378743
C 2.9435577127 0.9461487734 -0.3550531299
C 3.5429519346 2.0708954523 -0.8956738764
H 2.2106829475 4.3630234925 1.2361092867
H 1.2220179546 2.3514966885 2.1948189228

H 4.2800661482 1.9888122156 -1.6872817749
C 2.5438570315 -1.2221353408 0.4035203227
C 2.8970909188 -2.5745271891 0.5396231696
C 3.8690082155 -3.136099828 -0.2751635445
C 4.5297117222 -2.3694308112 -1.2423231574
C 4.2511765847 -1.0055221849 -1.3578978009
C 3.2918399212 -0.4539445953 -0.5297887081
H 2.4784133964 -3.2085808335 1.3052041964
H 4.1322218411 -4.1804588971 -0.1494716997
H 4.7903722922 -0.3921895581 -2.07196687
C 1.5039157964 -0.3383863173 1.0085678255
C -1.122395791 -0.3595075065 1.2787854677
C -1.9607046568 -1.4814083554 1.2802523612
C 0.2617527175 -0.8252213124 1.4181246142
C -1.7890399734 0.8915525075 0.9683812344
C -3.0640663545 0.8731439598 0.319288789
C -1.3303635464 2.1326632755 1.4425471399
H -0.4564380159 2.1371033374 2.0647050052
C 3.8141961483 4.5043522856 -0.8916108562
C 5.5182915106 -2.9737606666 -2.0814751573
N 4.2932584991 5.4653986632 -1.3251685259
N 6.3166226847 -3.4641022639 -2.7622760384
C -2.0065663916 3.3112517091 1.2298528969
H -1.6119580132 4.2393742601 1.6313673133
C -3.2270845253 3.2965993738 0.5477100559
H -3.7877185685 4.2131170236 0.3968357764
C -3.7472185282 2.0943453394 0.1276354367
H -4.7362701775 2.0891706706 -0.3049978299
C -3.7150851601 -0.3981753342 0.0231303831
C -3.223864851 -1.5161599597 0.6980422448
H -3.7490090745 -2.4609447177 0.6682098814
C 0.1867104407 -2.286820809 1.8704004259
H 0.6775117521 -2.9081880821 1.1230617698
C -1.2928932965 -2.6745099418 1.8690815204
H -1.6668412357 -2.8401659372 2.8866719868
H -1.4908416542 -3.5890695955 1.3040246172
C 0.8568802574 -2.4944323754 3.2300372862
H 0.3499333397 -1.8990535681 3.9960818641
H 0.7949764747 -3.5461475596 3.526395793
H 1.9093578777 -2.2023171866 3.2225870938
C -5.1102674907 0.3934791323 -1.9289215914
C -5.4560694743 -1.803949508 -0.9796333001
C -5.7130907493 -0.5380805033 -2.9755488471
H -4.2161686513 0.9135641576 -2.2762552368
H -5.8525646085 1.1422562931 -1.6325713912
C -6.4429036909 -1.5679563396 -2.116913533
H -5.9344945577 -2.1229384895 -0.0492980713
H -4.7206121091 -2.5689973177 -1.2597833934
H -6.365797404 -0.0032407299 -3.668385582
H -4.9186837905 -1.0217773281 -3.5528839361
H -7.3762838207 -1.1460200763 -1.7310261591
H -6.6793445715 -2.4919256774 -2.6478397968
N -4.7877881878 -0.5052952435 -0.8028202439

Energy= -1436.0480644
Zero-point correction= 0.498332 (Hartree/Particle)
Thermal correction to Gibbs Free Energy= 0.441080
Sum of electronic and zero-point Energies= -1435.549733
Sum of electronic and thermal Energies= -1435.522413
Sum of electronic and thermal Enthalpies= -1435.521469
Sum of electronic and thermal Free Energies= -1435.606985
Imaginary Frequencies 1

Half-Chair-2-TS-wB97X-D

C 3.126987242 3.3183876187 -0.3470692512
C 2.3341362325 3.351460013 0.8115438702
C 1.7616152059 2.1862131988 1.3089016096

C 1.9209475131 0.9746035916 0.6308197033
C 2.8712785773 0.9346251151 -0.4155696931
C 3.4374820055 2.0881267554 -0.9410085731
H 2.1928818449 4.2970637229 1.3372706
H 1.2560431094 2.2262006913 2.2705106953
H 4.1500072252 2.0445290286 -1.7666790298
C 2.5515594876 -1.2580892419 0.32723381
C 2.9514303736 -2.5961839265 0.4612513452
C 3.9230380444 -3.1299782965 -0.3821417373
C 4.5306339267 -2.3467103987 -1.3726299619
C 4.2008222487 -0.9887655481 -1.4785357701
C 3.2473175297 -0.4615204055 -0.6218947537
H 2.5673550549 -3.2381585964 1.2493501119
H 4.2288677744 -4.1703418639 -0.2604827241
H 4.7060697264 -0.3590463207 -2.213100951
C 1.4852887787 -0.4086032702 0.9479584007
C -1.150733137 -0.4656418627 1.125701656
C -1.9725735301 -1.5893201579 1.0373027007
C 0.2452580048 -0.9278374946 1.2885074924
C -1.8064707086 0.7999225161 0.8709947197
C -3.0896465391 0.8220638921 0.2341153137
C -1.3126916134 2.0221395695 1.3664083714
H -0.4106081894 1.9914045065 1.956274682
C 3.6877882225 4.5350683984 -0.8692706257
C 5.5151691637 -2.9239821127 -2.2468636544
N 4.1338428883 5.5168333341 -1.2904471283
N 6.3047645723 -3.3918549514 -2.952494661
C -1.9730951589 3.2191829863 1.206335954
H -1.5474822153 4.1352897197 1.6214374501
C -3.2165682805 3.2430480872 0.5577042015
H -3.7713799776 4.177438158 0.4544696479
C -3.7631025167 2.0622354273 0.1059269812
H -4.7689031634 2.0839405142 -0.304724341
C -3.7467680866 -0.4247376868 -0.129712336
C -3.2425316273 -1.5870735939 0.4468357577
H -3.7603327983 -2.5394502274 0.3469963665
C 0.1673390331 -2.4100445307 1.6623086414
H 0.735436195 -2.9869294125 0.9226752393
C -1.3002463465 -2.8243145523 1.5328895804
H -1.7317054816 -3.1176719124 2.5050263618
H -1.4428474348 -3.6777831304 0.8529344989
C 0.7482607333 -2.6627396932 3.0553604217
H 0.1661939665 -2.1182456985 3.8153463229
H 0.7121114839 -3.735158814 3.302978447
H 1.7909499373 -2.3220316756 3.131478008
C -5.1030553616 0.4718276203 -2.0536671377
C -5.5383984854 -1.7280369865 -1.1804113081
C -5.7435725732 -0.3976717312 -3.1326319135
H -4.1747407136 0.9608016284 -2.382260228
H -5.810858323 1.2635106374 -1.7534518552
C -6.5191391572 -1.4205439621 -2.3063187904
H -6.0267167327 -2.0824502729 -0.2587888128
H -4.8260529114 -2.5123718539 -1.501355564
H -6.3712678742 0.1852127948 -3.8203632079
H -4.9658936769 -0.9019745136 -3.7280677101
H -7.4398061779 -0.9688536728 -1.903809047
H -6.8033276861 -2.3200921251 -2.868672483
N -4.8417197934 -0.4646001218 -0.9524780831

Energy= -1435.2852325
Zero-point correction= 0.497619 (Hartree/Particle)
Thermal correction to Gibbs Free Energy= 0.440202
Sum of electronic and zero-point Energies= -1434.787614
Sum of electronic and thermal Energies= -1434.760307
Sum of electronic and thermal Enthalpies= -1434.759363
Sum of electronic and thermal Free Energies= -1434.845031
Imaginary Frequencies 1

Half-Chair-2-TS-wB97X-D-MeCN

C 3.130694942 3.3260590229 -0.3800664038
C 2.320575052 3.4045458354 0.7688557943
C 1.7491042699 2.258810712 1.3054476325
C 1.9265321346 1.0163308326 0.6812838267
C 2.8818776609 0.9436052687 -0.3631095497
C 3.4522584582 2.0775417313 -0.9278896322
H 2.1647642615 4.3689326557 1.2557833571
H 1.2294046708 2.3380854161 2.2550433538
H 4.1658144541 1.9990310484 -1.75062481
C 2.509684837 -1.2299431364 0.4071782915
C 2.8607991338 -2.5887483654 0.5263903844
C 3.8075515033 -3.1510370055 -0.3226858929
C 4.4452113329 -2.379558234 -1.3086986321
C 4.1655540862 -1.0105268827 -1.4110459867
C 3.2304872371 -0.4575889744 -0.5487903414
H 2.4559398848 -3.2312738681 1.3029869673
H 4.0701502312 -4.2043915402 -0.2089388769
H 4.6854624874 -0.3899933888 -2.1437404366
C 1.4935479992 -0.3478007038 1.0487607196
C -1.1218400691 -0.3598386805 1.3508140625
C -1.9687695107 -1.4812541048 1.3552025769
C 0.2551383762 -0.8355058811 1.482892542
C -1.7736408046 0.8949028969 1.0199162683
C -3.0322195082 0.8794485527 0.3350973781
C -1.3185363003 2.1360697196 1.506900764
H -0.4605954067 2.134715094 2.1639687151
C 3.6939797237 4.5208368898 -0.9411053177
C 5.408008323 -2.9846505796 -2.1833521246
N 4.1442954266 5.4869980002 -1.395288324
N 6.1835104269 -3.4750891903 -2.890990214
C -1.9820321256 3.3221025448 1.2696199742
H -1.590734885 4.2550889191 1.6821369529
C -3.1851552452 3.311424576 0.5501659944
H -3.7388761591 4.2366729074 0.3774698434
C -3.7042441208 2.106945774 0.1195200586
H -4.6874276011 2.1111792128 -0.3436219854
C -3.6817222061 -0.396901159 0.0311921335
C -3.2229539476 -1.5128112564 0.7474549073
H -3.7581626867 -2.460496833 0.7174025443
C 0.1766217328 -2.292480447 1.9431692997
H 0.6433223826 -2.9220521882 1.1758291876
C -1.3070778405 -2.6647072321 1.970983686
H -1.6750584741 -2.788895418 3.0037583517
H -1.5257456876 -3.600274716 1.43677451
C 0.88633118 -2.5075029532 3.2799295282
H 0.4077653478 -1.9036073276 4.0673586235
H 0.8256760604 -3.565776693 3.5778371463
H 1.9477808162 -2.2228185636 3.2370765565
C -5.0158956399 0.391548661 -1.9598553372
C -5.3847311066 -1.8035624721 -1.025846674
C -5.5964631537 -0.5326720147 -3.0242747721
H -4.1146457958 0.9244218186 -2.2923727844
H -5.7714115589 1.1383308243 -1.6651422272
C -6.3438537512 -1.5654525457 -2.1861996221
H -5.8914747295 -2.1125838138 -0.0989151643
H -4.6471645662 -2.5854313751 -1.2817317767
H -6.236723283 0.0087120683 -3.7339000392
H -4.7844551476 -1.0161234542 -3.5905981136
H -7.2925717662 -1.1428046163 -1.8191155168
H -6.5709256967 -2.4934160189 -2.7279209335
N -4.704698259 -0.5163380289 -0.845383263

Energy= -1435.3287554
Zero-point correction= 0.496996 (Hartree/Particle)
Thermal correction to Gibbs Free Energy= 0.439940
Sum of electronic and zero-point Energies= -1434.831759
Sum of electronic and thermal Energies= -1434.804532

Sum of electronic and thermal Enthalpies= -1434.803588
Sum of electronic and thermal Free Energies= -1434.888815
Imaginary Frequencies 1

Half-Chair-2-unstable-CAM-B3LYP

C 2.8449181173 3.3358109295 1.0268022151
C 1.4795499853 3.0910523358 1.2349239738
C 0.93509189 1.8449717389 0.973268809
C 1.7515218104 0.825346775 0.4794680847
C 3.1333113449 1.0706991196 0.3145779001
C 3.6829980716 2.3145737981 0.5745367112
H 0.8506989734 3.8910964031 1.6084949471
H -0.1170078909 1.6744093081 1.1589792684
H 4.7437394573 2.5015970044 0.4473347528
C 2.7858405797 -1.186577103 -0.1203817608
C 3.132917348 -2.4583369453 -0.582973153
C 4.441710951 -2.7240342581 -0.9491170937
C 5.419954521 -1.7208451005 -0.8841705261
C 5.080599598 -0.431717283 -0.468528647
C 3.7707927281 -0.1737752687 -0.1009969729
H 2.3918833583 -3.2435017503 -0.6707148605
H 4.7202128252 -3.7116401818 -1.2985805462
H 5.8358315821 0.3465081658 -0.4507673756
C 1.4815091329 -0.599699072 0.2262679565
C -1.0667331425 -0.7620597969 0.1798354138
C -1.9731503404 -1.6126803425 0.7947090566
C 0.2860718736 -1.2661871105 0.3601635544
C -1.5847003694 0.3212402614 -0.6085285527
C -2.9880165176 0.5979556078 -0.5805584245
C -0.7641382209 1.1077230401 -1.4501423743
H 0.2756800102 0.8344388034 -1.5592225902
C -1.253442885 2.1971400913 -2.1229775185
H -0.5970176234 2.7784723181 -2.7618317151
C -2.5949969336 2.5730761443 -1.9574656994
H -2.9725926417 3.4766693037 -2.42381344
C -3.4353963544 1.7836229137 -1.2147667953
H -4.45993948 2.0973674034 -1.0970177368
C -3.8939425161 -0.3151215471 0.094545474
C -3.3457234967 -1.3971086495 0.7799280852
H -3.983852414 -2.1147101391 1.2759632513
C 0.1686594462 -2.7127237686 0.8611155963
H 0.2480540026 -3.3971233906 0.0056394884
C -1.2790525553 -2.7888397149 1.4051102206
H -1.2668713416 -2.7073661296 2.4986916785
H -1.778123366 -3.7324694693 1.1653195108
C 1.1642422673 -3.1245484868 1.94617171
H 1.1107467656 -2.4283656442 2.787822485
H 0.9094965138 -4.1210910976 2.3189419219
H 2.1962719221 -3.15051738 1.6031957396
C 6.7693740768 -2.0187568932 -1.2672254916
C 3.3832335051 4.6369873207 1.2982943266
N 7.8577249539 -2.261941333 -1.5762516473
N 3.8148926162 5.6883762581 1.5163077394
C -6.1081065662 -1.0130438737 0.9126130692
C -6.0513556637 0.373692014 -1.0540847581
C -7.5293750153 -0.6475099627 0.5062123534
H -5.8945235185 -0.8471210242 1.9726227037
H -5.9295387417 -2.0764290999 0.6970792717
C -7.3675175562 -0.3991078388 -0.9915596742
H -5.519195445 0.2423986301 -1.9987440484
H -6.2573535754 1.4426384977 -0.9273911287
H -8.2435599061 -1.4375656992 0.7454990228
H -7.8506905028 0.2677909336 1.0131320869
H -7.2759260123 -1.3521842661 -1.5222498907
H -8.1929134588 0.1585949196 -1.4385267774
N -5.2634291065 -0.1548590085 0.0756378902

Energy= -1436.0380974
Zero-point correction= 0.499192 (Hartree/Particle)
Thermal correction to Gibbs Free Energy= 0.440202
Sum of electronic and zero-point Energies= -1435.538905
Sum of electronic and thermal Energies= -1435.510780
Sum of electronic and thermal Enthalpies= -1435.509836
Sum of electronic and thermal Free Energies= -1435.597895
Imaginary Frequencies 0

Half-Chair-2-unstable-CAM-B3LYP-MeCN

C 2.8542714203 3.3385413347 1.0119212319
C 1.487685653 3.0941977192 1.2373402268
C 0.9442495079 1.8478093458 0.9848883719
C 1.7592479699 0.8227192694 0.4869600206
C 3.1427872355 1.073985617 0.3054117249
C 3.6936682778 2.3196215122 0.5537334122
H 0.8604690589 3.8934799694 1.6164244443
H -0.1054039876 1.6775042587 1.1857134117
H 4.7529358447 2.5050244247 0.4100766296
C 2.7826468913 -1.1813419198 -0.1213508408
C 3.1323563761 -2.465943761 -0.5601308235
C 4.4385250945 -2.7350824218 -0.9254365064
C 5.4168966413 -1.7255611414 -0.8797580742
C 5.0839994541 -0.429221827 -0.4795162913
C 3.7744284764 -0.1683029986 -0.1124033576
H 2.3954830773 -3.2569756585 -0.6263204126
H 4.7159665961 -3.729535329 -1.2570470363
H 5.8375270462 0.3513413249 -0.4678747737
C 1.4894212282 -0.591504583 0.2294075147
C -1.0582227255 -0.7763397559 0.1618173223
C -1.9751259163 -1.6464481314 0.7567637821
C 0.2806233603 -1.2624807716 0.369870292
C -1.579860666 0.312104765 -0.6255773904
C -2.9872898201 0.5767450379 -0.6209284987
C -0.7527436852 1.1083920142 -1.4493057524
H 0.2907227234 0.843484732 -1.5445092132
C -1.2382800632 2.1960596143 -2.13283494
H -0.5746106137 2.7821911694 -2.7606077767
C -2.5844868213 2.5573088298 -1.9937000535
H -2.9663619653 3.4539562651 -2.4702660981
C -3.4321165624 1.7566529241 -1.2655944595
H -4.4608387294 2.0639652855 -1.1810915615
C -3.9002051534 -0.352737635 0.0432206191
C -3.3411214571 -1.442863063 0.728502145
H -3.9790678072 -2.1734771882 1.2045491308
C 0.1824573809 -2.698874445 0.8956873026
H 0.3307468172 -3.3900574143 0.0564002904
C -1.2817739795 -2.8177367855 1.3729220778
H -1.3236602783 -2.7545035764 2.4665221192
H -1.7526785791 -3.7637912583 1.0920427665
C 1.1500576727 -3.0532459734 2.0241518754
H 1.0369614574 -2.3513217305 2.8559424385
H 0.9209053492 -4.0553014931 2.3991718069
H 2.1944233058 -3.0464760977 1.7151716253
C 6.7617233973 -2.0271643657 -1.2626003924
C 3.3925217789 4.6378162918 1.2728825729
N 7.8506743927 -2.2723308796 -1.5723272639
N 3.8269293397 5.6907766915 1.4839391702
C -6.0946833629 -1.1252884638 0.8373401591
C -6.0858452422 0.4501943754 -0.9933339078
C -7.5191039702 -0.6888042868 0.5266796011
H -5.8297849982 -1.0372550915 1.8942729218
H -5.9470623513 -2.1701506207 0.536289271
C -7.4173746901 -0.2926027537 -0.9426449021
H -5.6126909958 0.4065191643 -1.9755298633
H -6.2434327676 1.5007519118 -0.7273221933
H -8.2391027472 -1.486933225 0.7165038578

H -7.7954571131 0.1772292848 1.1364284235
H -7.3760937097 -1.1869018814 -1.5724843902
H -8.2420531672 0.3336235041 -1.2885647894
N -5.2554318288 -0.2307486997 0.0207300727

Energy= -1436.0802961
Zero-point correction= 0.498379 (Hartree/Particle)
Thermal correction to Gibbs Free Energy= 0.439586
Sum of electronic and zero-point Energies= -1435.581917
Sum of electronic and thermal Energies= -1435.553827
Sum of electronic and thermal Enthalpies= -1435.552883
Sum of electronic and thermal Free Energies= -1435.640710
Imaginary Frequencies 0

Half-Chair-2-unstable-wB97X-D

C 3.011244572 3.2927880482 0.1829601848
C 1.6432610515 3.1688223922 0.4895629261
C 1.0351534557 1.9216068052 0.5352873795
C 1.7882273543 0.7750737312 0.2533498557
C 3.1735075637 0.9061338889 -0.0076449158
C 3.7877347839 2.1512247615 -0.053720932
H 1.059605629 4.0673704198 0.6956836145
H -0.0207673862 1.8502890414 0.7939707441
H 4.855696399 2.2490087684 -0.2573108444
C 2.7040500824 -1.373754025 0.0751057465
C 2.9719829206 -2.7369505152 -0.1055436984
C 4.255593823 -3.1485272656 -0.4383631866
C 5.286803745 -2.2080099189 -0.6202420997
C 5.0237009615 -0.8384366945 -0.4903399786
C 3.7378795609 -0.4337566816 -0.1544980075
H 2.1856255527 -3.4849138224 0.0000439563
H 4.4730495484 -4.2096783953 -0.5698298209
H 5.8188819987 -0.1109787015 -0.6637050058
C 1.444455091 -0.654480885 0.3209211845
C -1.1045529062 -0.6606576825 0.3473914196
C -2.0468614689 -1.2930221122 1.1500964401
C 0.2159226507 -1.2076867673 0.6129443901
C -1.5669855389 0.2762201593 -0.6401964152
C -2.9408258282 0.6844034436 -0.6271551263
C -0.7185266006 0.796272615 -1.6501175249
H 0.2923647332 0.4021957671 -1.7381260326
C -1.1383569675 1.7842797757 -2.5088335572
H -0.4590229844 2.1661560054 -3.2734089553
C -2.4321147367 2.3196009485 -2.3752223947
H -2.7494193054 3.1581352264 -2.9979914287
C -3.3086200775 1.7691828599 -1.468245524
H -4.2993598427 2.2040920484 -1.3881363402
C -3.891678896 0.0030388699 0.2427366591
C -3.4011314012 -0.969262401 1.1222530634
H -4.0772722525 -1.5218216425 1.7713212664
C 0.0268668622 -2.4973345544 1.4189767906
H 0.0528691905 -3.3575817688 0.7250963809
C -1.4127767729 -2.3688192742 1.9749158727
H -1.3789386425 -2.0692290867 3.0362363121
H -1.981355123 -3.3101917852 1.9303543802
C 1.028693596 -2.7163882566 2.5527076048
H 1.0368511904 -1.8429682276 3.222548779
H 0.7349162944 -3.5965866447 3.1447188792
H 2.0552370847 -2.8751481952 2.2043018688
C 6.6093996109 -2.6565153112 -0.9622221326
C 3.6160169424 4.5962628121 0.1319997159
N 7.6735782242 -3.0208736907 -1.2365974041
N 4.0992682588 5.6476113725 0.0893573026
C -6.1169280098 -0.3247905773 1.2346084599
C -6.0285696008 0.6829734809 -0.9454074463
C -7.516114962 0.1077609442 0.8169837792
H -5.8303036788 0.0154155941 2.2420280015

H -6.0535153729 -1.4298627581 1.2259155534
C -7.4086828417 0.0757133763 -0.704760904
H -5.5642413269 0.3407914651 -1.8816137425
H -6.1207960406 1.7813758229 -0.9947395318
H -8.2952416125 -0.5527897146 1.2204522686
H -7.7245612343 1.1315203706 1.1662438953
H -7.4372466886 -0.965184796 -1.0645139786
H -8.203361487 0.632636231 -1.2195578934
N -5.2413761444 0.2604311061 0.220489147

Energy= -1435.3195071
Zero-point correction= 0.497980 (Hartree/Particle)
Thermal correction to Gibbs Free Energy= 0.439132
Sum of electronic and zero-point Energies= -1434.821527
Sum of electronic and thermal Energies= -1434.793493
Sum of electronic and thermal Enthalpies= -1434.792549
Sum of electronic and thermal Free Energies= -1434.880375
Imaginary Frequencies 0

Half-Chair-2-unstable-wB97X-D-MeCN

C 2.7768631883 3.3498821968 0.9612872514
C 1.4143904511 3.0756123309 1.2065477276
C 0.8965928605 1.8096314472 0.9796131931
C 1.7334104316 0.7944658149 0.4860717223
C 3.11240562 1.0732042311 0.2881542095
C 3.6383456253 2.3395692539 0.5119112023
H 0.7661492235 3.8698915818 1.5813822749
H -0.1540345286 1.6167427438 1.1961941802
H 4.6987596387 2.5456669763 0.3524953763
C 2.7892732432 -1.1962107205 -0.1161148301
C 3.1583700483 -2.4838721006 -0.5426345668
C 4.4713449161 -2.7363262913 -0.9092324259
C 5.4376983092 -1.7081023374 -0.8757738162
C 5.0835187364 -0.4090347212 -0.4879839106
C 3.7661079367 -0.1641121491 -0.1194215353
H 2.4285787687 -3.2921985089 -0.5986547588
H 4.7639315502 -3.7370935488 -1.2324104094
H 5.8272369843 0.3906194995 -0.484702347
C 1.4894409904 -0.6247311809 0.2365236736
C -1.0522748372 -0.8233642342 0.1486469074
C -1.9749810054 -1.6904199223 0.7483226094
C 0.2829925931 -1.3096745772 0.3694559574
C -1.562711518 0.2758195822 -0.6340175049
C -2.9667272635 0.568331647 -0.6068866793
C -0.7272661113 1.0641987909 -1.4627933673
H 0.3152028148 0.7745683353 -1.5836349873
C -1.1973383064 2.1819196012 -2.116536365
H -0.5252336414 2.767371461 -2.7480718239
C -2.5337243224 2.5763006803 -1.9422846351
H -2.9027096607 3.5001311934 -2.3925206484
C -3.3934983467 1.776285327 -1.2189144105
H -4.4192734945 2.11132296 -1.111517883
C -3.8922344075 -0.3620436617 0.0497357229
C -3.3419018483 -1.4700928152 0.7266744555
H -3.9893970555 -2.2029079939 1.2030733081
C 0.1873850437 -2.738619748 0.9059777951
H 0.3586496496 -3.4351872317 0.0658305436
C -1.2840190226 -2.8673296963 1.3572931056
H -1.3484525285 -2.817092064 2.4571265335
H -1.7538933623 -3.8150356386 1.0552617307
C 1.1493872719 -3.0699712048 2.0453674915
H 1.0218734399 -2.3551184011 2.8735169766
H 0.9321694528 -4.0777520863 2.4313958252
H 2.2029048152 -3.0517737769 1.7406810788
C 6.7906127205 -1.9932107904 -1.2580695031
C 3.2879204442 4.6699531986 1.1929636543
N 7.8833942276 -2.2254996938 -1.5664930811

N 3.6984586381 5.7376484081 1.3797502449
C -6.0952673473 -1.1170150128 0.8243811832
C -6.0586392764 0.4968874489 -0.9652673566
C -7.5124631888 -0.6513893093 0.5233329105
H -5.8271686982 -1.0480388584 1.8892939841
H -5.964525056 -2.1678682105 0.5104054818
C -7.4034823604 -0.221255514 -0.9348882878
H -5.5870336192 0.475584415 -1.9570632684
H -6.1956172314 1.548505294 -0.6649971331
H -8.2509096112 -1.4455866192 0.696625337
H -7.7773533126 0.2094847234 1.1575631284
H -7.3774977053 -1.1056256393 -1.5912633006
H -8.2218728285 0.4307638682 -1.2689583318
N -5.2425210676 -0.2321173421 0.0188674606

Energy= -1435.3621301
Zero-point correction= 0.497081 (Hartree/Particle)
Thermal correction to Gibbs Free Energy= 0.438412
Sum of electronic and zero-point Energies= -1434.865049
Sum of electronic and thermal Energies= -1434.837043
Sum of electronic and thermal Enthalpies= -1434.836098
Sum of electronic and thermal Free Energies= -1434.923718
Imaginary Frequencies 0

Half-Chair-2-Inv-EZ-CAM-B3LYP

C 3.4748890921 -3.4702284838 0.0463736017
C 2.1778553691 -3.5886772842 0.6006732794
C 1.4270940829 -2.4742244392 0.8901362092
C 1.9561056204 -1.1913592034 0.6336785868
C 3.2700274399 -1.0752220928 0.0691490081
C 4.0140024577 -2.2114192933 -0.2169363643
H 1.7819899587 -4.5800342197 0.7934990356
H 0.4308777236 -2.5891760613 1.309063226
H 5.0092297758 -2.1336503911 -0.6434787054
C 2.3958492435 1.0494968865 0.3775644027
C 2.3919666723 2.4598445719 0.3059702324
C 3.4883687672 3.1181083443 -0.1986291547
C 4.622633348 2.404128889 -0.6533332276
C 4.6429302879 1.0107560484 -0.5999217892
C 3.5448805588 0.3288831851 -0.0945628638
H 1.5293615636 3.0286325675 0.6417513987
H 3.4960116163 4.201240241 -0.2567673214
H 5.516260443 0.4738381224 -0.9569400921
C 1.4235756051 0.1129101315 0.8197994443
C -1.0975556543 0.4912412594 0.7494800047
C -2.1407061009 0.6902153303 1.7005344006
C 0.1196771205 0.4014347053 1.3949489265
C -1.4388185812 0.4302277481 -0.6620869798
C -2.8110113849 0.4196342026 -1.0160206863
C -0.4703422869 0.4802052488 -1.6767581653
H 0.5749124994 0.4610529576 -1.3981382148
C -0.8439121777 0.5945565811 -2.9992023972
H -0.0806218745 0.6436149896 -3.7681680429
C -2.1941036363 0.7063206162 -3.3438334655
H -2.482788453 0.8782059077 -4.3749227514
C -3.1598177098 0.6207521602 -2.3660393057
H -4.1954428242 0.7793355235 -2.6364045207
C -3.8293400506 0.3197861656 0.0318184561
C -3.4674795805 0.6396720234 1.362536524
H -4.2199279203 0.7104649472 2.1352950945
C -0.0670317125 0.549713342 2.8902294744
H 0.1652135803 -0.4402900609 3.3085981452
C -1.5620644426 0.8618538099 3.0710501729
H -1.7092508765 1.8969139709 3.4015589684
H -2.0559573845 0.2237561585 3.8092910756
C 0.8868471699 1.5574897491 3.5318629019
H 0.6939500303 2.5695312527 3.1645587617

H 0.7620838743 1.5595360734 4.6175040202
H 1.9216904379 1.3036704096 3.2989909296
C 5.7441193558 3.1207642321 -1.175879804
C 4.2260643971 -4.6510592023 -0.2462944244
N 6.6482119167 3.7086083053 -1.600580832
N 4.8268859063 -5.6136224291 -0.4826302571
C -6.1699354855 0.0826946128 0.7600388722
C -5.542125551 -0.9987188752 -1.2777649441
C -7.4305165744 -0.4850208765 0.0819683217
H -6.2742296589 1.135489351 1.0348437485
H -5.9241800077 -0.4789052111 1.6674156783
C -7.0284214993 -0.7011939836 -1.3817620346
H -5.376234495 -2.0172002238 -0.9012774746
H -4.9974536391 -0.8978122618 -2.2078114427
H -7.7021197034 -1.4385504343 0.5409706309
H -8.2850961496 0.184127081 0.1906221548
H -7.5813402822 -1.5114371927 -1.8595631266
H -7.1829719734 0.2093196382 -1.9681937898
N -5.0945614936 -0.0465288012 -0.2405647499

Energy= -1435.9902825
Zero-point correction= 0.496779 (Hartree/Particle)
Thermal correction to Gibbs Free Energy= 0.436379
Sum of electronic and zero-point Energies= -1435.493503
Sum of electronic and thermal Energies= -1435.465264
Sum of electronic and thermal Enthalpies= -1435.464319
Sum of electronic and thermal Free Energies= -1435.553904
Imaginary Frequencies 1

Half-Chair-2-EZ-TS-wB97X-D

C 3.6794273816 2.583121362 1.9896084016
C 2.4238313423 3.1313615966 1.6156063098
C 1.6180346348 2.4972963185 0.6935924939
C 2.0504986226 1.2837875767 0.1086114092
C 3.3170380268 0.7251695586 0.4956717847
C 4.1191736857 1.3803367227 1.4269007203
H 2.1021429367 4.0689555033 2.0734596461
H 0.6518582324 2.9330044864 0.4233415548
H 5.0839378525 0.9637126887 1.7254467508
C 2.3084750706 -0.6889483223 -1.0439415249
C 2.1820590005 -1.8566666116 -1.8336188476
C 3.1909278936 -2.7969459933 -1.8224597453
C 4.3532127673 -2.6137376134 -1.0275659181
C 4.4894738481 -1.4717518386 -0.2310986977
C 3.4786653078 -0.5136566735 -0.2265400517
H 1.2919911852 -2.01994762 -2.4474030844
H 3.1050427344 -3.7009778476 -2.4286448202
H 5.383264039 -1.3441891618 0.3839933133
C 1.4412184974 0.4170887279 -0.8384794152
C -1.0921668622 0.1968806855 -0.8973633905
C -2.154355303 0.6429815019 -1.7411166057
C 0.1221663571 0.6053278255 -1.4177360465
C -1.4090509869 -0.5735395569 0.2953277704
C -2.7775331884 -0.7168717456 0.6468411985
C -0.4313140448 -1.2532245144 1.0462370707
H 0.6191347154 -1.1141124535 0.7932791086
C -0.7998032755 -2.119285576 2.0595390362
H -0.027284181 -2.6469663699 2.6218359473
C -2.1544228025 -2.3635783449 2.323795372
H -2.4444918572 -3.1098344449 3.0656211221
C -3.1262561119 -1.6718904984 1.627230485
H -4.173319931 -1.9182073551 1.7998946126
C -3.8006296296 0.0336515704 -0.0922946884
C -3.4763872645 0.5394334139 -1.379596032
H -4.2433831665 0.9722364635 -2.0188647557
C -0.0843576038 1.3792706924 -2.6981262688
H 0.187917111 2.4219799133 -2.4445047687

C -1.594090022 1.2833600819 -2.9733044063
H -1.7947971016 0.6308456852 -3.8401923502
H -2.0695681701 2.2512820797 -3.1912632565
C 0.8267514817 0.9254898934 -3.8373709816
H 0.6029154737 -0.1116386474 -4.1306453373
H 0.6913791008 1.5702444673 -4.7176566276
H 1.8792255612 0.9705550834 -3.5254007572
C 5.382563722 -3.6122917527 -1.0377165003
C 4.4905101584 3.2669098365 2.9546433502
N 6.2090697335 -4.4255175323 -1.0490832441
N 5.1385526003 3.8249839162 3.7377533283
C -6.1286005396 0.7580320326 -0.4149330872
C -5.395698502 0.3928659336 1.8296566035
C -7.3314901059 0.8795696664 0.5364363121
H -6.3103884071 0.0590767576 -1.2454065148
H -5.8662309452 1.7350614798 -0.8524130081
C -6.8953215487 0.1536382024 1.8126579191
H -5.1675185558 1.4307624909 2.1348004166
H -4.8395112406 -0.2708077142 2.4919876118
H -7.5304401779 1.938495254 0.7562658423
H -8.2458784616 0.4611641951 0.0964678281
H -7.3916640962 0.5328190726 2.7154450742
H -7.0977835139 -0.9265902718 1.7414880659
N -5.0278764775 0.2424257238 0.4116972724

Energy= -1435.2724785
Zero-point correction= 0.495200 (Hartree/Particle)
Thermal correction to Gibbs Free Energy= 0.434790
Sum of electronic and zero-point Energies= -1434.777279
Sum of electronic and thermal Energies= -1434.749083
Sum of electronic and thermal Enthalpies= -1434.748139
Sum of electronic and thermal Free Energies= -1434.837688
Imaginary Frequencies 1
